# An old confusion: Entomophthoromycosis versus mucormycosis and their main differences

**DOI:** 10.3389/fmicb.2022.1035100

**Published:** 2022-11-03

**Authors:** Jaime David Acosta-España, Kerstin Voigt

**Affiliations:** ^1^Jena Microbial Resource Collection, Leibniz Institute for Natural Product Research and Infection Biology – Hans Knöll Institute, Jena, Germany; ^2^Institute of Microbiology, Friedrich Schiller University Jena, Jena, Germany; ^3^Postgraduate Program in Infectious Diseases, School of Medicine, Pontificia Universidad Católica del Ecuador, Quito, Ecuador

**Keywords:** basidiobolomycosis, conidiobolomycosis, zygomycosis, phycomycosis, mucoralomycosis, entomophthoramycosis

## Abstract

Fungal diseases were underestimated for many years. And the global burden of fungal infections is substantial and has increased in recent years. Invasive fungal infections have been linked to several risk factors in humans which basically depend on the individual homeostasis of the patients. However, many fungi can infect even apparently healthy people. Knowledge of these pathogens is critical in reducing or stopping morbidity and/or mortality statistics due to fungal pathogens. Successful therapeutic strategies rely on rapid diagnosis of the causative fungal agent and the underlying disease. However, the terminology of the diseases was updated to existing phylogenetic classifications and led to confusion in the definition of mucormycosis, conidiobolomycosis, and basidiobolomycosis, which were previously grouped under the now-uncommon term zygomycosis. Therefore, the ecological, taxonomic, clinical, and diagnostic differences are addressed to optimize the understanding and definition of these diseases. The term “coenocytic hyphomycosis” is proposed to summarize all fungal infections caused by *Mucorales* and species of *Basidiobolus* and *Conidiobolus*.

## Highlights

- Systematic comparison between basidiobolomycosis (BM), conidiobolomycosis (CM) and mucormycosis (MM).
- World distribution of BM, CM and MM based on systematic review of case reports.
- Comprehensive pathophysiology of the role of virulence factors.

## Introduction

Fungal diseases have been underestimated and neglected worldwide (Rodrigues and Nosanchuk, 2020). Yet the fungi that infect humans are silent killers or cause misery for millions (Denning, 0000). The Global Action Fund for Fungal Infections (GAFFI) estimates that 300 million people suffer from severe fungal infections and 25 million are at high risk for death or loss of vision (fungal keratitis) (Global Action For Fungal Infections, 2020). According to the World Health Organization, not all countries report data on fungal diseases, incidence, resistance, and public health impact are poorly known (World Health Organization [WHO], 2022). The impact on mortality from fungi is estimated at 1.5 million people per year (Bongomin et al., 2017). The attention is focused on the most common diseases such as cryptococcal meningitis, invasive candidiasis, *Pneumocystis jirovecii* pneumonia, invasive aspergillosis, fungal asthma, and fungal keratitis (Bongomin et al., 2017).

In any case, there are several fungal microorganisms that can infect humans. These need to be carefully studied in the light of the factors which contribute to the increased incidence of invasive fungal infections. These risk factors include treatment with immunomodulatory drugs, patients with HIV/AIDS, patients with oncohematological diagnoses, diabetes, COVID -19 pandemic, antifungal prophylaxis, etc., (Cornely et al., 2008; Limper et al., 2017; Peter Donnelly et al., 2020; Saud et al., 2020; Hoenigl et al., 2022; Thomas-Rüddel et al., 2022). However, fungi do not only affect patients with risk factors. For example, dermatophyte onychomycosis, conidiobolomycosis, basidiobolomycosis, coccidioidomycosis, fungal keratitis, etc., can also occur in patients with apparently normal immunity (al Jarie et al., 2011; Weiblen et al., 2016; Lipner and Scher, 2019).

The list of fungal species, which become pathogenic, is long. Mucormycosis associated with COVID -19 (CAM) gained worldwide attention in early 2021 (Banerjee et al., 2021; Pal et al., 2021; Skaria et al., 2022). This was a disfiguring superinfection caused by *Mucorales* with high mortality that occurred in patients with SARS-CoV-2 infection with multiple risk factors (Patel et al., 2020; Aranjani et al., 2021; Mathew et al., 2021; Biswal et al., 2022; Hoenigl et al., 2022). In past and still present times, the terms “zygomycosis” and “zygomycetes” were used which resulted in confusion that affects *Basidiobolus* spp. and *Conidiobolus* spp. that were included in the old taxonomic classification. This led to the abolition of the class Zygomycetes and the misuse of the term zygomycosis.

**FIGURE 1:**
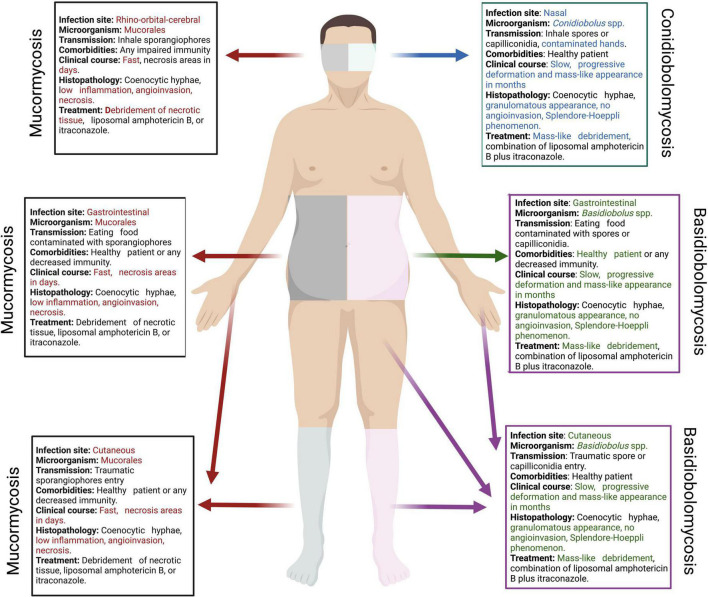
Main differences between basidiobolomycosis, conidiobolomycosis and mucormycosis. On the left, the main differences of the main forms of mucormycosis (red), conidiobolomycosis (blue) and basidiobolomycosis (green) are explained. This image was created with Biorender.com.

In the clinical field, the histopathology of the infected tissue plays a fundamental role in the diagnosis of patients infected with *Mucorales*, *Basidiobolus* spp. and *Conidiobolus* spp. since hyphae with sparse or non-septate hyphae (coenocytic) can be found in all of them. The occurrence of coenocytic hyphae was associated with zygomycosis for many years. However, these morphologically similar fungi had significant phylogenetic differences with dramatic consequences on the clinical outcome. The detailed differences are analyzed in this manuscript, and a summary is shown in Figure 1.

## Definitions of terms and pathogens associated with entomophthoromycosis and mucormycosis

Formerly, the fungal species causing entomophthoromycosis and mucormycosis were assigned to the class *Zygomycetes* (Voigt et al., 1999). This class included the orders *Entomophthorales* and *Mucorales*. This classification primarily included microorganisms with frequent asexual reproduction by sporangia, sexual reproduction by zygospores, and the formation of non-septate (coenocytic) hyphae, to name a few. These definitions led to the diagnosis of zygomycosis in patients with hyphal-infected tissue with little or no septation. This term also included the subtypes referred to as mucormycosis and entomophthoromycosis. And entomophthoromycosis included the terms “conidiobolosis” and “basidiobolosis” (Costa, 2012).

The phylum *Zygomycota* was abandoned (Hibbett et al., 2007). It was replaced by the phylum *Mucoromycota* and *Zoopagomycota*, based on the phylogenetic analyzes of Spatafora et al. (2016). These changes were supported by a higher number of loci and taxa in molecular phylogenetic analyzes within members of the phylum *Zygomycota*. As a result, the clinical names for infections with these pathogens changed. The order *Mucorales* was included in the phylum *Mucoromycota*, and patients infected with *Mucorales* develop mucormycosis (Chibucos et al., 2016; Jeong et al., 2019). The order *Basidiobolales* and *Entomophthorales*, on the other hand, belong to the subphylum *Entomophthoromycotina*, so patients infected with these fungi suffer from entomophthoromycosis. More specifically, the *Basidiobolales* cause basidiobolomycosis and the *Entomophthorales* infection produce conidiobolomycosis (El-Shabrawi et al., 2014).

**FIGURE 2:**
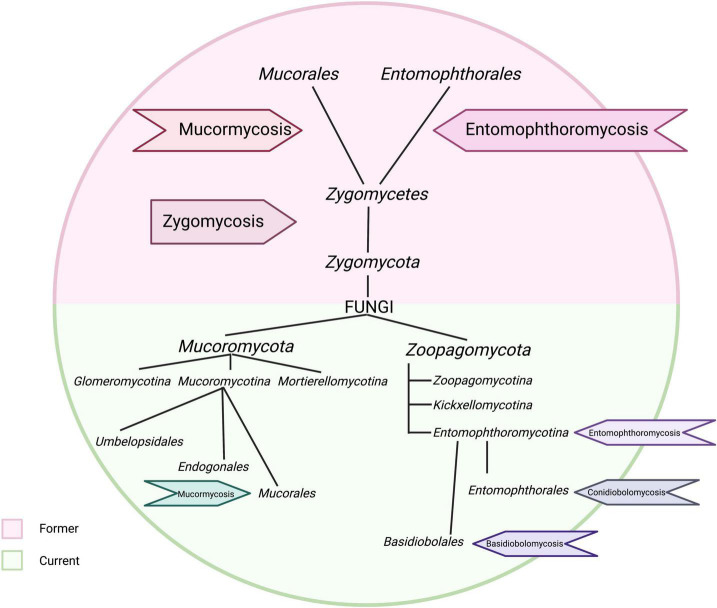
Taxonomy and terms used in mucormycosis and entomophthoromycosis. This image shows the old nomenclature for zygomycosis in the light purple section. The current taxonomic classification and terminology is indicated in the light green section. This image was created with Biorender.com.

With the new classification of the *Mucorales* and *Entomophthorales*, the term zygomycosis became obsolete (Figure 2). However, much of the scientific literature, especially case reports, retained or confused the definitions. The pathogens of the order *Mucorales* (subphylum: *Mucoromycotina*) are associated with mucormycosis and belong to the genera *Rhizopus*, *Mucor*, *Lichtheimia*, *Cunninghamella*, *Apophysomyces*, *Rhizomucor*, *Saksenaea*, *Synchephalastrum*, *Thamnostylum*, *Cokeromyces*, *Actinomucor*, *Cokeromyces* (Acosta-España and Voigt, 2022). Entomophthoromycosis is caused by species of orders *Basidiobolales* and *Entomophthorales* which are *Basidiobolus* and *Conidiobolus*, respectively (both in the subphylum: Entomophthoromycotina). The main species causing basidiobolomycosis is *B. ranarum*, but cases of *B. meristosporus*, *B. omanensis*, and *B. haptosporus* have also been reported (Kamalam and Thambiah, 1984; Sanglard et al., 2017; Bshabshe et al., 2020; Al-Hatmi et al., 2021). Finally, conidiobolomycosis is caused by *Conidiobolus coronatus, Conidiobolus pachyzygosporus, Conidiobolus lamprauges*, and *Conidiobolus incongruus* (Walsh et al., 1994; Wüppenhorst et al., 2010; Kimura et al., 2011; Das et al., 2019; Stavropoulou et al., 2020).

## Ecology and transmission of *Basidiobolales*, *Entomophthorales*, and *Mucorales*

### Basidiobolales

The fungi that cause mucormycosis and entomophthoromycosis are saprotrophic microorganisms from the environment. *Basidiobolus* sp. was originally isolated from the gut contents of frogs (Feio et al., 1999; Gugnani, 1999). And frogs normally feed on insects. Therefore, this fungus has been isolated from the entire bodies of insects in various environments. Also, in the excreta or gut contents of amphibians and reptiles and other animals (Feio et al., 1999; Hung et al., 2020). In addition, the isolation from plant debris and soil has also been reported (Chetambath et al., 2010). Basidiobolomycosis infection occurs after a scratch or puncture from an insect, plant, or other object that carry fungi (Clark, 1968). The gastrointestinal tract has also been found to become infected after ingestion of soil, animal feces, or food contaminated with *Basidiobolus ranarum* (AlSaleem et al., 2013).

### Entomophthorales

In a study over a 9-months in irrigated vegetable fields and citrus orchard soils *Conidiobolus coronatus* was the predominant species, accounting for 31.4% of all fungal isolates (Ali-Shtayeh et al., 2002). This confirms that the ecological distribution of this fungus as a primary saprobe relies on soil humus, decaying plant material in hot and humid climates. Occurrence in insects, reptiles, amphibians, and mammalians like dogs, deers, horses, llamas, non-human primates, and sheep has been reported (Weiblen et al., 2016). Conidiobolomycosis may occur by inhalation of spores, insect bites, or insertion of dirty hands into the nostrils (Thomas et al., 2006). An association of conidiobolomycosis with gastrointestinal transmission was not found: An extensive literature search for case reports in Academic Google, ScienceDirect, PubMed, and Taylor and Francis revealed no human case reports to support the mechanism of gastrointestinal transmission.

### Mucorales

Mucorales are found in a wide variety of organic substrates, including bread, vegetable matter (decomposing fruits), crop residues, soil, animal excrement, and compost piles (Hoffmann et al., 2013). Environmental studies have shown the presence of various species in buildings, contaminated medical material (tongue depressors, needles, etc.), ventilation systems, and flowerpots in hospitals (Ribes et al., 2000; Cornely et al., 2019; Biswal et al., 2022). Transmission occurs *via* entry of sporangiospores through the respiratory tract. This may allow inoculation in the naso-facial area or reach the lower respiratory tract. On the other hand, trauma with contaminated materials can lead to infection of the affected area. Transmission through contaminated food with concomitant intestinal infection has also been described (Lee et al., 2014).

## Difference between risk factors and affected populations in basidiobolomycosis, conidiobolomycosis, and mucormycosis

### Basidiobolomycosis

**FIGURE 3:**
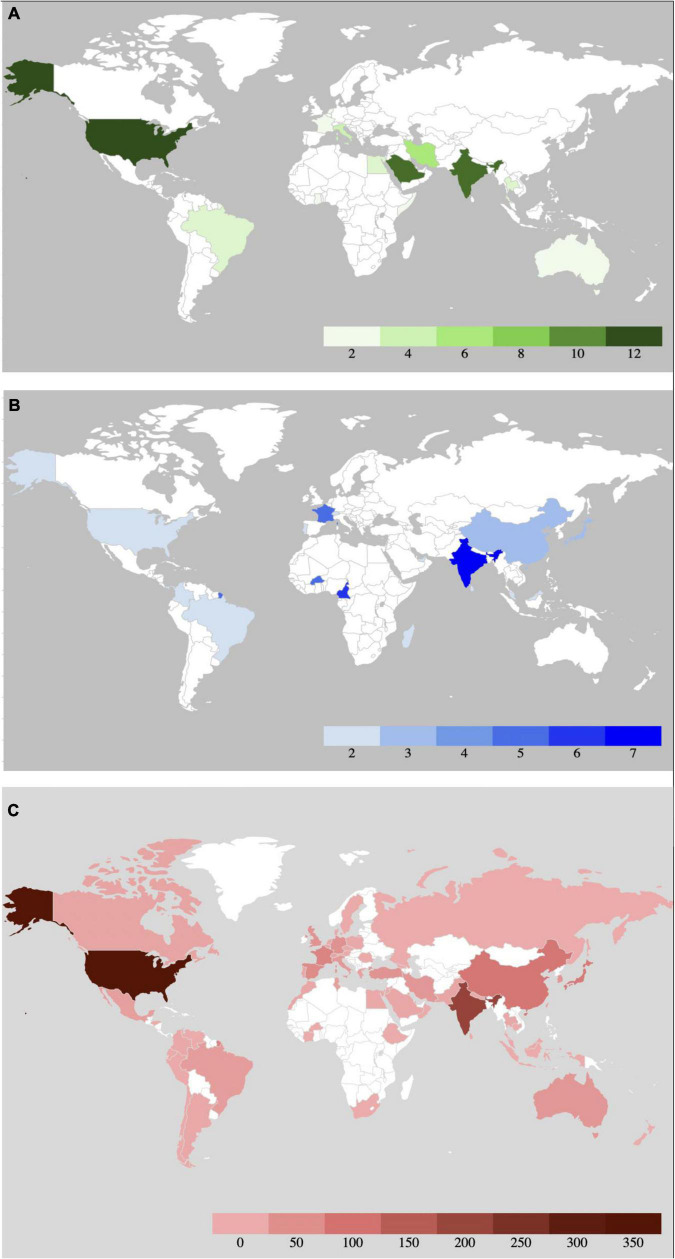
Global prevalence of infections by *Mucorales*, *Conidiobolus* spp. and *Basidiobolus* spp. based on a systematic review of case reports. **(A)** A systematic review of case reports with the term basidiobolomycosis and *Basidiobolus* from 1987 to 8/29/2022 was performed in PubMed. Cases with confirmed *Basidiobolus* spp. infections that allowed identification of the reporting country were included. Detailed information can be found in supplement A. **(B)** A systematic review of case reports with the term conidiobolomycosis and *Conidiobolus* spp. from 1978 to 8/29/2022 was performed in PubMed. Cases with confirmed *Conidiobolus* spp. infections that allowed identification of the reporting country were included. Detailed information can be found in supplement B. **(C)** A systematic review of case reports with the term mucormycosis between from 2000 to 8-22-2022 was performed in PubMed. Cases with confirmed *Mucorales* infections that allowed the identification of the reporting country were included. Detailed information can be found in supplement C. Systematic reviews were performed following the PRISMA 2020 criteria (Page et al., 2021).

Basidiobolomycosis is known for its burden in countries of the African continent (El-Shabrawi et al., 2017). In any case, the infection has been reported in several countries with tropical and subtropical climates (Figure 3A, Suppl. A). Pediatric patients are the most affected group (Gugnani, 1992). To a lesser extent, adolescents are also affected. Adults rarely contract the disease. In Uganda, the male-to-female ratio was reported 3:2, while a 3:1 ratio has been reported in Nigeria (Clark, 1968). The main risk factor is minor trauma related to insects or objects that carry the pathogen. The association between *Basidiobolus* spp. infection and comorbidity has not been described. On the other hand, a more invasive form of visceral basidiobolomycosis has been reported (Choonhakarn and Inthraburan, 2004).

### Conidiobolomycosis

Conidiobolomycosis is distributed worldwide, especially in tropical and subtropical countries (Figure 3B, Suppl. B). Nigeria is the country with the highest number of reported cases. From various studies, affected individual’s range in age from 20 to 50 years, but cases outside this age range have also been reported (Gupta and Soneja, 2019; Sigera et al., 2021). Studies show a different ratio between men and women. From a ratio of 4:1 in Gupta and Soneja to a ratio of 10:1 between men and women in Martinson and Clark (Martinson and Clark, 1967; Gupta and Soneja, 2019). Studies conducted in small populations with this disease show a lower male-to-female ratio (Chowdhary et al., 2010). It is particularly common in people who work or participate in outdoor activities (Martinson and Clark, 1967). However, disseminated infections with fatal outcome in a kidney transplant patient have been reported (Walker et al., 1992).

### Mucormycosis

In mucormycosis, the picture is different. Patients often present comorbidities related in some way to immune deficit. Major comorbidities include diabetes mellitus (diabetic ketoacidosis), hematologic malignancies, Coronavirus disease 2019 (COVID-19), solid organ transplantation, hematopoietic stem cell transplantation, liver disease, corticosteroid use, and neutropenia. Of course, this infection can also occur in patients without concomitant diseases associated with severe trauma such as a car accident, surgery, etc. However, it may also be associated with minor trauma such as injection sites (Song et al., 2017; Patel et al., 2020; Hoenigl et al., 2022). The *Mucorales* infection has been reported from several countries, with a significant burden in United States (Suppl. C) and India (Prakash and Chakrabarti, 2021). The global distribution of mucormycosis based on case reports is shown in Figure 3C.

The infection can occur in any age group. The multicenter observational study in India by Patel et al. (2020) showed an age range of 35–58 years. Meanwhile, a systematic review and meta-analysis of case reports found a range of 39–61 years (Jeong et al., 2019). It is important to note that the pediatric population may also be affected. For example, intestinal infections have been reported in neonates (Sarin, 2010). In addition, cerebral rhino-orbital form has been described in children with comorbidities (Amanati et al., 2021; Masmoudi et al., 2021). The male-to-female ratio is about 2.2:1, with slight variations between reports (Song et al.; Jeong et al., 2019; Patel et al., 2020; Gupta and Ahuja, 2021).

## Virulence and invasion factors of basidiobolomycosis, conidiobolomycosis, and mucormycosis

### Basiobolomycosis

Virulence factors are summarizing all properties that microorganisms use to establish infection, multiply, and evade host immunity (Hogan et al., 1996; Cross, 2008). Several of these virulence factors have been described in fungi pathogenic to humans (Nosanchuk and Martinez, 2015). However, in the case of basidiobolomycosis and conidiobolomycosis, information is still scarce. Elastase, esterase, collagenase, and lipase were suspected as virulence factors in *Basidiobolus* spp. (Shaikh et al., 2016). On the other hand, the production of urease, *N-*acetyl-β-glucosaminidase, trypsin, lipase, lecithinase, gelatinase, collagenase, and elastase were confirmed as virulence factors in 10 isolates of *Basidiobolus ranarum* (Feio et al., 1999). Additionally, extracellular enzyme activities of proteases and lipases were evaluated *in vitro* whilst amylase, RNAse, and DNAse activities were missing in *Basidiobolus* (Okafor et al., 1987). Even though the *in vitro* production of extracellular enzymes may be crucial for the development of basidiobolomycosis, infection models are essential to confirm the activity of these enzymes as virulence factors.

### Conidiobolomycosis

*Conidiobolus coronatus* was proven for the *in vitro* presence of extracellular lipases and proteases (Okafor et al., 1987) and for the production of collagenase, esterase, and elastase from pathogenic and saprophytic strains of *Conidiobolus coronatus* (Fromentin, 1978; Gugnani, 1992). In addition, the production of hydrolytic enzymes, including proteases and chitinases, has been demonstrated in insect cuticle infection models (Freimoser et al., 2003). *C. coronatus* also expressed a sequence tag (EST) (BQ622285) with similarity (*E* = 4 × 10^–32^) to a ferritin subunit (iron storage protein) (Freimoser et al., 2003). Components of iron uptake and iron storage are virulence factors that has been studied in other fungal pathogens (Haas, 2012; Fourie et al., 2018; Martínez-Pastor and Puig, 2020).

In *Galleria mellonella* infected by *C. coronatus*, the autophagic pathway is induced under the influence of stress and reactive oxygen species (Kazek et al., 2020). In another study, *C. coronatu*s was shown to kill larvae of *G. mellonella* by damaging hemocytes through the protein coronatin-2 (Boguś et al., 2017). In the case of conidiobolomycosis, most infection models have been performed on insects. It is likely that these virulence factors are also expressed in humans and contribute to pathogenesis. Invasion and destruction of infected tissue may be enhanced by enzyme production of *C. coronatus*.

### Mucormycosis

In the case of mucormycosis, the picture is entirely different from the previous fungal diseases described. Several studies have been performed describing virulence factors in different species within the order *Mucorales* (Hassan and Voigt, 2019). Epithelial cells are critical in patients infected with mucormycosis. In infection models using mice, glucose-regulated protein 78 (GRP78) expression was shown to increase in the presence of elevated glucose and iron concentrations (Liu et al., 2010). Indeed, diabetic mice showed increased expression of GRP78 in epithelial cells of the paranasal sinuses, lungs, and brain. Liu et al. (2010) showed that *Rhizopus arrhizus* (formerly: *Rhizopus oryzae*) enhanced epithelial cell invasion and damage in a receptor-dependent manner. Later, Gebremariam et al. (2014) described that spore coat protein homologs (CotH) from *Mucorales* is the fungal ligand for GRP78. Reduction of CotH expression in a mutant strain of *R. arrhizus* demonstrated decreased virulence in diabetic mouse models of infection (Gebremariam et al., 2014).

Other host factors associated with mucoralean invasion include contact with the extracellular matrix proteins and platelet-derived growth factor receptor (PDGFR). Bouchara et al. (1996) showed that *R. arrhizus* spores adhere to laminin and collagen type IV. This is important in patients with diabetes, chemotherapy and other diseases that can cause epithelial damage. As a result of epithelial damage, extracellular matrix proteins are exposed that can serve as fungi ligands (Bouchara et al., 1996). PDGFs, on the other hand, are a family of receptors found on various cell types. They have been found to affect blood vessel formation and early hematopoiesis (Andrae et al., 2008). A variety of gliomas, sarcomas, and leukemias are associated with autocrine PDGF activation (Andrae et al., 2008; Demoulin and Montano-Almendras, 2012). Epithelial damage has been linked to PDGFs in mucormycosis. When PDGFs were inhibited, endothelial damage was decreased in a model of infection with *R. delemar* (Chibucos et al., 2016). In addition, the results of Chibucos et al. (2016) suggest that *Rhizopus* may induce angiogenesis pathways to support its hematogenous dissemination. Also a massive platelet activation was reported in mice infected with *Lichtheimia corymbifera* leading to angioinvasion, thrombosis and necrosis followed by dissemination (Schulze et al., 2017).

After penetrating the tissue. It is possible that these dormant spores of *Mucorales* germinate and swell in the tissue, which in turn produces rapidly growing hyphae as part of their natural life cycle. According to various studies, filamentous growth, and germination lead to angioinvasion, vascular thrombosis, and tissue necrosis in the host (Warkentien et al., 2012). For survival in infected tissues, *Mucorales* require the uptake of iron. *Mucorales* can take up iron *via* three pathways: high-affinity iron permeases, low molecular weight iron chelators (siderophores such as rhizoferrin and xenosiderophores such as deferoxamine), and extraction of iron from host hemoglobin *via* heme oxygenase (Thieken and Winkelmann, 1992; Stanford and Voigt, 2020). *R. arrhizus* showed a significant increase in growth in serum samples with exogenous increase in iron concentration (Artis et al., 1982). Another example of the contribution of iron in the infection process is the detection of mucormycosis in dialysis patients after receiving therapy with the xenosiderophore deferoxamine (Boelaert et al., 1993). Moreover, the application of the xenosiderophore deferasirox resulted in an increased lethality in mice (Spellberg et al., 2012). So, these siderophores need to be carefully and conservatively administered in patients with risk of mucormycosis.

Proteases produced by *Mucorales* contribute to shocking tissue damages by expansive necroses. *Rhizopus* alkaline protease enzyme (Arp) has been detected in *R. microsporus* var. *rhizopodiformis*, which plays a role in promoting the coagulation process in patients with mucormycosis (Spreer et al., 2006). Serine and aspartate proteases (SAPs) produce proteolytic activities aid in the colonization and penetration of tissues (Schwartze et al., 2014; Mandujano-González et al., 2016). *In vitro* studies show the presence of polygalacturonases, amylases, proteases, lipases, and laccases but their role as virulence factors was not confirmed by animal models yet (Alves et al., 2002; Geethanjali et al., 2020). Although, the proteases may obviously contribute to mucormycosis-induced tissue damage.

The susceptibility of *Mucorales* to antifungal agents must be considered because fluconazole (FLU) is used as first-line choice drug in patients with suspected fungal disease. But these mucoralean fungi are not susceptible to short-chain azoles such as FLU (Lee et al., 2013; Caramalho et al., 2017). The resistance to FLU in *M. circinelloides* has been linked to the RNA interference (RNAi) pathway (Calo et al., 2014). RNAi uses double-stranded RNA molecules to silence genes through translation or transcription and was previously known as contributor to co-suppression, post-translational gene silencing (PTGS), or silencing. Epimutant strains of *M. circinelloides* produce sRNAs specific for the drug target gene and transiently suppress its expression by mRNA degradation. After several passages in a drug-free medium, the epimutants re-express the target gene and become sensitive to the antifungal drug again (Calo et al., 2014).

Phagocytic cells play a critical role in the clearance of pathogens. The macrophage-mediated response has been highlighted in various fungal pathogens such as *Cryptococcus neoformans*, *Candida albicans*, and others (Xu and Shinohara, 2017). The intracellular persistence of *R. arrhizus* spores in alveolar macrophages was widely demonstrated (Ghuman and Voelz, 2017; Andrianaki et al., 2018; López-Fernández et al., 2018). This intracellular survival is achieved by inhibition of phagolysosome formation by melanin on the spore surface in *R. arrhizus* (Andrianaki et al., 2018). Melanin is a pigment found in many organisms. This melanin has already been linked to the evasion of immunity by various mechanisms in fungi such as *Aspergillus fumigatus and C. neoformans* (Casadevall et al., 2000). In the case of *R. arrhizus* has a different structure from the 1,8-dihydroxynaphthalene (DHN) melanin found in *A. fumigatus* as it is eumelanin (Soliman et al., 2020). It inhibits LC3-associated phagocytosis (Andrianaki et al., 2018). On the other hand, through transcriptomics of the host and pathogen, studies on iron supplementation and genetic manipulation of the iron assimilation pathway of fungi. Iron restriction in macrophages was shown to regulate immunity to *Rhizopus* (Andrianaki et al., 2018).

Mucoricin is a cell-associated/secreted/shed is a ricin-like protein which is a carbohydrate binding lectin and highly potent toxin widely present in pathogenic mucoralean fungi (Soliman et al., 2021). The toxin has been associated with inhibition of protein synthesis and cell-destructive effects (apoptosis and necrosis), promotes inflammation and recruitment of polymorphonuclear cells, increases vascular permeability, and protects hyphae from phagocytosis in necrotic tissue. Treatment with IgG anti-mucoricin in models of intrathecal infection with *R. delemar* in mice decreased mortality compared to the control group (Soliman et al., 2021). In addition, in mice with pulmonary mucormycosis, the use of IgG anti-mucoricin protected the tissue from inflammation and infiltration with *R. delemar* (Soliman et al., 2021). A summary of virulence factors is shown in Table 1.

**TABLE 1:** Virulence factors associated with basidiobolomycosis, conidiobolomycosis, and mucormycosis.

A) Virulence in basidiobolomycosis		
Name	Mechanism	References
Elastase	Enzyme from the class of proteases (peptidases), which degrade proteins. Probably associated with tissue invasion.	Shaikh et al., 2016
Esterase	Enzyme breaks down esters into an acid and an alcohol during a process known as hydrolysis. Probably associated with tissue invasion.	Shaikh et al., 2016
Collagenase	A group of proteolytic enzymes that break down collagen and gelatin. Probably associated with tissue invasion.	Shaikh et al., 2016
Lipase	Enzymes that catalyzes the hydrolysis of fats. Probably associated with tissue invasion.	Shaikh et al., 2016

**B) Virulence in conidiobolomycosis**		
**Name**	**Mechanism**	**References**

Lipase	Enzymes that catalyzes the hydrolysis of fats. Probably associated with tissue invasion.	Shaikh et al., 2016
Proteasease	Protein degrading enzymes. Probably associated with tissue invasion.	Shaikh et al., 2016
EST (BQ622285)	Iron storage. Ferritin-like protein.	Freimoser et al., 2003
Coronatin-2	Coronatin-2 kills larvae of G. mellonella by damaging hemocytes	Boguś et al., 2017
Reactive Oxygen Species	The autophagic pathway is activated in Galleria mellonella that has C. coronatus infection as a result of stress and reactive oxygen species.	Kazek et al., 2020

**C) Proven virulence in mucormycosis**		
**Name**	**Mechanism**	**References**

High-affinity iron permease	Iron uptake and transport in *Mucorales* fungi especially during the lack of iron in the environment	Stanford et al., 2021
Spore coat protein	Invasins in the pathogenesis of mucormycosis	Gebremariam et al., 2014
Alkaline *Rhizopus* protease enzyme	Enhancing the coagulation process in patients suffering from mucormycosis	Spreer et al., 2006
ADP-ribosylation factor	Fungal dimorphism and virulence in *M. circinelloides* (Patiño-Medina et al., 2018)	
Dihydrolipoyl dehydrogenase	Most abundant antigen in the serum of patients suffered from FLD compared to healthy donors	Rognon et al., 2016
Calcineurin	Tangible role in the transition from yeast to hyphae in *M. circinelloides*	Lee et al., 2013
Serine and aspartate proteases	Most common candidates of the secreted proteases in the genome of *L. corymbifera*	Schwartze et al., 2014
	In *Rhizopus* act as secrete lytic enzymes	
Heme oxygenase	Obtain iron from host hemoglobin and might explain the angioinvasive nature	Stanford and Voigt, 2020
Rhizoferrin	Obtain iron through a receptor-mediated, energy-dependent process	Thieken and Winkelmann, 1992
RNAi-mediated resistance to fluconazole	*M. circineloides* hides the fluconazole target by RNAi	Calo et al., 2014
Intracellular macrophage survival	Inhibition of phagolysosome formation by melanin on the spore surface of *R. arrhizus.*	Andrianaki et al., 2018
Mucoricin	The mucoricin of *R. delemar* causes cell damage, promotes inflammation and recruitment of polymorphonuclear cells, increases vascular permeability, and protects hyphae from phagocytosis in necrotic tissue.	Soliman et al., 2021
Rhizoxin	An antimitotic macrocyclic polyketide metabolite.	Ibrahim et al., 2012
RNA interference pathways	A way of negatively regulating gene expression through small non-coding RNAs or short RNAs (sRNAs)	Calo et al., 2014

Description of virulence factors and mechanisms in: (A) basidiobolomycosis, (B) conidiobolomycosis, (C) proven virulence factors in mucormycosis, and (D) unproven virulence factors in mucormycosis. In the right column the references are indicated to the bibliographical source.

## Clinical presentation and diagnosis of basidiobolomycosis, conidiobolomycosis, and mucormycosis

### Basidiobolomycosis

Clinically, the patient may present with cutaneous, subcutaneous, intestinal, and/or disseminated infection. Initial clinical symptoms are usually non-specific, making it difficult for patients to seek medical attention (de Leon-Bojorge et al., 1988). The cutaneous or subcutaneous form is common in male pediatric patients (Shaikh et al., 2016). The lower limbs and gluteal region are frequently affected. However, several anatomic sites of infection have been reported (de Leon-Bojorge et al., 1988; Thotan et al., 2009; al Jarie et al., 2011; Kumar Verma et al., 2012; Mendoza et al., 2015). The cutaneous lesions are painless and have a mass appearance with skin color changes. They are well demarcated, have smooth margins, and no lymphatic involvement (de Leon-Bojorge et al., 1988). This infection is usually slow and progressive. Involvement of bone and muscle is unlikely. This clinical picture may resemble other pathologies such as lupus vulgaris, soft tissue tumors, Burkitt’s lymphoma, synovial sarcoma, and fibrosing panniculitis. Infectious differential diagnosis may consider *Actinomyces* spp. or *Nocardia* spp., cutaneous pythiosis, chromoblastomycosis, cutaneous atypical Mycobacterium species, primary subcutaneous mucormycosis, sporotrichosis, etc., (de Leon-Bojorge et al., 1988; al Jarie et al., 2011).

Food contamination with *Basidiobolus* spp. may lead to gastrointestinal infections (Khan et al., 2001). Clinical symptoms include abdominal pain, intermittent low-grade fever, vomiting, bloody mucosal diarrhea, intestinal bleeding, gastric or intestinal ulceration, abdominal distension, obstructive symptoms, etc., (Costa, 2012; Mendoza et al., 2015; Elzein et al., 2018; Omar Takrouni et al., 2019). In the context of non-specific symptomatology, the differential diagnosis can be complex. Takrouni et al. described the case of an 18-year-old patient with an obstructing cecal mass in whom a malignant tumor was initially suspected. Because of the obstructive symptoms, surgical resolution of the case was required. During laparotomy, tissue samples were obtained from the intestine. The biopsies showed fungal hyphae that had invaded the intestinal wall and exhibited Splendore-Hoeppli phenomenon (Omar Takrouni et al., 2019). This case shows us the importance of laboratory techniques support in any form of disease. Indeed, the diagnosis in these patients can be confirmed by culture, histopathology, and detection of the microorganism by polymerase chain reaction (PCR).

### Conidiobolomycosis

Infections with *C. coronatus* are common in male adults without a history of immunosuppression (Martinson and Clark, 1967). They usually involve the subcutaneous tissues of the nasal and facial regions (Martinson and Clark, 1967; Clark, 1968; Fischer et al., 2008; Isa-Isa et al., 2012). This trophism has led to the term rhinoconidiobolomycosis (Clark, 1968). In any case, it is important to note that *Basidiobolus* spp. can also infect the face (Goyal et al., 2010). Nasal nodules grow slowly during the infection. Initially, nasal obstruction occurs, followed by erythematous infiltration and thickening of the nasal skin. Eventually, the facial structures begin to deform and show a marked increase in volume (Clark, 1968; Fischer et al., 2008; Goyal et al., 2010; Isa-Isa et al., 2012). Chronic infection (in some cases several months to years), if untreated, leads to indolent facial deformities. Based on these findings, a classification was proposed using early, intermediate and late progression of the disease (Blumentrath et al., 2015); (1) early: the patient has been infected for less than 11 months and has no nodules in the nostril or invasion of the soft tissues of the face, (2) intermediate: the patient has been infected between 11 and 12 months and has a nodule in the nostril or invasion of the facial soft tissues, (3) late: patients with a development time of more than 12 months, (4) atypical: severe pain, necrosis, orbital involvement, or systemic spread occurs within the first 12 months of disease).

Invasive and disseminated disease in patients with conidiobolomycosis is rare but possible. Isolation of *Conidiobolus lamprauges* in a 61-year-old patient with recurrent mantle cell lymphoma and postmortem diagnosis revealed growth of *C. lamprauges* in the tracheal mucosa, lungs, kidneys, and spleen (Kimura et al., 2011). Moreover, invasive lung disease with *C. pachyzygosporus* in a 71-year-old patient with myeloid leukemia and histopathologic diagnosis of lung biopsy showed large and tortuous hyphal elements with occasional septa (Stavropoulou et al., 2020). Therefore, 18S rDNA pan-fungal polymerase chain reaction (PCR) was positive for a *Conidiobolus* spp. at 1,582 copies/ml. Finally, PCR was performed for the 28S ribosomal DNA (rDNA), which showed good discrimination for 100% identity with *C. pachyzygosporus* (Stavropoulou et al., 2020). This demonstrates that invasive and disseminated forms of conidiobolomycosis can be included in the differential diagnosis in patients with severe immunosuppression.

### Mucormycosis

*Mucorales* infection can cause rhino-orbital-cerebral, pulmonary, cutaneous, gastrointestinal, disseminated, and uncommon forms of mucormycosis (Cornely et al., 2019). A systematic review and meta-analysis of case reports revealed rhino-orbital-cerebral mucormycosis as the most common form and affected 34% of the population (Jeong et al., 2019). Cutaneous mucormycosis accounted for 22% and pulmonary mucormycosis occurred in 20% of the cases studied. After initial infection (rhino-orbital, pulmonary, cutaneous) spread is possible in 22% of cases. Unfortunately, patients with disseminated mucormycosis have a mortality of up to 94%. Although gastrointestinal mucormycosis occurred in 8% of case reports, mortality can be as high as 85%. Pulmonary mucormycosis ranks third in mortality with 76% of patients analyzed in case reports. It is interesting to note that in the pediatric population there is a greater association between the cutaneous and gastrointestinal forms of mucormycosis (Jeong et al., 2019).

A special section deserves COVID -19 associated mucormycosis (CAM) highlighted since early 2021. This superinfection has been reported in at least 18 countries. The country with the most reports was India, followed by the United States of America (Hoenigl et al., 2022). Most affected were severely ill patients with COVID -19, diabetics, and patients taking corticosteroids (Hoenigl et al., 2022). CAM Patients were particularly susceptible to a combination of risk factors. These included an elevated ferritin, islet cell damage, cytokine storm (interleukin 6), endothelitis, ketoacidosis, use of antifungal drugs, uncontrolled corticosteroid, etc., (Gupta and Ahuja, 2021; Mathew et al., 2021; Pal et al., 2021; Hoenigl et al., 2022). In the various studies, rhino-orbital-cerebral and pulmonary mucormycosis is shown as the main presentation (Cornely et al., 2019; Gupta and Ahuja, 2021).

Rhino-orbital-cerebral mucormycosis is caused by nasal invasion of *Mucorales* sporangiospore in susceptible patients. Once the infection spreads, surrounding tissues also become infected. Depending on growth, the fungi may invade the palate from below, infect the upper part of the sphenoid sinus, invade the cavernous sinus laterally, and infect the orbits. Fungi invade the skull through the orbital apex or cribriform plate of the ethmoid bone or by hematogenous dissemination. Invasion of cerebral vessels with or without mycotic aneurysms is possible. Initially, the patient reports fever and pain in the midface or retroocular region (Hosseini and Borghei, 2005; Orguc et al., 2005). Nasal congestion, rhinorrhea, nasal hypoesthesia, and epistaxis may also occur. If the eye is affected, diplopia, blurred vision, proptosis, conjunctival chemosis, nygtasms, and amaurosis may occur (Ribes et al., 2000). If the brain is affected, the patient may show dizziness, gait disturbances, altered mental status, and even seizures. Physical examination reveals nasal and orbital cellulitis. This rapidly progresses to necrosis of the affected areas in association with fungal angioinvasion (Ribes et al., 2000; Ganesan and Sivanandam, 2022).

When *Mucorales* spores reach the lower respiratory tract. They can cause endotracheal and/or bronchoalveolar infections in patients with risk factors. Endotracheal infection can lead to airway and lung collapse (Collins et al., 1999). Invasion into the hilar region with local spread to the mediastinum, pericardium, and thoracic cavity has also been described (Connor et al., 1979; Seifert et al., 2020). Patients may initially present with fever and non-specific chest pain. These manifestations may be confused with other etiologies. For example, bacterial infections, pulmonary candidiasis, or aspergillosis (Connor et al., 1979; Passamonte and Dix, 1985). Qu et al. (2021) analyzed 59 patients with proven pulmonary mucormycosis in western China. The three most common clinical manifestations were cough (93%), fever (52%), hemoptysis (45%), dyspnea (30%), and chest pain (15%). Because of clinical non-specificity, imaging studies are useful in these patients. In the early stages, a peribronchial opacity may be observed. In advanced stages, the disease progresses to consolidations, nodules, or masses. The reverse halo sign is associated with pulmonary mucormycosis rather than invasive pulmonary aspergillosis (Agrawal et al., 2020).

*Mucorales* can infect the skin directly by trauma. It has been associated with inoculation due to minor trauma in patients with comorbidities such as leukemia (Maleitzke et al., 2019). Cases have also been observed in severe trauma due to motor vehicle accidents (Tyll et al., 2016). Or associated with biomedical devices (Roden et al., 2005; Rammaert et al., 2012; Perz et al., 2020). Secondarily and rarely, the skin may be affected by the spread of pulmonary or gastrointestinal foci (Iyengar et al., 2017). Cutaneous mucormycosis can be classified as superficial and deep. It is superficial when it affects the skin or subcutaneous cellular tissue. On the other hand, if tendons, muscles and/or bones are affected, it is classified as deep (Roden et al., 2005; Spellberg et al., 2005). Initially, cutaneous mucormycosis usually has a cellulitis-like appearance. As it progresses, there is blackish skin discoloration, scabs, abscesses, ulcers, small nodules, indurated masses, etc., (Johnson et al., 1987; Eaton et al., 1994; Baradkar and Kumar, 2009; García-Pajares et al., 2012; Hsieh et al., 2013; Perz et al., 2020). This leads to an inflammatory periphery, a central black eschar surrounded by necrotic tissue (Baradkar and Kumar, 2009; García-Pajares et al., 2012; Rammaert et al., 2012; Perz et al., 2020). Although the bibliography highlights the black eschar. The analysis of 623 patients with cutaneous mucormycosis found that only 55% of the cases were associated with the term’s “necrosis,” “necrotic,” or “necrotizing” (Skiada et al., 2022). An individualized, holistic analysis of the patient’s risk factors, potential mechanisms of infection, and clinic is essential.

In gastrointestinal mucormycosis, the stomach, colon, and ileum are most affected (Michalak et al., 1980; Spellberg, 2012). In addition, patients may be mistaken for cancer due to masses in the cecum, ileum, or appendix (Busbait et al., 2020). The most important risk factors are corticosteroid use and diabetes (Michalak et al., 1980; Spellberg, 2012). In a systematic review of 80 cases with gastrointestinal mucormycosis, abdominal pain occurred in 50%, fever in 37%, and perforation in 27% (Skiada et al., 2022). This study also included neonates with abdominal pain and vomiting (40%) and gastrointestinal perforation (30%). In addition, fever, obstruction, and abdominal distension occurred in 20% of the neonates studied (Skiada et al., 2022). Imaging findings may contribute to the diagnosis. Computed tomography findings can range from gastric pneumatosis and reduced gastric wall enhancement to mass like thickening (Ghuman et al., 2021). Because of the wide range of differential diagnoses, culture, histopathology, and PCR of biopsies of the infected tissue are required for confirmation in all cases (Cornely et al., 2019). Over time, the disease may spread to many organs. After angioinvasion, *Mucorales* can migrate to any organ. This leads to rare forms such as endocarditis, osteomyelitis, peritonitis, and pyelonephritis (Echols et al., 1979; Virmani et al., 1982; Aymoré et al., 2005; Ram et al., 2007).

Within these disseminated forms, renal mucormycosis attracts our special attention. This mucoralean infection of the kidney is rare, usually unilateral, and potentially fatal. It has been observed in both immunocompromised and immunocompetent patients. Clinically, patients present with the triad of fever, low back pain, and sepsis. Assessment of general condition is important as it is associated with acute kidney injurie. Imaging studies are important as they reveal areas of lower density, focal vascular loss, and infarction. Urine microscopy and culture can be used to diagnose renal mucormycosis (Gupta and Gupta, 2012; Kuy et al., 2013; Bhadauria et al., 2018; Devana et al., 2019).

### Diagnosis

Diagnosis of these fungal infections should include analysis of risk and demographic factors, clinical presentation, site of infection, clinical evolution, and imaging studies. If suspicion exists, it must be confirmed by examination of biopsy fragments from infected tissue for fungal cultures, direct visualization with potassium hydroxide (KOH), histopathology, and detection of microorganisms by polymerase chain reaction (PCR).

### Direct observation from tissue and fungal cultures

It is commonly recommended to investigate several biopsy fragments of the affected tissue by microbiological and histopathological analyses. Specimens must be transported in a sterile container with a closed lid to avoid contamination by microorganisms from the environment. They should not be transported under refrigeration as this inhibits the growth of *B. ranarum* (Davis et al., 1994). Potassium hydroxide is a strong base. When it encounters tissues, it softens, digests, and cleans the tissues. Fungi are not attacked by KOH because of its composition. Therefore, it digests the cells of the tissue under study and allows observation of fungal structures (Cheesbrough, 2005; Tille, 2021). Small 2 mm^2^ blocks of biopsied tissue should be placed into a glass slide with 1 drop of 10% KOH (10 g KOH, 10 ml glycerol, 80 ml distilled water) (Cheesbrough, 2005; Tille, 2021). Which will allow shows under light microscopy broad ribbon like hyaline pauciseptate or aseptate hyphae (Cheesbrough, 2005; Tille, 2021). The importance of KOH smears in diagnosis cannot be overstated. Using a fluorescent microscope to examine Blankophor (fluorescent dye) wet-mount preparation of tissue biopsy increases diagnostic sensitivity (Kumar Verma et al., 2012).

Among the disadvantages of wet preparation with KOH is the large and variable sensitivity in detecting fungal pathogens (Afshar et al., 2018). Biopsies may have low or no microbial load at the site of collection. The site from which the biopsy was taken is not representative site of infection (Cheesbrough, 2005; Tille, 2021). It is important that personnel trained in mycology process and analyze the specimen. The detection of pauciseptate or aseptate hyphae confirms the etiology of the fungi. However, they do not allow differentiation between basidiobolomycosis, conidiobolomycosis, and mucormycosis because the findings are similar (Tille, 2021). For this reason, culture or molecular diagnosis is important for specific identification of the microorganism. Although 2% Sabouraud dextrose agar (SDA) is generally recommended as a culture medium for fungi. Some fungal pathogens may have difficulty growing. Therefore, potato dextrose agar (PDA) and even brain heart infusion agar (BHI) are much more enriched and facilitate the growth of microorganisms (Procop et al., 2016; Tille, 2021).

If the biopsy is from a tissue with a large saprophytic bacterial microbiota will be processed, the use of SDA or BHI supplemented with chloramphenicol is very helpful (Procop et al., 2016; Tille, 2021). The incubation of the cultures with the samples should be carried out at 37°C and at room temperature. *Basidiobolus ranarum* grows with a creamy rugose colony of on 2% Sabouraud dextrose agar. When genetic material is exchanged, zygospores develop with their characteristic beaks as a result. Primary culture in SDA shows a yellow-white colony of *Conidiobolus coronatus* after 48 h. Some aerial hyphae and satellite colonies can be seen on the lid of the SDA plate due to the ejection of conidia from the sporangiophores (Vilela and Mendoza, 2018). The name coronatus comes from the development into fully formed corona secondary conidia (Vilela and Mendoza, 2018). In just 5 days, *Rhizopus* and *Mucor* can growth cottony-like appearance in a Petri dish. As the sporangiospores mature within the sporangium, the growth usually turns grayish-brown with aging. The reverse remains a pale white. *Rhizopus* and *Mucor* are fast-growing fungi that can fill a Petri dish with fluffy, cotton candy-like growth in less than 5 days. The growth in PDA is usually whitish and may turn grayish brown with age due to maturation of sporangiospores in the sporangium. The reverse side remains pale white (Lass-Flör, 2009). Under the microscope, *Mucor* sp. has no apophysis, a spherical sporangium, and no rhizoids. *Rhizopus* sp., on the other hand, has not prominent apophysis, a spherical sporangium, and abundant rhizoids (often well developed) (Ribes et al., 2000; Lass-Flör, 2009).

### Histopathological findings

Histopathologic examination may provide clues to distinguish mucormycosis from basidiobolomycosis or conidiobolomycosis. If fungal disease is suspected, it is recommended that a Grocott (methenamine) silver stain (GMS) or Periodic acid-Schiff stain (PAS) be ordered. The morphology of the tissue around the fungus can be better identified with PAS. Meanwhile, the fungal structures are highlighted by GMS (Denning et al., 2003; Procop et al., 2016; Tille, 2021). A commonly used stain is hematoxylin and eosin (H&E). In conidiobolomycosis and basidiobolomycosis, H&E-stained tissues that are infected show extensive fibrosis and an inflammatory reaction (Denning et al., 2003; Procop et al., 2016; Tille, 2021). This inflammatory reaction is usually characterized by demonstrated as eosinophilic cuff surrounding the coenocytic hyphae that may include eosinophils (Splendore–Hoeppli phenomenon) (Denning et al., 2003; Gopinath, 2018). Although patients with entomophthoromycosis generally do not have angioinvasion (Denning et al., 2003). Rare cases of gastrointestinal conidiobolomycosis with angioinvasion have been reported (Elzein et al., 2018).

A *Mucorales* hyphae varies in width (6–25 μm), and it is usually non-septate or sparsely septate (coenocytic hyphae). In mucormycosis, angioinvasion by coenocytic hyphae occurs with subsequent necrosis or hemorrhage of the surrounding tissue. When inflammation is present, it is often purulent, less commonly granulomatous, and neutrophils may be observed. In the study by Ben-Ami et al. (2009) in cancer patients with pulmonary mucormycosis, it is described that inflammation is limited but there is extensive angioinvasion into the wall or lumen of blood vessels. Interestingly, *Mucorales* hyphae stain poorly in GMS. For this reason, mucormycosis should be suspected if staining of hyphae with GMS is poor (Denning et al., 2003).

### Molecular diagnosis

Detection of sequences of genetic material of pathogens can reduce the waiting time for results. Hata et al. reported the development of a real-time PCR assay for the detection of *Lichtheimia* (formerly *Absidia*), *Apophysomyces*, *Cunninghamella*, *Mucor*, *Rhizopus*, and *Saksenaea* in culture and tissue samples (Hata et al., 2008). The analytical sensitivity of this real-time PCR for the detection of *Mucorales* was 10 targets/μl and showed no cross-reactivity for bacteria and fungi (Hata et al., 2008). For samples from cultures, clinical sensitivity was 100% (39/39) and specificity was 92% (59/64). Meanwhile, for biopsies, unfortunately, only 2 samples were processed with a sensitivity and specificity of 100%. It is clearly advisable to increase the number of biopsies from patients with confirmed mucormycosis in order to calculate sensitivity and specificity correctly (Hata et al., 2008).

Another method used in patients with mucormycosis is the detection of circulating DNA in blood and cerebrospinal fluid (Shigemura et al., 2014; Millon et al., 2016; Hiramoto et al., 2020). In these studies, sequences of oligonucleotide primers and markers for detecting circulating DNA of *Mucor/Rhizopus*, *Lichtheimia*, or *Rhizomucor* with detection limit of 1.0 × 10^3^ copies/ml (Shigemura et al., 2014; Hiramoto et al., 2020). The advantage is that the detection of circulating DNA from the first day of infection (Hiramoto et al., 2020). In any case, more cases need to be studied and compared with other methods to determine the sensitivity and specificity of this technique. On the other hand, Alanio et al. evaluated the detection of *Mucorales* in 19 tissue samples by PCR/electrospray ionization mass spectrometry (PCR/ESI- MS) with a detection time of 6 h (Alanio et al., 2015). PCR/ESI-MS was more effective than other molecular methods in identifying *Mucorales* to species level. This methodology could not correctly identify samples with *Cunninghamella* spp. or *Saksenaea vasiformis*, PCR/ESI-MS databases need to be improved to improve the identification (Alanio et al., 2015).

Few studies have reported PCR primers for the identification of *Entomophthoromycotina* fungi in infected tissues in patients with conidiobolomycosis or basidiobolomycosis. Taxon-specific primer pairs were designed to amplify a 28S rDNA fragment for *Entomophthoromycotina* detection. For *B. haptosporus*/*ranarum* Ba1 (AAAATCTGTAAGGTTCAACCTTG) and Ba2 (TGCAGGAGAAGTACATCCGC) (Voigt et al., 1999). For the detection of *C. coronatus* was designed Cc1 (TCTCTTAACTTGCTTCTATGCC) and Cc2 (CTTTAATTAAGCTAATCAACATG) (Voigt et al., 1999). El-Shabrawi et al. detected *B. ranarum* after 6 months in paraffin-embedded tissue (FFPE). This result was consistent with morphological, physiological, and molecular evidence of basidiobolomycosis in a patient with a fungal mass (El-Shabrawi et al., 2014). While this is a promising result. Validation in other clinical cases is needed to determine sensitivity, and specificity in clinical samples.

The next-generation metagenomic sequencing (mNGS) technique analyses nucleic acids from a wide range of microorganisms at the same time. Studies and case reports have shown that this strategy can identify the causative pathogen when other strategies have failed. There are some examples in the scientific literature. Sitterle et al. detected *B. meristosporus* in a biopsy of a colonic mass (Sitterlé et al., 2017). Falces-Romero et al. reported colonization by *Conidiobolus* sp. in a respiratory specimen from a patient with chronic obstructive pulmonary disease (Falces-Romero et al., 2017). Sun et al. detected *Rhizopus microsporus* in skin and lung biopsies from a patient with clinical symptoms suggestive of disseminated mucormycosis (Sun et al., 2021). In any case, contamination can lead to false-positive results, and detailed analysis of the clinical case to validate the results by experts in clinical microbiology and infectious diseases is recommended.

## Conclusion

The class Zygomycetes has been rejected because of the phylogenetic differences of the microorganisms that compose it. Currently, the classes *Mucoromycota*, which includes the order *Mucorales*, and *Zoopagomycota*, which includes the orders *Entomophthorales* (*Conidiobolus* spp.) and *Basidiobolales* (*Basidiobolus* spp.), are used. The term zygomycosis should not be used because it can lead to confusion. Instead, infection with *Mucorales* is referred to as mucormycosis, *Basidiobolus* spp. as basidiobolomycosis, and infection with *Conidiobolus* spp. as conidiobolomycosis. On the other hand, entomophthoromycosis is a broad term that includes infection with *Conidiobolus* spp. or *Basidiobolus* spp. Mucormycosis is an infection that occurs primarily in patients with impaired cellular immunity. In contrast, basidiobolomycosis and conidiobolomycosis usually occur in immunocompetent patients. However, the coenocytic hyphomycosis is proposed as a new term to summarize fungal infections caused by coenocytic (non-septate), terrestrial and basal lineage filamentous fungi which were formerly subscribed to the “zygomycetes” in a colloquial sense.

The clinical picture of patients infected with *Mucorales* shows a rapid evolution with angioinvasion and necrosis, ranging from the rhino-orbital-cerebral form to the disseminated form. Clinically, patients with *Basidiobolus* spp. show a slow course lasting many months with mass-like infection of the skin. *Conidiobolus* spp. is characterized by nasal infection with the appearance of a mass and a slow course over months that may become obstructive. The differential diagnosis based on epidemiologic, clinical, imaging, histopathologic, microbiologic, and molecular criteria must be critically analyzed in each patient. Although histopathology can be helpful in differentiating between mucormycosis and entomophthoromycosis based on angioinvasion, necrosis, and the Splendore-Hoeppli phenomenon. The confirmatory diagnosis is based on the report of a mycological culture and molecular biology tests. Confirmation by culture or molecular biology helps in the choice of antifungal agent based on the susceptibility profile of each microorganism.

In the context of the limitations of this study, it is important to note that the heat maps show prevalence based on case reports. This does not necessarily reflect the real global burden of conidiobolomycosis, basidiobolomycosis, and mucormycosis. For this reason, an international surveillance network with trained personnel and a global perspective is the solution we need and has not yet been adequately implemented.

## Author contributions

JA-E and KV drafted and revised the manuscript. Both authors contributed to the article and approved the submitted version.
