# Supplementary material for: An old confusion: Entomophthoromycosis versus mucormycosis and their main differences
Source: Front Microbiol. 2022 Nov 3;13:1035100. doi: 10.3389/fmicb.2022.1035100 (PMC9670544; doi:10.3389/fmicb.2022.1035100)
Supplement: Supplementary file 1 [file Data_Sheet_1.docx]

**Supplement A: basidiobolomycosis**

Methodology: Systematic reviews were performed following the PRISMA 2020 criteria (Page et al., 2021).

Search parameters: Pubmed database. term basibiobolomycosis and *Basidiobolus*.

(*Basidiobolus ranarum* synonyms: *Basidiobolus haptosporus, Basidiobolus haptosporus var. meristosporus, Basidiobolus haptosporus var. minor, Basidiobolus heterosporus, Basidiobolus meristosporus* – [Speciesfungorum](http://www.speciesfungorum.org/) Access 13 September 2022).

Filters: human, 1987-29.8.2022, case reports, access to the full article.

Inclusion criteria: *Basidiobolus* spp. confirmed infections, the case report allow identification of the reporting country.

Exclusion criteria: All cases that did not meet the inclusion criteria were excluded.

Research results: 65 results

Manuscripts that met the inclusion criteria: 54

Number of cases: 54

1. Aljehani SM, Zaidan TID, AlHarbi NO, Allahyani BH, Zouaoui BR, Alsaidalan RH, Aljohani SM. Pediatric intussusception due to basidiobolomycosis: a case report and literature review. BMC Pediatr. 2022 Jul 20;22(1):427. doi: 10.1186/s12887-022-03495-9. PMID: 35854289; PMCID: PMC9297541. Link <https://pubmed.ncbi.nlm.nih.gov/35854289/>
2. Abduh MS, Aldaqal SM, Almaghrabi J, Aljiffry MM, Elbadrawy HA, Alsahafi MA. A Very Rare Basidiobolomycosis Case Presented with Cecal Perforation and Concomitant Hepatic Involvement in an Elderly Male Patient: A Case Study. Int J Environ Res Public Health. 2022 Mar 14;19(6):3412. doi: 10.3390/ijerph19063412. PMID: 35329103; PMCID: PMC8951099. Link <https://pubmed.ncbi.nlm.nih.gov/35329103/>
3. Ravindranath A, Pai G, Sen Sarma M, Srivastava A, Lal R, Agrawal V, Marak R, Yachha SK, Poddar U, Prasad P. Gastrointestinal Basidiobolomycosis: A Mimic of Lymphoma. J Pediatr. 2021 Jan;228:306-307. doi: 10.1016/j.jpeds.2020.09.009. Epub 2020 Sep 8. PMID: 32916142. Link <https://pubmed.ncbi.nlm.nih.gov/32916142/>
4. Mousavi MR, Pouladfar G, Taherifard E, Badiee P, Anbardar MH. An Infant with Acute Bloody Diarrhea and Gastrointestinal Basidiobolomycosis: An Unusual Presentation of a Rare Disease. J Trop Pediatr. 2021 Jul 2;67(3):fmaa039. doi: 10.1093/tropej/fmaa039. PMID: 32734302. Link <https://pubmed.ncbi.nlm.nih.gov/32734302/>
5. Baradkar V, Chatterjee N, Shastri JS, Vedpathak MU. Ocular basidiobolomycosis - Rare presentation: A case report. Indian J Pathol Microbiol. 2020 Apr-Jun;63(2):270-272. doi: 10.4103/IJPM.IJPM_687_19. PMID: 32317530. Link <https://pubmed.ncbi.nlm.nih.gov/32317530/>
6. Alsharidah A, Mahli Y, Alshabyli N, Alsuhaibani M. Invasive Basidiobolomycosis Presenting as Retroperitoneal Fibrosis: A Case Report. Int J Environ Res Public Health. 2020 Jan 15;17(2):535. doi: 10.3390/ijerph17020535. PMID: 31952125; PMCID: PMC7014094. Link <https://pubmed.ncbi.nlm.nih.gov/31952125/>
7. Parisio EM, Camarlinghi G, Nardone M, De Carolis E, Mattei R, Sanguinetti M. Gastrointestinal basidiobolomycosis in a patient suffering from duodenal ulcer with perforation: First case report from Italy. New Microbiol. 2019 Apr;42(2):125-128. Epub 2019 Apr 17. PMID: 30994179. Link <https://pubmed.ncbi.nlm.nih.gov/30994179/>
8. Omar Takrouni A, Heitham Schammut M, Al-Otaibi M, Al-Mulla M, Privitera A. Disseminated intestinal basidiobolomycosis with mycotic aneurysm mimicking obstructing colon cancer. BMJ Case Rep. 2019 Jan 29;12(1):e225054. doi: 10.1136/bcr-2018-225054. PMID: 30700449; PMCID: PMC6352788. Link <https://pubmed.ncbi.nlm.nih.gov/30700449/>
9. Pezzani MD, Di Cristo V, Parravicini C, Sonzogni A, Tonello C, Franzetti M, Sollima S, Corbellino M, Galli M, Milazzo L, Antinori S. Gastrointestinal basidiobolomycosis: An emerging mycosis difficult to diagnose but curable. Case report and review of the literature. Travel Med Infect Dis. 2019 Sep-Oct;31:101378. doi: 10.1016/j.tmaid.2019.01.013. Epub 2019 Jan 17. PMID: 30660554. Link <https://pubmed.ncbi.nlm.nih.gov/30660554/>
10. Mohammadi R, Ansari Chaharsoghi M, Khorvash F, Kaleidari B, Sanei MH, Ahangarkani F, Abtahian Z, Meis JF, Badali H. An unusual case of gastrointestinal basidiobolomycosis mimicking colon cancer; literature and review. J Mycol Med. 2019 Apr;29(1):75-79. doi: 10.1016/j.mycmed.2018.11.004. Epub 2018 Dec 13. PMID: 30553627. Link <https://pubmed.ncbi.nlm.nih.gov/30553627/>
11. Gummadi GK, Pai BS, Nayak UKS, Prakash PY, Pai K. Basidiobolomycosis mistaken for cutaneous tuberculosis. Indian J Dermatol Venereol Leprol. 2019 Mar-Apr;85(2):236. doi: 10.4103/ijdvl.IJDVL_16_17. PMID: 29536980. Link <https://pubmed.ncbi.nlm.nih.gov/29536980/>
12. Sackey A, Ghartey N, Gyasi R. Subcutaneous basidiobolomycosis: A Case Report. Ghana Med J. 2017 Mar;51(1):43-46. doi: 10.4314/gmj.v51i1.9. PMID: 28959073; PMCID: PMC5611946. Link <https://pubmed.ncbi.nlm.nih.gov/28959073/>
13. Kothari PP, Ambulgekar RK, Kandhari VK, Bhatia DN. Clinical and radiological features of an unusual fungal infection of shoulder. ANZ J Surg. 2019 Mar;89(3):E87-E89. doi: 10.1111/ans.14024. Epub 2017 May 14. PMID: 28503783. Link <https://pubmed.ncbi.nlm.nih.gov/28503783/>
14. Rermluk N, Laolerd W, Chantharit P, Lertsithichai P, Wiratkapun C, Larbcharoensub N. Mammary subcutaneous basidiobolomycosis in a male. Breast J. 2017 Nov;23(6):751-753. doi: 10.1111/tbj.12817. Epub 2017 Apr 10. PMID: 28397341. Link <https://pubmed.ncbi.nlm.nih.gov/28397341/>
15. Krishnamurthy S, Singh R, Chandrasekaran V, Mathiyazhagan G, Chidambaram M, Deepak Barathi S, Mahadevan S. Basidiobolomycosis complicated by hydronephrosis and a perinephric abscess presenting as a hypertensive emergency in a 7-year-old boy. Paediatr Int Child Health. 2018 May;38(2):146-149. doi: 10.1080/20469047.2016.1162392. Epub 2017 Jan 23. PMID: 28112037. Link <https://pubmed.ncbi.nlm.nih.gov/28112037/>
16. Rajan RJ, Mohanraj P, Rose W. Subcutaneous Basidiobolomycosis Resembling Fournier's Gangrene. J Trop Pediatr. 2017 Jun 1;63(3):217-220. doi: 10.1093/tropej/fmw075. PMID: 27794531. Link <https://pubmed.ncbi.nlm.nih.gov/27794531/>
17. Sanaei Dashti A, Nasimfar A, Hosseini Khorami H, Pouladfar G, Kadivar MR, Geramizadeh B, Khalifeh M. Gastro-intestinal basidiobolomycosis in a 2-year-old boy: dramatic response to potassium iodide. Paediatr Int Child Health. 2018 May;38(2):150-153. doi: 10.1080/20469047.2016.1186343. Epub 2016 Jul 4. PMID: 27376878. Link <https://pubmed.ncbi.nlm.nih.gov/27376878/>
18. Mandhan P, Hassan KO, Samaan SM, Ali MJ. Visceral basidiobolomycosis: An overlooked infection in immunocompetent children. Afr J Paediatr Surg. 2015 Jul-Sep;12(3):193-6. doi: 10.4103/0189-6725.170218. PMID: 26612126; PMCID: PMC4955431. Link <https://pubmed.ncbi.nlm.nih.gov/26612126/>
19. Hamid ME, Joseph MR, Al-Qahtani AS. Chronic rhinofacial basidiobolomycosis caused by Basidiobolus ranarum: Report of a case from Aseer Region, Kingdom of Saudi Arabia. J Mycol Med. 2015 Dec;25(4):306-9. doi: 10.1016/j.mycmed.2015.09.001. Epub 2015 Oct 21. PMID: 26482354. Link <https://pubmed.ncbi.nlm.nih.gov/26482354/>
20. Al-Naemi AQ, Khan LA, Al-Naemi I, Amin K, Athlawy YA, Awad A, Sun Z. A Case Report of Gastrointestinal Basidiobolomycosis Treated With Voriconazole: A Rare Emerging Entity. Medicine (Baltimore). 2015 Sep;94(35):e1430. doi: 10.1097/MD.0000000000001430. PMID: 26334903; PMCID: PMC4616499. Link <https://pubmed.ncbi.nlm.nih.gov/26334903/>
21. Albaradi BA, Babiker AM, Al-Qahtani HS. Successful treatment of gastrointestinal basidiobolomycosis with voriconazole without surgical intervention. J Trop Pediatr. 2014 Dec;60(6):476-9. doi: 10.1093/tropej/fmu047. Epub 2014 Sep 10. PMID: 25213739. Link <https://pubmed.ncbi.nlm.nih.gov/25213739/>
22. Flicek KT, Vikram HR, De Petris GD, Johnson CD. Abdominal imaging findings in gastrointestinal basidiobolomycosis. Abdom Imaging. 2015 Feb;40(2):246-50. doi: 10.1007/s00261-014-0212-z. PMID: 25099255. Link <https://pubmed.ncbi.nlm.nih.gov/25099255/>
23. Cazorla A, Grenouillet F, Piton G, Faure É, Delabrousse É, Mathieu P, Viennet G, Kantelip B, Millon L, Valmary-Degano S. Une forme gastro-intestinale de basidiobolomycose d'évolution fatale [A letal case of gastro-intestinal basidiobolomycosis]. Ann Pathol. 2014 Jun;34(3):228-32. French. doi: 10.1016/j.annpat.2014.03.006. Epub 2014 May 22. PMID: 24950873. Link <https://pubmed.ncbi.nlm.nih.gov/24950873/>
24. Zabolinejad N, Naseri A, Davoudi Y, Joudi M, Aelami MH. Colonic basidiobolomycosis in a child: report of a culture-proven case. Int J Infect Dis. 2014 May;22:41-3. doi: 10.1016/j.ijid.2013.11.016. Epub 2014 Mar 12. PMID: 24614138. Link <https://pubmed.ncbi.nlm.nih.gov/24614138/>
25. AlSaleem K, Al-Mehaidib A, Banemai M, bin-Hussain I, Faqih M, Al Mehmadi A. Gastrointestinal basidiobolomycosis: mimicking Crohns disease case report and review of the literature. Ann Saudi Med. 2013 Sep-Oct;33(5):500-4. doi: 10.5144/0256-4947.2013.500. PMID: 24188947; PMCID: PMC6074897. Link <https://pubmed.ncbi.nlm.nih.gov/24188947/>
26. Pandit V, Rhee P, Aziz H, Jehangir Q, Friese RS, Joseph B. Perforated appendicitis with gastrointestinal basidiobolomycosis: a rare finding. Surg Infect (Larchmt). 2014 Jun;15(3):339-42. doi: 10.1089/sur.2012.188. Epub 2013 Nov 4. PMID: 24180345. Link <https://pubmed.ncbi.nlm.nih.gov/24180345/>
27. Zahir ST, Sharahjin NS, Kargar S. Basidiobolomycosis a mysterious fungal infection mimic small intestinal and colonic tumour with renal insufficiency and ominous outcome. BMJ Case Rep. 2013 Jul 26;2013:bcr2013200244. doi: 10.1136/bcr-2013-200244. PMID: 23893284; PMCID: PMC3736213. Link <https://pubmed.ncbi.nlm.nih.gov/23893284/>
28. Geramizadeh B, Foroughi R, Keshtkar-Jahromi M, Malek-Hosseini SA, Alborzi A. Gastrointestinal basidiobolomycosis, an emerging infection in the immunocompetent host: a report of 14 patients. J Med Microbiol. 2012 Dec;61(Pt 12):1770-1774. doi: 10.1099/jmm.0.046839-0. Epub 2012 Aug 23. PMID: 22918871. Link <https://pubmed.ncbi.nlm.nih.gov/22918871/>
29. Gordon CL, Whiting S, Haran G, Ward A, Coleman M, Baird R, Jeremiah CJ, Mileva M, Davis JS, Warren LJ, Ellis DH, Currie BJ. Entomophthoromycosis caused by Basidiobolus ranarum in tropical northern Australia. Pathology. 2012 Jun;44(4):375-9. doi: 10.1097/PAT.0b013e328353e912. PMID: 22565326. Link <https://pubmed.ncbi.nlm.nih.gov/22565326/>
30. Saadah OI, Farouq MF, Daajani NA, Kamal JS, Ghanem AT. Gastrointestinal basidiobolomycosis in a child; an unusual fungal infection mimicking fistulising Crohn's disease. J Crohns Colitis. 2012 Apr;6(3):368-72. doi: 10.1016/j.crohns.2011.10.008. Epub 2011 Nov 17. PMID: 22405176. Link <https://pubmed.ncbi.nlm.nih.gov/22405176/>
31. Mendiratta V, Karmakar S, Jain A, Jabeen M. Severe cutaneous zygomycosis due to Basidiobolus ranarum in a young infant. Pediatr Dermatol. 2012 Jan-Feb;29(1):121-3. doi: 10.1111/j.1525-1470.2011.01476.x. Epub 2011 Sep 9. PMID: 21906146. Link <https://pubmed.ncbi.nlm.nih.gov/21906146/>
32. El-Shabrawi MHF, Kamal NM, Jouini R, Al-Harbi A, Voigt K, Al-Malki T. Gastrointestinal basidiobolomycosis: an emerging fungal infection causing bowel perforation in a child. J Med Microbiol. 2011 Sep;60(Pt 9):1395-1402. doi: 10.1099/jmm.0.028613-0. Epub 2011 May 12. PMID: 21566088. Link <https://pubmed.ncbi.nlm.nih.gov/21566088/>
33. El-Shabrawi MHF, Kamal NM, Jouini R, Al-Harbi A, Voigt K, Al-Malki T. Gastrointestinal basidiobolomycosis: an emerging fungal infection causing bowel perforation in a child. J Med Microbiol. 2011 Sep;60(Pt 9):1395-1402. doi: 10.1099/jmm.0.028613-0. Epub 2011 May 12. PMID: 21566088. Link <https://pubmed.ncbi.nlm.nih.gov/21566088/>
34. Arya AA, Naik C, Desmukh S, Babanagare SV, Muntode P. Mastoid infection caused by entomophthorales: a rare fungal disease. J Laryngol Otol. 2011 Jun;125(6):630-2. doi: 10.1017/S0022215111000661. Epub 2011 Apr 7. PMID: 21470443. Link <https://pubmed.ncbi.nlm.nih.gov/21470443/>
35. Saka B, Kombaté K, Mouhari-Toure A, Akakpo S, Tchangaï B, Amégbor K, Pitché P, Tchangaï-Walla K. Basidiobolomycose probable chez un jeune rural togolais traitée avec succès par du kétoconazole [Probable basidiobolomycosis in a Togolese rural young successfully treated with ketoconazole]. Bull Soc Pathol Exot. 2010 Dec;103(5):293-5. French. doi: 10.1007/s13149-010-0090-8. Epub 2010 Oct 13. PMID: 20949344. Link <https://pubmed.ncbi.nlm.nih.gov/20949344/>
36. Goyal A, Gupta N, Das S, Jain S. Basidiobolomycosis of the nose and face: a case report and a mini-review of unusual cases of basidiobolomycosis. Mycopathologia. 2010 Sep;170(3):165-8. doi: 10.1007/s11046-010-9310-9. Epub 2010 Apr 7. PMID: 20373026. Link <https://pubmed.ncbi.nlm.nih.gov/20373026/>
37. Nemenqani D, Yaqoob N, Khoja H, Al Saif O, Amra NK, Amr SS. Gastrointestinal basidiobolomycosis: an unusual fungal infection mimicking colon cancer. Arch Pathol Lab Med. 2009 Dec;133(12):1938-42. doi: 10.5858/133.12.1938. PMID: 19961248. Link <https://pubmed.ncbi.nlm.nih.gov/19961248/>
38. Hussein MR, Musalam AO, Assiry MH, Eid RA, El Motawa AM, Gamel AM. Histological and ultrastructural features of gastrointestinal basidiobolomycosis. Mycol Res. 2007 Aug;111(Pt 8):926-30. doi: 10.1016/j.mycres.2007.06.009. Epub 2007 Jun 29. PMID: 17719761. Link <https://pubmed.ncbi.nlm.nih.gov/17719761/>
39. van den Berk GE, Noorduyn LA, van Ketel RJ, van Leeuwen J, Bemelman WA, Prins JM. A fatal pseudo-tumour: disseminated basidiobolomycosis. BMC Infect Dis. 2006 Sep 15;6:140. doi: 10.1186/1471-2334-6-140. PMID: 16978407; PMCID: PMC1574330. Link <https://pubmed.ncbi.nlm.nih.gov/16978407/>
40. Vianna LM, de Lacerda MV, de Moraes MA. Case report of subcutaneous entomophthoromycosis with retroperitoneal invasion. Rev Soc Bras Med Trop. 2005 Jul-Aug;38(4):348-50. doi: 10.1590/s0037-86822005000400014. Epub 2005 Aug 3. PMID: 16082485. Link <https://pubmed.ncbi.nlm.nih.gov/16082485/>
41. Mathew R, Kumaravel S, Kuruvilla S, Varghese RG, Shashikala, Srinivasan S, Mani MZ. Successful treatment of extensive basidiobolomycosis with oral itraconazole in a child. Int J Dermatol. 2005 Jul;44(7):572-5. doi: 10.1111/j.1365-4632.2004.02419.x. PMID: 15985026. Link <https://pubmed.ncbi.nlm.nih.gov/15985026/>
42. Choonhakarn C, Inthraburan K. Concurrent subcutaneous and visceral basidiobolomycosis in a renal transplant patient. Clin Exp Dermatol. 2004 Jul;29(4):369-72. doi: 10.1111/j.1365-2230.2004.01533.x. PMID: 15245532. Link <https://pubmed.ncbi.nlm.nih.gov/15245532/>
43. Bigliazzi C, Poletti V, Dell'Amore D, Saragoni L, Colby TV. Disseminated basidiobolomycosis in an immunocompetent woman. J Clin Microbiol. 2004 Mar;42(3):1367-9. doi: 10.1128/JCM.42.3.1367-1369.2004. PMID: 15004122; PMCID: PMC356830. Link <https://pubmed.ncbi.nlm.nih.gov/15004122/>
44. Al Jarie A, Al-Mohsen I, Al Jumaah S, Al Hazmi M, Al Zamil F, Al Zahrani M, Al Modovar E, Al Dayel F, Al Arishii H, Shehrani D, Martins J, Al Mehaidib A, Rossi L, Olaiyan I, Le Quesne G, Al-Mazrou A. Pediatric gastrointestinal basidiobolomycosis. Pediatr Infect Dis J. 2003 Nov;22(11):1007-14. doi: 10.1097/01.inf.0000095166.94823.11. PMID: 14614376. Link <https://pubmed.ncbi.nlm.nih.gov/14614376/>
45. Khan ZU, Khoursheed M, Makar R, Al-Waheeb S, Al-Bader I, Al-Muzaini A, Chandy R, Mustafa AS. Basidiobolus ranarum as an etiologic agent of gastrointestinal zygomycosis. J Clin Microbiol. 2001 Jun;39(6):2360-3. doi: 10.1128/JCM.39.6.2360-2363.2001. PMID: 11376094; PMCID: PMC88148. Link <https://pubmed.ncbi.nlm.nih.gov/11376094/>
46. Lyon GM, Smilack JD, Komatsu KK, Pasha TM, Leighton JA, Guarner J, Colby TV, Lindsley MD, Phelan M, Warnock DW, Hajjeh RA. Gastrointestinal basidiobolomycosis in Arizona: clinical and epidemiological characteristics and review of the literature. Clin Infect Dis. 2001 May 15;32(10):1448-55. doi: 10.1086/320161. Epub 2001 Apr 20. PMID: 11317246. Link <https://pubmed.ncbi.nlm.nih.gov/11317246/>
47. Nguyen BD. CT features of basidiobolomycosis with gastrointestinal and urinary involvement. AJR Am J Roentgenol. 2000 Mar;174(3):878-9. doi: 10.2214/ajr.174.3.1740878. PMID: 10701653. Link <https://pubmed.ncbi.nlm.nih.gov/10701653/>
48. Yousef OM, Smilack JD, Kerr DM, Ramsey R, Rosati L, Colby TV. Gastrointestinal basidiobolomycosis. Morphologic findings in a cluster of six cases. Am J Clin Pathol. 1999 Nov;112(5):610-6. doi: 10.1093/ajcp/112.5.610. PMID: 10549247. Link <https://pubmed.ncbi.nlm.nih.gov/10549247/>
49. Smilack JD. Gastrointestinal basidiobolomycosis. Clin Infect Dis. 1998 Sep;27(3):663-4. doi: 10.1086/517154. PMID: 9770184. Link <https://pubmed.ncbi.nlm.nih.gov/9770184/>
50. Khan ZU, Prakash B, Kapoor MM, Madda JP, Chandy R. Basidiobolomycosis of the rectum masquerading as Crohn's disease: case report and review. Clin Infect Dis. 1998 Feb;26(2):521-3. doi: 10.1086/517107. PMID: 9502495. Link | <https://pubmed.ncbi.nlm.nih.gov/9502495/>
51. Sood S, Sethi S, Banerjee U. Entomophthoromycosis due to Basidiobolus haptosporus. Mycoses. 1997 Dec;40(9-10):345-6. doi: 10.1111/j.1439-0507.1997.tb00247.x. PMID: 9470419. Link <https://pubmed.ncbi.nlm.nih.gov/9470419/>
52. Pasha TM, Leighton JA, Smilack JD, Heppell J, Colby TV, Kaufman L. Basidiobolomycosis: an unusual fungal infection mimicking inflammatory bowel disease. Gastroenterology. 1997 Jan;112(1):250-4. doi: 10.1016/s0016-5085(97)70242-7. PMID: 8978366. Link <https://pubmed.ncbi.nlm.nih.gov/8978366/>
53. Bittencourt AL, Arruda SM, de Andrade JA, Carvalho EM. Basidiobolomycosis: a case report. Pediatr Dermatol. 1991 Dec;8(4):325-8. doi: 10.1111/j.1525-1470.1991.tb00943.x. PMID: 1792208. Link <https://pubmed.ncbi.nlm.nih.gov/1792208/>
54. Antonelli M, Vignetti P, Dahir M, Mohamed MS, Favah AA. Entomophthoromycosis due to Basidiobolus in Somalia. Trans R Soc Trop Med Hyg. 1987;81(2):186-7. doi: 10.1016/0035-9203(87)90209-4. PMID: 3617175. Link <https://pubmed.ncbi.nlm.nih.gov/3617175/>
