# Supplementary material for: An old confusion: Entomophthoromycosis versus mucormycosis and their main differences
Source: Front Microbiol. 2022 Nov 3;13:1035100. doi: 10.3389/fmicb.2022.1035100 (PMC9670544; doi:10.3389/fmicb.2022.1035100)
Supplement: Supplementary file 2 [file Data_Sheet_2.docx]

**Supplement B: conidiobolomycosis**

Methodology: Systematic reviews were performed following the PRISMA 2020 criteria (Page et al., 2021).

Search parameters: Pubmed database. term conidiobolomycosis and *Conidiobolus* (*Conidiobolus coronatus* synonyms: *Boudierella coronata, Conidiobolus villosus, Delacroixia coronata, Entomophthora coronata* – [Speciesfungorum](http://www.speciesfungorum.org/) Access 13 September 2022).

Filters: human, 1978-29.8.2022, case reports, access to the full article.

Inclusion criteria: *Conidiobolus* spp. confirmed infections, the case report allow identification of the reporting country.

Exclusion criteria: All cases that did not meet the inclusion criteria were excluded.

Research results: 30 results

Manuscripts that met the inclusion criteria: 26

Number of cases: 32

1. Gupta P, Pilania RK, Kaur H, Rudramurthy SM, Soni R, Batra N, Verma R, Singh S, Chatterjee D. Pediatric case of conidiobolomycosis: A rare entity. Pediatr Dermatol. 2022 Jan;39(1):149-150. doi: 10.1111/pde.14875. Epub 2021 Dec 21. PMID: 34935187. Link <https://pubmed.ncbi.nlm.nih.gov/34935187/>
2. Purohit G, Sable M, Rudramurthy SM, Sarkar S, Parida P, Deshmukh V, Hallur V. A rare case of condiobolomycosis due to Conidiobolus coronatus presenting with dysphagia. Indian J Med Microbiol. 2021 Oct-Dec;39(4):558-560. doi: 10.1016/j.ijmmb.2021.03.022. Epub 2021 Apr 21. PMID: 33892974. Link <https://pubmed.ncbi.nlm.nih.gov/33892974/>
3. Stavropoulou E, Coste AT, Beigelman-Aubry C, Letovanec I, Spertini O, Lovis A, Krueger T, Burger R, Bochud PY, Lamoth F. Conidiobolus pachyzygosporus invasive pulmonary infection in a patient with acute myeloid leukemia: case report and review of the literature. BMC Infect Dis. 2020 Jul 22;20(1):527. doi: 10.1186/s12879-020-05218-w. PMID: 32698804; PMCID: PMC7374966. Link <https://pubmed.ncbi.nlm.nih.gov/32698804/>
4. Sigera LSM, Janappriya GHDC, Lakshan MTD, Pitigalage NJ, Jayasekera PI, Dayasena RP, Patabendige CGUA, Gunasekera CN. Rhinofacial Conidiobolomycosis: A Case Series and Review of the Literature. Ear Nose Throat J. 2021 Sep;100(5_suppl):835S-841S. doi: 10.1177/0145561319892475. Epub 2020 Mar 23. PMID: 32204618. Link <https://pubmed.ncbi.nlm.nih.gov/32204618/>
5. Deak L, Mudalagiriyappa S, Ballin A, Saxton D, Chakrabarti A. A Rhinofacial Conidiobolus coronatus Fungal Infection Presenting as an Intranasal Tumour. Sultan Qaboos Univ Med J. 2018 Nov;18(4):e549-e552. doi: 10.18295/squmj.2018.18.04.022. Epub 2019 Mar 28. PMID: 30988980; PMCID: PMC6443265. Link <https://pubmed.ncbi.nlm.nih.gov/30988980/>
6. AbdullGaffar B. Intranasal Rhinofacial Conidiobolomycosis (Entomophthoromycosis) With Splendore-Hoeppli Phenomenon. Int J Surg Pathol. 2019 Feb;27(1):67-68. doi: 10.1177/1066896918767552. Epub 2018 Apr 6. PMID: 29623742. Link <https://pubmed.ncbi.nlm.nih.gov/29623742/>
7. Cao C, Khader JA. Rhinofacial Entomophthoromycosis. N Engl J Med. 2018 Mar 1;378(9):e13. doi: 10.1056/NEJMicm1709956. PMID: 29490172. Link <https://pubmed.ncbi.nlm.nih.gov/29490172/>
8. Bamba S, Konsegré V, Zida A, Sangaré I, Cissé M, Beogo R, Diallo B, Andonaba JB, Guiguemdé RT. Un cas d’entomophthoromycose rhinofaciale en climat tropical soudano-sahélien au Burkina Faso [A case of rhinofacial entomophthoromycosis in Soudano-Sahelian tropical climate in Burkina Faso]. J Mycol Med. 2017 Jun;27(2):254-260. French. doi: 10.1016/j.mycmed.2017.01.002. Epub 2017 Feb 14. PMID: 28214142. Link <https://pubmed.ncbi.nlm.nih.gov/28214142/>
9. Ramirez J, Maguina P. Invasive Conidiobolomycosis Can Be Successfully Treated on Burn Survivors. J Burn Care Res. 2017 Jan/Feb;38(1):e460-e463. doi: 10.1097/BCR.0000000000000365. PMID: 27294856. Link <https://pubmed.ncbi.nlm.nih.gov/27294856/>
10. Cherian LM, Varghese L, Panchatcharam BS, Parmar HV, Varghese GM. Nasal conidiobolomycosis: a successful treatment option for localized disease. J Postgrad Med. 2015 Apr-Jun;61(2):143-4. doi: 10.4103/0022-3859.153112. PMID: 25766357; PMCID: PMC4943445. Link <https://pubmed.ncbi.nlm.nih.gov/25766357/>
11. Yu WN, Chen CJ, Chen CM. Pharyngeal entomophthoromycosis. J Chin Med Assoc. 2014 Jun;77(6):337-40. doi: 10.1016/j.jcma.2014.02.013. Epub 2014 Apr 18. PMID: 24746408. Link <https://pubmed.ncbi.nlm.nih.gov/24746408/>
12. Twizeyimana E, Chauty A, Pihet M, Ardant MF, Adeye A, Zidane M, de Gentile L, Saint-André JP, Chabasse D. Conidiobolomycose rhinofaciale avec localisations cervicales, thoraciques et brachiales: à propos d'un cas nigérian [Rhinofacial conidiobolomycosis associated with cervical, thoracic and brachial localizations: one clinical case in Nigeria]. J Mycol Med. 2014 Mar;24(1):48-55. French. doi: 10.1016/j.mycmed.2013.12.001. Epub 2014 Jan 17. PMID: 24440611. Link <https://pubmed.ncbi.nlm.nih.gov/24440611/>
13. Choon SE, Kang J, Neafie RC, Ragsdale B, Klassen-Fischer M, Carlson JA. Conidiobolomycosis in a young Malaysian woman showing chronic localized fibrosing leukocytoclastic vasculitis: a case report and meta-analysis focusing on clinicopathologic and therapeutic correlations with outcome. Am J Dermatopathol. 2012 Jul;34(5):511-22. doi: 10.1097/DAD.0b013e31823db5c1. PMID: 22728716. Link <https://pubmed.ncbi.nlm.nih.gov/22728716/>
14. Kimura M, Yaguchi T, Sutton DA, Fothergill AW, Thompson EH, Wickes BL. Disseminated human conidiobolomycosis due to Conidiobolus lamprauges. J Clin Microbiol. 2011 Feb;49(2):752-6. doi: 10.1128/JCM.01484-10. Epub 2010 Dec 8. PMID: 21147951; PMCID: PMC3043483. Link <https://pubmed.ncbi.nlm.nih.gov/21147951/>
15. Bento DP, Tavares R, Martins Mda L, Faria N, Maduro AP, Araújo C, Ventura F, Mansinho K. Atypical presentation of entomophthoromycosis caused by Conidiobolus coronatus. Med Mycol. 2010 Dec;48(8):1099-104. doi: 10.3109/13693786.2010.497973. Epub 2010 Jul 7. PMID: 20608778. Link <https://pubmed.ncbi.nlm.nih.gov/20608778/>
16. Radhakrishnan N, Sachdeva A, Oberoi J, Yadav SP. Conidiobolomycosis in relapsed acute lymphoblastic leukemia. Pediatr Blood Cancer. 2009 Dec 15;53(7):1321-3. doi: 10.1002/pbc.22259. PMID: 19731329. Link <https://pubmed.ncbi.nlm.nih.gov/19731329/>
17. Yang X, Li Y, Zhou X, Wang Y, Geng S, Liu H, Yang Q, Lu X, Hiruma M, Sugita T, Ikeda S, Ogawa H. Rhinofacial conidiobolomycosis caused by Conidiobolus coronatus in a Chinese rice farmer. Mycoses. 2010 Jul;53(4):369-73. doi: 10.1111/j.1439-0507.2009.01716.x. Epub 2009 May 5. PMID: 19422524. Link <https://pubmed.ncbi.nlm.nih.gov/19422524/>
18. Barro-Traoré F, Ouédraogo D, Konsem T, Ouédraogo MS, Lompo-Goumbri O, Sanou A, Ouoba K, Traoré A. Conidiobolomycose, une tumeur fongique rare: un cas a Ouagadougou, Burkina Faso [Conidiobolomycosis, a rare fungal tumor: a case report in Ouagadougou, Burkina Faso]. Bull Soc Pathol Exot. 2008 Feb;101(1):14-6. French. PMID: 18431999. Link <https://pubmed.ncbi.nlm.nih.gov/18431999/>
19. Receveur MC, Roussin C, Mienniel B, Gasnier O, Rivière JP, Malvy D, Lortholary O. Entomophthoromycose rhinofaciale. A propos de deux nouveaux cas diagnostiqués a Mayotte [Rhinofacial entomophthoromycosis. About two new cases in Mayotte]. Bull Soc Pathol Exot. 2005 Dec;98(5):350-3. French. PMID: 16425711. Link <https://pubmed.ncbi.nlm.nih.gov/16425711/>
20. Pérez JA, Correa A, Fuentes J, Meléndez E. Conidiobolomicosis: hallazgos histopatológicos [Conidiobolomycosis: a case report with histophathologic findings]. Biomedica. 2004 Dec;24(4):350-5. Spanish. PMID: 15678798. Link <https://pubmed.ncbi.nlm.nih.gov/15678798/>
21. Pecarrere JL, Huerre M, Lafond P, Esterre P, Raharisolo C, De Rotalier P. Les entomophthoromycoses à Madagascar (à propos de trois cas malgaches) [Entomophthoromycoses in Madagascar (three cases)]. Arch Inst Pasteur Madagascar. 1994;61(2):99-102. French. PMID: 7575044. Link <https://pubmed.ncbi.nlm.nih.gov/7575044/>
22. de Castro e Souza Filho LG, Nico MM, Salebian A, Heins-Vaccari EM, de Castro LG, Sotto MN, Lacaz Cda S, Martins JE, Wu SL, Cucé LC. Entomoftoromicose rinofacial por Conidiobolus coronatus. Registro de um caso tratado com sucesso pelo fluconazol [Rhinofacial entomophthoromycosis due to Conidiobolus coronatus. A report of a case treated successfully with fluconazole]. Rev Inst Med Trop Sao Paulo. 1992 Sep-Oct;34(5):483-7. Portuguese. PMID: 1342115. Link <https://pubmed.ncbi.nlm.nih.gov/1342115/>
23. Michel G, Ravisse P, Lohoue-Petmy J, Steinmetz JP, Winter C, Mbakop A, Ave P, Ruffaud MA. Cinq nouveaux cas d'entomophthoromycose observés au Cameroun. Place de l'immunofluorescence dans le diagnostic [5 new cases of entomophthoromycosis observed in Cameroon. Role of immunofluorescence in thr diagnosis]. Bull Soc Pathol Exot. 1992;85(1):10-6. French. PMID: 1596951. Link <https://pubmed.ncbi.nlm.nih.gov/1596951/>
24. ebrie JC, Conessa C, Martet G, Lecamus JL, Touze JE. Approches nouvelles des phycomycoses (à propos de deux cas de rhino-entomophtoroses) [New approaches to phycomycoses (apropos of 2 cases of rhino-entomophthoromycosis)]. Med Trop (Mars). 1991 Apr-Jun;51(2):191-200. French. PMID: 1895919. Link <https://pubmed.ncbi.nlm.nih.gov/1895919/>
25. Moreno JL, de Rodríguez GC, Morón AE, Brown A, Burrows O. Un caso de entomophthoromicosis por Conidiobolus coronatus [A case of entomophthoromycosis caused by Conidiobolus coronatus]. Rev Med Panama. 1990 Sep;15(3):204-10. Spanish. PMID: 2284450. Link <https://pubmed.ncbi.nlm.nih.gov/2284450/>
26. Kamalam A, Thambiah AS. Lymph node invasion by Conidiobolus coronatus and its spore formation in vivo. Sabouraudia. 1978 Sep;16(3):175-84. PMID: 568318. Link <https://pubmed.ncbi.nlm.nih.gov/568318/>
