# Supplementary material for: An old confusion: Entomophthoromycosis versus mucormycosis and their main differences
Source: Front Microbiol. 2022 Nov 3;13:1035100. doi: 10.3389/fmicb.2022.1035100 (PMC9670544; doi:10.3389/fmicb.2022.1035100)
Supplement: Supplementary file 3 [file Data_Sheet_3.docx]

**Supplement C: mucormycosis**

Methodology: Systematic reviews were performed following the PRISMA 2020 criteria (Page et al., 2021).

Search parameters: Pubmed database. Term mucormycosis.

Filters: human, 2000-22-8-2022, case reports, access to the full article.

Inclusion criteria: Mucorales confirmed infections, the case report allow identification of the reporting country.

Exclusion criteria: All cases that did not meet the inclusion criteria were excluded.

Research results: 1,480 results

Manuscripts that met the inclusion criteria: 1304

Number of cases: 1344, some case reports included more than one patient.

1. Mandke C, Divekar R, Pradhan V, Arora S. Quantitative B and T cell abnormalities in four patients presenting with mucormycosis and prior asymptomatic COVID-19 infection. BMJ Case Rep. 2022 Aug 9;15(8):e247893. doi: 10.1136/bcr-2021-247893. PMID: 35944939. Link: <https://pubmed.ncbi.nlm.nih.gov/35944939/>
2. Fujiwara Y, Tobita H, Mochizuki N, Inomata T, Asano T, Ohishi H, Kanamitsu H, Kubonishi S, Mohri M, Hiramatsu Y. [Cunninghamella bertholletiae-infective endocarditis complicated by tricuspid valve giant vegetation in a patient with aplastic anemia]. Rinsho Ketsueki. 2022;63(7):740-745. Japanese. doi: 10.11406/rinketsu.63.740. PMID: 35922941. Link: https://pubmed.ncbi.nlm.nih.gov/35922941/
3. Luo S, Huang X, Li Y, Wang J. Isolated splenic mucormycosis secondary to diabetic ketoacidosis: a case report. BMC Infect Dis. 2022 Jul 7;22(1):596. doi: 10.1186/s12879-022-07564-3. PMID: 35799111; PMCID: PMC9264645. Link: https://pubmed.ncbi.nlm.nih.gov/35799111/
4. Khan MM, Hasnain SA, Hussain A, Faisal MJ. Black fungus: A rising consternation among COVID patients presenting to a tertiary care hospital in Islamabad-Pakistan Institute of Medical Sciences. J Pak Med Assoc. 2022 Jun;72(6):1225-1228. doi: 10.47391/JPMA.4225. PMID: 35751343. Link https://pubmed.ncbi.nlm.nih.gov/35751343/
5. Serris A, Ouedrani A, Uhel F, Gazzano M, Bedarida V, Rouzaud C, Bougnoux ME, Raphalen JH, Poirée S, Lambotte O, Martin-Blondel G, Lanternier F. Case Report: Immune Checkpoint Blockade Plus Interferon-Γ Add-On Antifungal Therapy in the Treatment of Refractory Covid-Associated Pulmonary Aspergillosis and Cerebral Mucormycosis. Front Immunol. 2022 Jun 1;13:900522. doi: 10.3389/fimmu.2022.900522. PMID: 35720319; PMCID: PMC9199385. Link: https://pubmed.ncbi.nlm.nih.gov/35720319/
6. Munhoz SD, Lellis RF, Reis APC, Del Negro GMB, Sousa MGT, Veasey JV. Rhino-orbito-cerebral mucormycosis caused by Rhizopus microsporus var. microsporus in a diabetic patient with COVID-19. An Bras Dermatol. 2022 Jul-Aug;97(4):501-504. doi: 10.1016/j.abd.2022.02.001. Epub 2022 Jun 9. PMID: 35691738; PMCID: PMC9181896. Link <https://pubmed.ncbi.nlm.nih.gov/35691738/>
7. Wu F, Tian J, She Z, Liu Y, Wan W, Wen C. [Clinical features of children with *Cunninghamella* spp. infection: a case report and literature review]. Nan Fang Yi Ke Da Xue Xue Bao. 2022 May 20;42(5):780-784. Chinese. doi: 10.12122/j.issn.1673-4254.2022.05.22. PMID: 35673925; PMCID: PMC9178633. Link <https://pubmed.ncbi.nlm.nih.gov/35673925/>
8. Yadav S, Saini A, Singh A, Verma H. Batwing incision for a case of bilateral fungal zygomatic osteomyelitis. BMJ Case Rep. 2022 May 31;15(5):e247387. doi: 10.1136/bcr-2021-247387. PMID: 35641085; PMCID: PMC9157357. Link https://pubmed.ncbi.nlm.nih.gov/35641085/
9. Shahid A, Fatima B, Khan YN, Ibrahim MN, Raza J. Early suspicion can save lives; mucormycosis in two children with diabetic ketoacidosis in Pakistan. J Pak Med Assoc. 2022 Apr;72(4):790-792. doi: 10.47391/JPMA.1659. PMID: 35614628. Link https://pubmed.ncbi.nlm.nih.gov/35614628/
10. Vadi S, Raut A, Shah S, Ismail AM. Post coronavirus disease- 19 invasive renal and gastrointestinal mucormycosis. Indian J Med Microbiol. 2022 Jul-Sep;40(3):462-464. doi: 10.1016/j.ijmmb.2022.03.006. Epub 2022 May 6. PMID: 35527119; PMCID: PMC9072856. Link https://pubmed.ncbi.nlm.nih.gov/35527119/
11. Ziaka M, Papakonstantinou E, Vasileiou E, Chorafa E, Antachopoulos C, Roilides E. Paediatric cutaneous mucormycosis: A case report and review of the literature. Mycoses. 2022 Jul;65(7):674-682. doi: 10.1111/myc.13452. Epub 2022 May 24. PMID: 35514044. Link https://pubmed.ncbi.nlm.nih.gov/35514044/
12. Shi X, Qi L, Du B, Yao X, Du X. Paraplegia secondary to disseminated mucormycosis: case report and literature review. BMC Infect Dis. 2022 Apr 25;22(1):405. doi: 10.1186/s12879-022-07373-8. PMID: 35468738; PMCID: PMC9036692. Link https://pubmed.ncbi.nlm.nih.gov/35468738/
13. Paliwal P, Rahar S, Sharma A, Gupta D, Ahuja A, Chauhan DS. Intestinal mucormycosis in a patient with COVID-19: A case report. Indian J Pathol Microbiol. 2022 Apr-Jun;65(2):475-477. doi: 10.4103/IJPM.IJPM_1436_20. PMID: 35435400. Link https://pubmed.ncbi.nlm.nih.gov/35435400/
14. Aparicio M, Tuculet B, Rivolier MG, Forte A, Vila A. Mucormicosis asociada a COVID-19. Un caso en Argentina [COVID-19 associated mucormycosis. A case in Argentina]. Medicina (B Aires). 2022;82(2):304-307. Spanish. PMID: 35417397. Link https://pubmed.ncbi.nlm.nih.gov/35417397/
15. Singh P, Gupta A, Sanepalli SR, Raj A. Transcutaneous retrobulbar amphotericin-B (TRAMB) injection in orbital mucormycosis. BMJ Case Rep. 2022 Mar 31;15(3):e246307. doi: 10.1136/bcr-2021-246307. PMID: 35361668; PMCID: PMC8971798. LINK https://pubmed.ncbi.nlm.nih.gov/35361668/
16. Reddy YM, Parida S, Reddy SB, Yeduguri S, Pidaparthi L, Jaiswal SK, Sadhvani B, Murthy JMK. Decoding "guitar pick sign" in COVID-19-associated mucormycosis: A case series. Indian J Ophthalmol. 2022 Apr;70(4):1425-1427. doi: 10.4103/ijo.IJO_2598_21. PMID: 35326073; PMCID: PMC9240521. LINK https://pubmed.ncbi.nlm.nih.gov/35326073/
17. Jian Y, Wang M, Yu Y, Zhuo Y, Xiao D, Lin S, Xuan J. Treatment and economic burden of mucormycosis in China: Case report review and burden estimation. J Clin Pharm Ther. 2022 Jul;47(7):905-914. doi: 10.1111/jcpt.13643. Epub 2022 Mar 18. PMID: 35304764. LINK https://pubmed.ncbi.nlm.nih.gov/35304764/
18. Taweesuk A, Chongtrakool P, Sitthinamsuwan P, Phoompoung P. Hematogenous dissemination of pulmonary mucormycosis manifested as multiple subcutaneous nodules: a case report and review of the literature. BMC Infect Dis. 2022 Mar 4;22(1):220. doi: 10.1186/s12879-022-07187-8. PMID: 35246058; PMCID: PMC8894548. Link https://pubmed.ncbi.nlm.nih.gov/35246058/
19. Shao B, Hagan MJ, Sastry RA, Kritselis M, Donahue JE, Toms SA. An Instructive Case of Cerebral Mucormycosis. R I Med J (2013). 2022 Mar 1;105(2):8-12. PMID: 35211702. Link https://pubmed.ncbi.nlm.nih.gov/35211702/
20. urien AA, Srinivasaprasad ND, Valavan KT. Renal infarction due to COVID-19-associated renal mucormycosis. Kidney Int. 2022 Mar;101(3):655. doi: 10.1016/j.kint.2021.07.027. PMID: 35190047; PMCID: PMC8854973. Link https://pubmed.ncbi.nlm.nih.gov/35190047/
21. Menezes S, Kumar JS, Rudra OS, Nagral A. Cutaneous mucormycosis: an unusual cause of decompensation in a patient with ethanol-related cirrhosis with COVID-19 exposure. BMJ Case Rep. 2022 Feb 9;15(2):e247399. doi: 10.1136/bcr-2021-247399. PMID: 35140094; PMCID: PMC8830195. Link https://pubmed.ncbi.nlm.nih.gov/35140094/
22. Yang Z, Zhang L, Cong Y, Liu ZY. [Type 1 diabetes mellitus complicated with gastric ulcer caused by mucormycosis infection: a case report]. Zhonghua Nei Ke Za Zhi. 2022 Feb 1;61(2):210-213. Chinese. doi: 10.3760/cma.j.cn112138-20210224-00158. PMID: 35090258. Link https://pubmed.ncbi.nlm.nih.gov/35090258/
23. Dave A, Joshi P, Jaiswal S, Kapdeo P. Successful outcome of severe COVID-19 in pregnancy: individualised approach. BMJ Case Rep. 2022 Jan 17;15(1):e246648. doi: 10.1136/bcr-2021-246648. PMID: 35039364; PMCID: PMC8768493. Link https://pubmed.ncbi.nlm.nih.gov/35039364/
24. Chandra A, Rao S N, Malhotra KP. Fatal allograft mucormycosis complicating severe COVID-19 infection and bacterial pyelonephritis. Transpl Infect Dis. 2022 Apr;24(2):e13793. doi: 10.1111/tid.13793. Epub 2022 Jan 28. PMID: 35029013. Link <https://pubmed.ncbi.nlm.nih.gov/35029013/>
25. Ingle Y, Sarode SC, Sarode G, Ingle M, Ingle S. Concurrent plexiform ameloblastoma and COVID-19-associated mucormycosis of the maxilla. Oral Oncol. 2022 Feb;125:105712. doi: 10.1016/j.oraloncology.2022.105712. Epub 2022 Jan 8. PMID: 35016140; PMCID: PMC8741420. Link https://pubmed.ncbi.nlm.nih.gov/35016140/
26. Melchers M, Festen B, den Dekker BM, Mooren ERM, van Binsbergen AL, van Bree SHW, Heusinkveld M, Schellaars R, Buil JB, Verweij PE, van Zanten ARH. A 67-Year-Old Male Patient With COVID-19 With Worsening Respiratory Function and Acute Kidney Failure. Chest. 2022 Jan;161(1):e5-e11. doi: 10.1016/j.chest.2021.08.045. PMID: 35000717; PMCID: PMC8733290. Link https://pubmed.ncbi.nlm.nih.gov/35000717/
27. Olivo Freites C, Sy H, Miguez P, Salonia J. Uncommon pathogens in an immunocompetent host: respiratory isolation of Cunninghamella bertholletiae, Aspergillus niger, Staphylococcus pseudintermedius and adenovirus in a patient with necrotising pneumonia. BMJ Case Rep. 2022 Jan 6;15(1):e240484. doi: 10.1136/bcr-2020-240484. PMID: 34992060; PMCID: PMC8738981. Link https://pubmed.ncbi.nlm.nih.gov/34992060/
28. Gökbulut Bektaş Ş, Kandemir AB, Ayaz ÇM, Yilmaz AN, İzdeş S. COVID-19-Related Rhino-Orbital-Cerebral Mucormycosis in a Renal Transplant Recipient. Exp Clin Transplant. 2022 Feb;20(2):213-217. doi: 10.6002/ect.2021.0317. Epub 2022 Jan 3. PMID: 34981710. Link <https://pubmed.ncbi.nlm.nih.gov/34981710/>
29. Shi L, Zhao X, Yan X, Liu Y, Liu Y, Cao H, Su K, Wang C, Gao S, Liu Q. Aggressive disseminated Rhizomucor pusillus infection in a Ph-like acute lymphoblastic leukemia patient: Early detection by cell-free DNA next-generation sequencing. J Infect Chemother. 2022 Mar;28(3):459-464. doi: 10.1016/j.jiac.2021.12.007. Epub 2021 Dec 23. PMID: 34955408. Link <https://pubmed.ncbi.nlm.nih.gov/34955408/>
30. Murthy R, Bagchi A, Gote Y, Desai S. Retrobulbar injection of amphotericin B using intravenous cannula for post-COVID-19 rhino-orbital mucormycosis. Indian J Ophthalmol. 2022 Jan;70(1):302-305. doi: 10.4103/ijo.IJO_1511_21. PMID: 34937263; PMCID: PMC8917598. Link <https://pubmed.ncbi.nlm.nih.gov/34937263/>
31. Kaushik KS, Acharya UV, Krupa L. Dual hit - Magnetic resonance imaging in concomitant anterior and posterior ischemic optic neuropathy in a case of rhino-orbital mucormycosis and COVID-19. Indian J Ophthalmol. 2022 Jan;70(1):300-301. doi: 10.4103/ijo.IJO_1312_21. PMID: 34937262; PMCID: PMC8917582. Link <https://pubmed.ncbi.nlm.nih.gov/34937262/>
32. Amanati A, Zekavat OR, Foroutan H, Azh O, Tadayon A, Monabati A, Anbardar MH, Bozorgi H. Case reports of invasive mucormycosis associated neutropenic enterocolitis in leukemic children: diagnostic and treatment challenges and review of literature. BMC Infect Dis. 2021 Dec 20;21(1):1268. doi: 10.1186/s12879-021-06957-0. PMID: 34930171; PMCID: PMC8686658. Link <https://pubmed.ncbi.nlm.nih.gov/34930171/>
33. Rajarajen AP, Sethi J, Shaji A, Singh AK, Mitra S, Vaiphei K. A Case of Esophageal Mucormycosis. Am J Gastroenterol. 2022 Feb 1;117(2):355. doi: 10.14309/ajg.0000000000001592. PMID: 34913879. Link <https://pubmed.ncbi.nlm.nih.gov/34913879/>
34. Aggarwal SK, Kaur U, Talda D, Pandey A, Jaiswal S, Kanakan A, Singh A, Chakrabarti SS. Case Report: Rhino-orbital Mucormycosis Related to COVID-19: A Case Series Exploring Risk Factors. Am J Trop Med Hyg. 2021 Dec 13;106(2):566-570. doi: 10.4269/ajtmh.21-0777. PMID: 34902834; PMCID: PMC8832906. Link <https://pubmed.ncbi.nlm.nih.gov/34902834/>
35. Vittitow SL, Rusu CA, Abubakar MO, Burnsed J, Gru AA, Zlotoff BJ. Primary cutaneous mucormycosis in a premature neonate treated conservatively with amphotericin B. Pediatr Dermatol. 2022 Jan;39(1):99-102. doi: 10.1111/pde.14879. Epub 2021 Dec 9. PMID: 34888931. Link <https://pubmed.ncbi.nlm.nih.gov/34888931/>
36. Wei LW, Zhu PQ, Chen XQ, Yu J. Mucormycosis in Mainland China: A Systematic Review of Case Reports. Mycopathologia. 2022 Feb;187(1):1-14. doi: 10.1007/s11046-021-00607-4. Epub 2021 Dec 2. PMID: 34855101; PMCID: PMC8637510. Link <https://pubmed.ncbi.nlm.nih.gov/34855101/>
37. Wang C, Yang Q, Liu N, Huang R, Fang M, Shao M. Emphysematous gastritis associated with mucormycosis in a patient with fulminant myocarditis requiring veno-arterial extracorporeal membrane oxygenation. Rev Esp Enferm Dig. 2022 Apr;114(4):236-237. doi: 10.17235/reed.2021.8477/2021. PMID: 34852632. Link <https://pubmed.ncbi.nlm.nih.gov/34852632/>
38. Crone CG, Helweg-Larsen J, Steensen M, Arendrup MC, Helleberg M. Pulmonary mucormycosis in the aftermath of critical COVID-19 in an immunocompromised patient: Mind the diagnostic gap. J Mycol Med. 2022 Mar;32(1):101228. doi: 10.1016/j.mycmed.2021.101228. Epub 2021 Nov 18. PMID: 34826672; PMCID: PMC8600800. Link <https://pubmed.ncbi.nlm.nih.gov/34826672/>
39. Kaur R, Khan B, Sharma A. Optical Coherence Tomography of Retinal Artery Occlusion Associated With Mucormycosis and COVID-19. JAMA Ophthalmol. 2021 Nov 1;139(11):e214064. doi: 10.1001/jamaophthalmol.2021.4064. Epub 2021 Nov 17. PMID: 34787678. Link <https://pubmed.ncbi.nlm.nih.gov/34787678/>
40. Yuan F, Chen J, Liu F, Dang YC, Kong QT, Sang H. Successful treatment of pulmonary mucormycosis caused by Rhizopus microsporus with posaconazole. Eur J Med Res. 2021 Nov 14;26(1):131. doi: 10.1186/s40001-021-00602-x. PMID: 34775981; PMCID: PMC8591890. Link <https://pubmed.ncbi.nlm.nih.gov/34775981/>
41. Dos Santos RLO, Elchin CB, Guiguer-Pinto VA, Vasconcelos DM, Ferreira MD, Dias RB, Sugaya NN, Paula CR, Coto NP. Diagnosis, treatment and maxillofacial rehabilitation in rhinocerebral mucormycosis patient: A case report and review of the literature. J Mycol Med. 2022 Mar;32(1):101211. doi: 10.1016/j.mycmed.2021.101211. Epub 2021 Oct 28. PMID: 34763149. Link <https://pubmed.ncbi.nlm.nih.gov/34763149/>
42. Teng P, Han X, Zhang S, Wei D, Wang Y, Liu D, Liu X. Mixed invasive pulmonary Mucor and Aspergillus infection: a case report and literature review. Chin Med J (Engl). 2022 Apr 5;135(7):854-856. doi: 10.1097/CM9.0000000000001839. PMID: 34759224; PMCID: PMC9276213. Link <https://pubmed.ncbi.nlm.nih.gov/34759224/>
43. Mendhe D, Wankhede P, Wanjari M, Alwadkar S. Mucormycotic osteomyelitis of maxilla post-COVID patient: a case report. Pan Afr Med J. 2021 Aug 27;39:275. doi: 10.11604/pamj.2021.39.275.30480. PMID: 34754352; PMCID: PMC8556725. Link <https://pubmed.ncbi.nlm.nih.gov/34754352/>
44. Ding WJ, Ma XY, Li L. [Rhinocerebral mucormycosis secondary to acute leukemia: a case report]. Zhonghua Er Bi Yan Hou Tou Jing Wai Ke Za Zhi. 2021 Nov 7;56(11):1207-1209. Chinese. doi: 10.3760/cma.j.cn115330-20210122-00030. PMID: 34749462. Link <https://pubmed.ncbi.nlm.nih.gov/34749462/>
45. Arora N, Gudipati A, Kundu R, Prabhakar N, Suri V, Malhotra P, Jain A. Post-COVID-19 mucormycosis presenting as chest wall cellulitis with mediastinitis. Lancet Infect Dis. 2021 Nov;21(11):1611. doi: 10.1016/S1473-3099(21)00582-X. PMID: 34717815; PMCID: PMC8550903. Link <https://pubmed.ncbi.nlm.nih.gov/34717815/>
46. Khera S, Singh V, Pattanayak S. Favourable outcome in a child with acute lymphoblastic leukaemia and pulmonary mucormycosis managed with combination antifungal therapy of liposomal amphotericin B and caspofungin. BMJ Case Rep. 2021 Oct 19;14(10):e245329. doi: 10.1136/bcr-2021-245329. PMID: 34667044; PMCID: PMC8527156. Link <https://pubmed.ncbi.nlm.nih.gov/34667044/>
47. Demiroğu YZ, Ödemiş İ, Oruç E, Özer F, Ulaş B, Canpolat ET, Yalçın Ç, Öğüç Şanlı Ö. COVID-19 Enfeksiyonu Sonrası Gelişen İki Rino-Orbito-Serebral Mukormikoz Olgusu [Two Case of Rhino-Orbito-Cerebral Mucormicosis Developed After COVID-19 Infection]. Mikrobiyol Bul. 2021 Oct;55(4):673-682. Turkish. doi: 10.5578/mb.20219719. PMID: 34666667. Link <https://pubmed.ncbi.nlm.nih.gov/34666667/>
48. Deek AJ, Boukovalas S, Rathfoot CJ, Gotcher JE. Rhinocerebral Mucormycosis as a Sequelae of COVID-19 Treatment: A Case Report & Literature Review. J Oral Maxillofac Surg. 2022 Feb;80(2):333-340. doi: 10.1016/j.joms.2021.09.009. Epub 2021 Sep 20. PMID: 34655530; PMCID: PMC8451406. Link <https://pubmed.ncbi.nlm.nih.gov/34655530/>
49. Holmes TR, Hepschke JL, Jacobson I, Maloof A. Mucormycosis: early treatment is the key to survival. Med J Aust. 2021 Nov 1;215(9):401-403. doi: 10.5694/mja2.51290. Epub 2021 Oct 9. PMID: 34628643. Link <https://pubmed.ncbi.nlm.nih.gov/34628643/>
50. Parmar KM, Akif S, Kumar S, Kaundal P. Isolated unilateral renal mucormycosis in a young immunocompetent male. BMJ Case Rep. 2021 Oct 8;14(10):e245309. doi: 10.1136/bcr-2021-245309. PMID: 34625442; PMCID: PMC8504170. Copy Download .nbib Form Link <https://pubmed.ncbi.nlm.nih.gov/34625442/>
51. Grach SL, Yetmar ZA, Rowan DJ, DeSimone DC. Rhinorbitocerebral Mucormycosis in Uncontrolled Diabetes. Mayo Clin Proc. 2021 Oct;96(10):2651-2652. doi: 10.1016/j.mayocp.2021.06.008. PMID: 34607635. Link <https://pubmed.ncbi.nlm.nih.gov/34607635/>
52. Kharbanda R, Mehndiratta A, Chatterjee R, Marak RSK, Aggarwal A. Pulmonary mucormycosis in systemic lupus erythematosus: successful management of a case along with review of literature. Clin Rheumatol. 2022 Jan;41(1):307-312. doi: 10.1007/s10067-021-05941-y. Epub 2021 Oct 4. PMID: 34606036; PMCID: PMC8488537. Link <https://pubmed.ncbi.nlm.nih.gov/34606036/>
53. Gupta D, Dosi T. A rare entity to major outbreak: a case report on mucormycosis. Pan Afr Med J. 2021 Jul 7;39:183. doi: 10.11604/pamj.2021.39.183.30479. PMID: 34584608; PMCID: PMC8449572. Link <https://pubmed.ncbi.nlm.nih.gov/34584608/>
54. Yuan NN, Guo LJ, Zhao L, Zhang S, Jing L, Li M, Liang CY, Lu BH, Chen JY, Chen WH. [Pulmonary mucormycosis after lung transplantation:3 cases report with literature review]. Zhonghua Jie He He Hu Xi Za Zhi. 2021 Oct 12;44(10):897-901. Chinese. doi: 10.3760/cma.j.cn112147-20210129-00084. PMID: 34565117. Link <https://pubmed.ncbi.nlm.nih.gov/34565117/>
55. Shimoyama K, Niwa T, Furukawa S, Morishita N, Nagakura Y, Yonezawa H, Hatakeyama M, Okubo Y, Suzuki D, Kosugi I, Shiogama K, Ogawa N. Behçet's Disease with Bilateral Renal Infarction Due to Mucormycosis. Intern Med. 2022 Apr 1;61(7):1077-1083. doi: 10.2169/internalmedicine.7462-21. Epub 2021 Sep 18. PMID: 34544946; PMCID: PMC9038466. Link <https://pubmed.ncbi.nlm.nih.gov/34544946/>
56. Nepomuceno Araújo MJCL, Nihei CH, Rodrigues AM, Higashino H, Ponzio V, Campos Pignatari AC, Barcellos MA, Braga O, Duayer IF. Case Report: Invasive Sinusitis due to Sporothrix Brasiliensis in a Renal Transplant Recipient. Am J Trop Med Hyg. 2021 Sep 20;105(5):1218-1221. doi: 10.4269/ajtmh.20-1602. PMID: 34544042; PMCID: PMC8592196. Link <https://pubmed.ncbi.nlm.nih.gov/34544042/>
57. Yadav S, Kumar R, Kumar R, Sagar P. Fungal central skull-base osteomyelitis: atypical presentation and management issues. BMJ Case Rep. 2021 Sep 16;14(9):e243530. doi: 10.1136/bcr-2021-243530. PMID: 34531233; PMCID: PMC8449942. Link <https://pubmed.ncbi.nlm.nih.gov/34531233/>
58. Ravindra CM, Rajaram M, Madhusmita M, ChToi P, Sneha L. Pulmonary Aspergillus and Mucor Co-Infection: A report of two cases. Sultan Qaboos Univ Med J. 2021 Aug;21(3):495-498. doi: 10.18295/squmj.8.2021.126. Epub 2021 Aug 29. PMID: 34522421; PMCID: PMC8407904. Link <https://pubmed.ncbi.nlm.nih.gov/34522421/>
59. Diwakar J, Samaddar A, Konar SK, Bhat MD, Manuel E, Hb V, Bn N, Parveen A, Hajira SN, Srinivas D, S N. First report of COVID-19-associated rhino-orbito-cerebral mucormycosis in pediatric patients with type 1 diabetes mellitus. J Mycol Med. 2021 Dec;31(4):101203. doi: 10.1016/j.mycmed.2021.101203. Epub 2021 Sep 4. PMID: 34517273; PMCID: PMC8418383. Link <https://pubmed.ncbi.nlm.nih.gov/34517273/>
60. Hubbard DC 2nd, Fleenor JW, Su MG, Tsai JH. A 47-year-old man with a necrotic wound after trauma. Digit J Ophthalmol. 2021 May 17;27(2):33-37. doi: 10.5693/djo.03.2020.12.001. PMID: 34512209; PMCID: PMC8406642. Link <https://pubmed.ncbi.nlm.nih.gov/34512209/>
61. Mahan KM, Molina MF, Coffey ECC, Manchanda ECC. New-Onset Pediatric Diabetes Complicated By Diabetic Ketoacidosis and Invasive Rhinocerebral Mucormycosis With Internal Carotid Artery Occlusion. J Emerg Med. 2022 Jan;62(1):95-100. doi: 10.1016/j.jemermed.2021.07.024. Epub 2021 Sep 9. PMID: 34509337. Link <https://pubmed.ncbi.nlm.nih.gov/34509337/>
62. Kumar Gupta S, Jyotsana P, Singh A, Phuyal D, Allam P. Rhinocerebral Mucormycosis in a COVID-19 Patient from Nepal: A Case Report. JNMA J Nepal Med Assoc. 2021 Jul 30;59(239):703-705. doi: 10.31729/jnma.6904. PMID: 34508493; PMCID: PMC9107863. Link <https://pubmed.ncbi.nlm.nih.gov/34508493/>
63. Jafarpour Z, Pouladfar G, Dehghan A, Anbardar MH, Foroutan HR. Case Report: Gastrointestinal Basidiobolomycosis with Multi-Organ Involvement Presented with Intussusception. Am J Trop Med Hyg. 2021 Sep 7;105(5):1222-1226. doi: 10.4269/ajtmh.20-1387. PMID: 34491224; PMCID: PMC8592186. Link <https://pubmed.ncbi.nlm.nih.gov/34491224/>
64. Mohammadi F, Badri M, Safari S, Hemmat N. A case report of rhino-facial mucormycosis in a non-diabetic patient with COVID-19: a systematic review of literature and current update. BMC Infect Dis. 2021 Sep 3;21(1):906. doi: 10.1186/s12879-021-06625-3. PMID: 34479495; PMCID: PMC8415695. Link <https://pubmed.ncbi.nlm.nih.gov/34479495/>
65. Whiteside W. Cutaneous Mucormycosis After Elective Outpatient Gastrocnemius Recession for Plantar Fasciitis in an Immunocompetent Host: A Case Report. JBJS Case Connect. 2021 Sep 2;11(3). doi: 10.2106/JBJS.CC.2100358. PMID: 34473677. Link <https://pubmed.ncbi.nlm.nih.gov/34473677/>
66. Guimarães JA, Moura FC. Refractory rhino-orbito-cerebral mucormycosis treated with intraconal amphotericin B. Arq Bras Oftalmol. 2022 Jan-Feb;85(1):77-81. doi: 10.5935/0004-2749.20220009. PMID: 34468552. Link <https://pubmed.ncbi.nlm.nih.gov/34468552/>
67. Garg M, Prabhakar N, Muthu V, Farookh S, Kaur H, Suri V, Agarwal R. CT Findings of COVID-19-associated Pulmonary Mucormycosis: A Case Series and Literature Review. Radiology. 2022 Jan;302(1):214-217. doi: 10.1148/radiol.2021211583. Epub 2021 Aug 31. PMID: 34463553; PMCID: PMC8717687. Link <https://pubmed.ncbi.nlm.nih.gov/34463553/>
68. Dilek A, Ozaras R, Ozkaya S, Sunbul M, Sen EI, Leblebicioglu H. COVID-19-associated mucormycosis: Case report and systematic review. Travel Med Infect Dis. 2021 Nov-Dec;44:102148. doi: 10.1016/j.tmaid.2021.102148. Epub 2021 Aug 26. PMID: 34454090; PMCID: PMC8387131. Link <https://pubmed.ncbi.nlm.nih.gov/34454090/>
69. Sahu ES, Sahu A, Ghodgaonkar P, Lahoti K, Bhargava A. COVID-19-associated rhino-orbital- cerebral mixed mycoses with intracranial fungal granuloma - An aggressively managed rare entity. Indian J Ophthalmol. 2021 Sep;69(9):2537-2539. doi: 10.4103/ijo.IJO_1598_21. PMID: 34427263; PMCID: PMC8544050. Link <https://pubmed.ncbi.nlm.nih.gov/34427263/>
70. Huang H, Xie L, Zheng Z, Yu H, Tu L, Cui C, Yu J. Mucormycosis-induced upper gastrointestinal ulcer perforation in immunocompetent patients: a report of two cases. BMC Gastroenterol. 2021 Aug 3;21(1):311. doi: 10.1186/s12876-021-01881-8. PMID: 34404350; PMCID: PMC8370051. Link <https://pubmed.ncbi.nlm.nih.gov/34404350/>
71. Venugopal A, Marya A. Palatal mucormycosis in a patient with SARS-CoV-2 infection. CMAJ. 2021 Aug 16;193(32):E1254. doi: 10.1503/cmaj.211026. PMID: 34400485; PMCID: PMC8386488. Link <https://pubmed.ncbi.nlm.nih.gov/34400485/>
72. Yu Q, Zhang Q, Yu J, Song Y, Zhang W, Que C. A 66-Year-Old Woman With Progressive Dyspnea and Obstructive Pneumonia. Chest. 2021 Aug;160(2):e177-e180. doi: 10.1016/j.chest.2021.02.078. PMID: 34366040. Link <https://pubmed.ncbi.nlm.nih.gov/34366040/>
73. Chauhan K, Soni D, Sarkar D, Karuna T, Sharma B, Singh S, Karkhur S. Mucormycosis after COVID-19 in a patient with diabetes. Lancet. 2021 Aug 21;398(10301):e10. doi: 10.1016/S0140-6736(21)01641-X. Epub 2021 Aug 4. PMID: 34363754; PMCID: PMC8336972. <https://pubmed.ncbi.nlm.nih.gov/34363754/>
74. Prado-Calleros HM, Brito-Vera JP, Moreno-Coutiño G, Andrade-Morelos LE, Escobedo-Torres MP, Vázquez-Zavala G, Hernández-Castro R. Rhino-Orbital mucormycosis in an immunocompetent pediatric patient with hyperglycemia of the hospitalized patient. J Infect Dev Ctries. 2021 Jul 31;15(7):1035-1038. doi: 10.3855/jidc.14299. PMID: 34343131. Link <https://pubmed.ncbi.nlm.nih.gov/34343131/>
75. Ostovan VR, Rezapanah S, Behzadi Z, Hosseini L, Jahangiri R, Anbardar MH, Rostami M. Coronavirus disease (COVID-19) complicated by rhino-orbital-cerebral mucormycosis presenting with neurovascular thrombosis: a case report and review of literature. J Neurovirol. 2021 Aug;27(4):644-649. doi: 10.1007/s13365-021-00996-8. Epub 2021 Aug 3. PMID: 34342852; PMCID: PMC8330178. Link <https://pubmed.ncbi.nlm.nih.gov/34342852/>
76. Shakir M, Maan MHA, Waheed S. Mucormycosis in a patient with COVID-19 with uncontrolled diabetes. BMJ Case Rep. 2021 Jul 29;14(7):e245343. doi: 10.1136/bcr-2021-245343. PMID: 34326123; PMCID: PMC8323390. Link <https://pubmed.ncbi.nlm.nih.gov/34326123/>
77. Manda D, Sen I, Thakral P, Das SS, Cb V, Malik D. Invasive Fungal Infection in COVID-19-Recovered Patient Detected on 18F-FDG-Labeled Leukocytes PET/CT Scan. Clin Nucl Med. 2022 Feb 1;47(2):e177-e179. doi: 10.1097/RLU.0000000000003852. PMID: 34319959; PMCID: PMC8745955. Link <https://pubmed.ncbi.nlm.nih.gov/34319959/>
78. Alfishawy M, Elbendary A, Younes A, Negm A, Hassan WS, Osman SH, Nassar M, Elanany MG. Diabetes mellitus and Coronavirus Disease (Covid-19) Associated Mucormycosis (CAM): A wake-up call from Egypt. Diabetes Metab Syndr. 2021 Sep-Oct;15(5):102195. doi: 10.1016/j.dsx.2021.102195. Epub 2021 Jul 8. PMID: 34314921; PMCID: PMC8264523 Link <https://pubmed.ncbi.nlm.nih.gov/34314921/>
79. Randhawa G, Hagaman S, Pourabdollah Tootkaboni M, Kundal SV, Oli S, Alrassi J, Schild SD, Abu-Ghanem S, Brejt S. A Rare Case of Invasive Mucormycosis in a Diabetic Patient Treated with a Short Course of Dexamethasone. Am J Case Rep. 2021 Jul 26;22:e932129. doi: 10.12659/AJCR.932129. PMID: 34310559; PMCID: PMC8323743. Link <https://pubmed.ncbi.nlm.nih.gov/34310559/>
80. Hammami F, Koubaa M, Chakroun A, Smaoui F, Marrakchi C, Hentati N, Mzali R, Rekik K, Jemaa MB. Survival of an immuno-competent patient from splenic and gastric mucormycosis-case report and review of the literature. J Mycol Med. 2021 Dec;31(4):101174. doi: 10.1016/j.mycmed.2021.101174. Epub 2021 Jul 5. PMID: 34274682. Link <https://pubmed.ncbi.nlm.nih.gov/34274682/>
81. Hattori S, Matono T, Hirakawa M, Nakamata Y, Okamura K, Hamashoji T, Kometani T, Nakashima T, Sasaki S, Minagawa R, Kajiyama K. Critical peritonitis secondary to gastrointestinal mucormycosis in a peritoneal dialysis patient: a case report. CEN Case Rep. 2022 Feb;11(1):31-35. doi: 10.1007/s13730-021-00628-4. Epub 2021 Jul 17. PMID: 34273082; PMCID: PMC8811074. Link <https://pubmed.ncbi.nlm.nih.gov/34273082/>
82. Tabarsi P, Khalili N, Pourabdollah M, Sharifynia S, Safavi Naeini A, Ghorbani J, Mohamadnia A, Abtahian Z, Askari E. Case Report: COVID-19-associated Rhinosinusitis Mucormycosis Caused by Rhizopus arrhizus: A Rare but Potentially Fatal Infection Occurring After Treatment with Corticosteroids. Am J Trop Med Hyg. 2021 Jul 8;105(2):449-453. doi: 10.4269/ajtmh.21-0359. PMID: 34237015; PMCID: PMC8437195. Link <https://pubmed.ncbi.nlm.nih.gov/34237015/>
83. Hu ZM, Wang LL, Zou L, Chen ZJ, Yi Y, Meng QB, Feng Y. Coinfection pulmonary mucormycosis and aspergillosis with disseminated mucormycosis involving gastrointestinalin in an acute B-lymphoblastic leukemia patient. Braz J Microbiol. 2021 Dec;52(4):2063-2068. doi: 10.1007/s42770-021-00554-8. Epub 2021 Jul 3. PMID: 34218427; PMCID: PMC8254622. Link <https://pubmed.ncbi.nlm.nih.gov/34218427/>
84. Singh RP, Gupta N, Kaur T, Gupta A. Rare case of gastrointestinal mucormycosis with colonic perforation in an immunocompetent patient with COVID-19. BMJ Case Rep. 2021 Jul 2;14(7):e244096. doi: 10.1136/bcr-2021-244096. PMID: 34215642; PMCID: PMC8256735. Link <https://pubmed.ncbi.nlm.nih.gov/34215642/>
85. Chiang TH, Lee YW, Tan JH, Kao CC, Chang CC, Fang KC. Mucormycosis causing massive lower gastrointestinal bleeding: a case report. BMC Gastroenterol. 2021 Jul 2;21(1):272. doi: 10.1186/s12876-021-01846-x. PMID: 34215188; PMCID: PMC8252205. Link <https://pubmed.ncbi.nlm.nih.gov/34215188/>
86. Garg P, Mittal S. Mass urine sugar testing to tackle post-COVID mucormycosis epidemic in India-MUSTARD concept. Diabetes Metab Syndr. 2021 Jul-Aug;15(4):102177. doi: 10.1016/j.dsx.2021.06.014. Epub 2021 Jun 18. PMID: 34198108. Link <https://pubmed.ncbi.nlm.nih.gov/34198108/>
87. Martin-Blais R, Pathak S, Fitzwater S, Dawson DW, Sisk AE, Farmer DG, Venick R, Yeganeh N. Intestinal mucormycosis initially identified by next-generation sequencing of cell-free DNA. Transpl Infect Dis. 2021 Aug;23(4):e13656. doi: 10.1111/tid.13656. Epub 2021 Jun 27. PMID: 34176206. Link <https://pubmed.ncbi.nlm.nih.gov/34176206/>
88. Baskar HC, Chandran A, Reddy CS, Singh S. Rhino-orbital mucormycosis in a COVID-19 patient. BMJ Case Rep. 2021 Jun 24;14(6):e244232. doi: 10.1136/bcr-2021-244232. PMID: 34167998; PMCID: PMC8230977. Link <https://pubmed.ncbi.nlm.nih.gov/34167998/>
89. Kachuei R, Badali H, Vaezi A, Jafari NJ, Ahmadikia K, Kord M, Aala F, Al-Hatmi AM, Khodavaisy S. Fatal necrotising cutaneous mucormycosis due to novel Saksenaea species: a case study. J Wound Care. 2021 Jun 2;30(6):465-468. doi: 10.12968/jowc.2021.30.6.465. PMID: 34121440. Link <https://pubmed.ncbi.nlm.nih.gov/34121440/>
90. Nehara HR, Puri I, Singhal V, Ih S, Bishnoi BR, Sirohi P. Rhinocerebral mucormycosis in COVID-19 patient with diabetes a deadly trio: Case series from the north-western part of India. Indian J Med Microbiol. 2021 Jul;39(3):380-383. doi: 10.1016/j.ijmmb.2021.05.009. Epub 2021 May 26. PMID: 34052046; PMCID: PMC8153224. Link <https://pubmed.ncbi.nlm.nih.gov/34052046/>
91. Arana C, Cuevas Ramírez RE, Xipell M, Casals J, Moreno A, Herrera S, Bodro M, Cofan F, Diekmann F, Esforzado N. Mucormycosis associated with COVID-19 in two kidney transplant patients. Transpl Infect Dis. 2021 Aug;23(4):e13652. doi: 10.1111/tid.13652. Epub 2021 Jun 13. PMID: 34038014; PMCID: PMC8209809. Link <https://pubmed.ncbi.nlm.nih.gov/34038014/>
92. Rao R, Shetty AP, Nagesh CP. Orbital infarction syndrome secondary to rhino-orbital mucormycosis in a case of COVID-19: Clinico-radiological features. Indian J Ophthalmol. 2021 Jun;69(6):1627-1630. doi: 10.4103/ijo.IJO_1053_21. PMID: 34011758; PMCID: PMC8302269. Link <https://pubmed.ncbi.nlm.nih.gov/34011758/>
93. Mejia Buritica L, Karduss Urueta AJ. Pulmonary Mucormycosis. N Engl J Med. 2021 May 6;384(18):e69. doi: 10.1056/NEJMicm2030205. Epub 2021 May 1. PMID: 33951358. Link <https://pubmed.ncbi.nlm.nih.gov/33951358/>
94. Khosravi MH, Zahra K, Hosseini MA, Dadgar S, Ziya D, Saeedi M. Ear and Face Mucormycosis; A Case Report. Int Tinnitus J. 2021 Mar 1;25(1):7-9. doi: 10.5935/0946-5448.2021002. PMID: 33944528. Link <https://pubmed.ncbi.nlm.nih.gov/33944528/>
95. Himaal Dev GJ, Venkategowda PM, Sutar AR, Shankar V. Intestinal mucormycosis in an adult with H1N1 pneumonia on extracorporeal membrane oxygenation. Ann Card Anaesth. 2021 Jan-Mar;24(1):92-94. doi: 10.4103/aca.ACA_1_20. PMID: 33938842; PMCID: PMC8081148. Link <https://pubmed.ncbi.nlm.nih.gov/33938842/>
96. Revannavar SM, P S S, Samaga L, V K V. COVID-19 triggering mucormycosis in a susceptible patient: a new phenomenon in the developing world? BMJ Case Rep. 2021 Apr 27;14(4):e241663. doi: 10.1136/bcr-2021-241663. PMID: 33906877; PMCID: PMC8088249. Link <https://pubmed.ncbi.nlm.nih.gov/33906877/>
97. Fujisaki T, Inagaki J, Kouroki M, Honda Y, Matsuishi T, Kamizono J, Yasui M. Pulmonary Actinomycosis and Mucormycosis Coinfection in a Patient With Philadelphia Chromosome-positive Acute Lymphoblastic Leukemia Undergoing Chemotherapy. J Pediatr Hematol Oncol. 2022 Mar 1;44(2):e529-e531. doi: 10.1097/MPH.0000000000002181. PMID: 33902064. Link <https://pubmed.ncbi.nlm.nih.gov/33902064/>
98. Wang J, Li Y, Luo S, Zheng H. Rhinocerebral mucormycosis secondary to severe acute pancreatitis and diabetic ketoacidosis: a case report. Diagn Pathol. 2021 Apr 21;16(1):34. doi: 10.1186/s13000-021-01094-3. PMID: 33882979; PMCID: PMC8061203. Link <https://pubmed.ncbi.nlm.nih.gov/33882979/>
99. Khatri A, Chang KM, Berlinrut I, Wallach F. Mucormycosis after Coronavirus disease 2019 infection in a heart transplant recipient - Case report and review of literature. J Mycol Med. 2021 Jun;31(2):101125. doi: 10.1016/j.mycmed.2021.101125. Epub 2021 Apr 2. PMID: 33857916; PMCID: PMC8017948 Link <https://pubmed.ncbi.nlm.nih.gov/33857916/>
100. Veisi A, Bagheri A, Eshaghi M, Rikhtehgar MH, Rezaei Kanavi M, Farjad R. Rhino-orbital mucormycosis during steroid therapy in COVID-19 patients: A case report. Eur J Ophthalmol. 2022 Jul;32(4):NP11-NP16. doi: 10.1177/11206721211009450. Epub 2021 Apr 10. PMID: 33843287; PMCID: PMC9294610. Link <https://pubmed.ncbi.nlm.nih.gov/33843287/>
101. Ghaemi N, Bagheri S, Shirdelzade S. Pulmonary and cutaneous mucormycosis in two children with diabetes mellitus type 1. J Pediatr Endocrinol Metab. 2021 Apr 6;34(7):941-945. doi: 10.1515/jpem-2020-0623. PMID: 33819417. Link https://pubmed.ncbi.nlm.nih.gov/33819417/
102. Valente Aguiar P, Carvalho B, Monteiro P, Linhares P, Camacho Ó, Vaz R. Hyperbaric oxygen treatment: Results in seven patients with severe bacterial postoperative central nervous system infections and refractory mucormycosis. Diving Hyperb Med. 2021 Mar 31;51(1):86-93. doi: 10.28920/dhm51.1.86-93. PMID: 33761547; PMCID: PMC8313776. Link <https://pubmed.ncbi.nlm.nih.gov/33761547/>
103. Karimi-Galougahi M, Arastou S, Haseli S. Fulminant mucormycosis complicating coronavirus disease 2019 (COVID-19). Int Forum Allergy Rhinol. 2021 Jun;11(6):1029-1030. doi: 10.1002/alr.22785. Epub 2021 Mar 13. PMID: 33713565; PMCID: PMC8250489. Link <https://pubmed.ncbi.nlm.nih.gov/33713565/>
104. Schneider M, Kobayashi K, Uldry E, Demartines N, Golshayan D, Halkic N. Rhizomucor hepatosplenic abscesses in a patient with renal and pancreatic transplantation. Ann R Coll Surg Engl. 2021 Apr;103(4):e131-e135. doi: 10.1308/rcsann.2020.7125. Epub 2021 Mar 8. PMID: 33682478. Link <https://pubmed.ncbi.nlm.nih.gov/33682478/>
105. Maeda Y, Toda K, Toi S, Yano T, Iijima M, Shimizu Y, Nagashima Y, Itakura Y, Iguchi S, Kikuchi K, Shibuya K, Nonaka M, Kitagawa K. Diagnostic Utility of Polymerase Chain Reaction for Paraffin-embedded Sinus Specimens for Rhinocerebral Mucormycosis Complicated by Internal Carotid Artery Thrombosis and Cerebral Infarction. Intern Med. 2021 Aug 15;60(16):2683-2686. doi: 10.2169/internalmedicine.6809-20. Epub 2021 Mar 8. PMID: 33678746; PMCID: PMC8429304. Link <https://pubmed.ncbi.nlm.nih.gov/33678746/>
106. Coomes DS, Logan RL, Backous CA, Adeyemi OA. A 51-Year-Old Woman With a Mediastinal Mass. Chest. 2021 Mar;159(3):e141-e145. doi: 10.1016/j.chest.2020.09.261. PMID: 33678281. <https://pubmed.ncbi.nlm.nih.gov/33678281/>
107. Baldwin XL, Serrano Rodriguez P, Nickeleit V, Toledo A. Graft Nephrectomy as Rescue Therapy for Posttransplant Rhizopus Pyelonephritis in a Pediatric Patient. Exp Clin Transplant. 2021 May;19(5):489-492. doi: 10.6002/ect.2020.0356. Epub 2021 Feb 19. PMID: 33605205. Link <https://pubmed.ncbi.nlm.nih.gov/33605205/>
108. Ahmadikia K, Hashemi SJ, Khodavaisy S, Getso MI, Alijani N, Badali H, Mirhendi H, Salehi M, Tabari A, Mohammadi Ardehali M, Kord M, Roilides E, Rezaie S. The double-edged sword of systemic corticosteroid therapy in viral pneumonia: A case report and comparative review of influenza-associated mucormycosis versus COVID-19 associated mucormycosis. Mycoses. 2021 Aug;64(8):798-808. doi: 10.1111/myc.13256. Epub 2021 Mar 5. PMID: 33590551; PMCID: PMC8013756. Link <https://pubmed.ncbi.nlm.nih.gov/33590551/>
109. Beiglboeck FM, Theofilou NE, Fuchs MD, Wiesli MG, Leiggener C, Igelbrink S, Augello M. Managing mucormycosis in diabetic patients: A case report with critical review of the literature. Oral Dis. 2022 Apr;28(3):568-576. doi: 10.1111/odi.13802. Epub 2021 Mar 2. PMID: 33583133. Link <https://pubmed.ncbi.nlm.nih.gov/33583133/>
110. Park YL, Cho S, Kim JW. Mucormycosis originated total maxillary and cranial base osteonecrosis: a possible misdiagnosis to malignancy. BMC Oral Health. 2021 Feb 12;21(1):65. doi: 10.1186/s12903-021-01411-8. PMID: 33579255; PMCID: PMC7881650. Link <https://pubmed.ncbi.nlm.nih.gov/33579255/>
111. Liang Y, Chen X, Wang J, Guo C, Liu F, Yang J. Oral posaconazole and bronchoscopy as a treatment for pulmonary mucormycosis in pediatric acute lymphoblastic leukemia patient: A case report. Medicine (Baltimore). 2021 Feb 12;100(6):e24630. doi: 10.1097/MD.0000000000024630. PMID: 33578579; PMCID: PMC7886464. Link <https://pubmed.ncbi.nlm.nih.gov/33578579/>
112. Deb A, Pathak P, Sreedharan S, Rao ACK, Patil V. A rare case of parotid mucormycosis diagnosed on aspiration cytology. Diagn Cytopathol. 2021 Apr;49(4):552-554. doi: 10.1002/dc.24719. Epub 2021 Feb 7. PMID: 33554465. Link <https://pubmed.ncbi.nlm.nih.gov/33554465/>
113. Garg D, Muthu V, Sehgal IS, Ramachandran R, Kaur H, Bhalla A, Puri GD, Chakrabarti A, Agarwal R. Coronavirus Disease (Covid-19) Associated Mucormycosis (CAM): Case Report and Systematic Review of Literature. Mycopathologia. 2021 May;186(2):289-298. doi: 10.1007/s11046-021-00528-2. Epub 2021 Feb 5. PMID: 33544266; PMCID: PMC7862973. Link <https://pubmed.ncbi.nlm.nih.gov/33544266/>
114. Martínez-Herrera E, Julián-Castrejón A, Frías-De-León MG, Moreno-Coutiño G. Rhinocerebral mucormycosis to the rise? The impact of the worldwide diabetes epidemic. An Bras Dermatol. 2021 Mar-Apr;96(2):196-199. doi: 10.1016/j.abd.2020.06.008. Epub 2021 Jan 23. PMID: 33531184; PMCID: PMC8007488. Link <https://pubmed.ncbi.nlm.nih.gov/33531184/>
115. Bellanger AP, Navellou JC, Lepiller Q, Brion A, Brunel AS, Millon L, Berceanu A. Mixed mold infection with Aspergillus fumigatus and Rhizopus microsporus in a severe acute respiratory syndrome Coronavirus 2 (SARS-CoV-2) patient. Infect Dis Now. 2021 Oct;51(7):633-635. doi: 10.1016/j.idnow.2021.01.010. Epub 2021 Jan 27. PMID: 33527098; PMCID: PMC7839422. Link <https://pubmed.ncbi.nlm.nih.gov/33527098/>
116. Redmann AJ, Myer CM 4th, Khandelwal P, Danzinger-Isakov L. Invasive fungal pharyngitis in a pediatric bone marrow transplant patient. Pediatr Transplant. 2021 Aug;25(5):e13853. doi: 10.1111/petr.13853. Epub 2021 Jan 23. PMID: 33484226. Link <https://pubmed.ncbi.nlm.nih.gov/33484226/>
117. Hall EM, Yin DE, Goyal RK, Ahmed AA, Mitchell GS, St Peter SD, Flatt TG, Ahmed IA, Li W, Hendrickson RJ, August KJ, Myers GD. Tisagenlecleucel infusion in patients with relapsed/refractory ALL and concurrent serious infection. J Immunother Cancer. 2021 Jan;9(1):e001225. doi: 10.1136/jitc-2020-001225. Erratum in: J Immunother Cancer. 2021 Oct;9(10): PMID: 33472856; PMCID: PMC7818837. Link <https://pubmed.ncbi.nlm.nih.gov/33472856/>
118. Garrido PM, Pimenta R, Viana I, Kutzner H, Filipe P, Soares-Almeida L. Cutaneous mucormycosis mimicking pancreatic panniculitis. J Cutan Pathol. 2021 Aug;48(8):1007-1009. doi: 10.1111/cup.13763. Epub 2021 Jan 20. PMID: 33470453. Link <https://pubmed.ncbi.nlm.nih.gov/33470453/>
119. Panchanatheeswaran K, Ram D, Prasad S, Srinivas BH, Rath D, SaiChandran BV, Munuswamy H. Thoracic mucormycosis in immunocompetent patients. J Card Surg. 2021 Apr;36(4):1183-1188. doi: 10.1111/jocs.15332. Epub 2021 Jan 19. PMID: 33470008. Link <https://pubmed.ncbi.nlm.nih.gov/33470008/>
120. Chaulk AL, Do TH, Supsupin EP, Bhattacharjee MB, Richani K, Adesina OO. A Unique Radiologic Case of Optic Nerve Infarction in a Patient With Mucormycosis. J Neuroophthalmol. 2021 Sep 1;41(3):e354-e356. doi: 10.1097/WNO.0000000000001179. PMID: 33449489. Link <https://pubmed.ncbi.nlm.nih.gov/33449489/>
121. Dadhich A, Nilesh K, Patil R, Saluja H. Unusual presentation of mucormycosis mimicking a localised sino-orbital pathology. BMJ Case Rep. 2021 Jan 11;14(1):e239199. doi: 10.1136/bcr-2020-239199. PMID: 33431470; PMCID: PMC7802651. Link <https://pubmed.ncbi.nlm.nih.gov/33431470/>
122. Jawanda MK, Narula R, Gupta S, Sharma V, Gupta P, Kaur M. Dual Fungal Infections (Aspergillosis and Mucormycosis) in a Diabetic Mellitus Patient Leading to Maxillary Sinusitis as a Post-COVID Manifestation: First Case Report. Acta Medica (Hradec Kralove). 2021;64(4):227-231. doi: 10.14712/18059694.2022.7. PMID: 35285446. Link <https://pubmed.ncbi.nlm.nih.gov/35285446/>
123. Jawanda MK, Narula R, Gupta S, Sharma V, Sidhu SK, Kaur N. Mixed Infections (Mucormycosis, Actinomycosis and Candidiasis) Leading to Maxillary Osteomyelitis in a Diabetic Mellitus Patient in Post COVID Phase: First Case Report. Acta Medica (Hradec Kralove). 2021;64(4):218-223. doi: 10.14712/18059694.2022.5. PMID: 35285444. Link <https://pubmed.ncbi.nlm.nih.gov/35285444/>
124. Jain A, Knoll B, Lim S, Kleinman G, Epelbaum O. For Whom the Atoll Tolls: Targeting the Reversed Halo Sign. Am J Med. 2021 Mar;134(3):e150-e152. doi: 10.1016/j.amjmed.2020.09.022. Epub 2020 Oct 25. PMID: 33353726. Link <https://pubmed.ncbi.nlm.nih.gov/33353726/>
125. Pasero D, Sanna S, Liperi C, Piredda D, Branca GP, Casadio L, Simeo R, Buselli A, Rizzo D, Bussu F, Rubino S, Terragni P. A challenging complication following SARS-CoV-2 infection: a case of pulmonary mucormycosis. Infection. 2021 Oct;49(5):1055-1060. doi: 10.1007/s15010-020-01561-x. Epub 2020 Dec 17. PMID: 33331988; PMCID: PMC7745708. Link <https://pubmed.ncbi.nlm.nih.gov/33331988/>
126. Thatipelli S, Santoiemma P, Echenique IA, Green R, Ison MG, Ladner D, Kanwar YS, Stosor V. Donor-derived renal allograft mucormycosis in a combined liver and kidney transplantation: Case report and review of the literature. Transpl Infect Dis. 2021 Jun;23(3):e13534. doi: 10.1111/tid.13534. Epub 2020 Dec 14. PMID: 33251715. Link <https://pubmed.ncbi.nlm.nih.gov/33251715/>
127. Shao W, Zhang Z, Feng H, Liang C, Liu D. Pulmonary mucormycosis: a case of pulmonary arterial hypertension, Westermark sign, and bronchopleural fistula. J Int Med Res. 2020 Nov;48(11):300060520971450. doi: 10.1177/0300060520971450. PMID: 33249953; PMCID: PMC7708708. Link <https://pubmed.ncbi.nlm.nih.gov/33249953/>
128. Hunt E. Recognizing mucormycosis in an immunocompromised patient. JAAPA. 2020 Dec;33(12):23-25. doi: 10.1097/01.JAA.0000721656.13462.67. PMID: 33234891. Link <https://pubmed.ncbi.nlm.nih.gov/33234891/>
129. Mekonnen ZK, Ashraf DC, Jankowski T, Grob SR, Vagefi MR, Kersten RC, Simko JP, Winn BJ. Acute Invasive Rhino-Orbital Mucormycosis in a Patient With COVID-19-Associated Acute Respiratory Distress Syndrome. Ophthalmic Plast Reconstr Surg. 2021 Mar-Apr 01;37(2):e40-e80. doi: 10.1097/IOP.0000000000001889. PMID: 33229953; PMCID: PMC7927902. Link <https://pubmed.ncbi.nlm.nih.gov/33229953/>
130. Yinadsawaphan T, Ngamskulrungroj P, Chalermwai W, Dhitinanmuang W, Angkasekwinai N. Gastrointestinal mucormycosis due to Rhizopus microsporus following Streptococcus pyogenes toxic shock syndrome in an HIV patient: a case report. BMC Infect Dis. 2020 Nov 10;20(1):817. doi: 10.1186/s12879-020-05548-9. PMID: 33167878; PMCID: PMC7654013. Link <https://pubmed.ncbi.nlm.nih.gov/33167878/>
131. V Ramesh DNS, Anjum G, Rukmangada T, Patil N. Rhinocerebral maxillary mucormycosis: A palatal ulcer. Indian J Dent Res. 2020 Jul-Aug;31(4):652-655. doi: 10.4103/ijdr.IJDR_234_18. PMID: 33107473. Link <https://pubmed.ncbi.nlm.nih.gov/33107473/>
132. Ringer M, Pischel L, Azar MM. Diagnosis of oesophageal mucormycosis managed with medical therapy alone. BMJ Case Rep. 2020 Oct 22;13(10):e236869. doi: 10.1136/bcr-2020-236869. PMID: 33093060; PMCID: PMC7583061. Link <https://pubmed.ncbi.nlm.nih.gov/33093060/>
133. Amanati A, Barzegar H, Pouladfar G, Sanaei Dashti A, Abtahi MB, Khademi B, Ashraf MJ, Badiee P, Hamzavi SS, Kashkooe A. Orbital mucormycosis in immunocompetent children; review of risk factors, diagnosis, and treatment approach. BMC Infect Dis. 2020 Oct 19;20(1):770. doi: 10.1186/s12879-020-05460-2. PMID: 33076815; PMCID: PMC7574198. Link <https://pubmed.ncbi.nlm.nih.gov/33076815/>
134. Busbait S, AlMusa Z, Al Duhileb M, Algarni AA, Balhareth A. A Cecal Mucormycosis Mass Mimicking Colon Cancer in a Patient with Renal Transplant: A Case Report and Literature Review. Am J Case Rep. 2020 Oct 19;21:e926325. doi: 10.12659/AJCR.926325. PMID: 33071279; PMCID: PMC7585455. Link <https://pubmed.ncbi.nlm.nih.gov/33071279/>
135. Durà-Miralles X, Escolà-Vergé L, Moreno D, Martínez-Ricarte F, Lung M, Fernández-Hidalgo N, Auger C, Martín-Gómez MT. Isolated cerebral mucormycosis associated with intravenous drug use. J Mycol Med. 2020 Dec;30(4):101046. doi: 10.1016/j.mycmed.2020.101046. Epub 2020 Oct 6. PMID: 33067115. Link <https://pubmed.ncbi.nlm.nih.gov/33067115/>
136. Davidson N, Campbell K, Foroughi F, Tayal V, Lynar S, Crawford LC, Kidd SE, Baird R, Davies J, Meumann EM. Disseminated Saksenaea infection in an immunocompromised host associated with a good clinical outcome: a case report and review of the literature. BMC Infect Dis. 2020 Oct 14;20(1):755. doi: 10.1186/s12879-020-05459-9. PMID: 33054720; PMCID: PMC7559758. Link <https://pubmed.ncbi.nlm.nih.gov/33054720/>
137. Chavan R, Menon A, Soman R, Rodrigues C, Shetty A, Bhadiyadra R, Agashe VM. Fungal osteomyelitis and soft tissue infections: Simple solutions to uncommon scenarios. J Infect Dev Ctries. 2020 Sep 30;14(9):1033-1039. doi: 10.3855/jidc.11449. PMID: 33031093. Link <https://pubmed.ncbi.nlm.nih.gov/33031093/>
138. Werthman-Ehrenreich A. Mucormycosis with orbital compartment syndrome in a patient with COVID-19. Am J Emerg Med. 2021 Apr;42:264.e5-264.e8. doi: 10.1016/j.ajem.2020.09.032. Epub 2020 Sep 16. PMID: 32972795; PMCID: PMC7493738. Link <https://pubmed.ncbi.nlm.nih.gov/32972795/>
139. Arora K, Mehta A, Virk RS, Saini M. Cerebrospinal fluid leak from lateral orbit during exenteration for mucormycosis. BMJ Case Rep. 2020 Sep 13;13(9):e237575. doi: 10.1136/bcr-2020-237575. PMID: 32928823; PMCID: PMC7488801. Link <https://pubmed.ncbi.nlm.nih.gov/32928823/>
140. Musharbash M, Zheng L, Guggina L. Extensive purpura and necrosis of the leg. Cutis. 2020 Jul;106(1):E1-E2. doi: 10.12788/cutis.0041. PMID: 32915940. Link <https://pubmed.ncbi.nlm.nih.gov/32915940/>
141. Papan C, Langer F, Bittenbring JT, Schäfers HJ, Bohle RM, Fries P, Becker SL. A 71-Year-Old Man With Chest Pain and a Solitary Pulmonary Mass. Chest. 2020 Sep;158(3):e123-e126. doi: 10.1016/j.chest.2020.02.083. PMID: 32892888. Link <https://pubmed.ncbi.nlm.nih.gov/32892888/>
142. Cibulas MA, Carrillo EH, Hranjec T, Kiffin CD, Lee SK, Pigneri DA, Sanchez RE, Spitzer RD, Toro TZ, Rosenthal AA. Overwhelming Mucormycosis Abdominal Gunshot Wound Infection in an Immunocompetent Host. Am Surg. 2021 Jan;87(1):140-141. doi: 10.1177/0003134820945220. Epub 2020 Aug 31. PMID: 32866074. Link <https://pubmed.ncbi.nlm.nih.gov/32866074/>
143. Wang X, Ding H, Chen Z, Zeng X, Sun J, Chen H, Fu M. CARD9 Deficiency in a Chinese Man with Cutaneous Mucormycosis, Recurrent Deep Dermatophytosis and a Review of the Literature. Mycopathologia. 2020 Dec;185(6):1041-1050. doi: 10.1007/s11046-020-00487-0. Epub 2020 Aug 31. PMID: 32865705. Link <https://pubmed.ncbi.nlm.nih.gov/32865705/>
144. Chen AJ, Ediriwickrema LS, Verma R, Vavinskaya V, Shaftel S, Deconde AS, Korn BS, Kikkawa DO, Liu CY. A case of mistaken identity: Saksenaea vasiformis of the orbit. Orbit. 2021 Dec;40(6):521-524. doi: 10.1080/01676830.2020.1814354. Epub 2020 Aug 30. PMID: 32862746. Link <https://pubmed.ncbi.nlm.nih.gov/32862746/>
145. Jevalikar G, Sharma R, Raghunathan V, Luthra M, Dhaliwal MS, Jain V, Mithal A. Intestinal mucormycosis complicated by iliac artery aneurysm and ureteric rupture in a child with new-onset type 1 diabetes mellitus. J Paediatr Child Health. 2021 Jul;57(7):1117-1119. doi: 10.1111/jpc.15126. Epub 2020 Aug 20. PMID: 32815594. Link <https://pubmed.ncbi.nlm.nih.gov/32815594/>
146. Martínez-Herrera E, Frías-De-León MG, Julián-Castrejón A, Cruz-Benítez L, Xicohtencatl-Cortes J, Hernández-Castro R. Rhino-orbital mucormycosis due to Apophysomyces ossiformis in a patient with diabetes mellitus: a case report. BMC Infect Dis. 2020 Aug 18;20(1):614. doi: 10.1186/s12879-020-05337-4. PMID: 32811466; PMCID: PMC7437167. Link <https://pubmed.ncbi.nlm.nih.gov/32811466/>
147. Boumaza X, Lelièvre L, Guenounou S, Borel C, Huynh A, Beziat G, Delavigne K, Guinault D, Garric M, Piel-Julian M, Paricaud K, Moulis G, Astudillo L, Sailler L, Farge D, Pugnet G. Pulmonary mucormycosis following autologous hematopoietic stem cell transplantation for rapidly progressive diffuse cutaneous systemic sclerosis: A case report. Medicine (Baltimore). 2020 Jul 31;99(31):e21431. doi: 10.1097/MD.0000000000021431. PMID: 32756151; PMCID: PMC7402716. Link <https://pubmed.ncbi.nlm.nih.gov/32756151/>
148. Yamamoto K, Mawatari M, Fujiya Y, Kutsuna S, Takeshita N, Hayakawa K, Nakamura M, Takanabe Y, Maruoka Y, Inoue M, Hara T, Nagasaka S, Tayama N, Miyazaki Y, Umeyama T, Ohmagari N. Survival case of rhinocerebral and pulmonary mucormycosis due to Cunninghamella bertholletiae during chemotherapy for acute myeloid leukemia: a case report. Infection. 2021 Feb;49(1):165-170. doi: 10.1007/s15010-020-01491-8. Epub 2020 Jul 27. PMID: 32720129. Link <https://pubmed.ncbi.nlm.nih.gov/32720129/>
149. Hagemann JB, Furitsch M, Wais V, Bunjes D, Walther G, Kurzai O, Essig A. First case of fatal Rhizomucor miehei endocarditis in an immunocompromised patient. Diagn Microbiol Infect Dis. 2020 Oct;98(2):115106. doi: 10.1016/j.diagmicrobio.2020.115106. Epub 2020 Jun 10. PMID: 32629298. Link <https://pubmed.ncbi.nlm.nih.gov/32629298/>
150. Hoshal SG, Timbang M, Harris BN, Darrow MA, Bewley AF. Endotracheal Tube Bridle Associated With Full-Thickness Facial Necrosis and Parotid Gland Mucormycosis. Ear Nose Throat J. 2022 Jan;101(1):NP18-NP20. doi: 10.1177/0145561320936489. Epub 2020 Jul 5. PMID: 32627585. Link <https://pubmed.ncbi.nlm.nih.gov/32627585/>
151. Pérez Fernández A, Rubio Mateos JM, Sánchez Fernández MJ. Gastric ulcer due to mucormycosis in a critical patient. Rev Esp Enferm Dig. 2020 Jul;112(7):576-577. doi: 10.17235/reed.2020.6636/2019. PMID: 32578998. Link <https://pubmed.ncbi.nlm.nih.gov/32578998/>
152. Agrawal U, Savaj P, Davda K, Soman R, Shetty A, Sunavala A. Successful treatment of disseminated granulomatous aspergillosis in an apparently immunocompetent host. Trop Doct. 2020 Oct;50(4):346-349. doi: 10.1177/0049475520934358. Epub 2020 Jun 23. PMID: 32576098. Link <https://pubmed.ncbi.nlm.nih.gov/32576098/>
153. Cataño JC, Ramirez IC. Disseminated Cunninghamella Bertholletiae Infection. Am J Med Sci. 2020 Oct;360(4):e9-e10. doi: 10.1016/j.amjms.2020.05.024. Epub 2020 May 21. PMID: 32571517. Link <https://pubmed.ncbi.nlm.nih.gov/32571517/>
154. Petrochko JM, Abrahamian G, Cigarroa F, Thomas E. Colonic mucormycosis in solid organ transplantation: Case report and review of the literature (colonic mucormycosis after DDLT). Transpl Infect Dis. 2020 Dec;22(6):e13362. doi: 10.1111/tid.13362. Epub 2020 Jul 10. PMID: 32510728. Link <https://pubmed.ncbi.nlm.nih.gov/32510728/>
155. Raju B, Santhanakumar KS, Kesavachandran U. Gastrointestinal involvement of unusual Mucormycete Syncephalastrum racemosum in a diabetic patient with adenocarcinoma: rare case presentation with review of literature. Infection. 2020 Oct;48(5):791-797. doi: 10.1007/s15010-020-01455-y. Epub 2020 Jun 6. PMID: 32506307. Link <https://pubmed.ncbi.nlm.nih.gov/32506307/>
156. Ramesh P, Kaur G, Deepak D, Kumar P. Disseminated pulmonary mucormycosis with concomitant tuberculosis infection in a diabetic patient. Int J Mycobacteriol. 2020 Jan-Mar;9(1):95-97. doi: 10.4103/ijmy.ijmy_186_19. PMID: 32474496. Link <https://pubmed.ncbi.nlm.nih.gov/32474496/>
157. Kubica PA, Rehrauer WM, Sterkel AK. An Unusual Case of Actinomucor elegans: A Challenging Diagnosis. Am J Case Rep. 2020 May 15;21:e921562. doi: 10.12659/AJCR.921562. PMID: 32409629; PMCID: PMC7252847. Link <https://pubmed.ncbi.nlm.nih.gov/32409629/>
158. Armstrong-James D, Koh M, Ostermann M, Cockwell P. Optimal management of acute kidney injury in critically ill patients with invasive fungal infections being treated with liposomal amphotericin B. BMJ Case Rep. 2020 May 12;13(5):e233072. doi: 10.1136/bcr-2019-233072. PMID: 32404321; PMCID: PMC7228453. Link <https://pubmed.ncbi.nlm.nih.gov/32404321/>
159. Makimoto Y, Obuchi T, Iwasaki A. [Pulmonary Mucormycosis with Reversed Halo Sign on Computed Tomography of Patient with Leukemia]. Kyobu Geka. 2020 Feb;73(2):127-130. Japanese. PMID: 32393721. Link <https://pubmed.ncbi.nlm.nih.gov/32393721/>
160. Pan J, Tsui C, Li M, Xiao K, de Hoog GS, Verweij PE, Cao Y, Lu H, Jiang Y. First Case of Rhinocerebral Mucormycosis Caused by Lichtheimia ornata, with a Review of Lichtheimia Infections. Mycopathologia. 2020 Jun;185(3):555-567. doi: 10.1007/s11046-020-00451-y. Epub 2020 May 9. PMID: 32388712. Link <https://pubmed.ncbi.nlm.nih.gov/32388712/>
161. Fujisawa Y, Hara S, Zoshima T, Maekawa N, Inoue D, Sasaki M, Gamou T, Nagata Y, Hayashi K, Takeji A, Ito K, Mizushima I, Fujii H, Kawano M. Fulminant myocarditis and pulmonary cavity lesion induced by disseminated mucormycosis in a chronic hemodialysis patient: Report of an autopsied case. Pathol Int. 2020 Aug;70(8):557-562. doi: 10.1111/pin.12943. Epub 2020 Apr 29. PMID: 32350952. Link <https://pubmed.ncbi.nlm.nih.gov/32350952/>
162. Negi R, Kaushik R, Singh S, Punia RS. Mucor as a cause of surgical site infection. Trop Doct. 2020 Jul;50(3):249-251. doi: 10.1177/0049475520921284. Epub 2020 Apr 29. PMID: 32349607. Link <https://pubmed.ncbi.nlm.nih.gov/32349607/>
163. Thomas L, Tay SY, Howard D, Falhammar H. Mucormycosis in a 40-year-old woman with diabetic ketoacidosis. CMAJ. 2020 Apr 20;192(16):E431-E433. doi: 10.1503/cmaj.191364. PMID: 32312825; PMCID: PMC7207186. Link <https://pubmed.ncbi.nlm.nih.gov/32312825/>
164. Prohaska S, Henn P, Wenz S, Frauenfeld L, Rosenberger P, Haeberle HA. A case report of fatal disseminated fungal sepsis in a patient with ARDS and extracorporeal membrane oxygenation. BMC Anesthesiol. 2020 May 7;20(1):107. doi: 10.1186/s12871-020-01031-9. PMID: 32381041; PMCID: PMC7203726. Link <https://pubmed.ncbi.nlm.nih.gov/32381041/>
165. Berns JS, Rapalino O, Fenves AZ, El Khoury JB, Klepeis VE, Anahtar MN. Case 11-2020: A 37-Year-Old Man with Facial Droop, Dysarthria, and Kidney Failure. N Engl J Med. 2020 Apr 9;382(15):1457-1466. doi: 10.1056/NEJMcpc1916252. PMID: 32268031. Link <https://pubmed.ncbi.nlm.nih.gov/32268031/>
166. Ashkenazi-Hoffnung L, Bilavsky E, Levy I, Grisaru G, Sadot E, Ben-Ami R, Novikov A, Fischer S, Nahum E, Scheuerman O. Isavuconazole As Successful Salvage Therapy for Mucormycosis in Pediatric Patients. Pediatr Infect Dis J. 2020 Aug;39(8):718-724. doi: 10.1097/INF.0000000000002671. PMID: 32251256. Link <https://pubmed.ncbi.nlm.nih.gov/32251256/>
167. Konigsberg MW, Wu CH, Strauch RJ. Topical Treatment for Cutaneous Mucormycosis of the Upper Extremity. J Hand Surg Am. 2020 Dec;45(12):1189.e1-1189.e5. doi: 10.1016/j.jhsa.2020.01.015. Epub 2020 Mar 23. PMID: 32216989. Link <https://pubmed.ncbi.nlm.nih.gov/32216989/>
168. Bardwell J, Youseffi B, Marquez J, Zangeneh TT, Al-Obaidi M. Pulmonary Mucormycosis in a Heart Transplant Patient. Am J Med. 2020 Sep;133(9):e524-e525. doi: 10.1016/j.amjmed.2020.02.020. Epub 2020 Mar 19. PMID: 32199812. Link <https://pubmed.ncbi.nlm.nih.gov/32199812/>
169. Maheshwari S, Patil M, Shendey S. Mucormycosis creeping along the nerves in an immunocompetent individual. J Radiol Case Rep. 2019 Oct 31;13(10):1-10. doi: 10.3941/jrcr.v13i10.3671. PMID: 32184922; PMCID: PMC7060009. Link <https://pubmed.ncbi.nlm.nih.gov/32184922/>
170. Hoffmann C, Guillerm G, Le Pape P, Carausu L, Lavergne RA, Nevez G, Le Gal S. Mucorales DNA detection in serum specimens for early diagnosis of mucormycosis. Diagn Microbiol Infect Dis. 2020 Jun;97(2):115004. doi: 10.1016/j.diagmicrobio.2020.115004. Epub 2020 Jan 29. PMID: 32156450. Link <https://pubmed.ncbi.nlm.nih.gov/32156450/>
171. Boukari M, Zhioua Braham I, Kaouel H, El Amri-Mezghanni K, Ayedi O, Maalej R, Mili Boussen I, Ammous I, Zhioua R. Mucormycose rhino-orbito-cérébrale, une urgence diagnostique et thérapeutique [Rhino-orbito-cerebral mucormycosis, a diagnostic and therapeutic emergency]. J Fr Ophtalmol. 2020 Apr;43(4):e153-e155. French. doi: 10.1016/j.jfo.2019.11.005. Epub 2020 Mar 4. PMID: 32145933. Link <https://pubmed.ncbi.nlm.nih.gov/32145933/>
172. Wotiye AB, Ks P, Ayele BA. Invasive intestinal mucormycosis in a 40-year old immunocompetent patient - a rarely reported clinical phenomenon: a case report. BMC Gastroenterol. 2020 Mar 6;20(1):61. doi: 10.1186/s12876-020-01202-5. PMID: 32143639; PMCID: PMC7060531. Link https://pubmed.ncbi.nlm.nih.gov/32143639/
173. Suo L, Dunn JJ. Closing the Brief Case: Cutaneous Fungal Infection in a Pediatric Patient with Newly Diagnosed Acute Lymphocytic Leukemia. J Clin Microbiol. 2020 Feb 24;58(3):e00788-19. doi: 10.1128/JCM.00788-19. PMID: 32094120; PMCID: PMC7041575. Link <https://pubmed.ncbi.nlm.nih.gov/32094120/>
174. Suo L, Dunn JJ. The Brief Case: Cutaneous Fungal Infection in a Pediatric Patient with Newly Diagnosed Acute Lymphocytic Leukemia. J Clin Microbiol. 2020 Feb 24;58(3):e00787-19. doi: 10.1128/JCM.00787-19. PMID: 32094119; PMCID: PMC7041591. Link <https://pubmed.ncbi.nlm.nih.gov/32094119/>
175. Sharma D. Successful management of emphysematous gastritis with invasive gastric mucormycosis. BMJ Case Rep. 2020 Feb 20;13(2):e231297. doi: 10.1136/bcr-2019-231297. PMID: 32086323; PMCID: PMC7046440. Link <https://pubmed.ncbi.nlm.nih.gov/32086323/>
176. Fatemizadeh R, Rodman E, Demmler-Harrison GJ, Dinu D. Rhizopus Infection in a Preterm Infant: A Novel Use of Posaconazole. Pediatr Infect Dis J. 2020 Apr;39(4):310-312. doi: 10.1097/INF.0000000000002554. PMID: 32084112. Link <https://pubmed.ncbi.nlm.nih.gov/32084112/>
177. Geyman LS, Pham CM, Aakalu VK. Acute-Onset Visual Acuity Loss in a Man With Advanced Diabetes Mellitus. JAMA Ophthalmol. 2020 Apr 1;138(4):416-417. doi: 10.1001/jamaophthalmol.2019.5388. PMID: 32077892. Link <https://pubmed.ncbi.nlm.nih.gov/32077892/>
178. Mensa J, Dueñas Gutiérrez C, Cardozo C, Rodríguez Fernández L, Kestler M, Muñoz P, Bouza E. Neck infection after allogenic hematopoietic progenitors transplantation. Rev Esp Quimioter. 2020 Apr;33(2):130-136. doi: 10.37201/req/100.2019. Epub 2020 Feb 14. PMID: 32056419; PMCID: PMC7111238. Link <https://pubmed.ncbi.nlm.nih.gov/32056419/>
179. Perz A, Makar G, Fernandez E, Weinstock J, Rafferty W. Primary cutaneous mucormycosis of the abdomen at the site of repeated insulin injections. BMJ Case Rep. 2020 Feb 10;13(2):e233284. doi: 10.1136/bcr-2019-233284. PMID: 32047088; PMCID: PMC7035857. Link <https://pubmed.ncbi.nlm.nih.gov/32047088/>
180. Kim M, Lim JH, Park M, Cha HK, Kim L, Nam HS. A Rare Case of Fatal Endobronchial Mucormycosis Masquerading as Endobronchial Tuberculosis. Medicina (Kaunas). 2020 Feb 6;56(2):64. doi: 10.3390/medicina56020064. PMID: 32041089; PMCID: PMC7074438. Link <https://pubmed.ncbi.nlm.nih.gov/32041089/>
181. Bellanger AP, Rocchi S, Berceanu A, Scherer E, Larosa F, Millon L. Positive quantitative PCR detecting Fusarium solani in a case of mixed invasive fungal disease due to Mucorales and Fusarium solani. Bone Marrow Transplant. 2020 May;55(5):873-876. doi: 10.1038/s41409-020-0819-3. Epub 2020 Feb 5. PMID: 32024993. Link <https://pubmed.ncbi.nlm.nih.gov/32024993/>
182. Sakai T, Sato K, Kikuchi T, Obata M, Konuma Y. A Radical Approach to Acute Lymphoblastic Leukemia Treatment: A Case Study of a Veterinarian Specializing in Livestock who Developed Disseminated Mucormycosis during Induction Therapy. Intern Med. 2020;59(2):261-266. doi: 10.2169/internalmedicine.3314-19. Epub 2020 Jan 15. PMID: 31941872; PMCID: PMC7008056. Link <https://pubmed.ncbi.nlm.nih.gov/31941872/>
183. Poyuran R, Dharan BS, Sandhyamani S, Narasimhaiah D. Mucormycosis-induced ileocecal perforation: A case report and review of literature. J Postgrad Med. 2020 Jan-Mar;66(1):48-50. doi: 10.4103/jpgm.JPGM_420_19. PMID: 31929312; PMCID: PMC6970318. Link https://pubmed.ncbi.nlm.nih.gov/31929312/
184. Srinivas R, Jacob TJK, Raj PM, Korula S, Mathew LG. Paediatric mucormycosis: tailoring surgical strategies to compliment antifungal chemotherapy. Different strokes for different folks. Trop Doct. 2020 Jan;50(1):87-90. doi: 10.1177/0049475519874270. PMID: 31928201. Link <https://pubmed.ncbi.nlm.nih.gov/31928201/>
185. Khafagy R, Gupta S, Campisi P, Waters V. Treatment of localized mucormycosis using nasal amphotericin B irrigation in pediatric oncology. Pediatr Blood Cancer. 2020 Apr;67(4):e28175. doi: 10.1002/pbc.28175. Epub 2020 Jan 11. PMID: 31925929. Link <https://pubmed.ncbi.nlm.nih.gov/31925929/>
186. Buckholz A, Kaplan A. Gastrointestinal Mucormycosis Presenting as Emphysematous Gastritis After Stem Cell Transplant for Myeloma. Mayo Clin Proc. 2020 Jan;95(1):33-34. doi: 10.1016/j.mayocp.2019.10.037. PMID: 31902426. Link <https://pubmed.ncbi.nlm.nih.gov/31902426/>
187. Allam SR, Madhrira MM, Memon IA, Tessier J, Johnson JA, Dao A, Rofaiel G. Invasive mucormycosis in a renal transplant recipient. Kidney Int. 2020 Jan;97(1):216. doi: 10.1016/j.kint.2019.08.011. PMID: 31901346. Link <https://pubmed.ncbi.nlm.nih.gov/31901346/>
188. Łanocha AA, Guzicka-Kazimierczak R, Zdziarska B, Wawrzynowicz-Syczewska M. Mucormycosis in a patient with acute myeloblastic leukemia following liver transplantation for Wilson's disease. Ann Agric Environ Med. 2019 Dec 19;26(4):665-668. doi: 10.26444/aaem/105533. Epub 2019 Mar 18. PMID: 31885243. Link <https://pubmed.ncbi.nlm.nih.gov/31885243/>
189. Huang YQ, Tremblay JA, Chapdelaine H, Luong ML, Carrier FM. Pulmonary mucormycosis in a patient with acute liver failure: A case report and systematic review of the literature. J Crit Care. 2020 Apr;56:89-93. doi: 10.1016/j.jcrc.2019.12.012. Epub 2019 Dec 12. PMID: 31881411. Link <https://pubmed.ncbi.nlm.nih.gov/31881411/>
190. Gupta N, Soneja M. Amphotericin-induced pancytopenia in a patient with rhino-orbital mucormycosis. Postgrad Med J. 2020 Sep;96(1139):572. doi: 10.1136/postgradmedj-2019-137378. Epub 2019 Dec 26. PMID: 31879335. <https://pubmed.ncbi.nlm.nih.gov/31879335/>
191. Biddeci G, Antonello M, Pizzi M, Petris MG, Pillon M, Donà D, Biffi A, Putti MC. Mucormycosis with peculiar aortic involvement in a child with acute lymphoblastic leukemia. Pediatr Hematol Oncol. 2020 Mar;37(2):164-169. doi: 10.1080/08880018.2019.1691294. Epub 2019 Dec 17. PMID: 31847684. Link <https://pubmed.ncbi.nlm.nih.gov/31847684/>
192. Haque H, Nettboy S, Kumar S. Surgical-site mucormycosis infection in a solid-organ transplant recipient and a concise review of the literature. BMJ Case Rep. 2019 Dec 10;12(12):e229687. doi: 10.1136/bcr-2019-229687. PMID: 31826901; PMCID: PMC6936439. Link <https://pubmed.ncbi.nlm.nih.gov/31826901/>
193. Haque H, Nettboy S, Kumar S. Surgical-site mucormycosis infection in a solid-organ transplant recipient and a concise review of the literature. BMJ Case Rep. 2019 Dec 10;12(12):e229687. doi: 10.1136/bcr-2019-229687. PMID: 31826901; PMCID: PMC6936439. Link <https://pubmed.ncbi.nlm.nih.gov/31826901/>
194. Saran S, Sharma Y, Khanna T, Singh AP. Maxillary mucormycosis causing oroantral fistula in a young female. Ann Afr Med. 2019 Oct-Dec;18(4):211-213. doi: 10.4103/aam.aam_1_19. PMID: 31823958; PMCID: PMC6918789. Link <https://pubmed.ncbi.nlm.nih.gov/31823958/>
195. Sullivan T, Rana M. The reversed halo sign and mucormycosis. Lancet Infect Dis. 2019 Dec;19(12):1379. doi: 10.1016/S1473-3099(19)30548-1. PMID: 31782402. <https://pubmed.ncbi.nlm.nih.gov/31782402/>
196. Uchida T, Okamoto M, Fujikawa K, Yoshikawa D, Mizokami A, Mihara T, Kondo A, Ohba K, Kurohama K, Nakashima M, Sekine I, Nakamura S, Miyazaki Y, Kawakami A. Gastric mucormycosis complicated by a gastropleural fistula: A case report and review of the literature. Medicine (Baltimore). 2019 Nov;98(48):e18142. doi: 10.1097/MD.0000000000018142. PMID: 31770250; PMCID: PMC6890297. Link <https://pubmed.ncbi.nlm.nih.gov/31770250/>
197. Yi Y, Cho SY, Lee DG, Jung JI, Park YJ, Lee KY. Invasive Pulmonary Aspergillosis Due to Aspergillus awamori: Role of Calcium Oxalate Crystal Precipitation Mimicking Mucormycosis. Mycopathologia. 2020 Apr;185(2):409-411. doi: 10.1007/s11046-019-00405-z. Epub 2019 Nov 18. PMID: 31741128. Link <https://pubmed.ncbi.nlm.nih.gov/31741128/>
198. Harada N, Kimura SI, Gomyo A, Hayakawa J, Tamaki M, Akahoshi Y, Ugai T, Kusuda M, Kameda K, Wada H, Ishihara Y, Kawamura K, Sakamoto K, Sato M, Terasako-Saito K, Kikuchi M, Nakasone H, Kako S, Tsubochi H, Kanda Y. Surgical resection for persistent localized pulmonary fungal infection prior to allogeneic hematopoietic stem cell transplantation: Analysis of six cases. J Infect Chemother. 2020 Feb;26(2):175-180. doi: 10.1016/j.jiac.2019.08.003. Epub 2019 Nov 15. PMID: 31735628. Link <https://pubmed.ncbi.nlm.nih.gov/31735628/>
199. Mantero V, Basilico P, Pozzetti U, Tonolo S, Rossi G, Spena G, Rigamonti A, Salmaggi A. Concomitant cerebral aspergillosis and mucormycosis in an immunocompetent woman treated with corticosteroids. J Neurovirol. 2020 Apr;26(2):277-280. doi: 10.1007/s13365-019-00804-4. Epub 2019 Nov 11. PMID: 31713052. Link <https://pubmed.ncbi.nlm.nih.gov/31713052/>
200. iang Y, Lu H. From the Pharynx to the Brain: A Case of Rapidly Progressing Mucormycosis. Mycopathologia. 2019 Dec;184(6):797-798. doi: 10.1007/s11046-019-00392-1. Epub 2019 Oct 26. PMID: 31655951. Link <https://pubmed.ncbi.nlm.nih.gov/31655951/>
201. Mascarella MA, Schweitzer L, Alreefi M, Silver J, Caglar D, Loo VG, Richardson K, Dufresne P, Lee TC, Sadeghi N. The infectious thyroid nodule: a case report of mucormycosis associated with ibrutinib therapy. J Otolaryngol Head Neck Surg. 2019 Oct 16;48(1):49. doi: 10.1186/s40463-019-0376-1. PMID: 31619294; PMCID: PMC6794875. Link <https://pubmed.ncbi.nlm.nih.gov/31619294/>
202. Li C, Zhu H, Tan Y, Liu D. Gastrointestinal bleeding due to duodenal mucormycosis in an immunocompetent host mimicking malignancy. Rev Esp Enferm Dig. 2019 Dec;111(12):961-962. doi: 10.17235/reed.2019.6462/2019. PMID: 31617363. Link <https://pubmed.ncbi.nlm.nih.gov/31617363/>
203. Lin L, Xue D, Lin TY, Wu YX, Jiang YT, Chen LM. Pulmonary aspergillosis, mucormycosis, and actinomycosis co-infection presenting as a cavitary lesion in a patient with diabetes. Chin Med J (Engl). 2019 Oct 20;132(20):2512-2513. doi: 10.1097/CM9.0000000000000468. PMID: 31613818; PMCID: PMC6831073. Link <https://pubmed.ncbi.nlm.nih.gov/31613818/>
204. Mani UM, Mohamed K, Krishna Kumar A, Inbarajan A. A modified technique to fabricate a complete hollow obturator for bilateral maxillectomy in a patient with mucormycosis-A technical case report. Spec Care Dentist. 2019 Nov;39(6):610-616. doi: 10.1111/scd.12423. Epub 2019 Oct 14. PMID: 31608482. Link <https://pubmed.ncbi.nlm.nih.gov/31608482/>
205. Malek A, Arias CA, Ostrosky L, Pankow S, Wanger A, Barnett B. A Fatal Case of Disseminated Mucormycosis Mimicking a Malignancy. Mycopathologia. 2019 Oct;184(5):699-700. doi: 10.1007/s11046-019-00396-x. Epub 2019 Oct 12. PMID: 31606811. Link <https://pubmed.ncbi.nlm.nih.gov/31606811/>
206. Tanabodee M, Sawasdipong J, Sudtikoonaseth P, Wessagowit V. Rare indolent zygomycosis caused by subcutaneous Saksenaea vasiformis infection. Australas J Dermatol. 2020 Feb;61(1):e94-e96. doi: 10.1111/ajd.13160. Epub 2019 Oct 2. PMID: 31578714. Link <https://pubmed.ncbi.nlm.nih.gov/31578714/>
207. Ojeda-Diezbarroso K, Aguilar-Rascón J, Jiménez-Juárez RN, Moreno-Espinosa S, Reséndiz-Sánchez J, Romero-Zamora JL. Successful posaconazole salvage therapy for rhinocerebral mucormycosis in a child with leukemia. Review of the literature. Rev Iberoam Micol. 2019 Jul-Sep;36(3):160-164. doi: 10.1016/j.riam.2018.07.008. Epub 2019 Sep 25. PMID: 31563327. Link <https://pubmed.ncbi.nlm.nih.gov/31563327/>
208. Arora SK, Swarnim S, Hemal A, Bidhuri N. Acute lymphoblastic leukemia presenting as nephromegaly in a child: A rare case report. Turk J Pediatr. 2019;61(1):97-101. doi: 10.24953/turkjped.2019.01.015. PMID: 31559728. Link <https://pubmed.ncbi.nlm.nih.gov/31559728/>
209. Wei LW, Wang H, Song YG, Yu J. Disfiguring Mucor irregularis Infection Cured by Amphotericin B and Itraconazole: A Case Report and Treatment Experience. Mycopathologia. 2019 Oct;184(5):677-682. doi: 10.1007/s11046-019-00380-5. Epub 2019 Sep 17. PMID: 31531755. Link <https://pubmed.ncbi.nlm.nih.gov/31531755/>
210. Belliere J, Rolland M, Tournier E, Cassaing S, Iriart X, Paul C, Kamar N. Early necrotic skin lesions after a ABO-incompatible kidney transplantation: The threat of Cunninghamella Spp. Transpl Infect Dis. 2019 Dec;21(6):e13173. doi: 10.1111/tid.13173. Epub 2019 Oct 21. PMID: 31529558. Link <https://pubmed.ncbi.nlm.nih.gov/31529558/>
211. Lee AS, Sullivan TJ. Orbital mycoses in a pediatric subtropical population: a case series. J AAPOS. 2019 Oct;23(5):270.e1-270.e7. doi: 10.1016/j.jaapos.2019.06.002. Epub 2019 Sep 9. PMID: 31513905. Link <https://pubmed.ncbi.nlm.nih.gov/31513905/>
212. Reis FPD, Campos SV, Aiello VD, Duarte MIS, Samano MN, Pego-Fernandes PM. Gastrointestinal mucormycosis post lung transplantation. Braz J Infect Dis. 2019 Sep-Oct;23(5):368-370. doi: 10.1016/j.bjid.2019.07.003. Epub 2019 Aug 30. Erratum in: Braz J Infect Dis. 2020 Jan - Feb;24(1):92. PMID: 31476279. Link <https://pubmed.ncbi.nlm.nih.gov/31476279/>
213. Marek C, Croxen MA, Dingle TC, Bharat A, Schwartz IS, Wiens R, Smith S. The use of genome sequencing to investigate an outbreak of hospital-acquired mucormycosis in transplant patients. Transpl Infect Dis. 2019 Oct;21(5):e13163. doi: 10.1111/tid.13163. Epub 2019 Sep 13. PMID: 31472083. Link <https://pubmed.ncbi.nlm.nih.gov/31472083/>
214. Samaras K, Markantonatou AM, Karapiperis D, Digonis P, Kartalis N, Kostogloudis N, Vyzantiadis TA. Saksenaea vasiformis infections: A case of an immunocompetent adult after mild injury and a literature review. J Mycol Med. 2019 Sep;29(3):260-264. doi: 10.1016/j.mycmed.2019.06.005. Epub 2019 Jul 25. PMID: 31445820. Link <https://pubmed.ncbi.nlm.nih.gov/31445820/>
215. Vermorel A, Issa N, Gabriel F, Accoceberry I, Valenzuela G, Darrigade AS, Camou F. A poisoned bouquet from Peru. Clin Microbiol Infect. 2019 Dec;25(12):1517-1518. doi: 10.1016/j.cmi.2019.08.010. Epub 2019 Aug 20. PMID: 31442609. Link <https://pubmed.ncbi.nlm.nih.gov/31442609/>
216. Bellanger AP, Berceanu A, Scherer E, Desbrosses Y, Daguindau E, Rocchi S, Millon L. Invasive Fungal Disease, Isavuconazole Treatment Failure, and Death in Acute Myeloid Leukemia Patients. Emerg Infect Dis. 2019 Sep;25(9):1778-1779. doi: 10.3201/eid2509.190598. PMID: 31441760; PMCID: PMC6711214. Link <https://pubmed.ncbi.nlm.nih.gov/31441760/>
217. Fadhel M, Patel SV, Liu E, Fune L, Wasserman EJ, Asif A. Disseminated Pulmonary with Isolated Muscular Mucormycosis in an Acute Myeloid Leukemia Patient: A Case Report and Literature Review. Am J Case Rep. 2019 Aug 16;20:1210-1215. doi: 10.12659/AJCR.916864. PMID: 31417073; PMCID: PMC6711263. Link <https://pubmed.ncbi.nlm.nih.gov/31417073/>
218. robisch A, Marterer R, Gorkiewicz G, Flaschberger S, Lackner H, Seidel M, Sperl D, Karastaneva A, Kohlmaier B, Egger M, Urban C, Benesch M, Strenger V. Invasive mucormycosis during treatment for acute lymphoblastic leukaemia-successful management of two life-threatening diseases. Support Care Cancer. 2020 May;28(5):2157-2161. doi: 10.1007/s00520-019-04962-3. Epub 2019 Aug 14. PMID: 31410599; PMCID: PMC7083803. Link <https://pubmed.ncbi.nlm.nih.gov/31410599/>
219. Berktaş B, Taşkapan H, Bayindir T, Kayabas U, Yildirim IO. Mucormycosis Presented with Facial Pain in a Renal Transplant Patient: A Case Report. Transplant Proc. 2019 Sep;51(7):2498-2500. doi: 10.1016/j.transproceed.2019.02.048. Epub 2019 Aug 9. PMID: 31405737. Link <https://pubmed.ncbi.nlm.nih.gov/31405737/>
220. Benjamin T, Wattier R, Dominic W. Allograft of Primary Cutaneous Mucormycosis in a Preterm Neonate: A Case Report. Wounds. 2019 Jul;31(7):E46-E48. PMID: 31373557. Link <https://pubmed.ncbi.nlm.nih.gov/31373557/>
221. Magaki S, Minasian T, Bork J, Harder SL, Deisch JK. Saksenaea infection masquerading as a brain tumor in an immunocompetent child. Neuropathology. 2019 Oct;39(5):382-388. doi: 10.1111/neup.12585. Epub 2019 Aug 2. PMID: 31373069. Link <https://pubmed.ncbi.nlm.nih.gov/31373069/>
222. Budhiraja R, Bhargava S, Sood N. Jejunal stricture due to mucormycosis. Trop Doct. 2019 Oct;49(4):318-320. doi: 10.1177/0049475519864250. Epub 2019 Jul 19. PMID: 31324130. Link https://pubmed.ncbi.nlm.nih.gov/31324130/
223. Rombola F, Spinoso A. Cranial nerve palsy: an unusual presentation of a fulminant fungal disease. New Microbiol. 2019 Oct;42(4):228-230. Epub 2019 Jul 15. PMID: 31305937. Link <https://pubmed.ncbi.nlm.nih.gov/31305937/>
224. Gupta S, Mehrotra A, Attri G, Pal L, Jaiswal AK, Kumar R. Isolated Intraventricular Chronic Mucormycosis in an Immunocompetent Infant: A Rare Case with Review of the Literature. World Neurosurg. 2019 Oct;130:206-210. doi: 10.1016/j.wneu.2019.06.190. Epub 2019 Jul 4. PMID: 31279104. Link <https://pubmed.ncbi.nlm.nih.gov/31279104/>
225. Uno K, Hishiya N, Matsuda M, Kai Y, Amano M, Nakamura A, Tohjyo T, Kawaguchi T, Nakano R, Yano H, Kasahara K, Mikasa K. Case of endobronchial metastasis from breast cancer accompanied with Cunninghamella bertholletiae tracheobronchial mycetoma. J Infect Chemother. 2019 Dec;25(12):1065-1069. doi: 10.1016/j.jiac.2019.05.031. Epub 2019 Jun 19. PMID: 31227382. <https://pubmed.ncbi.nlm.nih.gov/31227382/>
226. Singh PK, Saxena P. Reverse halo sign in uncontrolled patient with type 2 diabetes. BMJ Case Rep. 2019 Jun 18;12(6):e230111. doi: 10.1136/bcr-2019-230111. PMID: 31217218; PMCID: PMC6586211. Link <https://pubmed.ncbi.nlm.nih.gov/31217218/>
227. Bhattacharya D, Iyer R, Nallasamy K, Vaiphei K. Haemophagocytic lymphohistiocytosis with pulmonary mucormycosis: fatal association. BMJ Case Rep. 2019 May 30;12(5):e230587. doi: 10.1136/bcr-2019-230587. PMID: 31151981; PMCID: PMC6557396. Link <https://pubmed.ncbi.nlm.nih.gov/31151981/>
228. Souza LVS, Souza AS, Marchiori E. Mucormycotic Pulmonary Pseudoaneurysm Causing Fatal Hemoptysis. Arch Bronconeumol (Engl Ed). 2019 Nov;55(11):597-598. English, Spanish. doi: 10.1016/j.arbres.2019.03.019. Epub 2019 May 18. PMID: 31113670. Link https://pubmed.ncbi.nlm.nih.gov/31113670/
229. Vahabzadeh-Hagh AM, Chao KY, Blackwell KE. Invasive Oral Tongue Mucormycosis Rapidly Presenting After Orthotopic Liver Transplant. Ear Nose Throat J. 2019 Jun;98(5):268-270. doi: 10.1177/0145561319840535. Epub 2019 May 13. PMID: 31079483. Link <https://pubmed.ncbi.nlm.nih.gov/31079483/>
230. Chae HK, Lim J, Lee SC, Kim KS. The Significance of "Black Turbinate Sign" on MRI. Ear Nose Throat J. 2020 Jul;99(6):395-396. doi: 10.1177/0145561319847628. Epub 2019 May 9. PMID: 31072199. Link <https://pubmed.ncbi.nlm.nih.gov/31072199/>
231. de Roquetaillade C, Paktoris H, Hamon M, Bruneel F. Disseminated mucormycosis. Intensive Care Med. 2019 Oct;45(10):1480-1481. doi: 10.1007/s00134-019-05625-x. Epub 2019 May 6. PMID: 31062048. Link <https://pubmed.ncbi.nlm.nih.gov/31062048/>
232. Ally MT, Jenkins IH, Gupta V. Mucormycosis: More Than Meets the Eye! Am J Med. 2019 Sep;132(9):1044-1046. doi: 10.1016/j.amjmed.2019.04.007. Epub 2019 Apr 29. PMID: 31047865. Link <https://pubmed.ncbi.nlm.nih.gov/31047865/>
233. Cooper JD, Gotoff RA, Foltzer MA, Carter RA, Walsh TJ. Mediastinal Mucormycosis: Case report, review of literature and treatment with continuous liposomal amphotericin B irrigation. Mycoses. 2019 Sep;62(9):739-745. doi: 10.1111/myc.12922. Epub 2019 May 23. PMID: 31044442. Link <https://pubmed.ncbi.nlm.nih.gov/31044442/>
234. Narechania S, Koval CE, Chaisson NF. A Man with Diabetes and Nonresolving Cavitary Pneumonia. Ann Am Thorac Soc. 2019 May;16(5):626-630. doi: 10.1513/AnnalsATS.201807-465CC. PMID: 31042090. Link <https://pubmed.ncbi.nlm.nih.gov/31042090/>
235. Chen IW, Lin CW. Rhino-orbital-cerebral mucormycosis. CMAJ. 2019 Apr 23;191(16):E450. doi: 10.1503/cmaj.181210. PMID: 31015350; PMCID: PMC6476717. Link <https://pubmed.ncbi.nlm.nih.gov/31015350/>
236. Safai Nodeh SR, Dehghan Manshadi SA, Jahanbin B, Khodaveisi S, Giasvand F, Seifi A, Salehi M. Invasive fungal consecutive infections in a patient with acute myeloid leukaemia. Niger J Clin Pract. 2019 Apr;22(4):582-584. doi: 10.4103/njcp.njcp_359_17. PMID: 30975967. Link <https://pubmed.ncbi.nlm.nih.gov/30975967/>
237. de Moura Feitoza L, Altemani A, Adolfo da Silva N Jr, Reis F. Teaching NeuroImages: Mucormycosis-associated vasculitis: A new sequence to show an old invasive infection. Neurology. 2019 Apr 9;92(15):e1796-e1797. doi: 10.1212/WNL.0000000000007275. PMID: 30962306. Link <https://pubmed.ncbi.nlm.nih.gov/30962306/>
238. Ezanno AC, Perrenot C, Guerci-Bresler A, Aoun O, Bresler L. Bowel infarction due to mucormycosis in an immunocompromised patient. Med Mal Infect. 2019 Sep;49(6):479-481. doi: 10.1016/j.medmal.2019.03.005. Epub 2019 Mar 28. PMID: 30929970. <https://pubmed.ncbi.nlm.nih.gov/30929970/>
239. Peng H, Xiao J, Wan H, Shi J, Li J. Severe Gastric Mycormycosis Infection Followed by Cytomegalovirus Pneumonia in a Renal Transplant Recipient: A Case Report and Concise Review of the Literature. Transplant Proc. 2019 Mar;51(2):556-560. doi: 10.1016/j.transproceed.2018.12.023. Epub 2019 Jan 3. PMID: 30879589. Link <https://pubmed.ncbi.nlm.nih.gov/30879589/>
240. Zhang Q, Liu H, Qiu S, Wang W, Yang L, Chen H, Chen X, Shen Z. A Rare Case of Pulmonary Coinfection by Lichtheimia ramosa and Aspergillus fumigatus in a Patient With Delayed Graft Function After Renal Transplantation. Transplant Proc. 2019 Mar;51(2):551-555. doi: 10.1016/j.transproceed.2018.12.006. Epub 2018 Dec 12. PMID: 30879588. Link <https://pubmed.ncbi.nlm.nih.gov/30879588/>
241. Rashid S, Ben Abid F, Babu S, Christner M, Alobaidly A, Al Ansari AAA, Akhtar M. Fatal renal mucormycosis with Apophysomyces elegans in an apparently healthy male. Aging Male. 2020 Dec;23(5):746-749. doi: 10.1080/13685538.2019.1586871. Epub 2019 Mar 16. PMID: 30879364. Link <https://pubmed.ncbi.nlm.nih.gov/30879364/>
242. Makino K, Aoi J, Egashira S, Honda N, Kubo Y, Kawakami Y, Hayashi H, Mochizuki T. Multiple Skin Abscesses Caused by Rhizopus sp. Infection after Candida albicans Infection in an Immunocompromised Patient. Med Mycol J. 2019;60(1):17-21. doi: 10.3314/mmj.18-00002. PMID: 30814466. Link <https://pubmed.ncbi.nlm.nih.gov/30814466/>
243. Manji F, Lam JC, Meatherall BL, Church D, Missaghi B. Severe facial necrosis in a type 1 diabetic patient secondary to mucormycosis masquerading as an internal maxillary artery occlusion: a case report. BMC Infect Dis. 2019 Feb 22;19(1):184. doi: 10.1186/s12879-019-3822-9. PMID: 30795757; PMCID: PMC6387511. Link <https://pubmed.ncbi.nlm.nih.gov/30795757/>
244. Morado-Aramburo O, Ortiz-Brizuela E, Méndez-Flores S, Cuellar-Rodríguez J. Necrotic skin ulcers in an immunocompromised patient. Enferm Infecc Microbiol Clin (Engl Ed). 2019 Aug-Sep;37(7):476-479. English, Spanish. doi: 10.1016/j.eimc.2019.01.005. Epub 2019 Feb 15. PMID: 30777346. Link <https://pubmed.ncbi.nlm.nih.gov/30777346/>
245. Itakusu K, Inoue T, Abe M, Ueda R, Sakurai A, Miyazaki Y, Nakase K, Yoshida I, Nawa Y. [Acute myeloid leukemia with sudden onset bilateral lower extremity paralysis caused by disseminated mucormycosis following unrelated bone marrow transplantation]. Rinsho Ketsueki. 2019;60(1):17-21. Japanese. doi: 10.11406/rinketsu.60.17. PMID: 30726818. Link <https://pubmed.ncbi.nlm.nih.gov/30726818/>
246. Jimenez-Cauhe J, Molins-Ruiz M, Fernandez-Guarino M. Rapidly progressing ulcer and a urine drainage bag. Dermatol Online J. 2018 Nov 15;24(11):13030/qt4r53q48g. PMID: 30695980. Link <https://pubmed.ncbi.nlm.nih.gov/30695980/>
247. Totadri S, Sundersingh S, Natarajan R, Seshadri RA, Radhakrishnan V. Gastrointestinal mucormycosis in a child with acute lymphoblastic leukemia: An uncommon but ominous complication. Indian J Cancer. 2018 Jul-Sep;55(3):304-305. doi: 10.4103/ijc.IJC_260_18. PMID: 30693900. Link <https://pubmed.ncbi.nlm.nih.gov/30693900/>
248. Grossi O, Pineau S, Sadot-Lebouvier S, Hay B, Delaunay J, Miailhe AF, Bretonnière C, Jeddi F, Lavergne RA, Le Pape P. Disseminated mucormycosis due to Lichtheimia corymbifera during ibrutinib treatment for relapsed chronic lymphocytic leukaemia: a case report. Clin Microbiol Infect. 2019 Feb;25(2):261-263. doi: 10.1016/j.cmi.2018.10.004. Epub 2018 Oct 26. PMID: 30691617. Link <https://pubmed.ncbi.nlm.nih.gov/30691617/>
249. Debureaux PE, Paccoud O, Guitard J, Baujat B, Ruggeri A, Battipaglia G, Duléry R, Giannotti F, Malard F, Mohty M, Brissot E. Rhino-orbital Mucormycosis presenting as facial cellulitis in a patient with high-risk acute myeloid leukemia in relapse. Curr Res Transl Med. 2019 May;67(2):76-78. doi: 10.1016/j.retram.2019.01.004. Epub 2019 Jan 25. PMID: 30686650. Link <https://pubmed.ncbi.nlm.nih.gov/30686650/>
250. Sahu KK, Yanamandra U, Kakkar N, Malhotra P. Rare Presentation of Mucormycosis in Aplastic Anaemia: Isolated Hepatic Mucormycosis. Mycopathologia. 2019 Apr;184(2):347-348. doi: 10.1007/s11046-018-0309-y. Epub 2019 Jan 25. PMID: 30684143. Link <https://pubmed.ncbi.nlm.nih.gov/30684143/>
251. Bhatt M, Soneja M, Fazal F, Vyas S, Kumar P, Jorwal P, Raj U, Sachdev J, Singh G, Xess I, Alam S, Biswas A. Two cases of Osteoarticular Mucor menace: A diagnostic and management conundrum. Drug Discov Ther. 2018;12(6):374-378. doi: 10.5582/ddt.2018.01064. PMID: 30674773. Link <https://pubmed.ncbi.nlm.nih.gov/30674773/>
252. Fu MH, Liu J, Liang GZ, Li CR, Zhu XM, Wang L, Chen H, Hu WL, Lv GX, Liu WD. Successful Treatment of Eczema-Like Mucormycosis in a Child by Combination of Intravenous Drip and Percutaneous Injection Amphotericin B. Mycopathologia. 2019 Apr;184(2):309-313. doi: 10.1007/s11046-018-0273-6. Epub 2019 Jan 22. PMID: 30666543. Link <https://pubmed.ncbi.nlm.nih.gov/30666543/>
253. Adamsick ML, Elshaboury RH, Gift T, Mansour MK, Kotton CN, Gandhi RG. Therapeutic drug concentrations of isavuconazole following the administration of isavuconazonium sulfate capsules via gastro-jejunum tube: A case report. Transpl Infect Dis. 2019 Apr;21(2):e13048. doi: 10.1111/tid.13048. Epub 2019 Jan 29. PMID: 30636363. Link <https://pubmed.ncbi.nlm.nih.gov/30636363/>
254. McKenzie S, Zang P, Blackcloud P, Cohen B, Truong A, Worswick S, Arzeno J. Case series of cutaneous mucormycosis in the setting of Herpesviridae infection. Br J Dermatol. 2019 Aug;181(2):373-374. doi: 10.1111/bjd.17631. Epub 2019 Mar 26. PMID: 30633321. Link <https://pubmed.ncbi.nlm.nih.gov/30633321/>
255. Dotis J, Printza N, Stabouli S, Karava V, Gkogka C, Vyzantiadis TA, Roilides E, Papachristou F. Disseminated mucormycosis in an adolescent kidney transplant recipient. Kidney Int. 2019 Jan;95(1):236. doi: 10.1016/j.kint.2018.07.006. PMID: 30606422. Link <https://pubmed.ncbi.nlm.nih.gov/30606422/>
256. Liang CN, Li W, Xu MJ, Ma HF, Zhao HW, Wang QY, Kang J, Hou G. Pulmonary mucormycosis with bronchial fistula successfully treated with bronchoscopic and medical therapy. Int J Tuberc Lung Dis. 2018 Dec 1;22(12):1525-1527. doi: 10.5588/ijtld.18.0420. PMID: 30606329. Link <https://pubmed.ncbi.nlm.nih.gov/30606329/>
257. Hirano M, Ota Y, Koibuchi T, Takei T, Takeda R, Kawamata T, Yokoyama K, Uchimaru K, Yotsuyanagi H, Imai Y, Tojo A. Nested Polymerase Chain Reaction with Specific Primers for Mucorales in the Serum of Patients with Hematological Malignancies. Jpn J Infect Dis. 2019 May 23;72(3):196-198. doi: 10.7883/yoken.JJID.2018.379. Epub 2018 Dec 25. PMID: 30584197. Link <https://pubmed.ncbi.nlm.nih.gov/30584197/>
258. Yeo CD, Kim JS, Kwon SH, Lee EJ, Lee MH, Kim SG, You YS, Kim JS, Lee JH, Ryu JS. Rhinocerebral mucormycosis after functional endoscopic sinus surgery: A case report. Medicine (Baltimore). 2018 Dec;97(51):e13290. doi: 10.1097/MD.0000000000013290. PMID: 30572431; PMCID: PMC6319933. Link <https://pubmed.ncbi.nlm.nih.gov/30572431/>
259. Yang J, Zhang J, Feng Y, Peng F, Fu F. A case of pulmonary mucormycosis presented as Pancoast syndrome and bone destruction in an immunocompetent adult mimicking lung carcinoma. J Mycol Med. 2019 Apr;29(1):80-83. doi: 10.1016/j.mycmed.2018.10.005. Epub 2018 Dec 12. PMID: 30553628. Link <https://pubmed.ncbi.nlm.nih.gov/30553628/>
260. Sękowska A, Prażyńska M, Twarużek M, Deptuła A, Zastempowska E, Soszczyńska E, Gospodarek-Komkowska E. Fulminant mucormycosis after a traffic accident: a case report. Folia Microbiol (Praha). 2019 May;64(3):429-433. doi: 10.1007/s12223-018-00671-2. Epub 2018 Dec 14. PMID: 30552581. Link <https://pubmed.ncbi.nlm.nih.gov/30552581/>
261. Vulsteke JB, Deeren D. Cutaneous mucormycosis. Transpl Infect Dis. 2019 Apr;21(2):e13039. doi: 10.1111/tid.13039. Epub 2019 Jan 4. PMID: 30548761. Link <https://pubmed.ncbi.nlm.nih.gov/30548761/>
262. Cornu M, Bruno B, Loridant S, Navarin P, François N, Lanternier F, Amzallag-Bellenger E, Dubos F, Mazingue F, Sendid B. Successful outcome of disseminated mucormycosis in a 3-year-old child suffering from acute leukaemia: the role of isavuconazole? A case report. BMC Pharmacol Toxicol. 2018 Dec 6;19(1):81. doi: 10.1186/s40360-018-0273-7. PMID: 30522521; PMCID: PMC6282241. Link <https://pubmed.ncbi.nlm.nih.gov/30522521/>
263. Thomas S, Pawar B, Fernandes D, Nayar S, George P, Cherian S. An Unusual Case of Pulmonary Mucormycosis. Transplant Proc. 2018 Dec;50(10):3943-3945. doi: 10.1016/j.transproceed.2018.06.001. Epub 2018 Jun 6. PMID: 30503525. Link <https://pubmed.ncbi.nlm.nih.gov/30503525/>
264. Sethi J, Ramachandran R, Kohli HS, Gupta KL. Isolated renal mucormycosis in a patient with Idiopathic CD4 lymphocytopenia. BMJ Case Rep. 2018 Nov 8;2018:bcr2018225234. doi: 10.1136/bcr-2018-225234. PMID: 30413437; PMCID: PMC6229138. Link <https://pubmed.ncbi.nlm.nih.gov/30413437/>
265. Gupta AK, Parwal C, Mangal M, Gambhir SS, Nanda BS, Sarangi K. Treating mucormycosis using a multimodality approach: a case series. J Wound Care. 2018 Nov 2;27(11):735-742. doi: 10.12968/jowc.2018.27.11.735. PMID: 30398934. Link <https://pubmed.ncbi.nlm.nih.gov/30398934/>
266. Soto-Castillo JJ, Fortún-Abete J, Soria-Rivas A. Black ulcer in leg. Enferm Infecc Microbiol Clin (Engl Ed). 2019 May;37(5):344-346. English, Spanish. doi: 10.1016/j.eimc.2018.09.005. Epub 2018 Oct 30. PMID: 30389263. Link <https://pubmed.ncbi.nlm.nih.gov/30389263/>
267. Parize P, Mamez AC, Garcia-Hermoso D, Dumaine V, Poirée S, Kauffmann-Lacroix C, Jullien V, Lortholary O, Lanternier F. Successful Treatment of Saksenaea sp. Osteomyelitis by Conservative Surgery and Intradiaphyseal Incorporation of Amphotericin B Cement Beads. Antimicrob Agents Chemother. 2018 Dec 21;63(1):e01006-18. doi: 10.1128/AAC.01006-18. PMID: 30373790; PMCID: PMC6325185. Link <https://pubmed.ncbi.nlm.nih.gov/30373790/>
268. Fernández Tormos E, Corella Montoya F, Martínez Izquierdo MÁ, Sánchez-Artola B, Limousin Aranzabal B, Larraínzar-Garijo R. Infection Due to Saksenaea vasiformis Following a Spider Bite. J Hand Surg Am. 2019 Jul;44(7):619.e1-619.e5. doi: 10.1016/j.jhsa.2018.08.020. Epub 2018 Oct 18. PMID: 30344020. Link https://pubmed.ncbi.nlm.nih.gov/30344020/
269. Zbiba W, Bouayed E, Ben Abdesslem N, Elleuch I, Kharrat M. Ophtalmoplegia complicating sino-orbital mucormycosis. Tunis Med. 2018 Mar;96(3):224-226. PMID: 30325492. Link <https://pubmed.ncbi.nlm.nih.gov/30325492/>
270. Beketova TR, Bailey L, Crowell EL, Supsupin EP, Adesina OO. Orbitocerebral Mucormycosis in a Patient With Central Nervous System Lymphoma. Ophthalmic Plast Reconstr Surg. 2018 Nov/Dec;34(6):e197-e201. doi: 10.1097/IOP.0000000000001243. PMID: 30320719; PMCID: PMC6231966. Link <https://pubmed.ncbi.nlm.nih.gov/30320719/>
271. Mathew G, Arumugam V, Murugesan S, Duhli N, Agarwal I. Renal Mucormycosis: A Rare Cause of Urinary Tract Infection Leading to End-stage Renal Disease (ESRD). J Trop Pediatr. 2019 Aug 1;65(4):405-408. doi: 10.1093/tropej/fmy059. PMID: 30252109. Link <https://pubmed.ncbi.nlm.nih.gov/30252109/>
272. Mukherjee B, Kundu D. Necrotizing fungal infection due to Saksenaea erythrospora: A case report and review of literature. Indian J Ophthalmol. 2018 Oct;66(10):1513-1516. doi: 10.4103/ijo.IJO_389_18. PMID: 30249859; PMCID: PMC6173015. Link <https://pubmed.ncbi.nlm.nih.gov/30249859/>
273. Naor S, Sher O, Grisaru-Soen G, Levin D, Elhasid R, Geffen Y, Hershkovitz D, Aizic A. Mucor Appendicitis Resolution Following Surgical Excision without Antifungal Therapy. Isr Med Assoc J. 2018 Sep;20(9):592-593. PMID: 30221879. Link <https://pubmed.ncbi.nlm.nih.gov/30221879/>
274. Fattah SY, Hariri F, Ngui R, Husman SI. Tongue necrosis secondary to mucormycosis in a diabetic patient: A first case report in Malaysia. J Mycol Med. 2018 Sep;28(3):519-522. doi: 10.1016/j.mycmed.2018.06.004. Epub 2018 Jul 7. PMID: 30205883. Link <https://pubmed.ncbi.nlm.nih.gov/30205883/>
275. Hagemann JB, Haverkamp S, Grüner B, Kuchenbauer F, Essig A. Pulmonary Campylobacter concisus infection in an immunocompromised patient with underlying mucormycosis. Int J Infect Dis. 2018 Nov;76:45-47. doi: 10.1016/j.ijid.2018.08.021. Epub 2018 Sep 8. PMID: 30201509. Link <https://pubmed.ncbi.nlm.nih.gov/30201509/>
276. Dogra M, Bhutani G, Gupta V. Mucormycosis Endophthalmitis in a Silicone Oil-Filled Eye of an Immunocompetent Patient. Ocul Immunol Inflamm. 2019;27(8):1293-1295. doi: 10.1080/09273948.2018.1518462. Epub 2018 Sep 7. PMID: 30192700. Link <https://pubmed.ncbi.nlm.nih.gov/30192700/>
277. Wang X, Wang A, Wang X, Li R, Yu J. Cutaneous mucormycosis caused by Mucor irregularis in a patient with CARD9 deficiency. Br J Dermatol. 2019 Jan;180(1):213-214. doi: 10.1111/bjd.17144. Epub 2018 Oct 14. PMID: 30187457. Link <https://pubmed.ncbi.nlm.nih.gov/30187457/>
278. Sakamoto H, Itonaga H, Sawayama Y, Taguchi J, Saijo T, Kuwatsuka S, Hashisako M, Kinoshita N, Oishi M, Doi H, Kosai K, Nishimoto K, Tanaka K, Yanagihara K, Mukae H, Izumikawa K, Miyazaki Y. Primary Oral Mucormycosis Due to Rhizopus microsporus after Allogeneic Stem Cell Transplantation. Intern Med. 2018;57(17):2567-2571. doi: 10.2169/internalmedicine.0474-17. Epub 2018 Sep 1. PMID: 30175728; PMCID: PMC6172532 Link <https://pubmed.ncbi.nlm.nih.gov/30175728/>
279. France K, Stoopler ET, Tanaka TI. Palatal Swelling in a Patient With Refractory Leukemia. JAMA Dermatol. 2019 Jan 1;155(1):109-110. doi: 10.1001/jamadermatol.2018.2299. PMID: 30140847. Link <https://pubmed.ncbi.nlm.nih.gov/30140847/>
280. Clemente-Gutiérrez U, Perez-Soto RH, Álvarez-Bautista FE, Domínguez-Rosado I, Cuellar-Mendoza M. Gastrointestinal mucormycosis: An atypical cause of abdominal pain in an immunocompromised patient. Rev Gastroenterol Mex (Engl Ed). 2019 Jul-Sep;84(3):409-411. English, Spanish. doi: 10.1016/j.rgmx.2018.06.003. Epub 2018 Aug 17. PMID: 30126657. Link <https://pubmed.ncbi.nlm.nih.gov/30126657/>
281. Salinas TJ, Sinha N, Revuru V, Arce K. Prosthetic rehabilitation of a maxillary defect with a bone anchored prosthesis: A clinical report. J Prosthet Dent. 2019 Jan;121(1):173-178. doi: 10.1016/j.prosdent.2018.03.013. Epub 2018 Aug 7. PMID: 30093120. Link <https://pubmed.ncbi.nlm.nih.gov/30093120/>
282. Chang H, Wang PN, Huang Y. Cutaneous mucormycosis. Infection. 2018 Dec;46(6):901-902. doi: 10.1007/s15010-018-1179-4. Epub 2018 Aug 8. PMID: 30091018. Link <https://pubmed.ncbi.nlm.nih.gov/30091018/>
283. Brondfield S, Kaplan L, Dhaliwal G. Palatal Mucormycosis. J Gen Intern Med. 2018 Oct;33(10):1815. doi: 10.1007/s11606-018-4481-z. Epub 2018 May 9. PMID: 30088205; PMCID: PMC6153208. Link <https://pubmed.ncbi.nlm.nih.gov/30088205/>
284. Gkegkes ID, Kotrogiannis I, Konstantara F, Karetsou A, Tsiplakou S, Fotiou E, Stamopoulou S, Papazacharias C, Paraskevopoulos IA. Cutaneous Mucormycosis by Saksenaea vasiformis: An Unusual Case Report and Review of Literature. Mycopathologia. 2019 Feb;184(1):159-167. doi: 10.1007/s11046-018-0249-6. Epub 2018 Jul 30. PMID: 30062390. Link <https://pubmed.ncbi.nlm.nih.gov/30062390/>
285. Shah M, Nel J, Almansouri A, Van Duin D, Gerber DA. Combined Medical and Surgical Management of Hepatic Mucormycosis in an Adult with Acute Myeloid Leukemia: Case Report and Review of the Literature. Mycopathologia. 2019 Feb;184(1):155-158. doi: 10.1007/s11046-018-0289-y. Epub 2018 Jul 30. PMID: 30062389. Link <https://pubmed.ncbi.nlm.nih.gov/30062389/>
286. Tomotaki S, Takeyama E, Tanaka M, Ohyama M, Tanaka Y. Mucor mycelial thrombosis of the portal vein in an extremely low-birthweight infant. Pediatr Int. 2018 Aug;60(8):764-766. doi: 10.1111/ped.13611. Epub 2018 Jul 30. PMID: 30058248. Link <https://pubmed.ncbi.nlm.nih.gov/30058248/>
287. Karigane D, Kikuchi T, Sakurai M, Kato J, Yamane Y, Hashida R, Abe R, Hatano M, Hasegawa N, Wakayama M, Shibuya K, Okamoto S, Mori T. Invasive hepatic mucormycosis: A case report and review of the literature. J Infect Chemother. 2019 Jan;25(1):50-53. doi: 10.1016/j.jiac.2018.06.013. Epub 2018 Jul 26. PMID: 30057341. Link <https://pubmed.ncbi.nlm.nih.gov/30057341/>
288. Chatelain E, Grateau A, Baudry T, Argaud L. Autopsy-confirmed fulminant mucormycosis: a skin lesion revealing multiple organ dissemination. Intensive Care Med. 2019 May;45(5):694-696. doi: 10.1007/s00134-018-5331-5. Epub 2018 Jul 25. PMID: 30046873. Link <https://pubmed.ncbi.nlm.nih.gov/30046873/>
289. Pomorska A, Malecka A, Jaworski R, Radon-Proskura J, Hare RK, Nielsen HV, Andersen LO, Jensen HE, Arendrup MC, Irga-Jaworska N. Isavuconazole in a Successful Combination Treatment of Disseminated Mucormycosis in a Child with Acute Lymphoblastic Leukaemia and Generalized Haemochromatosis: A Case Report and Review of the Literature. Mycopathologia. 2019 Feb;184(1):81-88. doi: 10.1007/s11046-018-0287-0. Epub 2018 Jul 23. PMID: 30039238. <https://pubmed.ncbi.nlm.nih.gov/30039238/>
290. Elgarten CW, Levy EM, Mattei P, Fisher BT, Olson TS, Freedman JL. Successful treatment of pulmonary mucormycosis in two pediatric hematopoietic stem cell transplant patients. Pediatr Transplant. 2018 Nov;22(7):e13270. doi: 10.1111/petr.13270. Epub 2018 Jul 16. PMID: 30014584. Link <https://pubmed.ncbi.nlm.nih.gov/30014584/>
291. Bhatt H, Zilani G, Hayhurst C. Orbitocerebral mucormycosis and intracranial haemorrhage: a role for caution with steroids in suspected giant cell arteritis. BMJ Case Rep. 2018 Jul 15;2018:bcr2017224086. doi: 10.1136/bcr-2017-224086. PMID: 30012677; PMCID: PMC6047693. Link <https://pubmed.ncbi.nlm.nih.gov/30012677/>
292. Bannykh SI, Hunt B, Moser F. Intra-arterial spread of Mucormycetes mediates early ischemic necrosis of brain and suggests new venues for prophylactic therapy. Neuropathology. 2018 Oct;38(5):539-541. doi: 10.1111/neup.12501. Epub 2018 Jul 4. PMID: 29974522. Link <https://pubmed.ncbi.nlm.nih.gov/29974522/>
293. Abreu BFBB, Duarte ML, Santos LRD, Sementilli A, Figueiras FN. A rare case of gastric mucormycosis in an immunocompetent patient. Rev Soc Bras Med Trop. 2018 May-Jun;51(3):401-402. doi: 10.1590/0037-8682-0304-2017. PMID: 29972579. Link <https://pubmed.ncbi.nlm.nih.gov/29972579/>
294. Thielen BK, Barnes AMT, Sabin AP, Huebner B, Nelson S, Wesenberg E, Hansen GT. Widespread Lichtheimia Infection in a Patient with Extensive Burns: Opportunities for Novel Antifungal Agents. Mycopathologia. 2019 Feb;184(1):121-128. doi: 10.1007/s11046-018-0281-6. Epub 2018 Jul 2. PMID: 29967971; PMCID: PMC6445638. Link <https://pubmed.ncbi.nlm.nih.gov/29967971/>
295. Barg AA, Malkiel S, Bartuv M, Greenberg G, Toren A, Keller N. Successful treatment of invasive mucormycosis with isavuconazole in pediatric patients. Pediatr Blood Cancer. 2018 Oct;65(10):e27281. doi: 10.1002/pbc.27281. Epub 2018 Jun 22. PMID: 29932282. Link <https://pubmed.ncbi.nlm.nih.gov/29932282/>
296. Coleman E, Levy L, Panse G, Leventhal JS. Necrotic cheek ulcer in a liver transplant patient. Int J Dermatol. 2019 Mar;58(3):285-287. doi: 10.1111/ijd.14101. Epub 2018 Jun 21. PMID: 29926899; PMCID: PMC6309531. Link <https://pubmed.ncbi.nlm.nih.gov/29926899/>
297. Wang Y, Zhu M, Bao Y, Li L, Zhu L, Li F, Xu J, Liang J. Cutaneous mucormycosis caused by Rhizopus microsporus in an immunocompetent patient: A case report and review of literature. Medicine (Baltimore). 2018 Jun;97(25):e11141. doi: 10.1097/MD.0000000000011141. PMID: 29924015; PMCID: PMC6024029. Link <https://pubmed.ncbi.nlm.nih.gov/29924015/>
298. Geng C, Lv X, Li J, Jiang Q, Yang R, Zhan P. Chronic subcutaneous infection due to Lichtheimia ramosa. J Eur Acad Dermatol Venereol. 2019 Jan;33(1):e26-e29. doi: 10.1111/jdv.15137. Epub 2018 Jul 6. PMID: 29911306. Link <https://pubmed.ncbi.nlm.nih.gov/29911306/>
299. Gochhait D, Alexender A, Dehuri P, Rangarajan V, Jacob SE, Siddaraju N. Mucormycotic parotitis and otitis causing facial nerve palsy in a diabetic patient. Cytopathology. 2018 Dec;29(6):582-584. doi: 10.1111/cyt.12600. Epub 2018 Jul 30. PMID: 29904969. Link <https://pubmed.ncbi.nlm.nih.gov/29904969/>
300. Jones MU, Flores MS, Vereen RJ, Szabo SR, Logemann NF, Eberly MD. Self-resolving superficial primary cutaneous mucormycosis in a 7-week-old infant. Pediatr Dermatol. 2018 Jul;35(4):e248-e250. doi: 10.1111/pde.13531. Epub 2018 May 30. PMID: 29846009. Link <https://pubmed.ncbi.nlm.nih.gov/29846009/>
301. Devi RU, Balachandran A, Kamalarathnam CN, Pappathi S. Neonatal Mucormycosis with Gastrointestinal and Cutaneous involvement. Indian Pediatr. 2018 May 15;55(5):427-428. PMID: 29845959. Link <https://pubmed.ncbi.nlm.nih.gov/29845959/>
302. Sharifpour A, Gholinejad-Ghadi N, Ghasemian R, Seifi Z, Aghili SR, Zaboli E, Abdi R, Shokohi T. Voriconazole associated mucormycosis in a patient with relapsed acute lymphoblastic leukemia and hematopoietic stem cell transplant failure: A case report. J Mycol Med. 2018 Sep;28(3):527-530. doi: 10.1016/j.mycmed.2018.05.008. Epub 2018 May 26. PMID: 29807852. Link <https://pubmed.ncbi.nlm.nih.gov/29807852/>
303. Kalogerakos PD, Kiparakis M, Pavlaki K, Pontikoglou C, Lazopoulos G, Chalkiadakis G. Thoracoabdominal Resection of Mucormycosis Lesions in a Leukemic Patient. Ann Thorac Surg. 2018 Nov;106(5):e239-e241. doi: 10.1016/j.athoracsur.2018.04.066. Epub 2018 May 26. PMID: 29807006. Link <https://pubmed.ncbi.nlm.nih.gov/29807006/>
304. Danneels P, Mahieu R, Dib M, Abgueguen P, Rabier V, Klosek M, Le Govic Y, Dubée V. On the Nose: Disseminated Mucormycosis. Am J Med. 2018 Oct;131(10):1182-1184. doi: 10.1016/j.amjmed.2018.05.008. Epub 2018 May 26. PMID: 29806997. Link <https://pubmed.ncbi.nlm.nih.gov/29806997/>
305. Rangwala SD, Strickland BA, Rennert RC, Ravina K, Bakhsheshian J, Hurth K, Giannotta SL, Russin JJ. Ruptured Mycotic Aneurysm of the Distal Circulation in a Patient with Mucormycosis Without Direct Skull Base Extension: Case Report. Oper Neurosurg (Hagerstown). 2019 Mar 1;16(3):E101-E107. doi: 10.1093/ons/opy127. PMID: 29800469. Link <https://pubmed.ncbi.nlm.nih.gov/29800469/>
306. Swain SK, Sahu MC, Banerjee A. Non-sinonasal isolated facio-orbital mucormycosis - A case report. J Mycol Med. 2018 Sep;28(3):538-541. doi: 10.1016/j.mycmed.2018.05.003. PMID: 29773436. Link <https://pubmed.ncbi.nlm.nih.gov/29773436/>
307. Mertens A, Barche D, Scheinpflug L, Scholz FG, Vielhaber S, Scherlach C, Tröger U, Geginat G, Färber J, Arens C. Rhinocerebrale Mucormykose [Rhinocerebral Mucormycosis]. Laryngorhinootologie. 2018 Aug;97(8):550-554. German. doi: 10.1055/a-0619-5143. Epub 2018 May 16. PMID: 29768642. Link <https://pubmed.ncbi.nlm.nih.gov/29768642/>
308. Termos S, Othman F, Alali M, Al Bader BMS, Alkhadher T, Hassanaiah WF, Taqi A, Sapkal A. Total Gastric Necrosis Due to Mucormycosis: A Rare Case of Gastric Perforation. Am J Case Rep. 2018 May 4;19:527-533. doi: 10.12659/AJCR.908952. PMID: 29724988; PMCID: PMC5956728. Link <https://pubmed.ncbi.nlm.nih.gov/29724988/>
309. Bellanger AP, Berceanu A, Rocchi S, Valot B, Fontan J, Chauchet A, Belin N, Scherer E, Deconinck E, Navellou JC, Millon L. Development of a quantitative PCR detecting Cunninghamella bertholletiae to help in diagnosing this rare and aggressive mucormycosis. Bone Marrow Transplant. 2018 Sep;53(9):1180-1183. doi: 10.1038/s41409-018-0194-5. Epub 2018 Apr 30. PMID: 29712993. Link <https://pubmed.ncbi.nlm.nih.gov/29712993/>
310. Jevalikar G, Sudhanshu S, Mahendru S, Sarma S, Farooqui KJ, Mithal A. Cutaneous mucormycosis as a presenting feature of type 1 diabetes in a boy - case report and review of the literature. J Pediatr Endocrinol Metab. 2018 Jun 27;31(6):689-692. doi: 10.1515/jpem-2017-0404. PMID: 29672274. <https://pubmed.ncbi.nlm.nih.gov/29672274/>
311. Vos FI, Reitsma S, Adriaensen GFJPM, Fokkens WJ. Eye for an eye: near-fatal outcome of fungal infection in a young, diabetic girl. BMJ Case Rep. 2018 Apr 17;2018:bcr2017223059. doi: 10.1136/bcr-2017-223059. PMID: 29666080; PMCID: PMC5905810. Link <https://pubmed.ncbi.nlm.nih.gov/29666080/>
312. Choe YH, Lee YC, Kim SR. Endobronchial Mucormycosis Successfully Treated with Flexible Bronchoscopic Cryotherapy. Am J Respir Crit Care Med. 2018 Aug 1;198(3):387-389. doi: 10.1164/rccm.201708-1581IM. PMID: 29648882. Link <https://pubmed.ncbi.nlm.nih.gov/29648882/>
313. Wanat-Hawthorne A, Stubblefield J, Lynch I, Dellaria S, Kernstine K. Successful Use of a Double Lumen Endotracheal Tube and Bronchial Blocker for Lung Isolation in Pulmonary Mucormycosis. J Cardiothorac Vasc Anesth. 2019 Mar;33(3):776-780. doi: 10.1053/j.jvca.2018.03.011. Epub 2018 Mar 8. PMID: 29631944. Link <https://pubmed.ncbi.nlm.nih.gov/29631944/>
314. Marsh BM, Rajasingham R, Tawfic SH, Borofsky MS. Successful Conservative Management of Bilateral Renal Mucormycosis. Urology. 2018 Oct;120:2-5. doi: 10.1016/j.urology.2018.03.010. Epub 2018 Mar 27. PMID: 29601838. Link <https://pubmed.ncbi.nlm.nih.gov/29601838/>
315. Marechal E, Barry F, Dalle F, Basmaciyan L, Valot S, Sautour M, Duvillard C, Chavanet P, Piroth L, Blot M. Fatal invasive otitis with skull base osteomyelitis caused by Saksenaea vasiformis. QJM. 2018 Jul 1;111(7):499-500. doi: 10.1093/qjmed/hcy068. PMID: 29590490. Link <https://pubmed.ncbi.nlm.nih.gov/29590490/>
316. Long EB, Patel NC, Sautter RL, Antoszyk J, Dollar JD, Rupar D. Photo Quiz: A 9-Year-Old Girl with Eye Swelling and Drainage. J Clin Microbiol. 2018 Mar 26;56(4):e00463-16. doi: 10.1128/JCM.00463-16. PMID: 29581315; PMCID: PMC5869847. Link <https://pubmed.ncbi.nlm.nih.gov/29581315/>
317. Alfano G, Fontana F, Francesca D, Assirati G, Magistri P, Tarantino G, Ballarin R, Rossi G, Franceschini E, Codeluppi M, Guaraldi G, Mussini C, Di Benedetto F, Cappelli G. Gastric Mucormycosis in a Liver and Kidney Transplant Recipient: Case Report and Concise Review of Literature. Transplant Proc. 2018 Apr;50(3):905-909. doi: 10.1016/j.transproceed.2017.11.036. Epub 2018 Mar 22. PMID: 29573830. Link https://pubmed.ncbi.nlm.nih.gov/29573830/
318. Gupta S, Jayashree M, Chakrabarti A, Sodhi KS, Kanojia RP, Mitra S. Invasive Gastrointestinal Mucormycosis: A Master Masquerader. Pediatr Infect Dis J. 2018 Oct;37(10):1067-1070. doi: 10.1097/INF.0000000000001948. PMID: 29570587. Link <https://pubmed.ncbi.nlm.nih.gov/29570587/>
319. Downie ML, Alghounaim M, Davidge KM, Yau Y, Walsh TJ, Pope E, Somers GR, Waters V, Robinson LA. Isolated cutaneous mucormycosis in a pediatric renal transplant recipient. Pediatr Transplant. 2018 Jun;22(4):e13172. doi: 10.1111/petr.13172. Epub 2018 Mar 23. PMID: 29569805. Link <https://pubmed.ncbi.nlm.nih.gov/29569805/>
320. Mishra S, Shelly D, Gupta D, Bharadwaj R. Invasive cutaneous mucormycosis in a preterm neonate presenting as a vesicobullous lesion. Indian J Pathol Microbiol. 2018 Jan-Mar;61(1):103-105. doi: 10.4103/IJPM.IJPM_796_16. PMID: 29567895. Link <https://pubmed.ncbi.nlm.nih.gov/29567895/>
321. Brettholz AM, Mccauley SO. Mucormycosis: Early Identification of a Deadly Fungus [Formula: see text]. J Pediatr Oncol Nurs. 2018 Jul/Aug;35(4):257-266. doi: 10.1177/1043454218763092. Epub 2018 Mar 21. PMID: 29560764. Link <https://pubmed.ncbi.nlm.nih.gov/29560764/>
322. Bansal R, Duddempudi S, Thelmo W, Rajnish L. A unique case of upper GI bleed. Acta Gastroenterol Belg. 2017 Oct-Dec;80(4):553-554. PMID: 29560659. Link <https://pubmed.ncbi.nlm.nih.gov/29560659/>
323. Chang CF, Huang TY, Lin TY. Unusual giant gastric cardia ulcer. Intern Emerg Med. 2018 Aug;13(5):795-796. doi: 10.1007/s11739-018-1814-1. Epub 2018 Mar 20. PMID: 29557520. Link <https://pubmed.ncbi.nlm.nih.gov/29557520/>
324. Gholinejad Ghadi N, Seifi Z, Shokohi T, Aghili SR, Nikkhah M, Vahedi Larijani L, Ghasemi M, Haghani I. Fulminant mucormycosis of maxillary sinuses after dental extraction inpatients with uncontrolled diabetic: Two case reports. J Mycol Med. 2018 Jun;28(2):399-402. French. doi: 10.1016/j.mycmed.2018.01.003. Epub 2018 Mar 13. PMID: 29545122. Link https://pubmed.ncbi.nlm.nih.gov/29545122/
325. Kaneko Y, Oinuma KI, Terachi T, Arimura Y, Niki M, Yamada K, Kakeya H, Mizutani T. Successful Treatment of Intestinal Mycosis Caused by a Simultaneous Infection with Lichtheimia ramosa and Aspergillus calidoustus. Intern Med. 2018 Aug 15;57(16):2421-2424. doi: 10.2169/internalmedicine.0254-17. Epub 2018 Mar 9. PMID: 29526942; PMCID: PMC6148160. Link <https://pubmed.ncbi.nlm.nih.gov/29526942/>
326. Reich P, Shute T, Lysen C, Lockhart SR, Kelly Keating M, Custer P, Orscheln R. Saksenaea vasiformis Orbital Cellulitis in an Immunocompetent Child Treated With Posaconazole. J Pediatric Infect Dis Soc. 2018 Aug 17;7(3):e169-e171. doi: 10.1093/jpids/piy021. PMID: 29522137. Link <https://pubmed.ncbi.nlm.nih.gov/29522137/>
327. Kabulski GM, MacVane SH. Isavuconazole pharmacokinetics in a patient with cystic fibrosis following bilateral orthotopic lung transplantation. Transpl Infect Dis. 2018 Jun;20(3):e12878. doi: 10.1111/tid.12878. Epub 2018 Mar 25. PMID: 29512930. Link <https://pubmed.ncbi.nlm.nih.gov/29512930/>
328. Zehani A, Smichi I, Marrakchi J, Besbes G, Haouet S, Kchir N. Agressive infection following a dental extraction in a diabetic patient :Rhinocerebral mucormycosis. Tunis Med. 2017 May;95(5):378-380. PMID: 29509222. Link <https://pubmed.ncbi.nlm.nih.gov/29509222/>
329. Shumilov E, Bacher U, Perske C, Mohr A, Eiffert H, Hasenkamp J, Trümper L, Wulf GG, Ströbel P, Ibrahim AS, Venkataramani V. In Situ Validation of the Endothelial Cell Receptor GRP78 in a Case of Rhinocerebral Mucormycosis. Antimicrob Agents Chemother. 2018 Apr 26;62(5):e00172-18. doi: 10.1128/AAC.00172-18. PMID: 29483124; PMCID: PMC5923111. Link <https://pubmed.ncbi.nlm.nih.gov/29483124/>
330. Jundt JS, Wong MEK, Tatara AM, Demian NM. Invasive Cutaneous Facial Mucormycosis in a Trauma Patient. J Oral Maxillofac Surg. 2018 Sep;76(9):1930.e1-1930.e5. doi: 10.1016/j.joms.2018.01.022. Epub 2018 Feb 16. PMID: 29458027. Link <https://pubmed.ncbi.nlm.nih.gov/29458027/>
331. Sayan M, Arpag H, Tokur M, Bahar AY. Pulmonary mucormycosis mimicking an endobronchial mass. Asian Cardiovasc Thorac Ann. 2018 Mar;26(3):242-244. doi: 10.1177/0218492318760711. Epub 2018 Feb 15. PMID: 29448830. Link <https://pubmed.ncbi.nlm.nih.gov/29448830/>
332. Sharma SK, Balasubramanian P, Radotra B, Singhal M. Isolated splenic mucormycosis in a case of aplastic anaemia. BMJ Case Rep. 2018 Feb 8;2018:bcr2017223243. doi: 10.1136/bcr-2017-223243. PMID: 29437814; PMCID: PMC5836678. Link <https://pubmed.ncbi.nlm.nih.gov/29437814/>
333. Mills SEA, Yeldandi AV, Odell DD. Surgical Treatment of Multifocal Pulmonary Mucormycosis. Ann Thorac Surg. 2018 Aug;106(2):e93-e95. doi: 10.1016/j.athoracsur.2017.12.033. Epub 2018 Jan 31. PMID: 29391149; PMCID: PMC6085879. Link <https://pubmed.ncbi.nlm.nih.gov/29391149/>
334. Kumar M, Jantausch B, Torres C, Campos J, Zelazny A. Central Line-Associated Mucor velutinosus Bloodstream Infection in an Immunocompetent Pediatric Patient. J Pediatric Infect Dis Soc. 2018 May 15;7(2):e55-e57. doi: 10.1093/jpids/pix108. PMID: 29373689; PMCID: PMC6251637. Link <https://www.ncbi.nlm.nih.gov/pmc/articles/PMC6251637/>
335. Kumar M, Jantausch B, Torres C, Campos J, Zelazny A. Central Line-Associated Mucor velutinosus Bloodstream Infection in an Immunocompetent Pediatric Patient. J Pediatric Infect Dis Soc. 2018 May 15;7(2):e55-e57. doi: 10.1093/jpids/pix108. PMID: 29373689; PMCID: PMC6251637. Link <https://pubmed.ncbi.nlm.nih.gov/29373689/>
336. Chavez JA, Brat DJ, Hunter SB, Velazquez Vega J, Guarner J. Practical Diagnostic Approach to the Presence of Hyphae in Neuropathology Specimens With Three Illustrative Cases. Am J Clin Pathol. 2018 Jan 29;149(2):98-104. doi: 10.1093/ajcp/aqx144. PMID: 29365030. Link <https://pubmed.ncbi.nlm.nih.gov/29365030/>
337. Gupta N, Kumar A, Singh G, Ratnakar G, Vinod KS, Wig N. Breakthrough mucormycosis after voriconazole use in a case of invasive fungal rhinosinusitis due to Curvularia lunata. Drug Discov Ther. 2017;11(6):349-352. doi: 10.5582/ddt.2017.01060. PMID: 29332895. Link <https://pubmed.ncbi.nlm.nih.gov/29332895/>
338. Al-Sheikhli J, Taqi H, Drake J, Habib A. Rare cause of pulmonary cavitation in a 75-year-old man. BMJ Case Rep. 2018 Jan 10;2018:bcr2017222792. doi: 10.1136/bcr-2017-222792. PMID: 29326340; PMCID: PMC5786963. Link <https://pubmed.ncbi.nlm.nih.gov/29326340/>
339. Lerchbaumer MH, Lohneis P, Baur A. Angioinvasive pulmonale Mukormykose mit Aorteninfiltration. Rofo. 2018 Apr;190(4):365-367. German. doi: 10.1055/s-0043-123940. Epub 2017 Dec 28. PMID: 29284172. Link <https://pubmed.ncbi.nlm.nih.gov/29284172/>
340. Sawardekar KP. Gangrenous Necrotizing Cutaneous Mucormycosis in an Immunocompetent Neonate: A Case Report from Oman. J Trop Pediatr. 2018 Dec 1;64(6):548-552. doi: 10.1093/tropej/fmx094. PMID: 29253258. Link <https://pubmed.ncbi.nlm.nih.gov/29253258/>
341. Kawahara Y, Wada S, Nijima H, Hayase T, Furukawa R, Ashizawa K, Morimoto A. Rhinocerebral Mucormycosis With Temporal Artery Thrombosis in an Adolescent Following HLA-haploidentical Stem Cell Transplantation. J Pediatr Hematol Oncol. 2018 Oct;40(7):e461-e463. doi: 10.1097/MPH.0000000000001020. PMID: 29200154. Link <https://pubmed.ncbi.nlm.nih.gov/29200154/>
342. Kohan R, Delgado T, Zakariya-Yousef Breval I, Arbesú Cruz A. Mucormicosis pulmonar por Cunninghamella spp. en paciente trasplantado renal [Pulmonary mucormycosis due to Cunninghamella spp. in renal transplant patient]. Rev Esp Quimioter. 2017 Dec;30(6):472-473. Spanish. Epub 2017 Nov 27. PMID: 29199417. Link <https://pubmed.ncbi.nlm.nih.gov/29199417/>
343. Nawata T, Kubo M, Kobayashi S, Nakada N, Maeda M, Cui D, Kimura T, Ikeda E, Yano M. Mucormycete Infiltration in the Cardiac Conduction System. Can J Cardiol. 2018 Jan;34(1):92.e9-92.e10. doi: 10.1016/j.cjca.2017.10.002. Epub 2017 Oct 5. PMID: 29195769. <https://pubmed.ncbi.nlm.nih.gov/29195769/>
344. Kuo DJ. Multimodal Treatment of Rhinocerebral Mucormycosis in a Pediatric Patient With Relapsed Pre-B Acute Lymphoblastic Leukemia. Pediatr Infect Dis J. 2018 Jun;37(6):555-558. doi: 10.1097/INF.0000000000001839. PMID: 29189615. Link <https://pubmed.ncbi.nlm.nih.gov/29189615/>
345. Westerling G, Davis M, Khuon D. Do donated linens put patients at risk for fungal infections during hospitalization? A pediatric case investigation and subsequently implemented process changes. Am J Infect Control. 2018 Jan;46(1):118-119. doi: 10.1016/j.ajic.2017.09.021. Epub 2017 Nov 22. PMID: 29174656. Link <https://pubmed.ncbi.nlm.nih.gov/29174656/>
346. Margoles L, DeNofrio D, Patel AR, Golan Y, Vest AR, Arkun K, Boucher HW, Kiernan MS, Upshaw JN. Disseminated mucormycosis masquerading as rejection early after orthotopic heart transplantation. Transpl Infect Dis. 2018 Feb;20(1). doi: 10.1111/tid.12820. Epub 2018 Jan 16. PMID: 29172240. Link <https://pubmed.ncbi.nlm.nih.gov/29172240/>
347. Lu D, Hua C, Servy A, Foulet F, Botterel F, De Prost N, Ellrodt O, Sigha B, Popescu D, Ortonne N, Wolkenstein P, Ingen-Housz-Oro S, Chosidow O. Primary cutaneous mucormycosis as a complication of erosive dermatitis: two cases. Eur J Dermatol. 2018 Apr 1;28(2):227-229. doi: 10.1684/ejd.2017.3212. PMID: 29171398. Link <https://pubmed.ncbi.nlm.nih.gov/29171398/>
348. McNab AA. The 2017 Doyne Lecture: the orbit as a window to systemic disease. Eye (Lond). 2018 Feb;32(2):248-261. doi: 10.1038/eye.2017.224. Epub 2017 Nov 10. PMID: 29125145; PMCID: PMC5811731. Link https://pubmed.ncbi.nlm.nih.gov/29125145/
349. Cheng Y, Gao Y, Liu XY, Wang GY, Zhang GQ, Gao SQ. Rhinocerebral mucormycosis caused by Rhizopus arrhizus var. tonkinensis. J Mycol Med. 2017 Dec;27(4):586-588. French. doi: 10.1016/j.mycmed.2017.10.001. Epub 2017 Nov 7. PMID: 29122529. Link <https://pubmed.ncbi.nlm.nih.gov/29122529/>
350. Hashash JG, Minervini M, Cruz RJ Jr. Black Stoma: What Could It Be? Gastroenterology. 2017 Dec;153(6):e12-e13. doi: 10.1053/j.gastro.2017.04.014. Epub 2017 Nov 4. PMID: 29112828. Link <https://pubmed.ncbi.nlm.nih.gov/29112828/>
351. Dorin J, D'Aveni M, Debourgogne A, Cuenin M, Guillaso M, Rivier A, Gallet P, Lecoanet G, Machouart M. Update on Actinomucor elegans, a mucormycete infrequently detected in human specimens: how combined microbiological tools contribute efficiently to a more accurate medical care. Int J Med Microbiol. 2017 Dec;307(8):435-442. doi: 10.1016/j.ijmm.2017.10.010. Epub 2017 Oct 26. PMID: 29108709. Link <https://pubmed.ncbi.nlm.nih.gov/29108709/>
352. Liang GZ, Xu WQ, Zheng XL, Mei H, Lv GX, Shen YN, Li DM, Liu WD. Successful Treatment by Surgery of a Primary Cutaneous Mucormycosis Caused by Mucor irregularis. Mycopathologia. 2018 Apr;183(2):445-449. doi: 10.1007/s11046-017-0219-4. Epub 2017 Oct 30. PMID: 29086142. Link <https://pubmed.ncbi.nlm.nih.gov/29086142/>
353. Barnes AMT, Crespo-Diaz RJ, Cohenour J, Kirsch JD, Arbefeville S, Ferrieri P. A Noninvasive Rhizopus Infection With a Bladder Fungal Ball in a Patient With Poorly Controlled Diabetes Mellitus. Lab Med. 2017 Dec 22;49(1):75-79. doi: 10.1093/labmed/lmx060. PMID: 29069422. Link <https://pubmed.ncbi.nlm.nih.gov/29069422/>
354. Cheng W, Wang G, Yang M, Sun L, Dong H, Chen Y, Cheng H. Cutaneous mucormycosis in a patient with lupus nephritis: A case report and review of literature. Medicine (Baltimore). 2017 Oct;96(42):e8211. doi: 10.1097/MD.0000000000008211. PMID: 29049207; PMCID: PMC5662373. Link <https://pubmed.ncbi.nlm.nih.gov/29049207/>
355. He R, Hu C, Tang Y, Yang H, Cao L, Niu R. Report of 12 cases with tracheobronchial mucormycosis and a review. Clin Respir J. 2018 Apr;12(4):1651-1660. doi: 10.1111/crj.12724. Epub 2018 Feb 19. PMID: 29028140. Link <https://pubmed.ncbi.nlm.nih.gov/29028140/>
356. Farid S, AbuSaleh O, Liesman R, Sohail MR. Isolated cerebral mucormycosis caused by Rhizomucor pusillus. BMJ Case Rep. 2017 Oct 4;2017:bcr2017221473. doi: 10.1136/bcr-2017-221473. PMID: 28978601; PMCID: PMC5652351. Link <https://pubmed.ncbi.nlm.nih.gov/28978601/>
357. Le Gac G, Allyn J, Coolen-Allou N, Lagrange-Xelot M, Fernandez C, Allou N, Hoarau G. Mucormycose hépatique à Rhizopus microsporus : description d’un cas [Hepatic mucormycosis due to Rhizopus microsporus: A case report]. Med Mal Infect. 2017 Nov;47(7):504-507. French. doi: 10.1016/j.medmal.2017.05.008. Epub 2017 Sep 14. PMID: 28919389. Link <https://pubmed.ncbi.nlm.nih.gov/28919389/>
358. Cheong HS, Kim SY, Ki HK, Kim JY, Lee MH. Oral mucormycosis in patients with haematologic malignancies in a bone marrow transplant unit. Mycoses. 2017 Dec;60(12):836-841. doi: 10.1111/myc.12678. Epub 2017 Sep 6. PMID: 28877386. Link <https://pubmed.ncbi.nlm.nih.gov/28877386/>
359. Hunt WR. Pulmonary Mucormycosis in a Patient With Poorly Controlled Diabetes After a Liver Transplant. Am J Med Sci. 2017 Aug;354(2):e1. doi: 10.1016/j.amjms.2017.03.035. Epub 2017 Apr 8. PMID: 28864385. Link <https://pubmed.ncbi.nlm.nih.gov/28864385/>
360. Metussin D, Telisinghe PU, Chong PL, Chong VH. Gastrointestinal: Gastric mucormycosis. J Gastroenterol Hepatol. 2017 Sep;32(9):1537. doi: 10.1111/jgh.13661. PMID: 28845590. Link <https://pubmed.ncbi.nlm.nih.gov/28845590/>
361. Zafar S, Prabhu A. Rhino-orbito-cerebral mucormycosis: recovery against the odds. Pract Neurol. 2017 Dec;17(6):485-488. doi: 10.1136/practneurol-2017-001671. Epub 2017 Aug 26. PMID: 28844040. Link <https://pubmed.ncbi.nlm.nih.gov/28844040/>
362. Avelar Rodriguez D, Ochoa Virgen G, Miranda Ackerman RC. A tip from the nose: rhinocerebral mucormycosis in a patient with alcoholic liver cirrhosis and cocaine abuse, an uncommon association. BMJ Case Rep. 2017 Aug 7;2017:bcr2017220730. doi: 10.1136/bcr-2017-220730. PMID: 28784893; PMCID: PMC5623276. Link <https://pubmed.ncbi.nlm.nih.gov/28784893/>
363. Navarro Vergara DI, Barragán Pola G, Bonifaz A, Núñez Pérez-Redondo C, Choreño García O, Cicero Sabido R. Mucormicosis pulmonar en un paciente con trasplante renal y hemoptisis incoercible [Pulmonary mucormycosis in a patient with kidney transplant and uncontrolled haemoptysis]. Rev Iberoam Micol. 2017 Oct-Dec;34(4):233-236. Spanish. doi: 10.1016/j.riam.2017.03.005. Epub 2017 Jul 27. PMID: 28757006. Link <https://pubmed.ncbi.nlm.nih.gov/28757006/>
364. Gaut D, Cone BD, Gregson AL, Agopian VG. Gastrointestinal Mucormycosis After Orthotopic Liver Transplantation Presenting as Femoral Nerve Palsy: A Case Report and Review of the Literature. Transplant Proc. 2017 Sep;49(7):1608-1614. doi: 10.1016/j.transproceed.2017.03.074. Epub 2017 Jul 26. PMID: 28755897. Link <https://pubmed.ncbi.nlm.nih.gov/28755897/>
365. Salibi A, McArdle C, Morritt AN. Mucormycosis infection in the upper limb: a salvageable condition. J Hosp Infect. 2017 Dec;97(4):418-419. doi: 10.1016/j.jhin.2017.07.019. Epub 2017 Jul 25. PMID: 28751009. Link <https://pubmed.ncbi.nlm.nih.gov/28751009/>
366. Taj-Aldeen SJ, Almaslamani M, Theelen B, Boekhout T. Phylogenetic analysis reveals two genotypes of the emerging fungus Mucor indicus, an opportunistic human pathogen in immunocompromised patients. Emerg Microbes Infect. 2017 Jul 12;6(7):e63. doi: 10.1038/emi.2017.51. PMID: 28698667; PMCID: PMC5567167. Link <https://pubmed.ncbi.nlm.nih.gov/28698667/>
367. Sorensen EP, Matiz C. Painful necrotic ulcer on the vulva. Cutis. 2017 Jun;99(6):E13-E15. PMID: 28686761. Link <https://pubmed.ncbi.nlm.nih.gov/28686761/>
368. Turnbull A, Chembo CL, Leikis M, Pidgeon G, Arnold L, Hay N, Matheson P. A Case of Pulmonary Mucormycosis in a Renal Transplant Recipient. Nephrology (Carlton). 2017 Aug;22(8):657. doi: 10.1111/nep.12855. PMID: 28685939. Link <https://pubmed.ncbi.nlm.nih.gov/28685939/>
369. Martín LB, Rodríguez MÁM, Mercier N, Lafont MO, Fernández EO, de la Parte AR, Estefanía M. Rhizopus arrhizus Invasive Infection due to Self-Inflicted Scratch Injuries in a Diabetic Patient with Non-ketotic Acidosis. Mycopathologia. 2017 Oct;182(9-10):927-931. doi: 10.1007/s11046-017-0158-0. Epub 2017 Jun 23. PMID: 28646276. Link <https://pubmed.ncbi.nlm.nih.gov/28646276/>
370. Sánchez-Gil J, Guirao-Arrabal E, Parra-García GD, Luzón-García MDP, Fe Bautista-Marín M, Barayobre-Barayobre M, Fontalba-Navas A. Nosocomial Rhinocerebral Mucormycosis: Two Cases with a Temporal Relationship. Mycopathologia. 2017 Oct;182(9-10):933-935. doi: 10.1007/s11046-017-0164-2. Epub 2017 Jun 21. PMID: 28639065. Link <https://pubmed.ncbi.nlm.nih.gov/28639065/>
371. Pilch WT, Kinnear N, Hennessey DB. Saksenaea vasiformis infection in an immunocompetent patient in rural Australia. BMJ Case Rep. 2017 Jun 18;2017:bcr2017220341. doi: 10.1136/bcr-2017-220341. PMID: 28630247; PMCID: PMC5535000. Link <https://pubmed.ncbi.nlm.nih.gov/28630247/>
372. Kokkayil P, Pandey M, Agarwal R, Kale P, Singh G, Xess I. Rhizopus homothallicus Causing Invasive Infections: Series of Three Cases from a Single Centre in North India. Mycopathologia. 2017 Oct;182(9-10):921-926. doi: 10.1007/s11046-017-0153-5. Epub 2017 Jun 16. PMID: 28623532. Link <https://pubmed.ncbi.nlm.nih.gov/28623532/>
373. Compain F, Aït-Ammar N, Botterel F, Gibault L, Le Pimpec Barthes F, Dannaoui E. Fatal Pulmonary Mucormycosis due to Rhizopus homothallicus. Mycopathologia. 2017 Oct;182(9-10):907-913. doi: 10.1007/s11046-017-0151-7. Epub 2017 Jun 3. PMID: 28580534. Link <https://pubmed.ncbi.nlm.nih.gov/28580534/>
374. Ota H, Yamamoto H, Kimura M, Araoka H, Fujii T, Umeyama T, Ohno H, Miyazaki Y, Kaji D, Taya Y, Nishida A, Ishiwata K, Tsuji M, Takagi S, Asano-Mori Y, Yamamoto G, Uchida N, Izutsu K, Masuoka K, Wake A, Yoneyama A, Makino S, Taniguchi S. Successful Treatment of Pulmonary Mucormycosis Caused by Cunninghamella bertholletiae with High-Dose Liposomal Amphotericin B (10 mg/kg/day) Followed by a Lobectomy in Cord Blood Transplant Recipients. Mycopathologia. 2017 Oct;182(9-10):847-853. doi: 10.1007/s11046-017-0149-1. Epub 2017 Jun 2. PMID: 28577122. Link <https://pubmed.ncbi.nlm.nih.gov/28577122/>
375. DeFilippis EM, Cuddy S, Glass C, Priya S, Aghayev A, Mitchell RN, Marty FM, DiCarli MF, Blankstein R. Use of Multimodality Imaging in Diagnosing Invasive Fungal Diseases of the Heart. Circ Cardiovasc Imaging. 2017 Jun;10(6):e006550. doi: 10.1161/CIRCIMAGING.117.006550. PMID: 28576787. Link <https://pubmed.ncbi.nlm.nih.gov/28576787/>
376. Heller SF, Pochettino A, Rivera M, Schiller HJ. Amphotericin-impregnated polymethylmethacrylate beads as treatment for soft tissue mucormycosis. Surgery. 2017 Dec;162(6):1330-1331. doi: 10.1016/j.surg.2017.03.021. Epub 2017 May 24. PMID: 28549519. Link <https://pubmed.ncbi.nlm.nih.gov/28549519/>
377. Habeshian K, Hamdy R, Norton SA. Gray-Violet Plaque in an Immunocompromised Girl. JAMA Dermatol. 2017 Sep 1;153(9):923-924. doi: 10.1001/jamadermatol.2017.1131. PMID: 28538952. Link <https://pubmed.ncbi.nlm.nih.gov/28538952/>
378. Dworsky ZD, Bennett R, Kim JM, Kuo DJ. Severe medication-induced peripheral neuropathy treated with topical doxepin cream in a paediatric patient with leukaemia. BMJ Case Rep. 2017 May 22;2017:bcr2017219900. doi: 10.1136/bcr-2017-219900. PMID: 28536226; PMCID: PMC5753714. Link <https://pubmed.ncbi.nlm.nih.gov/28536226/>
379. Afolayan O, Copeland H, Zaheer S, Wallen JM. Pulmonary Mucormycosis Treated With Lobectomy. Ann Thorac Surg. 2017 Jun;103(6):e531-e533. doi: 10.1016/j.athoracsur.2017.01.102. PMID: 28528060. Link <https://pubmed.ncbi.nlm.nih.gov/28528060/>
380. Lowe CD, Sainato RJ, Stagliano DR, Morgan MM, Green BP. Primary Cutaneous Mucormycosis in an Extremely Preterm Infant Successfully Treated with Liposomal Amphotericin B. Pediatr Dermatol. 2017 May;34(3):e116-e119. doi: 10.1111/pde.13124. PMID: 28523894. Link <https://pubmed.ncbi.nlm.nih.gov/28523894/>
381. Bakshi SS. Image Gallery: Mucormycosis. Br J Dermatol. 2017 May;176(5):e98. doi: 10.1111/bjd.15395. PMID: 28504384. Link <https://pubmed.ncbi.nlm.nih.gov/28504384/>
382. Galván Fernández J, Jiménez Cuenca MI, Molpeceres Martínez I, Álvarez-Quiñones ML. A rare cause of emphysematous infectious gastritis. Rev Esp Enferm Dig. 2017 May;109(5):368. PMID: 28480721. Link <https://pubmed.ncbi.nlm.nih.gov/28480721/>
383. Sahota R, Gambhir R, Anand S, Dixit A. Rhinocerebral Mucormycosis: Report of a Rare Case. Ethiop J Health Sci. 2017 Jan;27(1):85-90. doi: 10.4314/ejhs.v27i1.11. PMID: 28458494; PMCID: PMC5390232. Link <https://pubmed.ncbi.nlm.nih.gov/28458494/>
384. Hirano T, Yamada M, Sato K, Murakami K, Tamai T, Mitsuhashi Y, Tamada T, Sugiura H, Sato N, Saito R, Tominaga J, Watanabe A, Ichinose M. Invasive pulmonary mucormycosis: rare presentation with pulmonary eosinophilia. BMC Pulm Med. 2017 Apr 28;17(1):76. doi: 10.1186/s12890-017-0419-1. PMID: 28454572; PMCID: PMC5410085. Link <https://pubmed.ncbi.nlm.nih.gov/28454572/>
385. Sahuquillo-Torralba A, Calle-Andrino A, Navarro-Mira MÁ, Llavador-Ros M, Botella-Estrada R. Acute Necrotic Plaque in an Immunocompromised Host. Am J Dermatopathol. 2017 May;39(5):e60-e61. doi: 10.1097/DAD.0000000000000471. PMID: 28426490.
386. Sun M, Hou X, Wang X, Chen G, Zhao Y. Gastrointestinal Mucormycosis of the Jejunum in an Immunocompetent Patient: A Case Report. Medicine (Baltimore). 2017 Apr;96(16):e6360. doi: 10.1097/MD.0000000000006360. PMID: 28422828; PMCID: PMC5406044. Link | <https://pubmed.ncbi.nlm.nih.gov/28422828/>
387. Kwok M, Maurice A, Carroll J, Brown J, Lisec C, Francis L, Patel B. Gastrointestinal mucormycosis in an immunocompromised host. ANZ J Surg. 2019 Jan;89(1-2):E26-E27. doi: 10.1111/ans.13955. Epub 2017 Apr 16. PMID: 28419686. Link <https://pubmed.ncbi.nlm.nih.gov/28419686/>
388. Nitinawarat J, Putcharoen O, Chindamporn A, Rerknimitr P. Subcutaneous Saksenaea vasiformis infection presenting as disfiguring facial plaques. Indian J Dermatol Venereol Leprol. 2017 May-Jun;83(3):346-348. doi: 10.4103/ijdvl.IJDVL_637_16. PMID: 28366910. Link <https://pubmed.ncbi.nlm.nih.gov/28366910/>
389. Winstead M, Ozolek J, Nowalk A, Williams J, Vander Lugt M, Lin P. Disseminated Lichtheimia ramosa Infection After Hematopoietic Stem Cell Transplantation in a Child With Chronic Granulomatous Disease. Pediatr Infect Dis J. 2017 Dec;36(12):1222-1224. doi: 10.1097/INF.0000000000001589. PMID: 28333712. <https://pubmed.ncbi.nlm.nih.gov/28333712/>
390. Iyengar S, Chambers CJ, Millsop JW, Fung MA, Sharon VR. Purple patches in an immunocompromised patient: a report of secondary disseminated cutaneous mucormycosis in a man with chronic lymphocytic leukemia. Dermatol Online J. 2017 Mar 15;23(3):13030/qt8cm6m764. PMID: 28329517. Link <https://pubmed.ncbi.nlm.nih.gov/28329517/>
391. Ino K, Nakase K, Nakamura A, Nakamori Y, Sugawara Y, Miyazaki K, Monma F, Fujieda A, Sugimoto Y, Ohishi K, Masuya M, Katayama N. Management of Pulmonary Mucormycosis Based on a Polymerase Chain Reaction (PCR) Diagnosis in Patients with Hematologic Malignancies: A Report of Four Cases. Intern Med. 2017;56(6):707-711. doi: 10.2169/internalmedicine.56.7647. Epub 2017 Mar 17. PMID: 28321075; PMCID: PMC5410485. Link <https://pubmed.ncbi.nlm.nih.gov/28321075/>
392. Lakhdar K, Houari N, Elbouazzaoui A, Ameuraoui T, Boukatta B, Sbai H, Kanjaa N. Une mucormycose faciale compliquant une angiocholite grave: à propos d’un cas [Facial mucormycosis complicating severe angiocholitis: about a case]. Pan Afr Med J. 2016 Dec 21;25:246. French. doi: 10.11604/pamj.2016.25.246.10977. PMID: 28293362; PMCID: PMC5337295. Link <https://pubmed.ncbi.nlm.nih.gov/28293362/>
393. Sahuquillo-Torralba A, Garrido-Jareño M, Llavador-Ros M, Botella-Estrada R. Rapidly progressive frontal necrotic plaque in an immunosuppressed host. Enferm Infecc Microbiol Clin (Engl Ed). 2018 May;36(5):315-316. English, Spanish. doi: 10.1016/j.eimc.2017.01.003. Epub 2017 Feb 23. PMID: 28237434. Link <https://pubmed.ncbi.nlm.nih.gov/28237434/>
394. Bamba S, Konsegré V, Zida A, Sangaré I, Cissé M, Beogo R, Diallo B, Andonaba JB, Guiguemdé RT. Un cas d’entomophthoromycose rhinofaciale en climat tropical soudano-sahélien au Burkina Faso [A case of rhinofacial entomophthoromycosis in Soudano-Sahelian tropical climate in Burkina Faso]. J Mycol Med. 2017 Jun;27(2):254-260. French. doi: 10.1016/j.mycmed.2017.01.002. Epub 2017 Feb 14. PMID: 28214142. Link <https://pubmed.ncbi.nlm.nih.gov/28214142/>
395. Mani RK, Mishra V, Sharma M, Kumar RA. Isolated pulmonary mucormycosis. BMJ Case Rep. 2017 Feb 16;2017:bcr2017219342. doi: 10.1136/bcr-2017-219342. PMID: 28209649; PMCID: PMC5318590. Link <https://pubmed.ncbi.nlm.nih.gov/28209649/>
396. Kreiniz N, Bejar J, Polliack A, Tadmor T. Severe pneumonia associated with ibrutinib monotherapy for CLL and lymphoma. Hematol Oncol. 2018 Feb;36(1):349-354. doi: 10.1002/hon.2387. Epub 2017 Feb 3. PMID: 28156016. Link <https://pubmed.ncbi.nlm.nih.gov/28156016/>
397. Jaffer F, Beatty N, Ahmad K. Mucormycosis pulmonary abscess, containment in a patient with uncontrolled diabetes mellitus. BMJ Case Rep. 2017 Jan 18;2017:bcr2016217945. doi: 10.1136/bcr-2016-217945. PMID: 28100573; PMCID: PMC5256081. Link <https://pubmed.ncbi.nlm.nih.gov/28100573/>
398. Vallverdú Vidal M, Iglesias Moles S, Palomera Fernandez M, Palomar Martinez M. Mucormicosis renal grave en un paciente crítico [Isolated renal mucormycosis in a critically ill patient]. Rev Iberoam Micol. 2017 Jan-Mar;34(1):57-58. Spanish. doi: 10.1016/j.riam.2016.05.002. Epub 2017 Jan 9. PMID: 28081878. Link <https://pubmed.ncbi.nlm.nih.gov/28081878/>
399. Espíldora-Hernández J, Pérez-López C, Abarca-Costalago M, Nuño-Álvarez E. Pulmonary Mucormycosis at Onset of Diabetes in a Young Patient. Arch Bronconeumol. 2017 Sep;53(9):531-533. English, Spanish. doi: 10.1016/j.arbres.2016.11.010. Epub 2017 Jan 4. PMID: 28063611. Link <https://pubmed.ncbi.nlm.nih.gov/28063611/>
400. Delie A, Vlummens P, Creytens D, Steel E. Cutaneous mucormycosis as result of insulin administration in an AML patient: Case report and review of the literature. Acta Clin Belg. 2017 Oct;72(5):352-356. doi: 10.1080/17843286.2016.1266802. Epub 2016 Dec 26. PMID: 28019140. Link <https://pubmed.ncbi.nlm.nih.gov/28019140/>
401. Giudice G, Cutrignelli DA, Sportelli P, Limongelli L, Tempesta A, Gioia GD, Santacroce L, Maiorano E, Favia G. Rhinocerebral Mucormycosis with Orosinusal Involvement: Diagnostic and Surgical Treatment Guidelines. Endocr Metab Immune Disord Drug Targets. 2016;16(4):264-269. doi: 10.2174/1871530316666161223145055. PMID: 28017141.Link <https://pubmed.ncbi.nlm.nih.gov/28017141/>
402. Oliveira FR, Couto NG, Bastos JO, Colleti J Junior, Carvalho WB. Abdominal mucormycosis in a child: a case report. Rev Soc Bras Med Trop. 2016 Nov-Dec;49(6):796-798. doi: 10.1590/0037-8682-0172-2016. PMID: 28001234. Link <https://pubmed.ncbi.nlm.nih.gov/28001234/>
403. Grimaldi D, Pradier O, Hotchkiss RS, Vincent JL. Nivolumab plus interferon-γ in the treatment of intractable mucormycosis. Lancet Infect Dis. 2017 Jan;17(1):18. doi: 10.1016/S1473-3099(16)30541-2. PMID: 27998559. Link <https://pubmed.ncbi.nlm.nih.gov/27998559/>
404. Andrey DO, Kaiser L, Emonet S, Erard V, Chalandon Y, van Delden C. Cerebral Rhizomucor Infection Treated by Posaconazole Delayed-Release Tablets in an Allogeneic Stem Cell Transplant Recipient. Int J Infect Dis. 2017 Feb;55:24-26. doi: 10.1016/j.ijid.2016.12.014. Epub 2016 Dec 14. PMID: 27988409. Link <https://pubmed.ncbi.nlm.nih.gov/27988409/>
405. Benachinmardi KK, Rajalakshmi P, Veenakumari HB, Bharath RD, Vikas V, Mahadevan A, Nagarathna S. Successful treatment of primary cerebral mucormycosis: Role of microbiologist. Indian J Med Microbiol. 2016 Oct-Dec;34(4):550-553. doi: 10.4103/0255-0857.195373. PMID: 27934843. Link <https://pubmed.ncbi.nlm.nih.gov/27934843/>
406. Bhagat M, Rapose A. Rapidly progressing dual infection with Aspergillus and Rhizopus: when soil inhabitants become deadly invaders. BMJ Case Rep. 2016 Dec 8;2016:bcr2016217535. doi: 10.1136/bcr-2016-217535. PMID: 27932434; PMCID: PMC5174834. Link <https://pubmed.ncbi.nlm.nih.gov/27932434/>
407. Farojov R, Aydın O, Yılmaz C, Iakobadze Z, Doğanay L, Camlı D, Demireller A, Küçükgül SC, Kılıç M. Rhino-Orbita-Maxillary Mucormycosis After Liver Transplantation: A Case Report. Transplant Proc. 2016 Nov;48(9):3210-3213. doi: 10.1016/j.transproceed.2016.08.016. PMID: 27932183. Link <https://pubmed.ncbi.nlm.nih.gov/27932183/>
408. Hazama A, Galgano M, Fullmer J, Hall W, Chin L. Affinity of Mucormycosis for Basal Ganglia in Intravenous Drug Users: Case Illustration and Review of Literature. World Neurosurg. 2017 Feb;98:872.e1-872.e3. doi: 10.1016/j.wneu.2016.11.130. Epub 2016 Dec 5. PMID: 27923750. Link <https://pubmed.ncbi.nlm.nih.gov/27923750/>
409. Singla V, Prabhakar N, Singh T, Khandelwal N, Singh G, Vasishta R. Primary Mucormycosis of the Breast: A Rare Entity. Breast J. 2017 Mar;23(2):231-233. doi: 10.1111/tbj.12716. Epub 2016 Nov 25. PMID: 27886415. Link <https://pubmed.ncbi.nlm.nih.gov/27886415/>
410. Razmi T M, Shivaprakash MR, Saikia UN, De D, Handa S. "All That Necroses Is Not Toxic Epidermal Necrolysis". J Cutan Med Surg. 2017 Mar/Apr;21(2):172-173. doi: 10.1177/1203475416679827. Epub 2016 Nov 23. PMID: 27879397. Link <https://pubmed.ncbi.nlm.nih.gov/27879397/>
411. McSpadden RP, Martin JR, Mehrotra S, Thorpe E. Mucormycosis Causing Ludwig Angina: A Unique Presentation. J Oral Maxillofac Surg. 2017 Apr;75(4):759-762. doi: 10.1016/j.joms.2016.10.025. Epub 2016 Oct 29. PMID: 27875707. Link <https://pubmed.ncbi.nlm.nih.gov/27875707/>
412. Benites BM, Fonseca FP, Parahyba CJ, Arap SS, Novis YA, Fregnani ER. Extensive Oral Mucormycosis in a Transplanted Patient. J Craniofac Surg. 2017 Jan;28(1):e4-e5. doi: 10.1097/SCS.0000000000003152. PMID: 27875508. Link <https://pubmed.ncbi.nlm.nih.gov/27875508/>
413. Kothari A, Shalin SC, Crescencio JC, Burgess MJ. Skin lesion in a patient with acute myeloid leukemia. Transpl Infect Dis. 2017 Feb;19(1). doi: 10.1111/tid.12635. Epub 2016 Dec 28. PMID: 27862750. Link <https://pubmed.ncbi.nlm.nih.gov/27862750/>
414. Jensen TSR, Arendrup MC, von Buchvald C, Frandsen TL, Juhler M, Nygaard U. Successful Treatment of Rhino-Orbital-Cerebral Mucormycosis in a Child With Leukemia. J Pediatr Hematol Oncol. 2017 May;39(4):e211-e215. doi: 10.1097/MPH.0000000000000701. PMID: 27841827. Link <https://pubmed.ncbi.nlm.nih.gov/27841827/>
415. Lorenzo de la Peña L, Martín González C, Marca Almeida L. Gastrointestinal mucormycosis. Case report. Med Clin (Barc). 2016 Dec 16;147(12):e69-e70. English, Spanish. doi: 10.1016/j.medcli.2016.09.029. Epub 2016 Nov 3. PMID: 27817940. Link <https://pubmed.ncbi.nlm.nih.gov/27817940/>
416. Papastavros V, Nathoo R, Potter KA, Gonzalez Santiago T. A refractory ulcer in an immunocompromised patient: what caused it? Int J Dermatol. 2017 Jun;56(6):597-598. doi: 10.1111/ijd.13422. Epub 2016 Nov 4. PMID: 27813132. Link <https://pubmed.ncbi.nlm.nih.gov/27813132/>
417. Rodríguez-Lobato E, Ramírez-Hobak L, Aquino-Matus JE, Ramírez-Hinojosa JP, Lozano-Fernández VH, Xicohtencatl-Cortes J, Hernández-Castro R, Arenas R. Primary Cutaneous Mucormycosis Caused by Rhizopus oryzae: A Case Report and Review of Literature. Mycopathologia. 2017 Apr;182(3-4):387-392. doi: 10.1007/s11046-016-0084-6. Epub 2016 Nov 3. PMID: 27807669. Link <https://pubmed.ncbi.nlm.nih.gov/27807669/>
418. Ferreira D, Davies A, Thiruchelvam T, Wark P. Acute myocardial infarction in disseminated mucormycosis infection. Eur Heart J. 2017 Mar 14;38(11):838. doi: 10.1093/eurheartj/ehw517. PMID: 27807051. Link <https://pubmed.ncbi.nlm.nih.gov/27807051/>
419. Mattingly JK, Ramakrishnan VR. Rhinocerebral Mucormycosis of the Optic Nerve. Otolaryngol Head Neck Surg. 2016 Nov;155(5):888-889. doi: 10.1177/0194599816658024. Epub 2016 Jun 28. PMID: 27352893. Link <https://pubmed.ncbi.nlm.nih.gov/27352893/>
420. Zahoor BA, Piercey JE, Wall DR, Tetsworth KD. A surgical approach in the management of mucormycosis in a trauma patient. Ann R Coll Surg Engl. 2016 Nov;98(8):e173-e177. doi: 10.1308/rcsann.2016.0221. Epub 2016 Aug 23. PMID: 27551903; PMCID: PMC5392881. Link Zahoor BA, Piercey JE, Wall DR, Tetsworth KD. A surgical approach in the management of mucormycosis in a trauma patient. Ann R Coll Surg Engl. 2016 Nov;98(8):e173-e177. doi: 10.1308/rcsann.2016.0221. Epub 2016 Aug 23. PMID: 27551903; PMCID: PMC5392881.
421. Hirabayashi KE, Kalin-Hajdu E, Brodie FL, Kersten RC, Russell MS, Vagefi MR. Retrobulbar Injection of Amphotericin B for Orbital Mucormycosis. Ophthalmic Plast Reconstr Surg. 2017 Jul/Aug;33(4):e94-e97. doi: 10.1097/IOP.0000000000000806. PMID: 27768642. Link <https://pubmed.ncbi.nlm.nih.gov/27768642/>
422. Seo YM, Hwang-Bo S, Kim SK, Han SB, Chung NG, Kang JH. Fatal systemic adenoviral infection superimposed on pulmonary mucormycosis in a child with acute leukemia: A case report. Medicine (Baltimore). 2016 Oct;95(40):e5054. doi: 10.1097/MD.0000000000005054. PMID: 27749571; PMCID: PMC5059074. Link <https://pubmed.ncbi.nlm.nih.gov/27749571/>
423. Vogt N, Heß K, Bialek R, Buerke B, Brüggemann M, Topp MS, Groth C, Berdel WE, Lenz G, Stelljes M. Epileptic seizures and rhinocerebral mucormycosis during blinatumomab treatment in a patient with biphenotypic acute leukemia. Ann Hematol. 2017 Jan;96(1):151-153. doi: 10.1007/s00277-016-2837-1. Epub 2016 Oct 3. PMID: 27696204. Link <https://pubmed.ncbi.nlm.nih.gov/27696204/>
424. Stanistreet B, Bell D. Burn Wound Mucormycosis: A Case Study on Poor Wound Healing. J Burn Care Res. 2017 Mar/Apr;38(2):e582-e584. doi: 10.1097/BCR.0000000000000430. PMID: 27617406. Link <https://pubmed.ncbi.nlm.nih.gov/27617406/>
425. Yacoub A, Soni KK, Mojica L, Mai J, Morano J, Cruse CW, Sandin RL, Nanjappa S, Bohra C, Gajanan G, Greene JN. Primary Gangrenous Cutaneous Mold Infections in a Patient With Cancer and Neutropenia. Cancer Control. 2016 Jul;23(3):265-71. doi: 10.1177/107327481602300309. PMID: 27556666. Link <https://pubmed.ncbi.nlm.nih.gov/27556666/>
426. Lee SW, Lee HS. [Gastric Mucormycosis Followed by Traumatic Cardiac Rupture in an Immunocompetent Patient]. Korean J Gastroenterol. 2016 Aug 25;68(2):99-103. Korean. doi: 10.4166/kjg.2016.68.2.99. PMID: 27554217. Link <https://pubmed.ncbi.nlm.nih.gov/27554217/>
427. Paduraru M, Moreno-Sanz C, Olalla Gallardo JM. Primary cutaneous mucormycosis in an immunocompetent patient. BMJ Case Rep. 2016 Aug 16;2016:bcr2016214982. doi: 10.1136/bcr-2016-214982. PMID: 27530872; PMCID: PMC5015179. Link <https://pubmed.ncbi.nlm.nih.gov/27530872/>
428. El Hachem G, Chamseddine N, Saidy G, Choueiry C, Afif C. Successful Nonsurgical Eradication of Invasive Gastric Mucormycosis. Clin Lymphoma Myeloma Leuk. 2016 Aug;16 Suppl:S145-8. doi: 10.1016/j.clml.2016.02.020. PMID: 27521312. Link <https://pubmed.ncbi.nlm.nih.gov/27521312/>
429. Moreira J, Ridolfi F, Almeida-Paes R, Varon A, Lamas CC. Cutaneous mucormycosis in advanced HIV disease. Braz J Infect Dis. 2016 Nov-Dec;20(6):637-640. doi: 10.1016/j.bjid.2016.06.004. Epub 2016 Jul 26. PMID: 27473891. Link <https://pubmed.ncbi.nlm.nih.gov/27473891/>
430. Wang Q, Liu B, Yan Y. Disseminated mucormycosis (DM) after pneumonectomy: a case report. BMC Infect Dis. 2016 Jul 22;16:337. doi: 10.1186/s12879-016-1639-3. PMID: 27450424; PMCID: PMC4957381. Link <https://pubmed.ncbi.nlm.nih.gov/27450424/>
431. Mouronte-Roibás C, Leiro-Fernández V, Botana-Rial M, Ramos-Hernández C, Lago-Preciado G, Fiaño-Valverde C, Fernández-Villar A. Lichtheimia ramosa: A Fatal Case of Mucormycosis. Can Respir J. 2016;2016:2178218. doi: 10.1155/2016/2178218. Epub 2016 Mar 29. PMID: 27445521; PMCID: PMC4904553. Link <https://pubmed.ncbi.nlm.nih.gov/27445521/>
432. Smith SV, Amram AL, Rodarte EM, Lee AG. Neuro-Ophthalmology Cases for the Neurologist. Neurol Clin. 2016 Aug;34(3):611-29. doi: 10.1016/j.ncl.2016.04.005. PMID: 27445244. Link <https://pubmed.ncbi.nlm.nih.gov/27445244/>
433. Martinez-Mugica C, Alba SR, Boga JA, Rodriguez-Guardado A. Cutaneous infection due to Mucor irregularis (Rhizomucor variabilis) in a immunocompetent traveller. Enferm Infecc Microbiol Clin. 2017 Jan;35(1):56-57. doi: 10.1016/j.eimc.2016.06.008. Epub 2016 Jul 18. PMID: 27435037. Link <https://pubmed.ncbi.nlm.nih.gov/27435037/>
434. Lee JH, Hyun JS, Kang DY, Lee HJ, Park SG. Rare complication of bronchoesophageal fistula due to pulmonary mucormycosis after induction chemotherapy for acute myeloid leukemia: a case report. J Med Case Rep. 2016 Jul 16;10:195. doi: 10.1186/s13256-016-0991-7. PMID: 27423701; PMCID: PMC4947348. Link <https://pubmed.ncbi.nlm.nih.gov/27423701/>
435. Bernardo RM, Gurung A, Jain D, Malinis MF. Therapeutic Challenges of Hepatic Mucormycosis in Hematologic Malignancy: A Case Report and Review of the Literature. Am J Case Rep. 2016 Jul 13;17:484-9. doi: 10.12659/ajcr.898480. PMID: 27406045; PMCID: PMC4948661. Link <https://pubmed.ncbi.nlm.nih.gov/27406045/>
436. Al-Tarrah K, Abdelaty M, Behbahani A, Mokaddas E, Soliman H, Albader A. Cutaneous mucormycosis postcosmetic surgery: A case report and review of the literature. Medicine (Baltimore). 2016 Jul;95(27):e4185. doi: 10.1097/MD.0000000000004185. PMID: 27399143; PMCID: PMC5058872. Link <https://pubmed.ncbi.nlm.nih.gov/27399143/>
437. Kim JH, Benefield RJ, Ditolla K. Utilization of posaconazole oral suspension or delayed-released tablet salvage treatment for invasive fungal infection. Mycoses. 2016 Nov;59(11):726-733. doi: 10.1111/myc.12524. PMID: 27392814. Link <https://pubmed.ncbi.nlm.nih.gov/27392814/>
438. Bhattacharya JB, Kaushal S, Aggarwal SK. Esophageal mucormycosis in an immunocompetent child: A rare presentation. Biomed J. 2016 Apr;39(2):155-7. doi: 10.1016/j.bj.2015.11.001. Epub 2016 Jun 20. PMID: 27372172; PMCID: PMC6140314. Link <https://pubmed.ncbi.nlm.nih.gov/27372172/>
439. Luo C, Wang J, Hu Y, Luo Y, Tan Y, Jin A, Wei B, Hu H, Huang H. Cunninghamella bertholletiae Infection in a HLA-Haploidentical Hematopoietic Stem Cell Transplant Recipient with Graft Failure: Case Report and Review of the Literature. Mycopathologia. 2016 Oct;181(9-10):753-8. doi: 10.1007/s11046-016-0030-7. Epub 2016 Jun 28. PMID: 27350325. Link <https://pubmed.ncbi.nlm.nih.gov/27350325/>
440. Chermetz M, Gobbo M, Rupel K, Ottaviani G, Tirelli G, Bussani R, Luzzati R, Di Lenarda R, Biasotto M. Combined Orofacial Aspergillosis and Mucormycosis: Fatal Complication of a Recurrent Paediatric Glioma-Case Report and Review of Literature. Mycopathologia. 2016 Oct;181(9-10):723-33. doi: 10.1007/s11046-016-0021-8. Epub 2016 Jun 27. PMID: 27350324. Link <https://pubmed.ncbi.nlm.nih.gov/27350324/>
441. Tathe SP, Dani AA, Chawhan SM, Meshram SA, Randale AA, Raut WK. Gastric mucormycosis: Diagnosis by imprint cytology. Diagn Cytopathol. 2016 Oct;44(10):820-2. doi: 10.1002/dc.23518. Epub 2016 Jun 20. PMID: 27321416. Link <https://pubmed.ncbi.nlm.nih.gov/27321416/>
442. Katsantonis NG, Hunter JB, O'Connell BP, He J, Lewis JS Jr, Wanna GB. Temporal Bone Mucormycosis. Ann Otol Rhinol Laryngol. 2016 Oct;125(10):850-3. doi: 10.1177/0003489416654711. Epub 2016 Jun 16. PMID: 27317314. Link <https://pubmed.ncbi.nlm.nih.gov/27317314/>
443. Huang YT, Liao CH, Hsueh PR. Image Gallery: Cutaneous infections caused by Alternaria alternata and Mucor irregularis 1 year apart in a patient with iatrogenic Cushing syndrome. Br J Dermatol. 2016 Jun;174(6):e82. doi: 10.1111/bjd.14595. PMID: 27317292. Link <https://pubmed.ncbi.nlm.nih.gov/27317292/>
444. Rodríguez JY, Rodríguez GJ, Morales-López SE, Cantillo CE, Le Pape P, Álvarez-Moreno CA. Saksenaea erythrospora infection after medical tourism for esthetic breast augmentation surgery. Int J Infect Dis. 2016 Aug;49:107-10. doi: 10.1016/j.ijid.2016.05.032. Epub 2016 Jun 4. PMID: 27267577. Link <https://pubmed.ncbi.nlm.nih.gov/27267577/>
445. Mulki R, Masab M, Eiger G, Perloff S. Lethargy and vision loss: successful management of rhinocerebral mucormycosis. BMJ Case Rep. 2016 Jun 2;2016:bcr2016215855. doi: 10.1136/bcr-2016-215855. PMID: 27256997; PMCID: PMC4904383. Link <https://pubmed.ncbi.nlm.nih.gov/27256997/>
446. Wolkow N, Jakobiec FA, Stagner AM, Cunnane ME, Piantadosi AL, Basgoz N, Lefebvre D. Chronic orbital and calvarial fungal infection with Apophysomyces variabilis in an immunocompetent patient. Surv Ophthalmol. 2017 Jan-Feb;62(1):70-82. doi: 10.1016/j.survophthal.2016.05.006. Epub 2016 May 30. PMID: 27256687. Link <https://pubmed.ncbi.nlm.nih.gov/27256687/>
447. Kataria SP, Sharma J, Singh G, Kumar S, Malik S, Kumar V. Primary breast mucormycosis: FNAC diagnosis of a rare entity. Diagn Cytopathol. 2016 Sep;44(9):761-3. doi: 10.1002/dc.23510. Epub 2016 May 24. PMID: 27218441. Link <https://pubmed.ncbi.nlm.nih.gov/27218441/>
448. Vallverdú Vidal M, Iglesias Moles S, Palomar Martínez M. Rhino-orbital-cerebral mucormycosis in a critically ill patient. Med Intensiva. 2017 Nov;41(8):509-510. English, Spanish. doi: 10.1016/j.medin.2016.03.001. Epub 2016 May 17. PMID: 27207730. Link <https://pubmed.ncbi.nlm.nih.gov/27207730/>
449. Mengji AK, Yaga US, Gollamudi N, Prakash B, Rajashekar E. Mucormycosis in a surgical defect masquerading as osteomyelitis: a case report and review of literature. Pan Afr Med J. 2016 Jan 26;23:16. doi: 10.11604/pamj.2016.23.16.8394. PMID: 27200123; PMCID: PMC4856500. Link <https://pubmed.ncbi.nlm.nih.gov/27200123/>
450. Zhang Y, Wang T, Liu GL, Li J, Gao SQ, Wan L. Mucormycosis or extranodal natural killer/T cell lymphoma, similar symptoms but different diagnosis. J Mycol Med. 2016 Sep;26(3):277-82. doi: 10.1016/j.mycmed.2016.04.005. Epub 2016 May 10. PMID: 27178138. Link <https://pubmed.ncbi.nlm.nih.gov/27178138/>
451. Iyengar S, Awasthi S, Fazel N, Okman J, Kamangar F, Sharon VR. Acral bullae in an infant. Arch Dis Child. 2016 Jul;101(7):603. doi: 10.1136/archdischild-2016-310535. Epub 2016 May 5. PMID: 27150267. Link <https://pubmed.ncbi.nlm.nih.gov/27150267/>
452. Al-Otaibi AM, Al-Shahrani DA, Al-Idrissi EM, Al-Abdely HM. Invasive mucormycosis in chronic granulomatous disease. Saudi Med J. 2016 May;37(5):567-9. doi: 10.15537/smj.2016.5.14239. PMID: 27146621; PMCID: PMC4880658. Link <https://pubmed.ncbi.nlm.nih.gov/27146621/>
453. Bakshi SS. Rhino-orbital mucormycosis in a patient with diabetes. Lancet Diabetes Endocrinol. 2017 Mar;5(3):234. doi: 10.1016/S2213-8587(15)00522-7. Epub 2016 Apr 29. PMID: 27138733. Link <https://pubmed.ncbi.nlm.nih.gov/27138733/>
454. Zimmerli S, Bialek R, Blau IW, Christe A, Lass-Flörl C, Presterl E. Lichtheimia Infection in a Lymphoma Patient: Case Report and a Brief Review of the Available Diagnostic Tools. Mycopathologia. 2016 Aug;181(7-8):561-6. doi: 10.1007/s11046-016-0010-y. Epub 2016 Apr 26. PMID: 27115610. Link <https://pubmed.ncbi.nlm.nih.gov/27115610/>
455. Sánchez Velázquez P, Pera M, Gimeno J, Zapatero A, Nolla J, Pera M. Mucormycosis: an unusual cause of gastric perforation and severe bleeding in immunocompetent patients. Rev Esp Enferm Dig. 2017 Mar;109(3):223-225. doi: 10.17235/reed.2016.4269/2016. PMID: 27088595. Link <https://pubmed.ncbi.nlm.nih.gov/27088595/>
456. Afolayan O, Copeland H, Hargrove R, Zaheer S, Wallen JM. Successful Treatment of Invasive Pulmonary Mucormycosis in an Immunocompromised Patient. Ann Thorac Surg. 2016 Apr;101(4):e117-9. doi: 10.1016/j.athoracsur.2015.09.098. PMID: 27000614. Link <https://pubmed.ncbi.nlm.nih.gov/27000614/>
457. Epstein JB, Kupferman SB, Zabner R, Rejali A, Hopp ML, Lill M, Tzachanis D. Early diagnosis and successful management of oral mucormycosis in a hematopoietic stem cell transplant recipient: case report and literature review. Support Care Cancer. 2016 Aug;24(8):3343-6. doi: 10.1007/s00520-016-3170-x. Epub 2016 Mar 14. PMID: 26971955. Link <https://pubmed.ncbi.nlm.nih.gov/26971955/>
458. Ahmed Y, Delaney S, Markarian A. Successful Isavuconazole therapy in a patient with acute invasive fungal rhinosinusitis and acquired immune deficiency syndrome. Am J Otolaryngol. 2016 Mar-Apr;37(2):152-5. doi: 10.1016/j.amjoto.2015.12.003. Epub 2015 Dec 9. PMID: 26954873. <https://pubmed.ncbi.nlm.nih.gov/26954873/>
459. Durila M, Pavlicek P, Hadacova I, Nahlovsky J, Janeckova D. Endogenous Heparinoids May Cause Bleeding in Mucor Infection and can be Detected by Nonactivated Thromboelastometry and Treated by Recombinant Activated Factor VII: A Case Report. Medicine (Baltimore). 2016 Feb;95(8):e2933. doi: 10.1097/MD.0000000000002933. PMID: 26937941; PMCID: PMC4779038. Link <https://pubmed.ncbi.nlm.nih.gov/26937941/>
460. Sahuquillo-Torralba A, Calle-Andrino A, Navarro-Mira MÁ, Llavador-Ros M, Botella-Estrada R. Acute Necrotic Plaque in an Immunocompromised Host. Am J Dermatopathol. 2017 May;39(5):369. doi: 10.1097/DAD.0000000000000470. PMID: 26909588. <https://pubmed.ncbi.nlm.nih.gov/26909588/>
461. Kang JW, Kim JH. Dark necrotic mucosa in sinonasal mucormycosis. Br J Hosp Med (Lond). 2016 Jan;77(1):51. doi: 10.12968/hmed.2016.77.1.51. PMID: 26903460. Link <https://pubmed.ncbi.nlm.nih.gov/26903460/>
462. Bakshi SS. Mucormicosis rinoorbital [Rhino-orbital mucormycosis]. Med Clin (Barc). 2016 Jul 1;147(1):48. Spanish. doi: 10.1016/j.medcli.2015.11.042. Epub 2016 Feb 17. PMID: 26897505. Link <https://pubmed.ncbi.nlm.nih.gov/26897505/>
463. Terry AR, Kahle KT, Larvie M, Vyas JM, Stemmer-Rachamimov A. CASE RECORDS of the MASSACHUSETTS GENERAL HOSPITAL. Case 5-2016. A 43-Year-Old Man with Altered Mental Status and a History of Alcohol Use. N Engl J Med. 2016 Feb 18;374(7):671-80. doi: 10.1056/NEJMcpc1509361. PMID: 26886525. Link <https://pubmed.ncbi.nlm.nih.gov/26886525/>
464. Zhou M, Farooq AV, Andreoli MT, Ali M, Traish AS. Bilateral Rhizopus keratitis in a cocaine user. Can J Ophthalmol. 2016 Feb;51(1):e21-3. doi: 10.1016/j.jcjo.2015.09.018. PMID: 26874166. Link <https://pubmed.ncbi.nlm.nih.gov/26874166/>
465. Deyo JC, Nicolsen N, Lachiewicz A, Kozlowski T. Salvage Treatment of Mucormycosis Post-Liver Transplant With Posaconazole During Sirolimus Maintenance Immunosuppression. J Pharm Pract. 2017 Apr;30(2):261-265. doi: 10.1177/0897190016628702. Epub 2016 Jul 8. PMID: 26864621. Link <https://pubmed.ncbi.nlm.nih.gov/26864621/>
466. Lagenaite-Desmaizières CF, Douma I, Agard E, El Chehab H, Ract-Madoux G, Dot C. Une ophtalmoplégie exceptionnelle, à propos d'un cas de mucormycose [A rare cause of ophthalmoplegia, report of a case of mycormycosis]. J Fr Ophtalmol. 2016 Feb;39(2):e21-3. French. doi: 10.1016/j.jfo.2014.12.006. Epub 2016 Jan 29. PMID: 26832318. Link <https://pubmed.ncbi.nlm.nih.gov/26832318/>
467. Paquette C, Slater SE, McMahon MD, Quddus MR. Cokeromyces recurvatus in a cervical papanicolaou test: A case report of a rare fungus with a brief review of the literature. Diagn Cytopathol. 2016 May;44(5):419-21. doi: 10.1002/dc.23432. Epub 2016 Jan 22. PMID: 26800441. Link <https://pubmed.ncbi.nlm.nih.gov/26800441/>
468. Shigemura T, Nishina S, Nakazawa H, Matsuda K, Yaguchi T, Nakazawa Y. Early detection of Rhizopus DNA in the serum of a patient with rhino-orbital-cerebral mucormycosis following allogeneic hematopoietic stem cell transplantation. Int J Hematol. 2016 Mar;103(3):354-5. doi: 10.1007/s12185-016-1938-x. Epub 2016 Jan 18. PMID: 26781616. Link <https://pubmed.ncbi.nlm.nih.gov/26781616/>
469. Torres-Damas W, Yumpo-Cárdenas D, Mota-Anaya E. Coinfección de mucormicosis rinocerebral y aspergilosis sinusal [Coinfection of rhinocerebral mucormycosis and sinus aspergillosis]. Rev Peru Med Exp Salud Publica. 2015 Oct;32(4):813-7. Spanish. PMID: 26732934. Link <https://pubmed.ncbi.nlm.nih.gov/26732934/>
470. Srivastava N, Bansal V, Kantoor P. Palatal Mucormycosis in An Infant. J Dent Child (Chic). 2015 Sep-Dec;82(3):153-6. PMID: 26731251. Link <https://pubmed.ncbi.nlm.nih.gov/26731251/>
471. Beatty N, Al Mohajer M. Primary cutaneous mucormycosis developing after incision and drainage of a subcutaneous abscess in an immunocompetent host. BMJ Case Rep. 2016 Jan 4;2016:bcr2015213700. doi: 10.1136/bcr-2015-213700. PMID: 26729834; PMCID: PMC4716362. Link <https://pubmed.ncbi.nlm.nih.gov/26729834/>
472. Higo T, Kobayashi T, Yamazaki S, Ando S, Gonoi W, Ishida M, Okuma H, Nakamura F, Ushiku T, Ohtomo K, Fukayama M, Kurokawa M. Cerebral embolism through hematogenous dissemination of pulmonary mucormycosis complicating relapsed leukemia. Int J Clin Exp Pathol. 2015 Oct 1;8(10):13639-42. PMID: 26722589; PMCID: PMC4680534. Link <https://pubmed.ncbi.nlm.nih.gov/26722589/>
473. Azar MM, Assi R, Patel N, Malinis MF. Fungal Mycotic Aneurysm of the Internal Carotid Artery Associated with Sphenoid Sinusitis in an Immunocompromised Patient: A Case Report and Review of the Literature. Mycopathologia. 2016 Jun;181(5-6):425-33. doi: 10.1007/s11046-015-9975-1. Epub 2015 Dec 19. PMID: 26687073. Link <https://pubmed.ncbi.nlm.nih.gov/26687073/>
474. Tisi MC, Giustiniani MC, D'Alò F, Sica S, Hohaus S, Pagano L. A T cell lymphoblastic lymphoma with mucormycosis as unusual etiology of acute cerebral ischemia. Ann Hematol. 2016 Feb;95(3):517-8. doi: 10.1007/s00277-015-2576-8. Epub 2015 Dec 14. PMID: 26666534. Link <https://pubmed.ncbi.nlm.nih.gov/26666534/>
475. Suzuki D, Kobayashi R, Hori D, Kishimoto K, Sano H, Yasuda K, Kobayashi K. Stem cell transplantation for acute myeloid leukemia with pulmonary and cerebral mucormycosis. Pediatr Int. 2016 Jul;58(7):569-72. doi: 10.1111/ped.12866. Epub 2016 Feb 19. PMID: 26645867. Link <https://pubmed.ncbi.nlm.nih.gov/26645867/>
476. Plewes K, Maude RJ, Ghose A, Dondorp AM. Severe falciparum malaria complicated by prolonged haemolysis and rhinomaxillary mucormycosis after parasite clearance: a case report. BMC Infect Dis. 2015 Dec 3;15:555. doi: 10.1186/s12879-015-1285-1. PMID: 26634340; PMCID: PMC4669602. Link <https://pubmed.ncbi.nlm.nih.gov/26634340/>
477. Berdai MA, Labib S, Harandou M. Mucormycose rhinocérébrale compliquant une acidocétose diabétique [Rhinocerebral mucormycosis complicating ketoacidosis diabetes]. Presse Med. 2016 Jan;45(1):145-6. French. doi: 10.1016/j.lpm.2015.11.002. Epub 2015 Nov 26. PMID: 26632090. Link <https://pubmed.ncbi.nlm.nih.gov/26632090/>
478. Garinet S, Tourret J, Barete S, Arzouk N, Meyer I, Frances C, Datry A, Mazier D, Barrou B, Fekkar A. Invasive cutaneous Neoscytalidium infections in renal transplant recipients: a series of five cases. BMC Infect Dis. 2015 Nov 19;15:535. doi: 10.1186/s12879-015-1241-0. PMID: 26586129; PMCID: PMC4653896. Link <https://pubmed.ncbi.nlm.nih.gov/26586129/>
479. Kumar NS, Padala RK, Tirupati S, Tatikonda AK. Rhinocerebral Mucormycosis with Top of Basilar Artery Syndrome. J Stroke Cerebrovasc Dis. 2016 Feb;25(2):378-82. doi: 10.1016/j.jstrokecerebrovasdis.2015.10.009. Epub 2015 Nov 14. PMID: 26584534. Link <https://pubmed.ncbi.nlm.nih.gov/26584534/>
480. Shelburne SA, Ajami NJ, Chibucos MC, Beird HC, Tarrand J, Galloway-Peña J, Albert N, Chemaly RF, Ghantoji SS, Marsh L, Pemmaraju N, Andreeff M, Shpall EJ, Wargo JA, Rezvani K, Alousi A, Bruno VM, Futreal PA, Petrosino JF, Kontoyiannis DP. Implementation of a Pan-Genomic Approach to Investigate Holobiont-Infecting Microbe Interaction: A Case Report of a Leukemic Patient with Invasive Mucormycosis. PLoS One. 2015 Nov 10;10(11):e0139851. doi: 10.1371/journal.pone.0139851. PMID: 26556047; PMCID: PMC4640583. Link <https://pubmed.ncbi.nlm.nih.gov/26556047/>
481. Dayan D, Abu-Abeid S, Klausner JM, Sagie B. Disseminated mucormycosis-induced perforated intestine in a late presenting AIDS patient with steroid-dependent secondary hemophagocytic lymphohistiocytosis. AIDS. 2015 Oct 23;29(16):2216-7. doi: 10.1097/QAD.0000000000000849. PMID: 26544587. Link <https://pubmed.ncbi.nlm.nih.gov/26544587/>
482. Malhotra KP, Shukla S, Singhal A. Fatal fungal nephropathy in an immunocompetent host: an interesting case. BMJ Case Rep. 2015 Nov 4;2015:bcr2015212244. doi: 10.1136/bcr-2015-212244. PMID: 26538130; PMCID: PMC4654206. Link <https://pubmed.ncbi.nlm.nih.gov/26538130/>
483. Nam Y, Jung J, Park SS, Kim SJ, Shin SJ, Choi JH, Kim M, Yoon HE. Disseminated mucormycosis with myocardial involvement in a renal transplant recipient. Transpl Infect Dis. 2015 Dec;17(6):890-6. doi: 10.1111/tid.12452. Epub 2015 Nov 5. PMID: 26538076. Link <https://pubmed.ncbi.nlm.nih.gov/26538076/>
484. Zhang D, Wang X, Lv J, Dong Y. Treatment of a patient with severe hemorrhagic fever accompanied by infection with methicillinresistant Staphylococcus aureus, Acinetobacter baumannii, aspergillus and mucor: a case report. Int J Clin Pharmacol Ther. 2015 Dec;53(12):1028-34. doi: 10.5414/CP202395. PMID: 26521926. Link <https://pubmed.ncbi.nlm.nih.gov/26521926/>
485. Leclercq A, Cinotti E, Labeille B, Perrot JL, Cambazard F. Ex vivo confocal microscopy: a new diagnostic technique for mucormycosis. Skin Res Technol. 2016 May;22(2):203-7. doi: 10.1111/srt.12251. Epub 2015 Oct 27. PMID: 26508203. Link <https://pubmed.ncbi.nlm.nih.gov/26508203/>
486. Yu S, Chen HB. Tuberculosis of Lymph Node Combined with Pulmonary Mucormycosis. Chin Med J (Engl). 2015 Oct 20;128(20):2812-4. doi: 10.4103/0366-6999.167363. PMID: 26481751; PMCID: PMC4736879. Link
487. Kara M, Erdoğan H, Toroslu T, Akçalı A, Tatman Okun M, Reşorlu M, Dereköy FS. Rino-orbito-serebral mukormikozis: Literatür eşliğinde iki olgu sunumu [Rhino-orbito-cerebral mucormycosis: two case reports in the light of the literature]. Kulak Burun Bogaz Ihtis Derg. 2015;25(5):295-301. Turkish. doi: 10.5606/kbbihtisas.2015.45649. PMID: 26476519. Link <https://pubmed.ncbi.nlm.nih.gov/26476519/>
488. Zimmermann N, Hagen MC, Schrager JJ, Hebbeler-Clark RS, Masineni S. Utility of frozen section analysis for fungal organisms in soft tissue wound debridement margin determination. Diagn Pathol. 2015 Oct 15;10:188. doi: 10.1186/s13000-015-0423-9. PMID: 26470865; PMCID: PMC4608183 Link <https://pubmed.ncbi.nlm.nih.gov/26470865/>
489. Dhakar MB, Rayes M, Kupsky W, Tselis A, Norris G. A Cryptic Case: Isolated Cerebral Mucormycosis. Am J Med. 2015 Dec;128(12):1296-9. doi: 10.1016/j.amjmed.2015.08.033. Epub 2015 Oct 9. PMID: 26450170. Link <https://pubmed.ncbi.nlm.nih.gov/26450170/>
490. Cofré F, Villarroel M, Castellón L, Santolaya ME. Tratamiento exitoso de una mucormicosis rinocerebral persistente en un paciente pediátrico durante el debut de una leucemia aguda [Successful treatment of a persistent rhino-cerebral mucormycosis in a pediatric patient with a debut of acute lymphoblastic leukemia]. Rev Chilena Infectol. 2015 Aug;32(4):458-63. Spanish. doi: 10.4067/S0716-10182015000500015. PMID: 26436794. Link <https://pubmed.ncbi.nlm.nih.gov/26436794/>
491. Lopez-Pastorini A, Koryllos A, Brockmann M, Windisch W, Stoelben E. Pseudoaneurysm of the pulmonary artery with massive haemoptysis due to an invasive pulmonary mucormycosis. Thorax. 2016 Feb;71(2):199-200. doi: 10.1136/thoraxjnl-2015-207713. Epub 2015 Sep 18. PMID: 26385775. Link <https://pubmed.ncbi.nlm.nih.gov/26385775/>
492. Ayadi-Kaddour A, Braham E, Marghli A, Ismail O, Helal I, Mlika M, Kilani T, El Mezni F. Une mycose pulmonaire d'issue fatale chez une patiente diabétique et cirrhotique [Fatal pulmonary mycosis in a diabetic and cirrhotic patient]. Tunis Med. 2015 Apr;93(4):259-62. French. PMID: 26375745. Link <https://pubmed.ncbi.nlm.nih.gov/26375745/>
493. Porto DA, Kohen LL. Pulsatile nodule on the ventral wrist. J Am Acad Dermatol. 2015 Oct;73(4):e129-30. doi: 10.1016/j.jaad.2015.05.008. PMID: 26369849. Link <https://pubmed.ncbi.nlm.nih.gov/26369849/>
494. Yang C, Friess SH, Dehner LP. Hepatic Mucormycosis Mimicking Veno-occlusive Disease: Report of a Case and Review of the Literature. Pediatr Dev Pathol. 2016 Mar-Apr;19(2):150-3. doi: 10.2350/15-06-1661-CR.1. Epub 2015 Sep 14. PMID: 26366930. Link <https://pubmed.ncbi.nlm.nih.gov/26366930/>
495. Gerlach MM, Lippmann N, Kobelt L, Petzold-Quinque S, Ritter L, Kiess W, Siekmeyer M. Possible pulmonary Rhizopus oryzae infection in a previously healthy child after a near-drowning incident. Infection. 2016 Jun;44(3):361-4. doi: 10.1007/s15010-015-0839-x. Epub 2015 Sep 13. PMID: 26365402 Link <https://pubmed.ncbi.nlm.nih.gov/26365402/>
496. Tyll T, Lyskova P, Hubka V, Muller M, Zelenka L, Curdova M, Tuckova I, Kolarik M, Hamal P. Early Diagnosis of Cutaneous Mucormycosis Due to Lichtheimia corymbifera After a Traffic Accident. Mycopathologia. 2016 Feb;181(1-2):119-24. doi: 10.1007/s11046-015-9943-9. Epub 2015 Sep 12. PMID: 26363921. Link <https://pubmed.ncbi.nlm.nih.gov/26363921/>
497. Kermani W, Bouttay R, Belcadhi M, Zaghouani H, Ben Ali M, Abdelkéfi M. ENT mucormycosis. Report of 4 cases. Eur Ann Otorhinolaryngol Head Neck Dis. 2016 Apr;133(2):83-6. doi: 10.1016/j.anorl.2015.08.027. Epub 2015 Sep 7. PMID: 26361005. Link <https://pubmed.ncbi.nlm.nih.gov/26361005/>
498. Crisan AM, Ghiaur A, Stancioaca MC, Bardas A, Ghita C, Manea CM, Ionescu B, Coriu D. Mucormycosis during Imatinib treatment: case report. J Med Life. 2015 Jul-Sep;8(3):365-70. PMID: 26351543; PMCID: PMC4556922. Link <https://pubmed.ncbi.nlm.nih.gov/26351543/>
499. Reddy SS, Rakesh N, Chauhan P, Sharma S. Rhinocerebral Mucormycosis Among Diabetic Patients: An Emerging Trend. Mycopathologia. 2015 Dec;180(5-6):389-96. doi: 10.1007/s11046-015-9934-x. Epub 2015 Sep 9. PMID: 26349570. Link <https://pubmed.ncbi.nlm.nih.gov/26349570/>
500. Dhooria S, Agarwal R, Chakrabarti A. Mediastinitis and Bronchial Perforations Due to Mucormycosis. J Bronchology Interv Pulmonol. 2015 Oct;22(4):338-42. doi: 10.1097/LBR.0000000000000170. PMID: 26348693. Link <https://pubmed.ncbi.nlm.nih.gov/26348693/>
501. de Almeida Júnior JN, Ibrahim KY, Del Negro GM, Bezerra ED, Duarte Neto AN, Batista MV, Siciliano RF, Giudice MC, Motta AL, Rossi F, Pierrotti LC, Freire MP, Bellesso M, Pereira J, Abdala E, Benard G. Rhizopus arrhizus and Fusarium solani Concomitant Infection in an Immunocompromised Host. Mycopathologia. 2016 Feb;181(1-2):125-9. doi: 10.1007/s11046-015-9936-8. Epub 2015 Sep 7. PMID: 26346377. Link <https://pubmed.ncbi.nlm.nih.gov/26346377/>
502. Jin H, Cho HH, Kim WJ, Song M, Kim HS, Ko HC, Kim BS, Kim MB, Mun JH. Cutaneous Mucor hiemalis infection diagnosed by molecular methods. Int J Dermatol. 2015 Dec;54(12):e546-8. doi: 10.1111/ijd.12937. Epub 2015 Sep 4. PMID: 26341108 Link <https://pubmed.ncbi.nlm.nih.gov/26341108/>
503. Liao GP, Karanjawala BE, Baba S, Khalil KG, Irani AD. The Whole Picture. A 23-Year-Old Diabetic Female with Persistent Pneumonia and Chronic Lung Abscess. Ann Am Thorac Soc. 2015 Aug;12(8):1231-4. doi: 10.1513/AnnalsATS.201503-145CC. PMID: 26317272. Link <https://pubmed.ncbi.nlm.nih.gov/26317272/>
504. Scharf EL, Cloft HJ, Wijdicks E. Mucor Thrombus. Neurocrit Care. 2016 Apr;24(2):268-72. doi: 10.1007/s12028-015-0186-x. PMID: 26227632. Link <https://pubmed.ncbi.nlm.nih.gov/26227632/>
505. Sarău CA, Lighezan DF, Doroş IC, Ştefănescu EH, Iovănescu G, Balica NC, Horhat ID, Poenaru M. The involvement of upper airway in Wegener's granulomatosis - about four cases. Rom J Morphol Embryol. 2015;56(2):613-8. PMID: 26193239. Link <https://pubmed.ncbi.nlm.nih.gov/26193239/>
506. Chander J, Kaur M, Bhalla M, Punia RS, Singla N, Bhola K, Alastruey-Izquierdo A, Stchigel AM, Guarro J. Changing Epidemiology of Mucoralean Fungi: Chronic Cutaneous Infection Caused by Mucor irregularis. Mycopathologia. 2015 Oct;180(3-4):181-6. doi: 10.1007/s11046-015-9908-z. Epub 2015 Jul 14. PMID: 26170185. Link <https://pubmed.ncbi.nlm.nih.gov/26170185/>
507. Salami A, Assouan C, Kouyaté M, Kadre A, Yavo-Dosso N, N'Guessan ND. Mucormycose rhinosinusienne révélée par une ulcération nécrotique vélopalatine [Sinonasal mucormycosis revealed by a necrotic velar ulceration]. J Mycol Med. 2015 Sep;25(3):204-7. French. doi: 10.1016/j.mycmed.2015.06.001. Epub 2015 Jul 2. PMID: 26142709. Link <https://pubmed.ncbi.nlm.nih.gov/26142709/>
508. Reinbold C, Derder M, Hivelin M, Ozil C, Al Hindi A, Lantieri L. Using free flaps for reconstruction during infections by mucormycosis: A case report and a structured review of the literature. Ann Chir Plast Esthet. 2016 Apr;61(2):153-61. doi: 10.1016/j.anplas.2015.05.006. Epub 2015 Jun 22. PMID: 26113355. Link <https://pubmed.ncbi.nlm.nih.gov/26113355/>
509. Camargo JF, Yakoub D, Cho-Vega JH. Successful Treatment of Primary Cutaneous Mucormycosis Complicating Anti-TNF Therapy with a Combination of Surgical Debridement and Oral Posaconazole. Mycopathologia. 2015 Oct;180(3-4):187-92. doi: 10.1007/s11046-015-9914-1. Epub 2015 Jun 26. PMID: 26112998. Link <https://pubmed.ncbi.nlm.nih.gov/26112998/>
510. Mohamed MS, Abdel-Motaleb HY, Mobarak FA. Management of rhino-orbital mucormycosis. Saudi Med J. 2015 Jul;36(7):865-8. doi: 10.15537/smj.2015.7.11859. PMID: 26108594; PMCID: PMC4503909. Link <https://pubmed.ncbi.nlm.nih.gov/26108594/>
511. Dhaliwal HS, Singh A, Sinha SK, Nampoothiri RV, Goyal A, Chatterjee D, Dewana SK, Lal A, Das A, Gupta KL, Kochhar R. Diagnosed only if considered: isolated renal mucormycosis. Lancet. 2015 Jun 6;385(9984):2322. doi: 10.1016/S0140-6736(15)60730-9. PMID: 26088500. Link <https://pubmed.ncbi.nlm.nih.gov/26088500/>
512. lvarado-Lezama J, Espinosa-González O, García-Cano E, Sánchez-Córdova G. Gastritis enfisematosa secundaria a mucormicosis gástrica [Emphysematous gastritis secondary to gastric mucormycosis]. Cir Cir. 2015 Jan-Feb;83(1):56-60. Spanish. doi: 10.1016/j.circir.2015.04.025. PMID: 25982610. Link <https://pubmed.ncbi.nlm.nih.gov/25982610/>
513. Berrevoets MA, Verweij PE, van der Velden WJ. A patient with flank pain and haematuria after allogeneic stem cell transplantation. Neth J Med. 2015 May;73(4):187-9. PMID: 25968292. Link <https://pubmed.ncbi.nlm.nih.gov/25968292/>
514. Al Barbarawi MM, Allouh MZ. Successful Management of a Unique Condition of Isolated Intracranial Mucormycosis in an Immunocompetent Child. Pediatr Neurosurg. 2015;50(3):165-7. doi: 10.1159/000381750. Epub 2015 May 7. PMID: 25967858. Link https://pubmed.ncbi.nlm.nih.gov/25967858/
515. Farooq AV, Patel RM, Lin AY, Setabutr P, Sartori J, Aakalu VK. Fungal Orbital Cellulitis: Presenting Features, Management and Outcomes at a Referral Center. Orbit. 2015 Jun;34(3):152-9. doi: 10.3109/01676830.2015.1014512. Epub 2015 Apr 23. PMID: 25906127; PMCID: PMC5466351. Link <https://pubmed.ncbi.nlm.nih.gov/25906127/>
516. Akers KS, Rowan MP, Niece KL, Graybill JC, Mende K, Chung KK, Murray CK. Antifungal wound penetration of amphotericin and voriconazole in combat-related injuries: case report. BMC Infect Dis. 2015 Apr 15;15:184. doi: 10.1186/s12879-015-0918-8. PMID: 25886578; PMCID: PMC4403850. Link <https://pubmed.ncbi.nlm.nih.gov/25886578/>
517. Chahal HS, Abgaryan N, Lakshminarayanan R, Glover AT. Orbital Mucormycosis Following Periorbital Cutaneous Infection. Ophthalmic Plast Reconstr Surg. 2017 May/Jun;33(3S Suppl 1):S146-S148. doi: 10.1097/IOP.0000000000000466. PMID: 25853503. Link <https://pubmed.ncbi.nlm.nih.gov/25853503/>
518. Zhu X, Liu H, Wang W, Song S, Jin M, Hu X, Zhang X. Two cases of transplant renal artery thrombosis and spontaneous rupture caused by mucormycosis. Transpl Infect Dis. 2015 Jun;17(3):442-8. doi: 10.1111/tid.12387. Epub 2015 May 26. PMID: 25846151. Link <https://pubmed.ncbi.nlm.nih.gov/25846151/>
519. Dodémont M, Hites M, Bailly B, Trepant AL, De Mendonça R, Denis O, Jacobs F, Montesinos I. When you can't see the wood for the trees. Mucor circinelloides: A rare case of primary cutaneous zygomycosis. J Mycol Med. 2015 Jun;25(2):151-4. doi: 10.1016/j.mycmed.2015.02.043. Epub 2015 Apr 1. PMID: 25840851. Link <https://pubmed.ncbi.nlm.nih.gov/25840851/>
520. Xia XJ, Shen H, Liu ZH. Primary cutaneous mucormycosis caused by Mucor irregularis. Clin Exp Dermatol. 2015 Dec;40(8):875-8. doi: 10.1111/ced.12642. Epub 2015 Mar 25. PMID: 25810249. Link <https://pubmed.ncbi.nlm.nih.gov/25810249/>
521. Ressaire Q, Padoin C, Chaouat M, Maurel V, Alanio A, Ferry A, Soussi S, Benyamina M, Denis B, Mimoun M, Mebazaa A, Legrand M. Muscle diffusion of liposomal amphotericin B and posaconazole in critically ill burn patients receiving continuous hemodialysis. Intensive Care Med. 2015 May;41(5):948-9. doi: 10.1007/s00134-015-3754-9. Epub 2015 Mar 24. PMID: 25800585. Link <https://pubmed.ncbi.nlm.nih.gov/25800585/>
522. Davies BW, Smith JM, Hink EM, Durairaj VD. Increased Incidence of Rhino-Orbital-Cerebral Mucormycosis After Colorado Flooding. Ophthalmic Plast Reconstr Surg. 2017 May/Jun;33(3S Suppl 1):S148-S151. doi: 10.1097/IOP.0000000000000448. PMID: 25794032. Link <https://pubmed.ncbi.nlm.nih.gov/25794032/>
523. Joos ZP, Patel BC. Intraorbital Irrigation of Amphotericin B in the Treatment of Rhino-Orbital Mucormycosis. Ophthalmic Plast Reconstr Surg. 2017 Jan/Feb;33(1):e13-e16. doi: 10.1097/IOP.0000000000000377. PMID: 25794024. Link <https://pubmed.ncbi.nlm.nih.gov/25794024/>
524. Zaman K, Kaur H, Rudramurthy SM, Singh M, Parashar A, Chakrabarti A. Cutaneous mucormycosis of scalp and eyelids in a child with type I diabetes mellitus. Indian J Dermatol Venereol Leprol. 2015 May-Jun;81(3):275-8. doi: 10.4103/0378-6323.152740. PMID: 25784223. Link <https://pubmed.ncbi.nlm.nih.gov/25784223/>
525. Aydi Z, Ben Dhaou B, Baili L, Daoud F, Koubaa W, Debbiche A, Boussema F. Une mucor mycose et une tuberculose retro-orbitaire: un train peut en cacher un autre!!! [Retro-orbital mucormycosis and tuberculosis: a train may hide another]. Tunis Med. 2014 Jul;92(7):505-7. French. PMID: 25775294. Link <https://pubmed.ncbi.nlm.nih.gov/25775294/>
526. Vercillo MS, Liptay MJ, Seder CW. Early pneumonectomy for pulmonary mucormycosis. Ann Thorac Surg. 2015 Mar;99(3):e67-8. doi: 10.1016/j.athoracsur.2014.11.061. PMID: 25742861. Link <https://pubmed.ncbi.nlm.nih.gov/25742861/>
527. Matsudate Y, Murao K, Urano Y, Yarita K, Kamei K, Takeichi H, Kubo Y. Primary cutaneous mucormycosis caused by Mucor irregularis in an immunocompetent patient. J Dermatol. 2015 Mar;42(3):267-8. doi: 10.1111/1346-8138.12788. PMID: 25736319. Link <https://pubmed.ncbi.nlm.nih.gov/25736319/>
528. Rodríguez-Gutiérrez G, Carrillo-Casas EM, Arenas R, García-Méndez JO, Toussaint S, Moreno-Morales ME, Schcolnik-Cabrera AA, Xicohtencatl-Cortes J, Hernández-Castro R. Mucormycosis in a Non-Hodgkin Lymphoma Patient Caused by Syncephalastrum racemosum: Case Report and Review of Literature. Mycopathologia. 2015 Aug;180(1-2):89-93. doi: 10.1007/s11046-015-9878-1. Epub 2015 Mar 5. PMID: 25736172. Link <https://pubmed.ncbi.nlm.nih.gov/25736172/>
529. Shahani L. Cerebral and pulmonary involvement in an immunocompromised host. BMJ Case Rep. 2015 Feb 25;2015:bcr2014208822. doi: 10.1136/bcr-2014-208822. PMID: 25716046; PMCID: PMC4342635. Link <https://pubmed.ncbi.nlm.nih.gov/25716046/>
530. Vallabhaneni S, Walker TA, Lockhart SR, Ng D, Chiller T, Melchreit R, Brandt ME, Smith RM; Centers for Disease Control and Prevention (CDC). Notes from the field: Fatal gastrointestinal mucormycosis in a premature infant associated with a contaminated dietary supplement--Connecticut, 2014. MMWR Morb Mortal Wkly Rep. 2015 Feb 20;64(6):155-6. PMID: 25695322; PMCID: PMC4584706. Link <https://pubmed.ncbi.nlm.nih.gov/25695322/>
531. Salgado P, Suarez-de-la-Rica A, Maseda E, Maggi G, Hernández-Gancedo C, Lopez-Tofiño A, Palacios E, Ruiz E, Gilsanz F. Severe Mucor necrotizing fasciitis associated to dipyrone-induced agranulocytosis. Rev Esp Quimioter. 2015 Feb;28(1):58-60. PMID: 25690148. Link <https://pubmed.ncbi.nlm.nih.gov/25690148/>
532. Church JT, Posluszny JA, Hemmila M, To KB, Cherry-Bukowiec JR, Waljee J. Methylene blue for burn-induced vasoplegia: case report and review of literature. J Burn Care Res. 2015 Mar-Apr;36(2):e107-11. doi: 10.1097/BCR.0000000000000134. PMID: 25687361. Link <https://pubmed.ncbi.nlm.nih.gov/25687361/>
533. Coronel-Pérez IM, Rodríguez-Rey EM, Castilla-Guerra L, Domínguez MC. Primary Cutaneous Mucormycosis Due to Saksenaea vasiformis in an Immunocompetent Patient. Actas Dermosifiliogr. 2015 Jul-Aug;106(6):516-8. English, Spanish. doi: 10.1016/j.ad.2014.12.005. Epub 2015 Feb 7. PMID: 25661417. Link <https://pubmed.ncbi.nlm.nih.gov/25661417/>
534. Alraiyes AH, Kumar A, Gildea TR. Peering beyond an occluded airway. Ann Am Thorac Soc. 2015 Jan;12(1):124-7. doi: 10.1513/AnnalsATS.201409-438CC. PMID: 25635659. Link <https://pubmed.ncbi.nlm.nih.gov/25635659/>
535. Gelman A, Valdes-Rodriguez R, Bhattacharyya S, Yosipovitch G. A case of primary cutaneous mucormycosis caused by minor trauma. Dermatol Online J. 2015 Jan 15;21(1):13030/qt8qn2g3zv. PMID: 25612128. Link <https://pubmed.ncbi.nlm.nih.gov/25612128/>
536. Chan Y, Selvaratnam V, Garg N. A fungating spica. BMJ Case Rep. 2015 Jan 21;2015:bcr2014206901. doi: 10.1136/bcr-2014-206901. PMID: 25608979; PMCID: PMC4307088. Link <https://pubmed.ncbi.nlm.nih.gov/25608979/>
537. Pozo Laderas JC, Pontes Moreno A, Pozo Salido C, Robles Arista JC, Linares Sicilia MJ. Mucormicosis diseminadas en pacientes sin inmunodeficiencias: una enfermedad que también existe [Disseminated mucormycosis in immunocompetent patients: A disease that also exists]. Rev Iberoam Micol. 2015 Apr-Jun;32(2):63-70. Spanish. doi: 10.1016/j.riam.2014.01.006. Epub 2014 Aug 29. PMID: 25543322. Link <https://pubmed.ncbi.nlm.nih.gov/25543322/>
538. Tan M, Gibney EM. Lung mass in a kidney transplant recipient. Am J Transplant. 2015 Jan;15(1):281-2; quiz 283. doi: 10.1111/ajt.13026. PMID: 25534451. Link <https://pubmed.ncbi.nlm.nih.gov/25534451/>
539. Mutchnick S, Soares D, Shkoukani M. To exenterate or not? An unusual case of pediatric rhinocerebral mucormycosis. Int J Pediatr Otorhinolaryngol. 2015 Feb;79(2):267-70. doi: 10.1016/j.ijporl.2014.11.028. Epub 2014 Dec 3. PMID: 25510987. Link <https://pubmed.ncbi.nlm.nih.gov/25510987/>
540. Yamaguchi S, Okubo Y, Katano A, Sano A, Uezato H, Takahashi K. Primary cutaneous mucormycosis caused by Mucor irregularis in an elderly person. J Dermatol. 2015 Feb;42(2):210-4. doi: 10.1111/1346-8138.12736. Epub 2014 Dec 16. PMID: 25510200. Link <https://pubmed.ncbi.nlm.nih.gov/25510200/>
541. Kanemaru M, Tashima S, Yamazaki A, Masuda K, Nagoshi H, Kobayashi T, Kuroda J, Hiruma M, Taniwaki M, Katoh N. Disseminated mucormycosis due to Rhizopus oryzae diagnosed by skin biopsy. J Dermatol. 2015 Jan;42(1):100-1. doi: 10.1111/1346-8138.12715. Epub 2014 Dec 3. PMID: 25470108. Link <https://pubmed.ncbi.nlm.nih.gov/25470108/>
542. Lambert D, Nerot C, Huguenin A, Diallo S, Mzabi A, Ohl X, Noel V, Rouger C, Strady C, Villena I, Bani-Sadr F, Toubas D. Mucormycoses post-traumatiques à Lichtheimia corymbifera: à propos de 3 cas [Post-traumatic mucormycosis due to Lichtheimia corymbifera: three case reports]. J Mycol Med. 2014 Dec;24(4):345-50. French. doi: 10.1016/j.mycmed.2014.10.013. Epub 2014 Nov 20. PMID: 25467816. Link <https://pubmed.ncbi.nlm.nih.gov/25467816/>
543. Shigemura T, Nakazawa Y, Matsuda K, Motobayashi M, Saito S, Koike K. Evaluation of Mucorales DNA load in cerebrospinal fluid in a patient with possible cerebral mucormycosis treated with intravenous liposomal amphotericin B. Int J Infect Dis. 2014 Dec;29:200-2. doi: 10.1016/j.ijid.2014.10.019. Epub 2014 Nov 6. PMID: 25461663. Link <https://pubmed.ncbi.nlm.nih.gov/25461663/>
544. Lai MC, Zhang W, Yang Z, Zhang W, Owusu-Ansah KG, Yu SF, Geng L, Xie HY, Zhou L, Zheng SS. First case report of isolated penile mucormycosis in a liver transplantation recipient. Int J Infect Dis. 2014 Dec;29:208-10. doi: 10.1016/j.ijid.2014.09.017. Epub 2014 Nov 5. PMID: 25447726. Link <https://pubmed.ncbi.nlm.nih.gov/25447726/>
545. Kim YI, Kang HC, Lee HS, Choi JS, Seo KH, Kim YH, Na J. Invasive pulmonary mucormycosis with concomitant lung cancer presented with massive hemoptysis by huge pseudoaneurysm of pulmonary artery. Ann Thorac Surg. 2014 Nov;98(5):1832-5. doi: 10.1016/j.athoracsur.2013.12.051. Epub 2014 Oct 30. PMID: 25441799. Link <https://pubmed.ncbi.nlm.nih.gov/25441799/>
546. Su YY, Chang TY, Wang CJ, Jaing TH, Hsueh C, Chiu CH, Huang YC, Chen SH. Disseminated Cunninghamella bertholletiae Infection During Induction Chemotherapy in a Girl with High-Risk Acute Lymphoblastic Leukemia. Pediatr Neonatol. 2016 Dec;57(6):531-534. doi: 10.1016/j.pedneo.2014.04.010. Epub 2014 Oct 28. PMID: 25440778. Link <https://pubmed.ncbi.nlm.nih.gov/25440778/>
547. Chamdine O, Gaur AH, Broniscer A. Effective treatment of cerebral mucormycosis associated with brain surgery. Pediatr Infect Dis J. 2015 May;34(5):542-3. doi: 10.1097/INF.0000000000000626. PMID: 25420158; PMCID: PMC4400195. Link <https://pubmed.ncbi.nlm.nih.gov/25420158/>
548. de Clerck F, Van Ryckeghem F, Depuydt P, Benoit D, Druwé P, Hugel A, Claeys G, Cools P, Decruyenaere J. Dual disseminated infection with Nocardia farcinica and Mucor in a patient with systemic lupus erythematosus: a case report. J Med Case Rep. 2014 Nov 20;8:376. doi: 10.1186/1752-1947-8-376. PMID: 25410282; PMCID: PMC4244119. Link <https://pubmed.ncbi.nlm.nih.gov/25410282/>
549. Forrester JD, Chandra V, Shelton AA, Weiser TG. Gastrointestinal mucormycosis requiring surgery in adults with hematologic malignant tumors: literature review. Surg Infect (Larchmt). 2015 Apr;16(2):194-202. doi: 10.1089/sur.2013.232. Epub 2014 Nov 18. PMID: 25405775. Link <https://pubmed.ncbi.nlm.nih.gov/25405775/>
550. Sathe KP, Mehta KP. Irreversible fatal renal failure resulting from isolated renal mucormycosis. Saudi J Kidney Dis Transpl. 2014 Nov;25(6):1312-4. doi: 10.4103/1319-2442.144298. PMID: 25394458. Link <https://pubmed.ncbi.nlm.nih.gov/25394458/>
551. Non L, Sta Cruz JP, Tuazon S. Sudden death in a patient with bone marrow transplant by a fungus among us. BMJ Case Rep. 2014 Nov 12;2014:bcr2014207403. doi: 10.1136/bcr-2014-207403. PMID: 25391828; PMCID: PMC4244385. Link <https://pubmed.ncbi.nlm.nih.gov/25391828/>
552. Davuodi S, Manshadi SA, Salehi MR, Yazdi F, Khazravi M, Fazli JT. Fatal cutaneous mucormycosis after kidney transplant. Exp Clin Transplant. 2015 Feb;13(1):82-5. doi: 10.6002/ect.2013.0216. Epub 2014 Nov 5. PMID: 25376012. Link <https://pubmed.ncbi.nlm.nih.gov/25376012/>
553. Cho E, Kim MH. What is your diagnosis? Rhino-orbital-cerebral mucormycosis. Cutis. 2014 Oct;94(4):168, 195-6. PMID: 25372258. Link <https://pubmed.ncbi.nlm.nih.gov/25372258/>
554. Atty C, Alagiozian-Angelova VM, Kowal-Vern A. Black plaques and white nodules in a burn patient. Fusarium and Mucormycosis. JAMA Dermatol. 2014 Dec;150(12):1355-6. doi: 10.1001/jamadermatol.2014.2463. PMID: 25338100. Link <https://pubmed.ncbi.nlm.nih.gov/25338100/>
555. Ferrell BA, Tolle JJ. Invasive endobronchial mucormycosis. Am J Respir Crit Care Med. 2014 Oct 15;190(8):e28. doi: 10.1164/rccm.201312-2229IM. PMID: 25317473. Link <https://pubmed.ncbi.nlm.nih.gov/25317473/>
556. Pajpani M, Webb R. Lingual necrosis caused by mucormycosis in a patient with aplastic anaemia: case report. Br J Oral Maxillofac Surg. 2014 Dec;52(10):e144-6. doi: 10.1016/j.bjoms.2014.09.012. Epub 2014 Oct 7. PMID: 25300888. Link https://pubmed.ncbi.nlm.nih.gov/25300888/
557. Bonifaz A, Stchigel AM, Guarro J, Guevara E, Pintos L, Sanchis M, Cano-Lira JF. Primary cutaneous mucormycosis produced by the new species Apophysomyces mexicanus. J Clin Microbiol. 2014 Dec;52(12):4428-31. doi: 10.1128/JCM.02138-14. Epub 2014 Oct 8. PMID: 25297328; PMCID: PMC4313335. Link <https://pubmed.ncbi.nlm.nih.gov/25297328/>
558. Mangaraj S, Sethy G, Patro MK, Padhi S. A rare case of subcutaneous mucormycosis due to Syncephalastrum racemosum: case report and review of literature. Indian J Med Microbiol. 2014 Oct-Dec;32(4):448-51. doi: 10.4103/0255-0857.142252. PMID: 25297037. Link <https://pubmed.ncbi.nlm.nih.gov/25297037/>
559. Ayadi-Kaddour A, Ammar J, Ismail O, Braham E, Hamzaoui A, Kilani T, Zidi A, El Mezni F. Mucormycose pulmonaire chez une enfant diabétique, compliquée d'un thrombus intra-auriculaire gauche [Pulmonary zygomycosis in a diabetic child complicated with thrombus of the left atrial auricle]. Arch Pediatr. 2014 Nov;21(11):1241-5. French. doi: 10.1016/j.arcped.2014.08.004. Epub 2014 Oct 2. PMID: 25282464. Link <https://pubmed.ncbi.nlm.nih.gov/25282464/>
560. Ville S, Talarmin JP, Gaultier-Lintia A, Bouquié R, Sagan C, Le Pape P, Giral M, Morio F. Disseminated Mucormycosis With Cerebral Involvement Owing to Rhizopus Microsporus in a Kidney Recipient Treated With Combined Liposomal Amphotericin B and Posaconazole Therapy. Exp Clin Transplant. 2016 Feb;14(1):96-9. doi: 10.6002/ect.2014.0093. Epub 2014 Sep 30. PMID: 25275881. Link <https://pubmed.ncbi.nlm.nih.gov/25275881/>
561. Cañete-Gómez J, Segura-Sampedro JJ, Reguera-Rosal J. An unusual cause of pneumoperitoneum. Gastroenterology. 2014 Nov;147(5):e3-5. doi: 10.1053/j.gastro.2014.05.041. Epub 2014 Sep 26. PMID: 25263298. Link <https://pubmed.ncbi.nlm.nih.gov/25263298/>
562. Silberstein E, Krieger Y, Rosenberg N, Bogdanov-Berezovsky A, Shoham Y, Saphier O, Pagkalos VA, Joshua BZ. Facial Reconstruction of a Mucormycosis Survivor by Free Rectus Abdominis Muscle Flap, Tissue Expansion, and Ocular Prosthesis. Ophthalmic Plast Reconstr Surg. 2016 Nov/Dec;32(6):e131-e132. doi: 10.1097/IOP.0000000000000314. PMID: 25233097. Link <https://pubmed.ncbi.nlm.nih.gov/25233097/>
563. Klimko NN, Khostelidi SN, Volkova AG, Popova MO, Bogomolova TS, Zuborovskaya LS, Kolbin AS, Medvedeva NV, Zuzgin IS, Simkin SM, Vasilyeva NV, Afanasiev BV. Mucormycosis in haematological patients: case report and results of prospective study in Saint Petersburg, Russia. Mycoses. 2014 Dec;57 Suppl 3:91-6. doi: 10.1111/myc.12247. Epub 2014 Sep 3. PMID: 25187314. Link <https://pubmed.ncbi.nlm.nih.gov/25187314/>
564. Yusef D, Waran A, Vamvakiti E. A 16-year-old boy with emphysematous gastritis and oesophageal candidiasis. BMJ Case Rep. 2014 Sep 11;2014:bcr2014203755. doi: 10.1136/bcr-2014-203755. PMID: 25213783; PMCID: PMC4166240. Link <https://www.ncbi.nlm.nih.gov/pmc/articles/PMC4166240/>
565. Joulali T, Khatouf M. Une mucormycose mammaire compliquant un intertrigo: une localisation atypique avec évolution fatale [Mucormycosis complicating breast intertrigo: an unusual location with fatal outcome]. Pan Afr Med J. 2014 Jan 13;17:5. French. doi: 10.11604/pamj.2014.17.5.3811. PMID: 25184022; PMCID: PMC4149792. Link <https://pubmed.ncbi.nlm.nih.gov/25184022/>
566. Dave VP, Sharma S, Yogi R, Reddy S. Apophysomyces elegans: a novel cause of endogenous endophthalmitis in an immunocompetent individual. Int Ophthalmol. 2014 Dec;34(6):1285-9. doi: 10.1007/s10792-014-9999-4. Epub 2014 Sep 3. PMID: 25183462. Link <https://pubmed.ncbi.nlm.nih.gov/25183462/>
567. Kaur R, Bala K, Ahuja RB, Srivastav P, Bansal U. Primary cutaneous mucormycosis in a patient with burn wounds due to Lichtheimia ramosa. Mycopathologia. 2014 Oct;178(3-4):291-5. doi: 10.1007/s11046-014-9805-x. Epub 2014 Aug 29. PMID: 25168130. Link <https://pubmed.ncbi.nlm.nih.gov/25168130/>
568. Layios N, Canivet JL, Baron F, Moutschen M, Hayette MP. Mortierella wolfii-associated invasive disease. Emerg Infect Dis. 2014 Sep;20(9):1591-2. doi: 10.3201/eid2009.140469. PMID: 25153198; PMCID: PMC4178392. Link <https://pubmed.ncbi.nlm.nih.gov/25153198/>
569. Neto FM, Camargo PC, Costa AN, Teixeira RH, Carraro RM, Afonso JE Jr, Campos SV, Samano MN, Fernandes LM, Abdalla LG, Pêgo-Fernandes PM. Fungal infection by Mucorales order in lung transplantation: 4 case reports. Transplant Proc. 2014 Jul-Aug;46(6):1849-51. doi: 10.1016/j.transproceed.2014.05.033. PMID: 25131052. Link <https://pubmed.ncbi.nlm.nih.gov/25131052/>
570. Knoll BM. Pharmacokinetics of oral isavuconazole in a patient after Roux-en-Y gastric bypass surgery. J Antimicrob Chemother. 2014 Dec;69(12):3441-3. doi: 10.1093/jac/dku311. Epub 2014 Aug 11. PMID: 25114167. Link <https://pubmed.ncbi.nlm.nih.gov/25114167/>
571. Ermak D, Kanekar S, Specht CS, Wojnar M, Lowden M. Looks like a stroke, acts like a stroke, but it's more than a stroke: a case of cerebral mucormycosis. J Stroke Cerebrovasc Dis. 2014 Sep;23(8):e403-e404. doi: 10.1016/j.jstrokecerebrovasdis.2014.02.024. Epub 2014 Aug 6. PMID: 25106832. Link <https://pubmed.ncbi.nlm.nih.gov/25106832/>
572. Malkan AD, Wahid FN, Rao BN, Sandoval JA. Aggressive Cunninghamella pneumonia in an adolescent. J Pediatr Hematol Oncol. 2014 Oct;36(7):581-2. doi: 10.1097/MPH.0000000000000235. PMID: 25089609. Link <https://pubmed.ncbi.nlm.nih.gov/25089609/>
573. Davoudi S, Anderlini P, Fuller GN, Kontoyiannis DP. A long-term survivor of disseminated Aspergillus and mucorales infection: an instructive case. Mycopathologia. 2014 Dec;178(5-6):465-70. doi: 10.1007/s11046-014-9785-x. Epub 2014 Aug 3. PMID: 25086667. Link <https://pubmed.ncbi.nlm.nih.gov/25086667/>
574. Klimova K, Padilla Suárez C, Peláez T, Salcedo Plaza M. Mucormicosis cutánea como complicación poco frecuente de trasplante hepático [Cutaneous mucormycosis as a rare complication of a liver transplantation]. Enferm Infecc Microbiol Clin. 2014 Oct;32(8):537-9. Spanish. doi: 10.1016/j.eimc.2014.05.007. Epub 2014 Jul 28. PMID: 25081551. Link <https://pubmed.ncbi.nlm.nih.gov/25081551/>
575. Lim MY, Alker AP, Califano S, Trembath DG, Alby K, Gilligan PH, Jamieson K, Serody JS, Shea TC. Concurrent Disseminated Nocardiosis and GI Mucormycosis in a Stem-Cell Transplantation Recipient. J Clin Oncol. 2016 Apr 1;34(10):e84-6. doi: 10.1200/JCO.2013.51.4042. Epub 2014 Jul 28. PMID: 25071136. Link <https://pubmed.ncbi.nlm.nih.gov/25071136/>
576. Dimaka K, Mallis A, Naxakis SS, Marangos M, Papadas TA, Stathas T, Mastronikolis NS. Chronic rhinocerebral mucormycosis: a rare case report and review of the literature. Mycoses. 2014 Nov;57(11):699-702. doi: 10.1111/myc.12219. Epub 2014 Jul 15. PMID: 25039925. Link <https://pubmed.ncbi.nlm.nih.gov/25039925/>
577. Kogure Y, Nakamura F, Shinozaki-Ushiku A, Watanabe A, Kamei K, Yoshizato T, Nannya Y, Fukayama M, Kurokawa M. Pulmonary mucormycosis with embolism: two autopsied cases of acute myeloid leukemia. Int J Clin Exp Pathol. 2014 May 15;7(6):3449-53. PMID: 25031775; PMCID: PMC4097268. Link <https://pubmed.ncbi.nlm.nih.gov/25031775/>
578. Byard RW. Unusual patterned skin lesions caused by postmortem fungal activity. Forensic Sci Med Pathol. 2014 Dec;10(4):651-3. doi: 10.1007/s12024-014-9589-9. Epub 2014 Jul 16. PMID: 25027416. Link <https://pubmed.ncbi.nlm.nih.gov/25027416/>
579. Malik AN, Bi WL, McCray B, Abedalthagafi M, Vaitkevicius H, Dunn IF. Isolated cerebral mucormycosis of the basal ganglia. Clin Neurol Neurosurg. 2014 Sep;124:102-5. doi: 10.1016/j.clineuro.2014.06.022. Epub 2014 Jun 23. PMID: 25019460; PMCID: PMC4256958. Link <https://pubmed.ncbi.nlm.nih.gov/25019460/>
580. Carnovale S, Daneri GL. Lichtheimia sp. en un paciente inmunocomprometido [Lichtheimia sp. in an immunodepressed patient]. Rev Argent Microbiol. 2014 Apr-Jun;46(2):161-2. Spanish. doi: 10.1016/S0325-7541(14)70065-3. PMID: 25011601. Link <https://pubmed.ncbi.nlm.nih.gov/25011601/>
581. Mohindra S, Gupta B, Gupta K, Bal A. Tracheal mucormycosis pneumonia: a rare clinical presentation. Respir Care. 2014 Nov;59(11):e178-81. doi: 10.4187/respcare.03174. Epub 2014 Jul 8. PMID: 25006272. Link Mohindra S, Gupta B, Gupta K, Bal A. Tracheal mucormycosis pneumonia: a rare clinical presentation. Respir Care. 2014 Nov;59(11):e178-81. doi: 10.4187/respcare.03174. Epub 2014 Jul 8. PMID: 25006272.
582. Panigrahi MK, Manju R, Kumar SV, Toi PC. Pulmonary mucormycosis presenting as nonresolving pneumonia in a patient with diabetes mellitus. Respir Care. 2014 Dec;59(12):e201-5. doi: 10.4187/respcare.03205. Epub 2014 Jul 8. PMID: 25006269. Link <https://pubmed.ncbi.nlm.nih.gov/25006269/>
583. Rajagopal K, Watkins AC, Gibber M, Kon ZN, Sanchez PG, Iacono AT, Griffith BP. Reoperative lung transplantation for donor-derived pulmonary mucormycosis. Ann Thorac Surg. 2014 Jul;98(1):327-9. doi: 10.1016/j.athoracsur.2013.08.065. PMID: 24996716. Link <https://pubmed.ncbi.nlm.nih.gov/24996716/>
584. Bini R, Addeo A, Maganuco L, Fontana D, Viora T, Leli R. The role of surgery in a case of diffuse mucormycosis with haematemesis and gastric necrosis. Ann R Coll Surg Engl. 2014 Jul;96(5):e31-3. doi: 10.1308/003588414X13946184901687. PMID: 24992411; PMCID: PMC4473966. Link <https://pubmed.ncbi.nlm.nih.gov/24992411/>
585. Navanukroh O, Jitmuang A, Chayakulkeeree M, Ngamskulrungroj P. Disseminated Cunninghamella bertholletiae infection with spinal epidural abscess in a kidney transplant patient: case report and literature review. Transpl Infect Dis. 2014 Aug;16(4):658-65. doi: 10.1111/tid.12251. Epub 2014 Jun 24. PMID: 24962170. Link <https://pubmed.ncbi.nlm.nih.gov/24962170/>
586. Kang D, Jiang X, Wan H, Ran Y, Hao D, Zhang C. Mucor irregularis infection around the inner canthus cured by amphotericin B: a case report and review of published literatures. Mycopathologia. 2014 Aug;178(1-2):129-33. doi: 10.1007/s11046-014-9770-4. Epub 2014 Jun 20. PMID: 24947172. Link <https://pubmed.ncbi.nlm.nih.gov/24947172/>
587. Sirignano S, Blake P, Turrentine JE, Dominguez AR. Primary cutaneous zygomycosis secondary to minor trauma in an immunocompromised pediatric patient: a case report. Dermatol Online J. 2014 Jun 15;20(6):13030/qt4r2455h9. PMID: 24945642. Link <https://pubmed.ncbi.nlm.nih.gov/24945642/>
588. Joichi Y, Chijimatsu I, Yarita K, Kamei K, Miki M, Onodera M, Harada M, Yokozaki M, Kobayashi M, Ohge H. Detection of Mucor velutinosus in a blood culture after autologous peripheral blood stem cell transplantation : a pediatric case report. Med Mycol J. 2014;55(2):E43-8. doi: 10.3314/mmj.55.e43. PMID: 24943207. Link <https://pubmed.ncbi.nlm.nih.gov/24943207/>
589. Mandegari E, Fu L, Arambú C, Montoya S, Peña A, Johnson KM, Perfect JR, Caniza MA. Mucormycosis Rhinosinusitis at Diagnosis of Acute Lymphoblastic Leukemia: Diagnostics and Management Challenges in a Low-Middle-income Country. J Pediatr Hematol Oncol. 2015 Apr;37(3):e173-7. doi: 10.1097/MPH.0000000000000198. PMID: 24942033. Link <https://pubmed.ncbi.nlm.nih.gov/24942033/>
590. Kutlu M, Ergin C, Bir F, Hilmioğlu-Polat S, Gümral R, Necan C, Koçyiğit A, Sayın-Kutlu S. Pulmonary mucormycosis due to Lichtheimia ramosa in a patient with HIV infection. Mycopathologia. 2014 Aug;178(1-2):111-5. doi: 10.1007/s11046-014-9761-5. Epub 2014 Jun 13. PMID: 24925144. Link <https://pubmed.ncbi.nlm.nih.gov/24925144/>
591. Cloyd JM, Brown J, Sinclair T, Jenks D, Desai J, Longacre T, Chandra V, Shelton A. Gastrointestinal mucormycosis initially manifest as hematochezia from arterio-enteric fistula. Dig Dis Sci. 2014 Dec;59(12):2905-8. doi: 10.1007/s10620-014-3239-7. PMID: 24906697. Link <https://pubmed.ncbi.nlm.nih.gov/24906697/>
592. Abuzayed B, Al-Abadi H, Al-Otti S, Baniyaseen K, Al-Sharki Y. Neuronavigation-guided endoscopic endonasal resection of extensive skull base mucormycosis complicated with cerebral vasospasm. J Craniofac Surg. 2014 Jul;25(4):1319-23. doi: 10.1097/SCS.0000000000000786. PMID: 24902115. Link <https://pubmed.ncbi.nlm.nih.gov/24902115/>
593. Barnajian M, Gioia W, Iordache F, Bergamaschi R. Mucormycosis-induced colon perforation after renal transplantation. Surg Infect (Larchmt). 2014 Oct;15(5):665-6. doi: 10.1089/sur.2013.131. Epub 2014 May 27. PMID: 24865304. Link <https://pubmed.ncbi.nlm.nih.gov/24865304/>
594. Bird J, Telang G, Robinson-Bostom L. Two pink nodules in a patient with acute myeloid leukemia. J Cutan Pathol. 2014 Jun;41(6):483-6. doi: 10.1111/cup.12337. PMID: 24853324. Link <https://pubmed.ncbi.nlm.nih.gov/24853324/>
595. Chi M, Kim HJ, Basham R, Yoon MK, Vagefi R, Kersten RC. Temporal Artery Calciphylaxis Presenting as Temporal Arteritis in a Case of Rhinoorbitocerebral Mucormycosis. Ophthalmic Plast Reconstr Surg. 2015 Sep-Oct;31(5):e132-5. doi: 10.1097/IOP.0000000000000181. PMID: 24853118. Link <https://pubmed.ncbi.nlm.nih.gov/24853118/>
596. Grannan BL, Yanamadala V, Venteicher AS, Walcott BP, Barr JC. Use of external ventriculostomy and intrathecal anti-fungal treatment in cerebral mucormycotic abscess. J Clin Neurosci. 2014 Oct;21(10):1819-21. doi: 10.1016/j.jocn.2014.01.008. Epub 2014 May 19. PMID: 24852901. Link <https://pubmed.ncbi.nlm.nih.gov/24852901/>
597. Shigemura T, Nakazawa Y, Matsuda K, Sano K, Yaguchi T, Motobayashi M, Saito S, Noda S, Kobayashi N, Agematsu K, Honda T, Koike K. Serial monitoring of Mucorales DNA load in serum samples of a patient with disseminated mucormycosis after allogeneic bone marrow transplantation. Int J Hematol. 2014 Aug;100(2):206-9. doi: 10.1007/s12185-014-1597-8. Epub 2014 May 22. PMID: 24848632. Link <https://pubmed.ncbi.nlm.nih.gov/24848632/>
598. Idiga J, Rootman DB, Nagiel A, Goldberg RA. Isolated Zygomycetes Endophthalmitis: A Case Report. Ophthalmic Plast Reconstr Surg. 2015 Nov-Dec;31(6):e145-7. doi: 10.1097/IOP.0000000000000187. PMID: 24833448. Link <https://pubmed.ncbi.nlm.nih.gov/24833448/>
599. McCrory MC, Moore BA, Nakagawa TA, Givner LB, Jason DR, Palavecino EL, Ajizian SJ. Disseminated mucormycosis in an adolescent with newly diagnosed diabetes mellitus. Pediatr Infect Dis J. 2014 Oct;33(10):1094-6. doi: 10.1097/INF.0000000000000383. PMID: 24830701. Link <https://pubmed.ncbi.nlm.nih.gov/24830701/>
600. Yuda J, Kato K, Kikushige Y, Ohkusu K, Kiyosuke M, Sakamoto K, Oku S, Miyake N, Kadowaki M, Iino T, Tanimoto K, Takenaka K, Iwasaki H, Miyamoto T, Shimono N, Teshima T, Akashi K. Successful treatment of invasive zygomycosis based on a prompt diagnosis using molecular methods in a patient with acute myelogenous leukemia. Intern Med. 2014;53(10):1087-91. doi: 10.2169/internalmedicine.53.1366. PMID: 24827491. Link <https://pubmed.ncbi.nlm.nih.gov/24827491/>
601. Hamdi A, Mulanovich VE, Matin SF, Landon G, Sircar K, Tu SM, Nieto Y. Isolated renal mucormycosis in a transplantation recipient. J Clin Oncol. 2015 Apr 1;33(10):e50-1. doi: 10.1200/JCO.2013.49.1969. Epub 2014 May 12. PMID: 24821884. Link <https://pubmed.ncbi.nlm.nih.gov/24821884/>
602. Williams KE, Parish JM, Lyng PJ, Viggiano RW, Wesselius LJ, Ocal IT, Vikram HR. Pseudomembranous tracheobronchitis caused by Rhizopus sp. After allogeneic stem cell transplantation. J Bronchology Interv Pulmonol. 2014 Apr;21(2):166-9. doi: 10.1097/LBR.0000000000000058. PMID: 24739694. Link
603. Di Pentima MC, Chan S, Powell J, Napoli JA, Walter AW, Walsh TJ. Topical amphotericin B in combination with standard therapy for severe necrotizing skin and soft-tissue mucormycosis in an infant with bilineal leukemia: case report and review. J Pediatr Hematol Oncol. 2014 Oct;36(7):e468-70. doi: 10.1097/MPH.0000000000000166. PMID: 24732057. <https://pubmed.ncbi.nlm.nih.gov/24732057/>
604. Bhandary SV, VijayaPai H, Rao LG, Yegneswaran PP. Cunninghamella spinosum fungal corneal ulcer- first case report. Indian J Ophthalmol. 2014 Mar;62(3):375-6. doi: 10.4103/0301-4738.130436. PMID: 24722275; PMCID: PMC4061691. Link <https://pubmed.ncbi.nlm.nih.gov/24722275/>
605. Gupta V, Rajagopalan N, Patil M, Shivaprasad C. Aspergillus and mucormycosis presenting with normal chest X-ray in an immunocompromised host. BMJ Case Rep. 2014 Apr 9;2014:bcr2014204022. doi: 10.1136/bcr-2014-204022. PMID: 24717585; PMCID: PMC3987642. Link <https://pubmed.ncbi.nlm.nih.gov/24717585/>
606. Mehmood M, Halloush RA, Khasawneh FA. A 29-year-old man with hospital-acquired cavitary pneumonia. Can Respir J. 2014 Jul-Aug;21(4):208-10. doi: 10.1155/2014/614148. Epub 2014 Apr 7. PMID: 24712011; PMCID: PMC4173885. Link <https://pubmed.ncbi.nlm.nih.gov/24712011/>
607. Kumar N, Hanks ME, Chandrasekaran P, Davis BC, Hsu AP, Van Wagoner NJ, Merlin JS, Spalding C, La Hoz RM, Holland SM, Zerbe CS, Sampaio EP. Gain-of-function signal transducer and activator of transcription 1 (STAT1) mutation-related primary immunodeficiency is associated with disseminated mucormycosis. J Allergy Clin Immunol. 2014 Jul;134(1):236-9. doi: 10.1016/j.jaci.2014.02.037. Epub 2014 Apr 4. PMID: 24709374; PMCID: PMC4125455. Link <https://pubmed.ncbi.nlm.nih.gov/24709374/>
608. Gorovoy IR, Vagefi MR, Russell MS, Gorovoy M, Bloomer MM, Glastonbury CM. Loss of contrast enhancement of the inferior rectus muscle on magnetic resonance imaging in acute fulminant invasive fungal sinusitis. Clin Exp Ophthalmol. 2014 Dec;42(9):885-7. doi: 10.1111/ceo.12337. Epub 2014 Apr 29. PMID: 24666541. Link <https://pubmed.ncbi.nlm.nih.gov/24666541/>
609. Park W, Jang M, Hwang E, Han S, Park S, Kim H, Choe M. Allograft mucormycosis due to Rhizopus microsporus in a kidney transplant recipient. Transplant Proc. 2014;46(2):623-5. doi: 10.1016/j.transproceed.2013.12.017. PMID: 24656029. Link <https://pubmed.ncbi.nlm.nih.gov/24656029/>
610. Desoubeaux G, Leperlier M, Chaussade H, Schneider C, Roriz M, Houssin C, Rogez C, De Muret A, García-Hermoso D, Bailly É, Le Fourn É, Machet L, Chandenier J, Bernard L. Mucormycose cutanée à Rhizopus microsporus [Cutaneous mucormycosis caused by Rhizopus microsporus]. Ann Dermatol Venereol. 2014 Mar;141(3):201-5. French. doi: 10.1016/j.annder.2013.10.044. Epub 2013 Dec 11. PMID: 24635954. Link https://pubmed.ncbi.nlm.nih.gov/24635954/
611. Tuysuz G, Ozdemir N, Senyuz OF, Emre S, Kantarcioglu S, Adaletli I, Kepil N, Tutuncu C, Celkan T. Successful management of hepatic mucormycosis in an acute lymphoblastic leukaemia patient: a case report and review of the literature. Mycoses. 2014 Aug;57(8):513-8. doi: 10.1111/myc.12184. Epub 2014 Mar 18. PMID: 24635874. Link
612. Ishak RS, Kurban M, Abbas O. Erythematous purpuric indurated plaque over right forearm. Pediatr Dermatol. 2014 Mar-Apr;31(2):249-50. doi: 10.1111/pde.12016. PMID: 24606205. Link <https://pubmed.ncbi.nlm.nih.gov/24606205/>
613. Kaiser P, Maggio EM, Pfammatter T, Misselwitz B, Flury S, Schneider PM, Dutkowski P, Breitenstein S, Müllhaupt B, Clavien PA, Mueller NJ. Histopathological evidence of invasive gastric mucormycosis after transarterial chemoembolization and liver transplantation. Infection. 2014 Aug;42(4):779-83. doi: 10.1007/s15010-014-0603-7. Epub 2014 Mar 5. PMID: 24595492. Link <https://pubmed.ncbi.nlm.nih.gov/24595492/>
614. Lundy JB, Driscoll IR. Experience with proctectomy to manage combat casualties sustaining catastrophic perineal blast injury complicated by invasive mucor soft-tissue infections. Mil Med. 2014 Mar;179(3):e347-50. doi: 10.7205/MILMED-D-13-00493. PMID: 24594473. Link <https://pubmed.ncbi.nlm.nih.gov/24594473/>
615. Matsumoto K, Yamamoto W, Ohgusa E, Tanaka M, Maruta A, Ishigatsubo Y, Kanamori H. Disseminated Cunninghamella bertholletiae infection with septic pulmonary embolism after allogeneic bone marrow transplantation. Transpl Infect Dis. 2014 Apr;16(2):304-6. doi: 10.1111/tid.12190. Epub 2014 Mar 5. PMID: 24593246. Link <https://pubmed.ncbi.nlm.nih.gov/24593246/>
616. Calvert W, Mullassery D, Shukla R, Lamont G. Novel therapeutic use of Versajet for intestinal mucormycosis. BMJ Case Rep. 2014 Mar 3;2014:bcr2013202773. doi: 10.1136/bcr-2013-202773. PMID: 24591385; PMCID: PMC3948008. Link <https://pubmed.ncbi.nlm.nih.gov/24591385/>
617. Monteagudo M, Palazón-García E, Lozano-Setién E, García-García J. Signo del cornete negro en un caso de mucormicosis rinocerebral [The 'black turbinate sign' in a case of rhinocerebral mucormycosis]. Rev Neurol. 2014 Mar 1;58(5):234-5. Spanish. PMID: 24570362. Link <https://pubmed.ncbi.nlm.nih.gov/24570362/>
618. He YY, Wang Y, Li M, Xue L, Li CH. [Concurrent mucormycosis in children with acute lymphoblastic leukemia at induced remission stage: two case report]. Zhongguo Dang Dai Er Ke Za Zhi. 2014 Feb;16(2):152-4. Chinese. PMID: 24568908. Link <https://pubmed.ncbi.nlm.nih.gov/24568908/>
619. Shiraishi K, Sasaki S, Sadamoto Y. Cutaneous mucormycosis in a patient with acute lymphocytic leukemia. Eur J Dermatol. 2014 Jan-Feb;24(1):116-7. doi: 10.1684/ejd.2013.2240. PMID: 24566121. Link <https://pubmed.ncbi.nlm.nih.gov/24566121/>
620. Machicado JD, Younes M, Wolf DS. A rare cause of gastrointestinal bleeding in the intensive care unit. Healthcare-associated mucormycosis. Gastroenterology. 2014 Apr;146(4):911, 1136-7. doi: 10.1053/j.gastro.2013.11.038. Epub 2014 Feb 19. PMID: 24560854. Link <https://pubmed.ncbi.nlm.nih.gov/24560854/>
621. Juan YH, Saboo SS, Lin YC, Conner JR, Jacobson FL, Khandelwal A. Reverse halo sign in pulmonary mucormyosis. QJM. 2014 Sep;107(9):777-8. doi: 10.1093/qjmed/hcu031. Epub 2014 Feb 6. PMID: 24509236. Link <https://pubmed.ncbi.nlm.nih.gov/24509236/>
622. Puy-Núñez A, Regal AR, Amigo-Jorrín Mdel C, Álvarez-Martínez M, Gómez-Castro A. Multineuritis craneal aguda de causa fúngica [Acute cranial multineuritis caused by a fungus]. Rev Neurol. 2014 Feb 16;58(4):188-90. Spanish. PMID: 24504884. Link <https://pubmed.ncbi.nlm.nih.gov/24504884/>
623. Nieto-Ríos JF, Moreno-Coral LF, Zapata-Cárdenas A, Ocampo-Kohn C, Aristizabal-Alzate A, Serna-Higuita LM, Ramírez-Sánchez IC, Zuluaga-Valencia GA. Tratamiento exitoso de mucormicosis rino-órbito-cerebral en un paciente trasplantado renal [Successful treatment of rhino-orbital-cerebral mucormycosis in a kidney transplant patient]. Nefrologia. 2014;34(1):120-4. Spanish. doi: 10.3265/Nefrologia.pre2013.Nov.12084. PMID: 24463869. Link <https://pubmed.ncbi.nlm.nih.gov/24463869/>
624. Henn A, Mellon G, Benoît H, Roos-Weil D, Jauréguiberry S, Mordant P, Fekkar A, Caumes E. Disseminated cryptococcosis, invasive aspergillosis, and mucormycosis in a patient treated with alemtuzumab for chronic lymphocytic leukaemia. Scand J Infect Dis. 2014 Mar;46(3):231-4. doi: 10.3109/00365548.2013.866269. Epub 2014 Jan 23. PMID: 24450842. Link <https://pubmed.ncbi.nlm.nih.gov/24450842/>
625. Vondran FW, Knitsch W, Krech T, Erichsen TJ, Sedlacek L, Abbas M, Klempnauer J, Bektas H, Lehner F, Kousoulas L. Intestinal mucormycosis with Rhizopus microsporus after liver transplantation--successful treatment of a rare but life-threatening complication. Transplantation. 2014 Jan 27;97(2):e11-3. doi: 10.1097/01.tp.0000438631.21591.1e. PMID: 24434484. Link <https://pubmed.ncbi.nlm.nih.gov/24434484/>
626. Kim MJ, Park PW, Ahn JY, Kim KH, Seo JY, Jeong JH, Park MJ, Jung JW, Seo YH. Fatal pulmonary mucormycosis caused by Rhizopus microsporus in a patient with diabetes. Ann Lab Med. 2014 Jan;34(1):76-9. doi: 10.3343/alm.2014.34.1.76. Epub 2013 Dec 6. PMID: 24422203; PMCID: PMC3885781. Link <https://pubmed.ncbi.nlm.nih.gov/24422203/>
627. Ye W, Wang Y, Wen Y, Li H, Li X. Dramatic remission of nephrotic syndrome after unusual complication of mucormycosis in idiopathic membranous nephropathy. Int Urol Nephrol. 2014 Jun;46(6):1247-51. doi: 10.1007/s11255-013-0628-3. Epub 2014 Jan 11. PMID: 24414303. Link <https://pubmed.ncbi.nlm.nih.gov/24414303/>
628. Peixoto D, Gagne LS, Hammond SP, Gilmore ET, Joyce AC, Soiffer RJ, Marty FM. Isavuconazole treatment of a patient with disseminated mucormycosis. J Clin Microbiol. 2014 Mar;52(3):1016-9. doi: 10.1128/JCM.03176-13. Epub 2014 Jan 8. PMID: 24403304; PMCID: PMC3957744. Link <https://pubmed.ncbi.nlm.nih.gov/24403304/>
629. Harman M, Uçmak D, Dal T. A rare case of mucormycosis in the scalp. Acta Med Port. 2013 Nov-Dec;26(6):754-7. Epub 2013 Dec 20. PMID: 24388265. Link <https://pubmed.ncbi.nlm.nih.gov/24388265/>
630. de Chaumont A, Pierret C, Janvier F, Goudard Y, de Kerangal X, Chapuis O. Mucormycosis: a rare complication of an amputation. Ann Vasc Surg. 2014 May;28(4):1035.e15-9. doi: 10.1016/j.avsg.2013.10.008. Epub 2013 Dec 14. PMID: 24342831. Link <https://pubmed.ncbi.nlm.nih.gov/24342831/>
631. Ebadi M, Alavi S, Ghojevand N, Aghdam MK, Yazdi MK, Zahiri A. Infantile splenorenopancreatic mucormycosis complicating neuroblastoma. Pediatr Int. 2013 Dec;55(6):e152-5. doi: 10.1111/ped.12182. PMID: 24330301. Link <https://pubmed.ncbi.nlm.nih.gov/24330301/>
632. Relloso S, Romano V, Landaburu MF, Herrera F, Smayevsky J, Veciño C, Mujica MT. Saksenaea erythrospora infection following a serious sailing accident. J Med Microbiol. 2014 Feb;63(Pt 2):317-321. doi: 10.1099/jmm.0.062174-0. Epub 2013 Dec 2. PMID: 24298050. Link <https://pubmed.ncbi.nlm.nih.gov/24298050/>
633. Stewart JI, D'Alonzo GE, Ciccolella DE, Patel NB, Durra H, Clauss HE. Reverse halo sign on chest imaging in a renal transplant recipient. Transpl Infect Dis. 2014 Feb;16(1):115-8. doi: 10.1111/tid.12166. Epub 2013 Dec 2. PMID: 24289813. Link <https://pubmed.ncbi.nlm.nih.gov/24289813/>
634. Ervens J, Ghannoum M, Graf B, Schwartz S. Successful isavuconazole salvage therapy in a patient with invasive mucormycosis. Infection. 2014 Apr;42(2):429-32. doi: 10.1007/s15010-013-0552-6. Epub 2013 Nov 12. PMID: 24217961. Link <https://pubmed.ncbi.nlm.nih.gov/24217961/>
635. Xu S, Nambudiri VE, Tahan S, Seo SJ. Violaceous necrotic plaques on the leg of an immunosuppressed patient. Cutaneous mucormycosis. JAMA Dermatol. 2014 Jan;150(1):79-81. doi: 10.1001/jamadermatol.2013.5322. Erratum in: JAMA Dermatol. 2014 Mar;150(3):337. PMID: 24196118. Link <https://pubmed.ncbi.nlm.nih.gov/24196118/>
636. Gómez-Camarasa C, Rojo-Martín MD, Miranda-Casas C, Alastruey-Izquierdo A, Aliaga-Martínez L, Labrador-Molina JM, Navarro-Marí JM. Disseminated infection due to Saksenaea vasiformis secondary to cutaneous mucormycosis. Mycopathologia. 2014 Feb;177(1-2):97-101. doi: 10.1007/s11046-013-9715-3. Epub 2013 Nov 1. PMID: 24178374. Link <https://pubmed.ncbi.nlm.nih.gov/24178374/>
637. Singh V, Singh M, Joshi C, Sangwan J. Rhinocerebral mucormycosis in a patient with type 1 diabetes presenting as toothache: a case report from Himalayan region of India. BMJ Case Rep. 2013 Oct 30;2013:bcr2013200811. doi: 10.1136/bcr-2013-200811. PMID: 24172773; PMCID: PMC3822077. LINK <https://pubmed.ncbi.nlm.nih.gov/24172773/>
638. Verma R, Nair V, Vasudevan B, Vijendran P, Behera V, Neema S. Rare case of primary cutaneous mucormycosis of the hand caused by Rhizopus microsporus in an immunocompetent patient. Int J Dermatol. 2014 Jan;53(1):66-9. doi: 10.1111/ijd.12204. Epub 2013 Oct 29. PMID: 24168663. Link <https://pubmed.ncbi.nlm.nih.gov/24168663/>
639. Poirier P, Nourrisson C, Gibold L, Chalus E, Guelon D, Descamp S, Traore O, Cambon M, Aumeran C. Three cases of cutaneous mucormycosis with Lichtheimia spp. (ex Absidia/Mycocladus) in ICU. Possible cross-transmission in an intensive care unit between 2 cases. J Mycol Med. 2013 Dec;23(4):265-9. doi: 10.1016/j.mycmed.2013.09.002. Epub 2013 Oct 18. PMID: 24139734. Link <https://pubmed.ncbi.nlm.nih.gov/24139734/>
640. Dwarakanath S, Kumar V, Blackburn J, Castresana MR. A rare case of multi-chambered fungal endocarditis from a virulent Cunninghamella infection. Eur Heart J. 2014 Feb;35(6):343. doi: 10.1093/eurheartj/eht444. Epub 2013 Oct 17. PMID: 24135836. Link <https://pubmed.ncbi.nlm.nih.gov/24135836/>
641. Corey KE, Gupta NK, Agarwal S, Xiao HD. Case records of the Massachusetts General Hospital. Case 32-2013. A 55-year-old woman with autoimmune hepatitis, cirrhosis, anorexia, and abdominal pain. N Engl J Med. 2013 Oct 17;369(16):1545-53. doi: 10.1056/NEJMcpc1208153. PMID: 24131180. Link <https://pubmed.ncbi.nlm.nih.gov/24131180/>
642. Bhaskar N, Jagana R, Johnson LG. Nontuberculous empyema necessitatis. Am J Respir Crit Care Med. 2013 Oct 15;188(8):e65-6. doi: 10.1164/rccm.201301-0093IM. PMID: 24127812. Link <https://pubmed.ncbi.nlm.nih.gov/24127812/>
643. Ye B, Yu D, Zhang X, Shao K, Chen D, Wu D, Zhang Y, Zhou Y, Shen Y, Yu Q. Disseminated Rhizopus microsporus infection following allogeneic hematopoietic stem cell transplantation in a child with severe aplastic anemia. Transpl Infect Dis. 2013 Dec;15(6):E216-23. doi: 10.1111/tid.12144. Epub 2013 Oct 14. PMID: 24119033. Link <https://pubmed.ncbi.nlm.nih.gov/24119033/>
644. Qu LF, Yang J, Wu XW, Xing XQ. Endobronchial mucormycosis showing as a bronchial tumor. Chin Med J (Engl). 2013;126(19):3620. PMID: 24112152. Link https://pubmed.ncbi.nlm.nih.gov/24112152/
645. Irtan S, Lamerain M, Lesage F, Verkarre V, Bougnoux ME, Lanternier F, Zahar JR, Salvi N, Talbotec C, Lortholary O, Lacaille F, Chardot C. Mucormycosis as a rare cause of severe gastrointestinal bleeding after multivisceral transplantation. Transpl Infect Dis. 2013 Dec;15(6):E235-8. doi: 10.1111/tid.12147. Epub 2013 Sep 18. PMID: 24103142. Link <https://pubmed.ncbi.nlm.nih.gov/24103142/>
646. Hirayama Y, Yajima N, Kaimori M, Akagi T, Kubo K, Saito D, Hizawa Y, Wada R, Yagihashi S. Disseminated infection and pulmonary embolization of Cunninghamella bertholletiae complicated with hemophagocytic lymphohistiocytosis. Intern Med. 2013;52(19):2275-9. doi: 10.2169/internalmedicine.52.0171. Epub 2012 Mar 1. PMID: 24088766. Link <https://pubmed.ncbi.nlm.nih.gov/24088766/>
647. Labbadia F, Sanchez-Salinas A. Invasive mucormycosis without radiological alterations. Blood. 2013 Aug 8;122(6):862. doi: 10.1182/blood-2013-02-478446. PMID: 24079015. Link <https://pubmed.ncbi.nlm.nih.gov/24079015/>
648. Vijayabala GS, Annigeri RG, Sudarshan R. Mucormycosis in a diabetic ketoacidosis patient. Asian Pac J Trop Biomed. 2013 Oct;3(10):830-3. doi: 10.1016/S2221-1691(13)60164-1. Epub 2013 Sep 4. PMID: 24075351; PMCID: PMC3761145. Link <https://pubmed.ncbi.nlm.nih.gov/24075351/>
649. Abela L, Toelle SP, Hackenberg A, Scheer I, Güngör T, Plecko B. Fatal outcome of rhino-orbital-cerebral mucormycosis due to bilateral internal carotid occlusion in a child after hematopoietic stem cell transplantation. Pediatr Infect Dis J. 2013 Oct;32(10):1149-50. doi: 10.1097/INF.0b013e31829e69e7. PMID: 24067555. Link <https://pubmed.ncbi.nlm.nih.gov/24067555/>
650. Agrawal P, Saikia U, Ramanaathan S, Samujh R. Neonatal small intestinal zygomyocosis misdiagnosed as intussusception in a two-day-old child with a review of the literature. Fetal Pediatr Pathol. 2013 Dec;32(6):418-21. doi: 10.3109/15513815.2013.789948. Epub 2013 Sep 17. PMID: 24044628. Link <https://pubmed.ncbi.nlm.nih.gov/24044628/>
651. Pan AS, Srinath L. Mucormycosis in a patient with AIDS receiving systemic steroids. J Am Osteopath Assoc. 2013 Sep;113(9):708-11. doi: 10.7556/jaoa.2013.037. PMID: 24005091. Link <https://pubmed.ncbi.nlm.nih.gov/24005091/>
652. Nagakawa H, Igari H, Konishi K, Kashizaki F, Aoyama M, Watanabe A, Tatsumi K, Kamei K. [An autopsy case of tension pneumothorax due to the rupture of intrapulmonary cavity by mucormycosis during treatment with a ventilator]. Med Mycol J. 2013;54(3):285-9. Japanese. doi: 10.3314/mmj.54.285. PMID: 23995418. Link <https://pubmed.ncbi.nlm.nih.gov/23995418/>
653. Couldwell WT, MacDonald JD, Taussky P. Complete resection of the cavernous sinus-indications and technique. World Neurosurg. 2014 Dec;82(6):1264-70. doi: 10.1016/j.wneu.2013.08.026. Epub 2013 Aug 29. PMID: 23994071. Link <https://pubmed.ncbi.nlm.nih.gov/23994071/>
654. Mahadevaiah AH, Rajagopalan N, Patil M, C S. Coinfection of pulmonary mucormycosis and aspergillosis presenting as bilateral vocal cord palsy. BMJ Case Rep. 2013 Aug 20;2013:bcr2013009615. doi: 10.1136/bcr-2013-009615. PMID: 23964036; PMCID: PMC3761694. Link <https://pubmed.ncbi.nlm.nih.gov/23964036/>
655. Lee HJ, Kwon JC, Kim SH, Choi SM, Lee DG, Park SH, Choi JH, Yoo JH, Cho BS, Lee S, Kim HJ, Min CK, Lee JW, Min WS. Posaconazole treatment in Korea: single-center experience over 5 years. Yonsei Med J. 2013 Sep;54(5):1234-40. doi: 10.3349/ymj.2013.54.5.1234. PMID: 23918575; PMCID: PMC3743190. Link <https://pubmed.ncbi.nlm.nih.gov/23918575/>
656. Guymer C, Khurana S, Suppiah R, Hennessey I, Cooper C. Successful treatment of disseminated mucormycosis in a neutropenic patient with T-cell acute lymphoblastic leukaemia. BMJ Case Rep. 2013 Jul 31;2013:bcr2013009577. doi: 10.1136/bcr-2013-009577. PMID: 23904418; PMCID: PMC3736398. Link <https://pubmed.ncbi.nlm.nih.gov/23904418/>
657. Echaiz JF, Burnham CA, Bailey TC. A case of Apophysomyces trapeziformis necrotizing soft tissue infection. Int J Infect Dis. 2013 Dec;17(12):e1240-2. doi: 10.1016/j.ijid.2013.06.008. Epub 2013 Jul 26. PMID: 23891642. Link <https://pubmed.ncbi.nlm.nih.gov/23891642/>
658. Carceller F, Oñoro G, Buitrago MJ, Herrero B, Lassaletta Á, Pérez-Martínez A, González-Vicent M, Madero L. Cunninghamella bertholletiae infection in children: review and report of 2 cases with disseminated infection. J Pediatr Hematol Oncol. 2014 Mar;36(2):e109-14. doi: 10.1097/MPH.0b013e31829eec5a. PMID: 23887023. Link <https://pubmed.ncbi.nlm.nih.gov/23887023/>
659. Sunagawa K, Ishige T, Kusumi Y, Asano M, Nisihikawa E, Kato M, Yagasaki H, Nemoto N. Renal abscess involving mucormycosis by immunohistochemical detection in a patient with acute lymphocytic leukemia: a case report and literature review. Jpn J Infect Dis. 2013;66(4):345-7. doi: 10.7883/yoken.66.345. PMID: 23883851. Link <https://pubmed.ncbi.nlm.nih.gov/23883851/>
660. Angeline, Hanifah M, Balachandran G, Rajesh NG. Subcutaneous mucor zygomycosis with potential life-threatening visceral complication. Indian J Med Microbiol. 2013 Apr-Jun;31(2):182-4. doi: 10.4103/0255-0857.115226. PMID: 23867678. Link <https://pubmed.ncbi.nlm.nih.gov/23867678/>
661. Asimakopoulos P, Supriya M, Kealey S, Vernham GA. A case-based discussion on a patient with non-otogenic fungal skull base osteomyelitis: pitfalls in diagnosis. J Laryngol Otol. 2013 Aug;127(8):817-21. doi: 10.1017/S002221511300145X. Epub 2013 Jul 18. PMID: 23866727. Link <https://pubmed.ncbi.nlm.nih.gov/23866727/>
662. Kazak E, Aslan E, Akalın H, Saraydaroğlu O, Hakyemez B, Erişen L, Yazıcı B, Gürcüoğlu E, Yılmaz E, Ener B, Helvacı S. A mucormycosis case treated with a combination of caspofungin and amphotericin B. J Mycol Med. 2013 Sep;23(3):179-84. doi: 10.1016/j.mycmed.2013.06.003. Epub 2013 Jul 12. PMID: 23856448. Link <https://pubmed.ncbi.nlm.nih.gov/23856448/>
663. Strasfeld L, Espinosa-Aguilar L, Gajewski JL, Stenzel P, Pimentel A, Mater E, Maziarz RT. Emergence of Cunninghamella as a pathogenic invasive mold infection in allogeneic transplant recipients. Clin Lymphoma Myeloma Leuk. 2013 Oct;13(5):622-8. doi: 10.1016/j.clml.2013.05.002. Epub 2013 Jul 10. PMID: 23850285. Link <https://pubmed.ncbi.nlm.nih.gov/23850285/>
664. Zaki SM, Elkholy IM, Elkady NA, Abdel-Ghany K. Mucormycosis in Cairo, Egypt: review of 10 reported cases. Med Mycol. 2014 Jan;52(1):73-80. doi: 10.3109/13693786.2013.809629. PMID: 23848229. Link <https://pubmed.ncbi.nlm.nih.gov/23848229/>
665. Sanavi S, Afshar R, Afshin-Majd S. Rhino-orbitocerebral mucormycosis in a patient with idiopathic crescentic glomerulonephritis. Saudi J Kidney Dis Transpl. 2013 Jul;24(4):768-72. doi: 10.4103/1319-2442.113878. PMID: 23816728. Link <https://pubmed.ncbi.nlm.nih.gov/23816728/>
666. Do GW, Jung SW, Jun JB, Seo JH, Nah YW. Colonic mucormycosis presented with ischemic colitis in a liver transplant recipient. World J Gastroenterol. 2013 Jun 14;19(22):3508-11. doi: 10.3748/wjg.v19.i22.3508. PMID: 23801847; PMCID: PMC3683693. Link <https://pubmed.ncbi.nlm.nih.gov/23801847/>
667. Azari AA, Nehls S, Ghoghawala SY, Lee V, Kanavi MR, Potter HD. Rhizopus keratitis following corneal trauma. JAMA Ophthalmol. 2013 Jun;131(6):776. doi: 10.1001/2013.jamaophthalmol.380. PMID: 23764702; PMCID: PMC4435688. Link <https://pubmed.ncbi.nlm.nih.gov/23764702/>
668. Zanation A, Fleischman D, Chavala SH. Ptosis, erythema, and rapidly decreasing vision. JAMA. 2013 Jun 12;309(22):2382-3. doi: 10.1001/jama.2013.5517. PMID: 23757086. Link <https://pubmed.ncbi.nlm.nih.gov/23757086/>
669. Chaari A, Ghadoun H, Ben Algia N, Bahloul M, Bouaziz M. La mucormycose : une cause rare d'exophtalmie unilatérale [Mucormycosis: an unusual cause of unilateral exophthalmia]. J Mycol Med. 2013 Jun;23(2):140-3. French. doi: 10.1016/j.mycmed.2013.04.008. Epub 2013 May 28. PMID: 23721996. Link <https://pubmed.ncbi.nlm.nih.gov/23721996/>
670. Erami M, Shams-Ghahfarokhi M, Jahanshiri Z, Sharif A, Razzaghi-Abyaneh M. Rhinocerebral mucormycosis due to Rhizopus oryzae in a diabetic patient: a case report. J Mycol Med. 2013 Jun;23(2):123-9. doi: 10.1016/j.mycmed.2013.04.002. Epub 2013 May 27. PMID: 23721995. Link <https://pubmed.ncbi.nlm.nih.gov/23721995/>
671. Herford AS, Tandon R, Pivetti L, Cicciù M. Closure of large palatal defect using a tongue flap. J Craniofac Surg. 2013 May;24(3):875-7. doi: 10.1097/SCS.0b013e318285d474. PMID: 23714900. Link <https://pubmed.ncbi.nlm.nih.gov/23714900/>
672. Ram R, Swarnalatha G, Naidu GD, Kaligotla DV. Multiple ring enhancing lesions in brain due to disseminated Zygomycosis in a renal transplant recipient. Nephrology (Carlton). 2013 Jun;18(6):479-480. doi: 10.1111/nep.12069. PMID: 23710934. Link <https://pubmed.ncbi.nlm.nih.gov/23710934/>
673. Elguazzar S, Benouachane T, Nasri A, Malihy A, Tligui H, Bentahila A. Mucormycose cutanée ilio-fémorale avec extension endo-pelvienne chez un enfant immunocompétent [Iliofemoral cutaneous mucormycosis with endopelvic extension in an immunocompetent child]. Arch Pediatr. 2013 Jul;20(7):754-7. French. doi: 10.1016/j.arcped.2013.04.003. Epub 2013 May 22. PMID: 23706611. Link <https://pubmed.ncbi.nlm.nih.gov/23706611/>
674. Okubo Y, Ishiwatari T, Izumi H, Sato F, Aki K, Sasai D, Ando T, Shinozaki M, Natori K, Tochigi N, Wakayama M, Hata Y, Nakayama H, Nemoto T, Shibuya K. Pathophysiological implication of reversed CT halo sign in invasive pulmonary mucormycosis: a rare case report. Diagn Pathol. 2013 May 17;8:82. doi: 10.1186/1746-1596-8-82. PMID: 23683872; PMCID: PMC3658989. Link <https://pubmed.ncbi.nlm.nih.gov/23683872/>
675. Ananthaneni AR, Undavalli SB, Velagapudi RP, Guduru VS. Mucormycosis: an atrocious mate of patients with diabetes. BMJ Case Rep. 2013 May 2;2013:bcr2013009600. doi: 10.1136/bcr-2013-009600. PMID: 23645655; PMCID: PMC3669953. Link <https://pubmed.ncbi.nlm.nih.gov/23645655/>
676. Li H, Hwang SK, Zhou C, Du J, Zhang J. Gangrenous cutaneous mucormycosis caused by Rhizopus oryzae: a case report and review of primary cutaneous mucormycosis in China over Past 20 years. Mycopathologia. 2013 Aug;176(1-2):123-8. doi: 10.1007/s11046-013-9654-z. Epub 2013 Apr 25. PMID: 23615822. Link <https://pubmed.ncbi.nlm.nih.gov/23615822/>
677. LeBlanc RE, Meriden Z, Sutton DA, Thompson EH, Neofytos D, Zhang SX. Cunninghamella echinulata causing fatally invasive fungal sinusitis. Diagn Microbiol Infect Dis. 2013 Aug;76(4):506-9. doi: 10.1016/j.diagmicrobio.2013.03.009. Epub 2013 Apr 18. PMID: 23602784. Link <https://pubmed.ncbi.nlm.nih.gov/23602784/>
678. Pozo-Laderas JC, Pontes-Moreno A, Robles-Arista JC, Bautista-Rodriguez MD, Candau-Alvarez A, Caro-Cuenca MT, Linares-Sicilia MJ. Infección fúngica invasiva mixta por Rhizomucor pusillus y Aspergillus niger en un paciente inmunocompetente [Mixed invasive fungal infection due to Rhizomucor pusillus and Aspergillus niger in an immunocompetent patient]. Rev Iberoam Micol. 2015 Jan-Mar;32(1):46-50. Spanish. doi: 10.1016/j.riam.2013.03.002. Epub 2013 Apr 11. PMID: 23583263. Link <https://pubmed.ncbi.nlm.nih.gov/23583263/>
679. Lord JD, Chen J, Kozarek RA. A case of fatal idiopathic enteritis and multiple opportunistic infections associated with dendritic cell deficiencies. J Gastrointestin Liver Dis. 2013 Mar;22(1):87-91. PMID: 23539396; PMCID: PMC4366003. Link <https://pubmed.ncbi.nlm.nih.gov/23539396/>
680. Sanz-Bueno J, Castellanos-González M, Rodríguez-Peralto JL, Rivera R. Zigomicosis diseminada en una paciente con leucemia linfática crónica [Disseminated zygomycosis in a patient with chronic lymphocytic leukemia]. Med Clin (Barc). 2013 Jun 4;140(11):e21. Spanish. doi: 10.1016/j.medcli.2013.01.040. Epub 2013 Mar 26. PMID: 23537740. Link <https://pubmed.ncbi.nlm.nih.gov/23537740/>
681. Liu Y, Wu H, Huang F, Fan Z, Xu B. Utility of 18F-FDG PET/CT in diagnosis and management of mucormycosis. Clin Nucl Med. 2013 Sep;38(9):e370-1. doi: 10.1097/RLU.0b013e3182867d13. PMID: 23531773. Link <https://pubmed.ncbi.nlm.nih.gov/23531773/>
682. Chakravarti A, Bhargava R, Bhattacharya S. Cutaneous mucormycosis of nose and facial region in children: a case series. Int J Pediatr Otorhinolaryngol. 2013 May;77(5):869-72. doi: 10.1016/j.ijporl.2013.02.025. Epub 2013 Mar 23. PMID: 23528899. Link <https://pubmed.ncbi.nlm.nih.gov/23528899/>
683. Trabelsi S, Marrakchi J, Aloui D, Lahiani R, Sellami A, Ben Salah M, Ferjaoui M, Khaled S. La mucormycose rhino-orbito-cérébrale [Rhino-orbito-cerebral mucormycosis]. Tunis Med. 2013 Feb;91(2):164-5. French. PMID: 23526289. Link <https://pubmed.ncbi.nlm.nih.gov/23526289/>
684. Kim ST, Kim WS, Lee HH, Kim JY. Successful treatment of invasive rhinopulmonary mucormycosis with an indolent presentation by combined medical and surgical therapy. J Craniofac Surg. 2013 Mar;24(2):e182-4. doi: 10.1097/SCS.0b013e318280196b. PMID: 23524831. Link <https://pubmed.ncbi.nlm.nih.gov/23524831/>
685. Gen R, Horasan EŞ, Vaysoğlu Y, Arpaci RB, Ersöz G, Özcan C. Rhino-orbito-cerebral mucormycosis in patients with diabetic ketoacidosis. J Craniofac Surg. 2013 Mar;24(2):e144-7. doi: 10.1097/SCS.0b013e31827c7eb8. PMID: 23524816. Link <https://pubmed.ncbi.nlm.nih.gov/23524816/>
686. Razouk S, Sebbani S, Agoumi A, Benouchen T, Malihi A, Nacir A, Abouhafsse A, Al Hamany Z, Tligui H. Les mucormycoses sous-cutanées àLichtheimia corymbifera : à propos d'un cas chez un enfant immunocompétent [The subcutaneous mucormycosis due to Lichtheimia corymbifera: A case report in an immunocompetent child]. J Mycol Med. 2012 Jun;22(2):185-8. French. doi: 10.1016/j.mycmed.2012.01.006. Epub 2012 May 23. PMID: 23518022. Link <https://pubmed.ncbi.nlm.nih.gov/23518022/>
687. Pourahmad M, Sepidkar A, Farokhnia MH, Tadayon SM, Salehi H, Zabetian H. Mucormycosis after scorpion sting: case report. Mycoses. 2013 Sep;56(5):589-91. doi: 10.1111/myc.12066. Epub 2013 Mar 18. PMID: 23496060. Lionk <https://pubmed.ncbi.nlm.nih.gov/23496060/>
688. Hallur V, Singh G, Rudramurthy SM, Kapoor R, Chakrabarti A. Demodex mite infestation of unknown significance in a patient with rhinocerebral mucormycosis due to Apophysomyces elegans species complex. J Med Microbiol. 2013 Jun;62(Pt 6):926-928. doi: 10.1099/jmm.0.054338-0. Epub 2013 Mar 8. PMID: 23475907. Link <https://pubmed.ncbi.nlm.nih.gov/23475907/>
689. Rodrigues S, Santos L, Nuak J, Pardal J, Sarmento JA, Macedo G. Colonic mucormycosis. Endoscopy. 2013;45 Suppl 2 UCTN:E20. doi: 10.1055/s-0032-1326110. Epub 2013 Mar 6. PMID: 23468148. Link <https://pubmed.ncbi.nlm.nih.gov/23468148/>
690. Marutsuka T, Masuda Y, Saishoji T. [Resected case of pulmonary mucormycosis]. Kyobu Geka. 2013 Mar;66(3):223-6. Japanese. PMID: 23445649. Link <https://pubmed.ncbi.nlm.nih.gov/23445649/>
691. Almannai M, Imran H, Estrada B, Siddiqui AH. Successful treatment of rhino-orbital mucormycosis with posaconazole and hyperbaric oxygen therapy. Pediatr Hematol Oncol. 2013 Apr;30(3):184-6. doi: 10.3109/08880018.2013.770587. Epub 2013 Feb 27. PMID: 23444832. Link <https://pubmed.ncbi.nlm.nih.gov/23444832/>
692. Ribeiro EF, dos Santos VM, Paixão GT, Cruz LR, Danilow MZ, Campos VF. Mucormycosis in a patient with acute myeloid leukemia successfully treated with liposomal amphotericin B associated with deferasirox and hyperbaric oxygen. Mycopathologia. 2013 Apr;175(3-4):295-300. doi: 10.1007/s11046-013-9629-0. Epub 2013 Feb 27. PMID: 23443451. Link <https://pubmed.ncbi.nlm.nih.gov/23443451/>
693. Ozkaya-Parlakay A, Karadag-Oncel E, Cengiz AB, Kara A, Yigit A, Gucer S, Gur D. Trichosporon asahii sepsis in a patient with pediatric malignancy. J Microbiol Immunol Infect. 2016 Feb;49(1):146-9. doi: 10.1016/j.jmii.2013.01.003. Epub 2013 Feb 16. PMID: 23419599. Link <https://pubmed.ncbi.nlm.nih.gov/23419599/>
694. Radhakrishnan N, Yadav SP, Oberoi J, Kulshreshta R, Bhalla S, Sachdeva A. Intestinal mucormycosis: a rare entity in pediatric oncology. Pediatr Hematol Oncol. 2013 Apr;30(3):178-83. doi: 10.3109/08880018.2013.769286. Epub 2013 Feb 14. PMID: 23410194. Link <https://pubmed.ncbi.nlm.nih.gov/23410194/>
695. Bergantim R, Rios E, Trigo F, Guimarães JE. Invasive coinfection with Aspergillus and Mucor in a patient with acute myeloid leukemia. Clin Drug Investig. 2013 Feb;33 Suppl 1:S51-5. doi: 10.1007/s40261-012-0022-4. PMID: 23381986. Link <https://pubmed.ncbi.nlm.nih.gov/23381986/>
696. Pacheco P, Ventura AS, Branco T, Gonçalves L, Carvalho C. Clinical experience in invasive fungal infections. Clin Drug Investig. 2013 Feb;33 Suppl 1:S23-6. doi: 10.1007/s40261-012-0017-1. PMID: 23381980. Link <https://pubmed.ncbi.nlm.nih.gov/23381980/>
697. Badior M, Trigo F, Eloy C, Guimarães JE. Mucor infection: difficult diagnosis. Clin Drug Investig. 2013 Feb;33 Suppl 1:S19-21. doi: 10.1007/s40261-012-0014-4. PMID: 23381979. Link <https://pubmed.ncbi.nlm.nih.gov/23381979/>
698. Rahman A, Akter K, Hossain S, Rashid HU. Rhino-orbital mucourmycosis in a non-immunocompromised patient. BMJ Case Rep. 2013 Feb 6;2013:bcr2012007863. doi: 10.1136/bcr-2012-007863. PMID: 23391952; PMCID: PMC3604437. Link <https://pubmed.ncbi.nlm.nih.gov/23391952/>
699. Rahman A, Akter K, Hossain S, Rashid HU. Rhino-orbital mucourmycosis in a non-immunocompromised patient. BMJ Case Rep. 2013 Feb 6;2013:bcr2012007863. doi: 10.1136/bcr-2012-007863. PMID: 23391952; PMCID: PMC3604437. Link <https://pubmed.ncbi.nlm.nih.gov/23391952/>
700. Teixeira CA, Medeiros PB, Leushner P, Almeida F. Rhinocerebral mucormycosis: literature review apropos of a rare entity. BMJ Case Rep. 2013 Feb 5;2013:bcr2012008552. doi: 10.1136/bcr-2013-008552. PMID: 23389725; PMCID: PMC3603946. Link <https://pubmed.ncbi.nlm.nih.gov/23389725/>
701. Schofield C, Stern A, Jevtic A. Disseminated zygomycosis due to Mycocladus corymbifera with cutaneous and cerebral involvement. Australas J Dermatol. 2013 Feb;54(1):e8-11. doi: 10.1111/j.1440-0960.2011.00752.x. Epub 2011 May 5. PMID: 23373895. Link <https://pubmed.ncbi.nlm.nih.gov/23373895/>
702. Derkaoui A, Khatouf M. La mucormycose rhino-orbito-cérébrale [Rhino-orbito-cerebral mucormycosis]. Pan Afr Med J. 2012;13:69. French. Epub 2012 Nov 29. PMID: 23396825; PMCID: PMC3567418. Link <https://pubmed.ncbi.nlm.nih.gov/23396825/>
703. Goel P, Jain V, Sengar M, Mohta A, Das P, Bansal P. Gastrointestinal mucormycosis: a success story and appraisal of concepts. J Infect Public Health. 2013 Feb;6(1):58-61. doi: 10.1016/j.jiph.2012.08.004. Epub 2012 Nov 16. PMID: 23290094. Link <https://pubmed.ncbi.nlm.nih.gov/23290094/>
704. Erbey F, Kocabaş E, Bayram İ, Soyupak S, Gümürdülü D, Tanyeli A. Pediatric invasive mucormycosis cured with high dose liposomal amphotericin B. Tuberk Toraks. 2012;60(4):375-9. doi: 10.5578/tt.2364. PMID: 23289469. Link <https://pubmed.ncbi.nlm.nih.gov/23289469/>
705. Webb BJ, Blair JE, Kusne S, Scott RL, Steidley DE, Arabia FA, Vikram HR. Concurrent pulmonary Aspergillus fumigatus and mucor infection in a cardiac transplant recipient: a case report. Transplant Proc. 2013 Mar;45(2):792-7. doi: 10.1016/j.transproceed.2012.03.056. Epub 2012 Sep 15. PMID: 23267784. Link <https://pubmed.ncbi.nlm.nih.gov/23267784/>
706. Xia ZK, Wang WL, Yang RY. Slowly progressive cutaneous, rhinofacial, and pulmonary mucormycosis caused by Mucor irregularis in an immunocompetent woman. Clin Infect Dis. 2013 Apr;56(7):993-5. doi: 10.1093/cid/cis1045. Epub 2012 Dec 14. PMID: 23243187. Link <https://pubmed.ncbi.nlm.nih.gov/23243187/>
707. Murphy AD, Williamson PA, Vesely M. Reconstruction of an extensive peri-orbital defect secondary to mucormycosis in a patient with myelodysplasia. J Plast Reconstr Aesthet Surg. 2013 Mar;66(3):e69-71. doi: 10.1016/j.bjps.2012.11.032. Epub 2012 Dec 8. PMID: 23232448. Link <https://pubmed.ncbi.nlm.nih.gov/23232448/>
708. Odronic SI, Scheidemantel T, Tuohy MJ, Chute D, Procop GW, Booth CN. Two cases of Cokeromyces recurvatus in liquid-based Papanicolaou tests and a review of the literature. Arch Pathol Lab Med. 2012 Dec;136(12):1593-6. doi: 10.5858/arpa.2011-0493-CR. PMID: 23194052. Link <https://pubmed.ncbi.nlm.nih.gov/23194052/>
709. Eickhardt S, Braendstrup P, Clasen-Linde E, Jensen KE, Alhede M, Bjarnsholt T, Høiby N, Vindeløv L, Moser C. A non-fatal case of invasive zygomycete (Lichtheimia corymbifera) infection in an allogeneic haematopoietic cell transplant recipient. APMIS. 2013 May;121(5):456-9. doi: 10.1111/apm.12008. Epub 2012 Nov 23. PMID: 23176289. Link <https://pubmed.ncbi.nlm.nih.gov/23176289/>
710. Zhang L, Tian X, Wang P, Zhang H, Feng R. Recurrent pulmonary mucormycosis after lobectomy in a non-smoking patient without predisposing risk factors. Braz J Infect Dis. 2012 Nov-Dec;16(6):590-3. doi: 10.1016/j.bjid.2012.10.005. Epub 2012 Nov 8. PMID: 23141973. Link <https://pubmed.ncbi.nlm.nih.gov/23141973/>
711. Dusart A, Duprez T, Van Snick S, Godfraind C, Sindic C. Fatal rhinocerebral mucormycosis with intracavernous carotid aneurysm and thrombosis: a late complication of transsphenoidal surgery? Acta Neurol Belg. 2013 Jun;113(2):179-84. doi: 10.1007/s13760-012-0151-9. Epub 2012 Nov 8. PMID: 23135781. Link <https://pubmed.ncbi.nlm.nih.gov/23135781/>
712. Scully MA, Yeaney GA, Compton ML, Berg MJ. SWAN MRI revealing multiple microhemorrhages secondary to septic emboli from mucormycosis. Neurology. 2012 Oct 30;79(18):1932-3. doi: 10.1212/WNL.0b013e318271f86c. PMID: 23109654. Link <https://pubmed.ncbi.nlm.nih.gov/23109654/>
713. Munoz J, Hughes A, Guo Y. Mucormycosis-associated intracranial hemorrhage. Blood Coagul Fibrinolysis. 2013 Jan;24(1):100-1. doi: 10.1097/MBC.0b013e32835a72df. PMID: 23103724. Link <https://pubmed.ncbi.nlm.nih.gov/23103724/>
714. González-Abad MJ, Alonso Sanz M. Zygomycosis in children: disseminated infection caused by Cunninghamella bertholletiae. Arch Bronconeumol. 2013 Jan;49(1):35. English, Spanish. doi: 10.1016/j.arbres.2012.04.023. Epub 2012 Oct 23. PMID: 23099279. Link <https://pubmed.ncbi.nlm.nih.gov/23099279/>
715. Schneidawind D, Nann D, Vogel W, Faul C, Fend F, Horger M, Kanz L, Bethge W. Allogeneic hematopoietic cell transplantation in patients with acute myeloid leukemia and pulmonary mucormycosis. Transpl Infect Dis. 2012 Dec;14(6):E166-72. doi: 10.1111/tid.12019. Epub 2012 Oct 17. PMID: 23075207. Link <https://pubmed.ncbi.nlm.nih.gov/23075207/>
716. Abu El-Naaj I, Leiser Y, Wolff A, Peled M. The surgical management of rhinocerebral mucormycosis. J Craniomaxillofac Surg. 2013 Jun;41(4):291-5. doi: 10.1016/j.jcms.2012.03.019. Epub 2012 Oct 9. PMID: 23058177. Link <https://pubmed.ncbi.nlm.nih.gov/23058177/>
717. Arena V, De-Giorgio F, Pennacchia I, Manna R, Vetrugno G, Stigliano E, Milic N, Gasbarrini G, Abenavoli L. Haemophagocytic syndrome associated with mucormycosis infection. Int J Immunopathol Pharmacol. 2012 Jul-Sep;25(3):751-5. doi: 10.1177/039463201202500321. PMID: 23058025. Link <https://pubmed.ncbi.nlm.nih.gov/23058025/>
718. Turan MN, Tatar E, Yaprak M, Arda B, Kitiş Ö, Metin DY, Hoşcoşkun C, Töz H. A mucormycosis case presented with orbital apex syndrome and hemiplegia in a renal transplant patient. Int Urol Nephrol. 2013 Dec;45(6):1815-9. doi: 10.1007/s11255-012-0295-9. Epub 2012 Sep 30. PMID: 23054315. Link <https://pubmed.ncbi.nlm.nih.gov/23054315/>
719. Vashi N, Avedian R, Brown J, Arai S. Successful surgical and medical treatment of rhizopus osteomyelitis following hematopoietic cell transplantation. Orthopedics. 2012 Oct;35(10):e1556-61. doi: 10.3928/01477447-20120919-30. PMID: 23027498. Link <https://pubmed.ncbi.nlm.nih.gov/23027498/>
720. Abboud CS, Bergamasco MD, Baía CE, Lallée MP, Zan AS, Zamorano MM, Pereira OI, Mies S. Case report of hepatic mucormycosis after liver transplantation: successful treatment with liposomal amphotericin B followed by posaconazole sequential therapy. Transplant Proc. 2012 Oct;44(8):2501-2. doi: 10.1016/j.transproceed.2012.07.042. PMID: 23026630. Link <https://pubmed.ncbi.nlm.nih.gov/23026630/>
721. Chaaban MR, Bell W, Woodworth BA. Invasive mucormycosis in an immunocompetent patient with allergic fungal rhinosinusitis. Otolaryngol Head Neck Surg. 2013 Jan;148(1):174-5. doi: 10.1177/0194599812460988. Epub 2012 Sep 24. PMID: 23008329. Link <https://pubmed.ncbi.nlm.nih.gov/23008329/>
722. Irga N, Kosiak W, Jaworski R, Komarnicka J, Birkholz D. Hyperthyroidism secondary to disseminated mucormycosis in a child with acute lymphoblastic leukemia: case report and a review of published reports. Mycopathologia. 2013 Feb;175(1-2):123-7. doi: 10.1007/s11046-012-9584-1. Epub 2012 Sep 25. PMID: 23007613; PMCID: PMC3550700. Link <https://pubmed.ncbi.nlm.nih.gov/23007613/>
723. Taj-Aldeen SJ, Falamarzi A, AlMuzrkchi A, Guarro J. Rare pediatric rhino-orbital infection caused by Saksenaea vasiformis. Infection. 2012 Dec;40(6):703-7. doi: 10.1007/s15010-012-0338-2. Epub 2012 Sep 24. PMID: 23001544. Link <https://pubmed.ncbi.nlm.nih.gov/23001544/>
724. Trasmonte MV, Jiménez JD, Santiago MÁ, Gálvez E, Jerez V, Pérez D, Robles M, Farje VK, Martínez P, Nieto P, Rubio JA. Association of topical amphotericin B lipid complex treatment to standard therapy for rhinomaxillary mucormycosis after liver transplantation: a case report. Transplant Proc. 2012 Sep;44(7):2120-3. doi: 10.1016/j.transproceed.2012.07.081. PMID: 22974930. Link <https://pubmed.ncbi.nlm.nih.gov/22974930/>
725. Johnson JB, Affolter KE, Samadder NJ. A rare cause of hematochezia: colon mucormycosis. Clin Gastroenterol Hepatol. 2013 Aug;11(8):A22. doi: 10.1016/j.cgh.2012.08.025. Epub 2012 Aug 28. PMID: 22935264. Link <https://pubmed.ncbi.nlm.nih.gov/22935264/>
726. Bahloul M, Tounsi A, Chaari A, Ben Aljia N, Ammar R, Chelly H, Bouaziz M. Mucormycose rhinocérébrale compliquée d'un syndrome d'activation macrophagique: une observation Tunisienne [Rhinocerebral mucormycosis complicated by hemophagocytic lymphohistiocytosis: case report from Tunisia]. Med Sante Trop. 2012 Apr-Jun;22(2):210-2. French. doi: 10.1684/mst.2012.0049. PMID: 22907991. Link <https://pubmed.ncbi.nlm.nih.gov/22907991/>
727. Galasso S, Chilelli NC, Burlina S, Vitturi N, Avogaro A. Palpebral ptosis, fixed mydriasis and exophthalmos: an uncommon presentation of new diabetes mellitus. Intern Emerg Med. 2013 Feb;8(1):89-91. doi: 10.1007/s11739-012-0842-5. Epub 2012 Aug 21. PMID: 22907808. Link <https://pubmed.ncbi.nlm.nih.gov/22907808/>
728. Gurevich M, Levi I, Steinberg R, Shonfeld T, Shapiro R, Israeli M, Sprecher H, Shalit I, Mor E. Mucormycosis in a liver allograft: salvage re-transplantation and targeted immunosuppressive management. Transpl Infect Dis. 2012 Oct;14(5):E97-101. doi: 10.1111/j.1399-3062.2012.00776.x. Epub 2012 Aug 16. PMID: 22897560. Link <https://pubmed.ncbi.nlm.nih.gov/22897560/>
729. Christopeit M, Lindner A, Surov A, Weber T, Vehreschild MJ, Bialek R, Schmoll HJ. Right flank pain and high fever in a neutropenic patient with acute lymphoblastic leukaemia. Mycoses. 2013 Jan;56(1):90-2. doi: 10.1111/j.1439-0507.2012.02231.x. Epub 2012 Aug 13. PMID: 22889321. Link <https://pubmed.ncbi.nlm.nih.gov/22889321/>
730. Wu BR, Li CH, Chen CH. Cavitary pulmonary opacity in a diabetic patient. Intern Med. 2012;51(15):2061-2. doi: 10.2169/internalmedicine.51.8000. Epub 2012 Aug 1. PMID: 22864139. Link <https://pubmed.ncbi.nlm.nih.gov/22864139/>
731. Tan J, Manickam R, Pisharam J, Telisinghe P, Chong VH. Mucormycosis--a possible trigger pathogen for encapsulating peritoneal sclerosis. Perit Dial Int. 2012 Jul-Aug;32(4):479-81. doi: 10.3747/pdi.2011.00241. PMID: 22859845; PMCID: PMC3524856. Link <https://pubmed.ncbi.nlm.nih.gov/22859845/>
732. García-Pajares F, Sánchez-Antolín G, Almohalla Alvárez C, Madrigal Rubiales B, Núñez-Rodríguez H, Sancho del Val L, Ruiz-Zorrilla R, Barrera A, Gómez-Nieto A, Peñas Herrero I, Vargas García A, Caro-Patón A. Cutaneous mucormycosis infection by Absidia in two consecutive liver transplant patients. Transplant Proc. 2012 Jul-Aug;44(6):1562-4. doi: 10.1016/j.transproceed.2012.05.022. PMID: 22841214. Link <https://pubmed.ncbi.nlm.nih.gov/22841214/>
733. Alsuhaibani AH, Al-Thubaiti G, Al Badr FB. Optic nerve thickening and infarction as the first evidence of orbital involvement with mucormycosis. Middle East Afr J Ophthalmol. 2012 Jul-Sep;19(3):340-2. doi: 10.4103/0974-9233.97957. PMID: 22837632; PMCID: PMC3401808. Link <https://pubmed.ncbi.nlm.nih.gov/22837632/>
734. Kumar M, Tanwar P, Radhika S, Dey P. Fine needle aspiration cytology of primary mucormycosis of the breast in a young immunocompetent pregnant woman. Cytopathology. 2013 Dec;24(6):411-2. doi: 10.1111/j.1365-2303.2012.01002.x. Epub 2012 Jul 26. PMID: 22830302. Link <https://pubmed.ncbi.nlm.nih.gov/22830302/>
735. Uchida Y, Tsukino M, Shigemori W, Hayashi E, Watanabe I, Nakayama T, Yamada E, Moro K. Diagnosis of pulmonary mucormycosis aiding the diagnosis of small cell lung cancer. J Med Microbiol. 2012 Nov;61(Pt 11):1610-1613. doi: 10.1099/jmm.0.040766-0. Epub 2012 Jul 19. PMID: 22820691. Link <https://pubmed.ncbi.nlm.nih.gov/22820691/>
736. Dang CJ, Li YJ, Zhan FH, Shang XM. The appearance of pulmonary mucormycosis on FDG PET/CT. Clin Nucl Med. 2012 Aug;37(8):801-3. doi: 10.1097/RLU.0b013e31825ae470. PMID: 22785517. Link <https://pubmed.ncbi.nlm.nih.gov/22785517/>
737. Mayayo E, Stchigel AM, Cano JF, Bernal-Escoté X, Guarro J. Fascitis necrotizante por Saksenaea vasiformis en una paciente inmunocompetente tras un accidente de tráfico [Necrotising fasciitis caused by Saksenaea vasiformis in an immunocompetent patient after a car accident]. Rev Iberoam Micol. 2013 Jan 3;30(1):57-60. Spanish. doi: 10.1016/j.riam.2012.06.002. Epub 2012 Jun 29. PMID: 22749974. Link <https://pubmed.ncbi.nlm.nih.gov/22749974/>
738. Azarpira N, Ashraf MJ, Kazemi K, Khademi B. Rhinomaxillary mucormycosis in a renal transplant recipient: case report. Exp Clin Transplant. 2012 Dec;10(6):605-8. doi: 10.6002/ect.2012.0006. Epub 2012 Jun 29. PMID: 22747311. Link <https://pubmed.ncbi.nlm.nih.gov/22747311/>
739. Lin SY, Lu PL, Tsai KB, Lin CY, Lin WR, Chen TC, Chang YT, Huang CH, Chen CY, Lai CC, Chen YH. A mucormycosis case in a cirrhotic patient successfully treated with posaconazole and review of published literature. Mycopathologia. 2012 Dec;174(5-6):499-504. doi: 10.1007/s11046-012-9561-8. Epub 2012 Jun 29. PMID: 22744722. Link <https://pubmed.ncbi.nlm.nih.gov/22744722/>
740. Lalwani S, Govindasamy M, Gupta M, Siraj F, Varma V, Mehta N, Kumaran V, Mohan N, Chopra P, Arora A, Agarwal S, Soin A, Nundy S. Gastrointestinal mucormycosis--four cases with different risk factors, involving different anatomical sites. Indian J Gastroenterol. 2012 Jun;31(3):139-43. doi: 10.1007/s12664-012-0215-z. Epub 2012 Jun 29. PMID: 22744237. Link <https://pubmed.ncbi.nlm.nih.gov/22744237/>
741. Kawasaki M, Kawakami N, Kawai K, Kanekura T. Cutaneous mucormycosis in bone marrow transplantation recipients. Eur J Dermatol. 2012 Jul-Aug;22(4):578-9. doi: 10.1684/ejd.2012.1770. PMID: 22743341. Link <https://pubmed.ncbi.nlm.nih.gov/22743341/>
742. Kimura M, Nishimura K, Enoki E, Chikugo T, Maenishi O. Chlamydospores of Rhizopus microsporus var. rhizopodiformis in tissue of pulmonary mucormycosis. Mycopathologia. 2012 Dec;174(5-6):441-50. doi: 10.1007/s11046-012-9560-9. Epub 2012 Jun 26. PMID: 22733079. Link <https://pubmed.ncbi.nlm.nih.gov/22733079/>
743. Shatriah I, Mohd-Amin N, Tuan-Jaafar TN, Khanna RK, Yunus R, Madhavan M. Rhino-orbito-cerebral mucormycosis in an immunocompetent patient: case report and review of literature. Middle East Afr J Ophthalmol. 2012 Apr-Jun;19(2):258-61. doi: 10.4103/0974-9233.95269. PMID: 22623872; PMCID: PMC3353681. Link <https://pubmed.ncbi.nlm.nih.gov/22623872/>
744. dela Cruz WP, Calvano TP, Griffith ME, White CE, Kim SH, Sutton DA, Thompson EH, Fu J, Wickes BL, Guarro J, Hospenthal DR. Invasive Apophysomyces variabilis infection in a burn patient. J Clin Microbiol. 2012 Aug;50(8):2814-7. doi: 10.1128/JCM.00671-12. Epub 2012 May 23. PMID: 22622444; PMCID: PMC3421509. Link <https://pubmed.ncbi.nlm.nih.gov/22622444/>
745. Rodríguez Pérez M, Zambrano Castaño D, Cáceres Gómez-Valadés R, de Tena Rocha I, Sánchez Sánchez J, Romero Bernal J, Pérez Gayte J. Mucormicosis sin compromiso inmunológico previo en la unidad de reanimación. Descripción de 2 casos [Mucormycosis with no previous immune compromise in the resuscitation unit]. Rev Esp Anestesiol Reanim. 2012 Nov;59(9):516-8. Spanish. doi: 10.1016/j.redar.2012.02.030. Epub 2012 May 18. PMID: 22609266. Link <https://pubmed.ncbi.nlm.nih.gov/22609266/>
746. Honeybul S, Morrison DA. Skull vault destruction after rhinocerebral mucormycosis. World Neurosurg. 2012 Nov;78(5):553.e1-4. doi: 10.1016/j.wneu.2011.12.009. Epub 2011 Dec 10. PMID: 22609027. Link <https://pubmed.ncbi.nlm.nih.gov/22609027/>
747. Charles P, Kahn JE, Ackermann F, Honderlick P, Lortholary O. Renal mucormycosis complicating extracorporeal membrane oxygenation. Med Mycol. 2013 Feb;51(2):193-5. doi: 10.3109/13693786.2012.686069. Epub 2012 May 15. PMID: 22587731. Link <https://pubmed.ncbi.nlm.nih.gov/22587731/>
748. Bhutada K, Borkar SS, Mendiratta DK, Shende VR. Successful treatment of peritonitis by C. bertholletiae in a chronic kidney failure patient on continuous ambulatory peritoneal dialysis after kidney rejection. Singapore Med J. 2012 May;53(5):e106-9. PMID: 22584986. Link <https://pubmed.ncbi.nlm.nih.gov/22584986/>
749. Zhao L, Wang CX, Zhang L, Tu XA, Wang W, Chen Y, Liu LS. Mucormycosis extending from the surgical wound to the transplanted kidney: case report and literature review. Exp Clin Transplant. 2012 Aug;10(4):403-5. doi: 10.6002/ect.2011.0107. Epub 2012 May 14. PMID: 22583076. Link <https://pubmed.ncbi.nlm.nih.gov/22583076/>
750. Mohammadi A, Mehdizadeh A, Ghasemi-Rad M, Habibpour H, Esmaeli A. Pulmonary mucormycosis in patients with diabetic ketoacidosis: a case report and review of literature. Tuberk Toraks. 2012;60(1):66-9. doi: 10.5578/tt.2464. PMID: 22554371. Link <https://pubmed.ncbi.nlm.nih.gov/22554371/>
751. Manrique K, Martín C, Sánchez-Vilar O, Aragón C, Cazorla A, Rovira A. Rhinomucormycosis and type 1 diabetes mellitus. Endocrinol Nutr. 2013 Mar;60(3):149-51. English, Spanish. doi: 10.1016/j.endonu.2012.01.025. Epub 2012 Apr 20. PMID: 22521299. Link <https://pubmed.ncbi.nlm.nih.gov/22521299/>
752. Su H, Thompson GR 3rd, Cohen SH. Hepatic mucormycosis with abscess formation. Diagn Microbiol Infect Dis. 2012 Jun;73(2):192-4. doi: 10.1016/j.diagmicrobio.2012.02.021. Epub 2012 Apr 12. PMID: 22502961. Link <https://pubmed.ncbi.nlm.nih.gov/22502961/>
753. Dussaule C, Nifle C, Therby A, Eloy O, Cordoliani Y, Pico F. Teaching neuroimages: brain MRI aspects of isolated cerebral mucormycosis. Neurology. 2012 Apr 3;78(14):e93. doi: 10.1212/WNL.0b013e31824e8ed0. PMID: 22474302. Link <https://pubmed.ncbi.nlm.nih.gov/22474302/>
754. Lmekki S, Zaki Z, El Alami MN. Mucormycose rhinocérébrale [Rhinocerebral mucormycosis]. Med Mal Infect. 2012 Apr;42(4):171-3. French. doi: 10.1016/j.medmal.2012.02.007. Epub 2012 Mar 31. PMID: 22465059. Link <https://pubmed.ncbi.nlm.nih.gov/22465059/>
755. Nawange SR, Singh SM, Naidu J, Jain S, Nagpal T, Behrani DS, Mellado E, Tudela JL. Zygomycosis caused by Rhizopus microsporus and Rhizopus oryzae in Madhya Pradesh (M.P.) Central India: a report of two cases. Mycopathologia. 2012 Aug;174(2):171-6. doi: 10.1007/s11046-012-9532-0. Epub 2012 Mar 23. PMID: 22437261. Link <https://pubmed.ncbi.nlm.nih.gov/22437261/>
756. Metzen D, Böhm H, Zimmermann M, Reuther T, Kübler AC, Müller-Richter UD. Mucormycosis of the head and neck. J Craniomaxillofac Surg. 2012 Dec;40(8):e321-7. doi: 10.1016/j.jcms.2012.01.015. Epub 2012 Mar 16. PMID: 22425500. Link <https://pubmed.ncbi.nlm.nih.gov/22425500/>
757. <https://pubmed.ncbi.nlm.nih.gov/22416104/> Link <https://pubmed.ncbi.nlm.nih.gov/22416104/>
758. Alrefai AH, Berger JR, Saadeh RS. Against all odds: surviving rhino-orbital-cerebral mucormycosis: a case report. J Neurovirol. 2012 Apr;18(2):144-7. doi: 10.1007/s13365-012-0086-1. Epub 2012 Mar 13. PMID: 22411001. Link <https://pubmed.ncbi.nlm.nih.gov/22411001/>
759. García-Rodríguez J, Quiles-Melero I, Humala-Barbier K, Monzon A, Cuenca-Estrella M. Isolation of Cunninghamella blakesleeana in an immunodepressed patient. Mycoses. 2012 Sep;55(5):463-5. doi: 10.1111/j.1439-0507.2012.02174.x. Epub 2012 Feb 24. PMID: 22360287. Link <https://pubmed.ncbi.nlm.nih.gov/22360287/>
760. Rabie NB, Althaqafi AO. Rhizopus-associated soft tissue infection in an immunocompetent air-conditioning technician after a road traffic accident: a case report and review of the literature. J Infect Public Health. 2012 Mar;5(1):109-11. doi: 10.1016/j.jiph.2011.10.002. Epub 2011 Dec 4. PMID: 22341850. Link <https://pubmed.ncbi.nlm.nih.gov/22341850/>
761. Lineberry KD, Boettcher AK, Blount AL, Burgess SD. Cutaneous mucormycosis of the upper extremity in an immunocompetent host: case report. J Hand Surg Am. 2012 Apr;37(4):787-91. doi: 10.1016/j.jhsa.2011.11.010. Epub 2012 Feb 2. PMID: 22305738. Link <https://pubmed.ncbi.nlm.nih.gov/22305738/>
762. Weddle G, Gandy K, Bratcher D, Pahud B, Jackson MA. Apophysomyces trapeziformis infection associated with a tornado-related injury. Pediatr Infect Dis J. 2012 Jun;31(6):640-2. doi: 10.1097/INF.0b013e31824bcc36. PMID: 22301481. Link <https://pubmed.ncbi.nlm.nih.gov/22301481/>
763. Xess I, Mohapatra S, Shivaprakash MR, Chakrabarti A, Benny GL, O'Donnell K, Padhye AA. Evidence implicating Thamnostylum lucknowense as an etiological agent of rhino-orbital mucormycosis. J Clin Microbiol. 2012 Apr;50(4):1491-4. doi: 10.1128/JCM.06611-11. Epub 2012 Feb 1. PMID: 22301030; PMCID: PMC3318503. Link <https://pubmed.ncbi.nlm.nih.gov/22301030/>
764. Jain S, Kumar S, Kaushal A. Rhinocerebral mucormycosis with isolated sixth nerve palsy in an immunocompetent patient. Med J Malaysia. 2011 Oct;66(4):376-8. PMID: 22299566. Link <https://pubmed.ncbi.nlm.nih.gov/22299566/>
765. Kaushik R, Chander J, Gupta S, Sharma R, Punia RS. Fatal primary cutaneous zygomycosis caused by Saksenaea vasiformis: case report and review of literature. Surg Infect (Larchmt). 2012 Apr;13(2):125-9. doi: 10.1089/sur.2010.078. Epub 2012 Jan 26. PMID: 22280152. Link <https://pubmed.ncbi.nlm.nih.gov/22280152/>
766. Kumar S, Pushkarna A, Sharma V, Ganesamoni R, Nada R. Fournier's gangrene with testicular infarction caused by mucormycosis. Indian J Pathol Microbiol. 2011 Oct-Dec;54(4):847-8. doi: 10.4103/0377-4929.91520. PMID: 22234133. Link <https://pubmed.ncbi.nlm.nih.gov/22234133/>
767. Jiménez Caballero PE, Falcón García AM, Portilla Cuenca JC, Casado Naranjo I. Multiple subcortical strokes caused by mucormycosis in a patient with lymphoma. Arq Neuropsiquiatr. 2012 Jan;70(1):69-70. doi: 10.1590/s0004-282x2012000100014. PMID: 22218477. Link <https://pubmed.ncbi.nlm.nih.gov/22218477/>
768. Kontoyiannis DP. Invasive mycoses: strategies for effective management. Am J Med. 2012 Jan;125(1 Suppl):S25-38. doi: 10.1016/j.amjmed.2011.10.009. PMID: 22196206. Link <https://pubmed.ncbi.nlm.nih.gov/22196206/>
769. Mahmud A, Lee R, Munfus-McCray D, Kwiatkowski N, Subramanian A, Neofytos D, Carroll K, Zhang SX. Actinomucor elegans as an emerging cause of Mucormycosis. J Clin Microbiol. 2012 Mar;50(3):1092-5. doi: 10.1128/JCM.05338-11. Epub 2012 Jan 11. PMID: 22205785; PMCID: PMC3295095. Link <https://pubmed.ncbi.nlm.nih.gov/22205785/>
770. Dua R, Poate T, Abe F, Diaz-Cano S. Lingual necrosis secondary to mucormycosis. Br J Oral Maxillofac Surg. 2012 Sep;50(6):e96-8. doi: 10.1016/j.bjoms.2011.11.008. Epub 2011 Dec 5. PMID: 22153179. Link <https://pubmed.ncbi.nlm.nih.gov/22153179/>
771. Rudler M, Barret M, Poynard T, Thabut D. Gastric mucormycosis: a rare cause of gastrointestinal bleeding in cirrhosis. Clin Res Hepatol Gastroenterol. 2012 Apr;36(2):e32-3. doi: 10.1016/j.clinre.2011.10.009. Epub 2011 Dec 3. PMID: 22138061. Link <https://pubmed.ncbi.nlm.nih.gov/22138061/>
772. Anand J, Ghazala K, Chong VH. Massive lower gastrointestinal bleeding secondary to colonic mucormycosis. Med J Malaysia. 2011 Aug;66(3):266-7. PMID: 22111457. Link <https://pubmed.ncbi.nlm.nih.gov/22111457/>
773. Khandelwal A, Gupta P, Gupta A, Virmani V. Renal mucormycosis in aplastic anemia: a novel presentation. Int Urol Nephrol. 2013 Feb;45(1):285-8. doi: 10.1007/s11255-011-0078-8. Epub 2011 Nov 23. PMID: 22109676 Link <https://pubmed.ncbi.nlm.nih.gov/22109676/>
774. Hadaschik E, Koschny R, Willinger B, Hallscheidt P, Enk A, Hartschuh W. Pulmonary, rhino-orbital and cutaneous mucormycosis caused by Rhizomucor pusillus in an immunocompromised patient. Clin Exp Dermatol. 2012 Jun;37(4):355-7. doi: 10.1111/j.1365-2230.2011.04235.x. Epub 2011 Nov 21. PMID: 22103628. Link <https://pubmed.ncbi.nlm.nih.gov/22103628/>
775. Kimura M, Araoka H, Uchida N, Ohno H, Miyazaki Y, Fujii T, Nishida A, Izutsu K, Wake A, Taniguchi S, Yoneyama A. Cunninghamella bertholletiae pneumonia showing a reversed halo sign on chest computed tomography scan following cord blood transplantation. Med Mycol. 2012 May;50(4):412-6. doi: 10.3109/13693786.2011.631153. Epub 2011 Nov 22. PMID: 22103345. Link <https://pubmed.ncbi.nlm.nih.gov/22103345/>
776. González Ballester D, González-García R, Moreno García C, Ruiz-Laza L, Monje Gil F. Mucormycosis of the head and neck: report of five cases with different presentations. J Craniomaxillofac Surg. 2012 Oct;40(7):584-91. doi: 10.1016/j.jcms.2011.10.015. Epub 2011 Nov 13. PMID: 22082732. Link <https://pubmed.ncbi.nlm.nih.gov/22082732/>
777. Kumar AR, Hunt P, Ritter EM, Howard R. Successful knee extensor mechanism reconstruction in a warfare-related open lower extremity injury complicated by mucormycosis infection: a case report. J Orthop Trauma. 2012 Feb;26(2):e7-10. doi: 10.1097/BOT.0b013e318214e2c6. PMID: 22048185. Link <https://pubmed.ncbi.nlm.nih.gov/22048185/>
778. Li F, Yang HM, Chai JK, Wang HW. Burn wound mucormycosis: a case report. J Burn Care Res. 2012 Jan-Feb;33(1):e24-5. doi: 10.1097/BCR.0b013e3182335a39. PMID: 22042238. Link <https://pubmed.ncbi.nlm.nih.gov/22042238/>
779. Tan S, Aronowitz P. A common fungus, an unusual (and deadly) infection. Am J Med. 2011 Nov;124(11):1023-4. doi: 10.1016/j.amjmed.2011.08.005. PMID: 22017779. Link <https://pubmed.ncbi.nlm.nih.gov/22017779/>
780. Radowsky JS, Strawn AA, Sherwood J, Braden A, Liston W. Invasive mucormycosis and aspergillosis in a healthy 22-year-old battle casualty: case report. Surg Infect (Larchmt). 2011 Oct;12(5):397-400. doi: 10.1089/sur.2010.065. Epub 2011 Oct 17. PMID: 22004440. Link <https://pubmed.ncbi.nlm.nih.gov/22004440/>
781. Domínguez Mdel C, Sánchez J, Carmona E, Vergara-López S. Paciente anciana con lesiones cutáneas de rápida progresión [Elderly patient with rapidly progressive skin lesions]. Enferm Infecc Microbiol Clin. 2012 Jan;30(1):43-5. Spanish. doi: 10.1016/j.eimc.2011.07.015. Epub 2011 Oct 11. PMID: 21996424. Link <https://pubmed.ncbi.nlm.nih.gov/21996424/>
782. Itoh M, Oki M, Yanagi H, Oka A, Tajiri S, Fukuda R, Ozawa H, Takagi A. Disseminated mucormycosis infection after the first course of dose-modified R-EPOCH for advanced-stage lymphoma. J Infect Chemother. 2012 Jun;18(3):395-8. doi: 10.1007/s10156-011-0318-y. Epub 2011 Oct 12. PMID: 21989517. Link <https://pubmed.ncbi.nlm.nih.gov/21989517/>
783. Thomas S, Singh VD, Vaithilingam Y, Thayil SC, Kothari R. Rhinocerebral mucormycosis--a case report. Oral Maxillofac Surg. 2012 Jun;16(2):233-6. doi: 10.1007/s10006-011-0292-7. Epub 2011 Sep 28. PMID: 21952910. Link <https://pubmed.ncbi.nlm.nih.gov/21952910/>
784. Ribeiro LC, Wanke B, da Silva M, Dias LB, Mello R, Canavarros FA, Leite DP Jr, Hahn RC. Mucormycosis in Mato Grosso, Brazil: a case reports, caused by Rhizopus microsporus var. oligosporus and Rhizopus microsporus var. rhizopodiformis. Mycopathologia. 2012 Mar;173(2-3):187-92. doi: 10.1007/s11046-011-9472-0. Epub 2011 Sep 28. PMID: 21952835. Link <https://pubmed.ncbi.nlm.nih.gov/21952835/>
785. Chhaya V, Gupta S, Arnaout A. Mucormycosis causing giant gastric ulcers. Endoscopy. 2011;43 Suppl 2 UCTN:E289-90. doi: 10.1055/s-0030-1256425. Epub 2011 Sep 13. PMID: 21915831. Link <https://pubmed.ncbi.nlm.nih.gov/21915831/>
786. Mohta A, Neogi S, Das S. Gastrointestinal mucormycosis in an infant. Indian J Pathol Microbiol. 2011 Jul-Sep;54(3):664-5. doi: 10.4103/0377-4929.85149. PMID: 21934265. Link <https://pubmed.ncbi.nlm.nih.gov/21934265/>
787. von Scheven R, Lebiedz P, Spieker T, Uekoetter A, Berdel WE, Kessler T. Fulminant invasive pulmonary mucormycosis with Rhizopus oryzae in a patient with severe aplastic anaemia and common variable immunodeficiency. Mycoses. 2012 Mar;55(2):e32-5. doi: 10.1111/j.1439-0507.2011.02119.x. Epub 2011 Sep 13. PMID: 21914004. Link <https://pubmed.ncbi.nlm.nih.gov/21914004/>
788. Kiratli H, Erkan K. Mucormycose rhino-orbitaire: une pathologie à forte létalité [Naso-orbital mucormycosis: a rapidly lethal condition]. J Fr Ophtalmol. 2012 Jan;35(1):76-7. French. doi: 10.1016/j.jfo.2011.07.002. Epub 2011 Sep 8. PMID: 21906839. Link <https://pubmed.ncbi.nlm.nih.gov/21906839/>
789. Doni BR, Peerapur BV, Thotappa LH, Hippargi SB. Sequence of oral manifestations in rhino-maxillary mucormycosis. Indian J Dent Res. 2011 Mar-Apr;22(2):331-5. doi: 10.4103/0970-9290.84313. PMID: 21891908. Link <https://pubmed.ncbi.nlm.nih.gov/21891908/>
790. Chaganti J, Marriott D, Steel T, Donovan J, Biggs N. Perineural trigeminal nerve abscess secondary to mucor sinusitis: serial diffusion-weighted MRI and literature review. Clin Radiol. 2011 Nov;66(11):1106-9. doi: 10.1016/j.crad.2011.05.016. Epub 2011 Aug 31. PMID: 21885047. Link <https://pubmed.ncbi.nlm.nih.gov/21885047/>
791. Shakoor S, Jabeen K, Idrees R, Jamil B, Irfan S, Zafar A. Necrotising fasciitis due to Absidia corymbifera in wounds dressed with non sterile bandages. Int Wound J. 2011 Dec;8(6):651-5. doi: 10.1111/j.1742-481X.2011.00839.x. Epub 2011 Aug 25. PMID: 21883933; PMCID: PMC7950822. Link <https://pubmed.ncbi.nlm.nih.gov/21883933/>
792. Tapia E O, Chahín A C, Concha F C. Mucormicosis cutánea primaria: a propósito de dos casos. Revisión de la literatura [Primary cutaneous mucormycosis: two case reports and review of the literature]. Rev Chilena Infectol. 2011 Jun;28(3):269-73. Spanish. Epub 2011 Jul 14. PMID: 21879155 Link <https://pubmed.ncbi.nlm.nih.gov/21879155/>
793. Singh V, Sharma B, Sen R, Agrawal S, Bhagol A, Bali R. Rhinocerebral mucormycosis: a diagnostic challenge and therapeutic dilemma in immunocompetent host. J Oral Maxillofac Surg. 2012 Jun;70(6):1369-75. doi: 10.1016/j.joms.2011.06.209. Epub 2011 Aug 24. PMID: 21864966. Link <https://pubmed.ncbi.nlm.nih.gov/21864966/>
794. Viterbo S, Fasolis M, Garzino-Demo P, Griffa A, Boffano P, Iaquinta C, Tanteri G, Modica R. Management and outcomes of three cases of rhinocerebral mucormycosis. Oral Surg Oral Med Oral Pathol Oral Radiol Endod. 2011 Dec;112(6):e69-74. doi: 10.1016/j.tripleo.2011.04.048. Epub 2011 Sep 8. PMID: 21862361. Link <https://pubmed.ncbi.nlm.nih.gov/21862361/>
795. Geisen M, Fodor P, Eich G, Zollinger A, Dzemali O, Blumenthal S. Disseminated cutaneous mucormycosis in a patient on high-dose steroid therapy for severe ARDS. Intensive Care Med. 2011 Nov;37(11):1895-6. doi: 10.1007/s00134-011-2347-5. Epub 2011 Aug 20. PMID: 21858519. Link <https://pubmed.ncbi.nlm.nih.gov/21858519/>
796. Ilyas S, Al-Abbadi MA, Raval B, Shams WE. Mucor causing nonhealing skin ulcer diagnosed by scrape cytology: description of unusual presentation. Diagn Cytopathol. 2011 Sep;39(9):714-5. doi: 10.1002/dc.21514. Epub 2010 Nov 2. PMID: 21837662. Link <https://pubmed.ncbi.nlm.nih.gov/21837662/>
797. De Yao JT, Al-Ameri A, Garcia-Manero G, Quintás-Cardama A. Infrequent presentations of mucormycosis in patients with myelodysplastic syndrome and acute leukemia: case series and review of literature. Clin Lymphoma Myeloma Leuk. 2011 Oct;11(5):446-51. doi: 10.1016/j.clml.2011.05.041. Epub 2011 Aug 5. PMID: 21820986. Link <https://pubmed.ncbi.nlm.nih.gov/21820986/>
798. Aras MH, Kara MI, Erkiliç S, Ay S. Mandibular mucormycosis in immunocompromised patients: report of 2 cases and review of the literature. J Oral Maxillofac Surg. 2012 Jun;70(6):1362-8. doi: 10.1016/j.joms.2011.05.012. Epub 2011 Aug 6. PMID: 21820787. Link <https://pubmed.ncbi.nlm.nih.gov/21820787/>
799. Chiu HY, Chang CY, Hsueh PR, Tsai TF, Liu IL, Wang LF. Multiple discrete, punched-out ulcers in a patient with pemphigus vulgaris. Clin Infect Dis. 2011 Aug;53(4):377-8, 396-8. doi: 10.1093/cid/cir351. PMID: 21810755. Link <https://pubmed.ncbi.nlm.nih.gov/21810755/>
800. Aabideen K, Lashkari HP, Holmes K, Taj M. Successful treatment of gastrointestinal mucormycosis in an adolescent with acute lymphoblastic leukaemia (ALL). Pediatr Blood Cancer. 2012 Feb;58(2):312-3. doi: 10.1002/pbc.23255. Epub 2011 Jul 25. PMID: 21793182. Link <https://pubmed.ncbi.nlm.nih.gov/21793182/>
801. Lacarrière E, Lacaze L, Schwarz L, Huet E, Lemoine F, Scotté M. First case of gastrointestinal mucormycosis in an immunocompromised patient with gallbladder and duodenum involvement. Infection. 2011 Dec;39(6):595-8. doi: 10.1007/s15010-011-0165-x. Epub 2011 Jul 23. PMID: 21786018. Link <https://pubmed.ncbi.nlm.nih.gov/21786018/>
802. Gumral R, Yildizoglu U, Saracli MA, Kaptan K, Tosun F, Yildiran ST. A case of rhinoorbital mucormycosis in a leukemic patient with a literature review from Turkey. Mycopathologia. 2011 Nov;172(5):397-405. doi: 10.1007/s11046-011-9449-z. Epub 2011 Jul 15. PMID: 21761152. Link <https://pubmed.ncbi.nlm.nih.gov/21761152/>
803. Ono A, Okada F, Ando Y, Maeda T, Saburi Y, Kondo Y, Mori H. Multiple pulmonary arteriolar emboli in a patient with disseminated mucormycosis and myelodysplastic syndrome. Clin Radiol. 2011 Oct;66(10):998-1000. doi: 10.1016/j.crad.2011.06.003. Epub 2011 Jul 13. PMID: 21742319. Link <https://pubmed.ncbi.nlm.nih.gov/21742319/>
804. Busca A, Limerutti G, Locatelli F, Barbui A, De Rosa FG, Falda M. The reversed halo sign as the initial radiographic sign of pulmonary zygomycosis. Infection. 2012 Feb;40(1):77-80. doi: 10.1007/s15010-011-0156-y. Epub 2011 Jul 7. PMID: 21735109. Link <https://pubmed.ncbi.nlm.nih.gov/21735109/>
805. Prasad K, Lalitha RM, Reddy EK, Ranganath K, Srinivas DR, Singh J. Role of early diagnosis and multimodal treatment in rhinocerebral mucormycosis: experience of 4 cases. J Oral Maxillofac Surg. 2012 Feb;70(2):354-62. doi: 10.1016/j.joms.2011.02.017. Epub 2011 Jun 16. PMID: 21680075. Link <https://pubmed.ncbi.nlm.nih.gov/21680075/>
806. Chaudhry A, Hirano SA, Hayes TJ, Torosky C. Fatal rhino-orbito-cerebral mucormycosis in a patient with liver disease. J Am Acad Dermatol. 2011 Jul;65(1):241-3. doi: 10.1016/j.jaad.2010.01.002. PMID: 21679839. Link <https://pubmed.ncbi.nlm.nih.gov/21679839/>
807. Grigoriadis G, Chang CC, Walker P, Patil S, Avery S, Morrissey O, Spencer A. Failure of haematopoietic recovery overcome by SCT despite invasive mucormycosis infection. Bone Marrow Transplant. 2012 Apr;47(4):591-2. doi: 10.1038/bmt.2011.116. Epub 2011 Jun 13. PMID: 21666738. Link <https://pubmed.ncbi.nlm.nih.gov/21666738/>
808. Lara-Aguayo P, De La Fuente-Martos C, Morán-Fernández E, Soriano-Rodríguez F, Rojas-Amezcua M, Aguilar-Alonso E. Mucormicosis fatal no sospechada en UCI [Fatal mucormycosis not suspected in the ICU]. Med Intensiva. 2012 Mar;36(2):155-7. Spanish. doi: 10.1016/j.medin.2011.04.010. Epub 2011 Jun 8. PMID: 21641682. Link <https://pubmed.ncbi.nlm.nih.gov/21641682/>
809. Saraiya HA. Successful management of cutaneous mucormycosis by delaying debridement. Ann Plast Surg. 2012 Sep;69(3):301-6. doi: 10.1097/SAP.0b013e31821bd49f. PMID: 21629068. Link <https://pubmed.ncbi.nlm.nih.gov/21629068/>
810. Däbritz J, Attarbaschi A, Tintelnot K, Kollmar N, Kremens B, von Loewenich FD, Schrod L, Schuster F, Wintergerst U, Weig M, Lehrnbecher T, Groll AH. Mucormycosis in paediatric patients: demographics, risk factors and outcome of 12 contemporary cases. Mycoses. 2011 Nov;54(6):e785-8. doi: 10.1111/j.1439-0507.2011.02025.x. Epub 2011 May 30. PMID: 21623951. Link <https://pubmed.ncbi.nlm.nih.gov/21623951/>
811. Chawla B, Sharma S, Kashyap S, Kabra SK, Pushker N, Bajaj MS. Primary orbital mycosis in immunocompetent infants. J AAPOS. 2011 Apr;15(2):211-3. doi: 10.1016/j.jaapos.2010.12.016. PMID: 21596302. Link <https://pubmed.ncbi.nlm.nih.gov/21596302/>
812. Ogawa T, Takezawa K, Tojima I, Shibayama M, Kouzaki H, Ishida M, Okabe H, Shimizu T. Successful treatment of rhino-orbital mucormycosis by a new combination therapy with liposomal amphotericin B and micafungin. Auris Nasus Larynx. 2012 Apr;39(2):224-8. doi: 10.1016/j.anl.2011.03.006. Epub 2011 May 17. PMID: 21592699. Link <https://pubmed.ncbi.nlm.nih.gov/21592699/>
813. Sugui JA, Christensen JA, Bennett JE, Zelazny AM, Kwon-Chung KJ. Hematogenously disseminated skin disease caused by Mucor velutinosus in a patient with acute myeloid leukemia. J Clin Microbiol. 2011 Jul;49(7):2728-32. doi: 10.1128/JCM.00387-11. Epub 2011 May 4. PMID: 21543575; PMCID: PMC3147817. Link <https://pubmed.ncbi.nlm.nih.gov/21543575/>
814. Jain SK, Kaza RC, Tanwar R. Mucormycosis of the anterior chest wall presenting as a soft tissue tumour. J Wound Care. 2011 Apr;20(4):176-8. doi: 10.12968/jowc.2011.20.4.176. PMID: 21537305. Link <https://pubmed.ncbi.nlm.nih.gov/21537305/>
815. Lau CI, Wang HC, Yeh HL, Li CH. Isolated orbito-cerebral mucormycosis. Neurologist. 2011 May;17(3):151-3. doi: 10.1097/NRL.0b013e3182173395. PMID: 21532384. Link <https://pubmed.ncbi.nlm.nih.gov/21532384/>
816. Peterson EA, Gerrie AS, Power MM, Poulin MP, Dalal BI, Forrest DL. Disseminated mucormycosis presenting as transplant-associated thrombotic microangiopathy. Leuk Res. 2011 Jul;35(7):e138-40. doi: 10.1016/j.leukres.2011.03.003. Epub 2011 Apr 22. PMID: 21514669. Link <https://pubmed.ncbi.nlm.nih.gov/21514669/>
817. Kim HJ, Rha SE, Kang WK. A patient with neutropenic fever and abdominal pain showing absent bowel wall on CT. Br J Radiol. 2011 May;84(1001):478-80. doi: 10.1259/bjr/13586512. PMID: 21511752; PMCID: PMC3473660. Link <https://pubmed.ncbi.nlm.nih.gov/21511752/>
818. Hemashettar BM, Patil RN, O'Donnell K, Chaturvedi V, Ren P, Padhye AA. Chronic rhinofacial mucormycosis caused by Mucor irregularis (Rhizomucor variabilis) in India. J Clin Microbiol. 2011 Jun;49(6):2372-5. doi: 10.1128/JCM.02326-10. Epub 2011 Apr 20. PMID: 21508154; PMCID: PMC3122748. Link <https://pubmed.ncbi.nlm.nih.gov/21508154/>
819. Chambers CJ, Reyes Merin M, Fung MA, Huntley A, Sharon VR. Primary cutaneous mucormycosis at sites of insulin injection. J Am Acad Dermatol. 2011 May;64(5):e79-81. doi: 10.1016/j.jaad.2010.07.012. PMID: 21496690. Link <https://pubmed.ncbi.nlm.nih.gov/21496690/>
820. Gupta R, Parelkar SV, Oak S, Sanghvi B, Prakash A. Neonatal lingual and gastrointestinal mucormycosis in a case of low anorectal malformation-a rare presentation. J Pediatr Surg. 2011 Apr;46(4):745-748. doi: 10.1016/j.jpedsurg.2010.12.024. PMID: 21496547. Link <https://pubmed.ncbi.nlm.nih.gov/21496547/>
821. Shivananda P, Mahabala C, Kausalya S, Suchitra S, Anand KU. Cutaneous mucormycosis with necrotising fasciitis in a young immunocompetent individual. Trop Doct. 2011 Jul;41(3):183-4. doi: 10.1258/td.2011.100420. Epub 2011 Apr 14. PMID: 21493646. Link <https://pubmed.ncbi.nlm.nih.gov/21493646/>
822. Lin CT, Lee JC, Chan DC, Yu JC, Hsieh CB. Successful treatment of mucormycosis infection after liver transplantation: report of a case and review of the literature. Z Gastroenterol. 2011 Apr;49(4):449-51. doi: 10.1055/s-0029-1245694. Epub 2011 Apr 7. PMID: 21476181. Link <https://pubmed.ncbi.nlm.nih.gov/21476181/>
823. Schell WA, O'Donnell K, Alspaugh JA. Heterothallic mating in Mucor irregularis and first isolate of the species outside of Asia. Med Mycol. 2011 Oct;49(7):714-23. doi: 10.3109/13693786.2011.568975. Epub 2011 Apr 1. Erratum in: Med Mycol. 2011 Oct;49(7):723. PMID: 21453223. Link <https://pubmed.ncbi.nlm.nih.gov/21453223/>
824. Durand CM, Alonso CD, Subhawong AP, Kwiatkowski NP, Showel M, Carroll KC, Marr KA. Rapidly progressive cutaneous Rhizopus microsporus infection presenting as Fournier's gangrene in a patient with acute myelogenous leukemia. Transpl Infect Dis. 2011 Aug;13(4):392-6. doi: 10.1111/j.1399-3062.2011.00601.x. Epub 2011 Mar 28. PMID: 21443549; PMCID: PMC3593591. Link <https://pubmed.ncbi.nlm.nih.gov/21443549/>
825. Ashkenazi-Hoffnung L, Bilavsky E, Avitzur Y, Amir J. Successful treatment of cutaneous zygomycosis with intravenous amphotericin B followed by oral posaconazole in a multivisceral transplant recipient. Transplantation. 2010 Nov 27;90(10):1133-5. doi: 10.1097/TP.0b013e3181f86916. PMID: 21427635. Link <https://pubmed.ncbi.nlm.nih.gov/21427635/>
826. Singh V, Sharma B, Sen R, Agrawal S, Dhingra R. Abnormal growth of the maxillary sinus and orbit. J Oral Maxillofac Surg. 2011 Aug;69(8):2167-72. doi: 10.1016/j.joms.2010.10.036. Epub 2011 Mar 2. PMID: 21367500. Link <https://pubmed.ncbi.nlm.nih.gov/21367500/>
827. Rawlinson NJ, Fung B, Gross TG, Termuhlen AM, Skeens M, Garee A, Soni S, Pietryga D, Bajwa RP. Disseminated Rhizomucor pusillus causing early multiorgan failure during hematopoietic stem cell transplantation for severe aplastic anemia. J Pediatr Hematol Oncol. 2011 Apr;33(3):235-7. doi: 10.1097/MPH.0b013e3182050a4f. PMID: 21358340. Link <https://pubmed.ncbi.nlm.nih.gov/21358340/>
828. Lin WY, Chang TK, Chou CM, Shen CY, Wang JD. Intraabdominal mass as presentation of colonic mucormycosis in a child with acute lymphoblastic leukemia. J Pediatr Hematol Oncol. 2011 Mar;33(2):e72-4. doi: 10.1097/MPH.0b013e3181f46b97. PMID: 21317813. Link <https://pubmed.ncbi.nlm.nih.gov/21317813/>
829. Colón-Santos E, González-Ramos M, Bertrán-Pasarell J, Rodríguez-Vega G, Almira-Suarez M, Vélez-Rosario R. Disseminated nocardiosis masking an atypical zygomycosis presentation in a kidney transplant recipient. Transpl Infect Dis. 2011 Aug;13(4):380-4. doi: 10.1111/j.1399-3062.2011.00606.x. Epub 2011 Feb 10. PMID: 21309966. Link <https://pubmed.ncbi.nlm.nih.gov/21309966/>
830. Van Sickels N, Hoffman J, Stuke L, Kempe K. Survival of a patient with trauma-induced mucormycosis using an aggressive surgical and medical approach. J Trauma. 2011 Feb;70(2):507-9. doi: 10.1097/TA.0b013e31820784ff. PMID: 21307754. Link <https://pubmed.ncbi.nlm.nih.gov/21307754/>
831. Cherfan CG, Mansour AM, Younis MH, Korn BS. Unilateral proptosis in a 60-year-old man. Surv Ophthalmol. 2011 Jul-Aug;56(4):374-8. doi: 10.1016/j.survophthal.2010.08.009. Epub 2011 Jan 14. PMID: 21236458. Link <https://pubmed.ncbi.nlm.nih.gov/21236458/>
832. Infante-Cossio P, Gacto-Sanchez P, Hens-Aumente E, Sicilia-Castro D. Postoperative donor-site mucormycosis after fibula flap harvest. Br J Oral Maxillofac Surg. 2011 Mar;49(2):158-9. doi: 10.1016/j.bjoms.2010.12.004. Epub 2011 Jan 12. PMID: 21232834. Link <https://pubmed.ncbi.nlm.nih.gov/21232834/>
833. Gupta A, Lal C, Dogra PM, Mahajan S, Agarwal SK. Insulin site wound in a renal allograft recipient. Saudi J Kidney Dis Transpl. 2011 Jan;22(1):134-5. PMID: 21196630. Link <https://pubmed.ncbi.nlm.nih.gov/21196630/>
834. Guarro J, Chander J, Alvarez E, Stchigel AM, Robin K, Dalal U, Rani H, Punia RS, Cano JF. Apophysomyces variabilis infections in humans. Emerg Infect Dis. 2011 Jan;17(1):134-5. doi: 10.3201/eid1701.101139. PMID: 21192877; PMCID: PMC3204648. Link <https://pubmed.ncbi.nlm.nih.gov/21192877/>
835. Dalgic B, Bukulmez A, Sari S. Pyogenic liver abscess and peritonitis due to Rhizopus oryzae in a child with Papillon-Lefevre syndrome. Eur J Pediatr. 2011 Jun;170(6):803-5. doi: 10.1007/s00431-010-1368-3. Epub 2010 Dec 17. PMID: 21165748. Link <https://pubmed.ncbi.nlm.nih.gov/21165748/>
836. Mukasa Y, Ichiyasu H, Akaike K, Okamoto S, Komohara Y, Kohrogi H. [Autopsy case of pulmonary zygomycosis and pneumocystis pneumonia in a patient with interstitial pneumonia treated by corticosteroid therapy]. Nihon Kokyuki Gakkai Zasshi. 2010 Nov;48(11):847-54. Japanese. PMID: 21141065. Link <https://pubmed.ncbi.nlm.nih.gov/21141065/>
837. Blazquez D, Ruiz-Contreras J, Fernández-Cooke E, González-Granado I, Delgado MD, Menendez MT, Rodriguez-Gil Y, Ballen A, Del Palacio A. Lichtheimia corymbifera subcutaneous infection successfully treated with amphotericin B, early debridement, and vacuum-assisted closure. J Pediatr Surg. 2010 Dec;45(12):e13-5. doi: 10.1016/j.jpedsurg.2010.08.011. PMID: 21129524. Link <https://pubmed.ncbi.nlm.nih.gov/21129524/>
838. Pinto ME, Manrique HA, Guevara X, Acosta M, Villena JE, Solís J. Hyperglycemic hyperosmolar state and rhino-orbital mucormycosis. Diabetes Res Clin Pract. 2011 Feb;91(2):e37-9. doi: 10.1016/j.diabres.2010.09.038. Epub 2010 Nov 23. PMID: 21106269. Link <https://pubmed.ncbi.nlm.nih.gov/21106269/>
839. Pandey A, Bansal V, Asthana AK, Trivedi V, Madan M, Das A. Maxillary osteomyelitis by mucormycosis: report of four cases. Int J Infect Dis. 2011 Jan;15(1):e66-9. doi: 10.1016/j.ijid.2010.09.003. Epub 2010 Nov 18. PMID: 21093341. Link <https://pubmed.ncbi.nlm.nih.gov/21093341/>
840. Al Akhrass F, Debiane L, Abdallah L, Best L, Mulanovich V, Rolston K, Kontoyiannis DP. Palatal mucormycosis in patients with hematologic malignancy and stem cell transplantation. Med Mycol. 2011 May;49(4):400-5. doi: 10.3109/13693786.2010.533391. Epub 2010 Nov 15. PMID: 21077735. Link <https://pubmed.ncbi.nlm.nih.gov/21077735/>
841. Tomita H, Muroi E, Takenaka M, Nishimoto K, Kakeya H, Ohno H, Miyazaki Y, Utani A. Rhizomucor variabilis infection in human cutaneous mucormycosis. Clin Exp Dermatol. 2011 Apr;36(3):312-4. doi: 10.1111/j.1365-2230.2010.03956.x. Epub 2010 Nov 10. PMID: 21070336. Link <https://pubmed.ncbi.nlm.nih.gov/21070336/>
842. Ataseven H, Yüksel I, Gültuna S, Köklü S, Uysal S, Basar O, Sasmaz N. Fatal rhinocerebral mucormycosis under the shade of hepatic encephalopathy. Ann Hepatol. 2010 Oct-Dec;9(4):462-4. PMID: 21057167. Link <https://pubmed.ncbi.nlm.nih.gov/21057167/>
843. Mohapatra S, Xess I, Shwetha JV, Choudhary A. Soil extract media for sporulation of Apophysomyces elegans. Indian J Pathol Microbiol. 2010 Oct-Dec;53(4):897-8. doi: 10.4103/0377-4929.72052. PMID: 21045478. Link <https://pubmed.ncbi.nlm.nih.gov/21045478/>
844. Li WF, He C, Liu XF, Wang SY, Qu JL, Lin ZF. A diagnosis neglected for 6 years: report of a misdiagnosed case of pulmonary mucormycosis and review of the literature. Chin Med J (Engl). 2010 Sep;123(17):2480-2. PMID: 21034571. Link <https://pubmed.ncbi.nlm.nih.gov/21034571/>
845. Calcagno A, Baietto L, De Rosa FG, Tettoni MC, Libanore V, Bertucci R, D'Avolio A, Di Perri G. Posaconazole cerebrospinal concentrations in an HIV-infected patient with brain mucormycosis. J Antimicrob Chemother. 2011 Jan;66(1):224-5. doi: 10.1093/jac/dkq385. Epub 2010 Oct 20. PMID: 20961910. Link <https://pubmed.ncbi.nlm.nih.gov/20961910/>
846. Luo LC, Cheng DY, Zhu H, Shu X, Chen WB. Inflammatory pseudotumoural endotracheal mucormycosis with cartilage damage. Eur Respir Rev. 2009 Sep;18(113):186-9. doi: 10.1183/09059180.00000709. PMID: 20956142. Link <https://pubmed.ncbi.nlm.nih.gov/20956142/>
847. Weng TF, Ho MW, Lin HC, Lu MY, Peng CT, Wu KH. Successful treatment of disseminated mixed invasive fungal infection after hematopoietic stem cell transplantation for severe aplastic anemia. Pediatr Transplant. 2012 Mar;16(2):E35-8. doi: 10.1111/j.1399-3046.2010.01406.x. Epub 2010 Oct 14. PMID: 20946236. Link <https://pubmed.ncbi.nlm.nih.gov/20946236/>
848. Ingham A, Gilbert JD, Byard RW. Disseminated fungal infection with renal infarction simulating homicide. Am J Forensic Med Pathol. 2010 Dec;31(4):390-2. doi: 10.1097/PAF.0b013e3181f69cb6. PMID: 20938325. Link <https://pubmed.ncbi.nlm.nih.gov/20938325/>
849. Alexander BD, Schell WA, Siston AM, Rao CY, Bower WA, Balajee SA, Howell DN, Moore ZS, Noble-Wang J, Rhyne JA, Fleischauer AT, Maillard JM, Kuehnert M, Vikraman D, Collins BH, Marroquin CE, Park BJ. Fatal Apophysomyces elegans infection transmitted by deceased donor renal allografts. Am J Transplant. 2010 Sep;10(9):2161-7. doi: 10.1111/j.1600-6143.2010.03216.x. Epub 2010 Aug 25. PMID: 20883549. Link <https://pubmed.ncbi.nlm.nih.gov/20883549/>
850. Cano P, Horseman MA, Surani S. Rhinocerebral mucormycosis complicated by bacterial brain abscess. Am J Med Sci. 2010 Dec;340(6):507-10. doi: 10.1097/MAJ.0b013e3181f0aad1. PMID: 20861715. Link <https://pubmed.ncbi.nlm.nih.gov/20861715/>
851. Wüppenhorst N, Lee MK, Rappold E, Kayser G, Beckervordersandforth J, de With K, Serr A. Rhino-orbitocerebral zygomycosis caused by Conidiobolus incongruus in an immunocompromised patient in Germany. J Clin Microbiol. 2010 Nov;48(11):4322-5. doi: 10.1128/JCM.01188-10. Epub 2010 Sep 22. PMID: 20861341; PMCID: PMC3020825. Link <https://pubmed.ncbi.nlm.nih.gov/20861341/>
852. Struck MF, Illert T, Stiller D, Steen M. Basilar artery occlusion after multifactor coagulopathy including Rhizopus oryzae infection in burns. J Burn Care Res. 2010 Nov-Dec;31(6):955-8. doi: 10.1097/BCR.0b013e3181f93912. PMID: 20859211. Link <https://pubmed.ncbi.nlm.nih.gov/20859211/>
853. Iida T, Sawada N, Takahashi M, Zendejas IR, Kayler LK, Magliocca JF, Kim RD, Hemming AW, Fujita S. Successful treatment of invasive mucormycosis in a liver transplant patient by arm amputation. Transplant Proc. 2010 Sep;42(7):2794-6. doi: 10.1016/j.transproceed.2010.06.001. PMID: 20832590. Link <https://pubmed.ncbi.nlm.nih.gov/20832590/>
854. Mimouni O, Curto CL, Danvin JB, Thomassin JM, Dessi P. Sinonasal mucormycosis: case report. Eur Ann Otorhinolaryngol Head Neck Dis. 2010 Mar;127(1):27-9. doi: 10.1016/j.anorl.2010.02.007. Epub 2010 Mar 30. PMID: 20822753. Link <https://pubmed.ncbi.nlm.nih.gov/20822753/>
855. Ukkola-Pons E, Weber-Donat G, Potet J, Fagot T, Kossowski M, Minvielle F, Baccialone J, Teriitehau C. Infections rhinocérébrales chez l'immunodéprimé en hématologie [Rhinocerebral infections in immunosuppressed patients with hematological disorders]. J Radiol. 2010 Jun;91(6):713-6. French. doi: 10.1016/s0221-0363(10)70102-5. PMID: 20808273. Link <https://pubmed.ncbi.nlm.nih.gov/20808273/>
856. Ojeda-Uribe M, Herbrecht R, Kiefer MH, Schultz P, Chain J, Chenard MP, Servant JM, Debry C. Lessons from a case of oromandibular mucormycosis treated with surgery and a combination of amphotericin B lipid formulation plus caspofungin. Acta Haematol. 2010;124(2):98-102. doi: 10.1159/000315675. Epub 2010 Aug 3. PMID: 20689269. Link <https://pubmed.ncbi.nlm.nih.gov/20689269/>
857. Ting JY, Chan SY, Lung DC, Ho AC, Chiang AK, Ha SY, Tsoi NN, Chan GC. Intra-abdominal Rhizopus microsporus infection successfully treated by combined aggressive surgical, antifungal, and iron chelating therapy. J Pediatr Hematol Oncol. 2010 Aug;32(6):e238-40. doi: 10.1097/MPH.0b013e3181e622bf. PMID: 20661158. Link <https://pubmed.ncbi.nlm.nih.gov/20661158/>
858. Chung JH, Godwin JD, Chien JW, Pipavath SJ. Case 160: Pulmonary mucormycosis. Radiology. 2010 Aug;256(2):667-70. doi: 10.1148/radiol.10081907. PMID: 20656848. Link <https://pubmed.ncbi.nlm.nih.gov/20656848/>
859. Kompoti M, Michalia M, Kallitsi G, Giannopoulou P, Arabatzis M, Liapi G, Velegraki A, Trikka-Graphakos E, Clouva-Molyvdas PM. Fatal cutaneous Saksenaea vasiformis infection in a critically ill trauma patient. Mycoses. 2011 Sep;54(5):e599-601. doi: 10.1111/j.1439-0507.2010.01902.x. Epub 2010 Jun 14. PMID: 20557464. Link <https://pubmed.ncbi.nlm.nih.gov/20557464/>
860. Borrás R, Roselló P, Chilet M, Bravo D, de Lomas JG, Navarro D. Positive result of the Aspergillus galactomannan antigen assay using bronchoalveolar lavage fluid from a patient with an invasive infection due to Lichtheimia ramosa. J Clin Microbiol. 2010 Aug;48(8):3035-6. doi: 10.1128/JCM.00902-10. Epub 2010 Jun 16. PMID: 20554823; PMCID: PMC2916633. Link <https://pubmed.ncbi.nlm.nih.gov/20554823/>
861. Maeda H, Kanzaki M, Isaka T, Onuki T. Successful resection of localized pulmonary mucormycosis after bone marrow transplantation using a virtual 3-dimensional pulmonary model on a personal computer. J Thorac Cardiovasc Surg. 2010 Dec;140(6):1434-6. doi: 10.1016/j.jtcvs.2010.05.007. Epub 2010 Jun 11. PMID: 20541225. Link <https://pubmed.ncbi.nlm.nih.gov/20541225/>
862. Gupta K, Nada R, Joshi K, Rohilla M, Walia R. Can ascending infection from bladder serve as the portal of entry for primary renal zygomycosis? Mycopathologia. 2010 Nov;170(5):357-60. doi: 10.1007/s11046-010-9329-y. Epub 2010 Jun 8. PMID: 20532826. Link <https://pubmed.ncbi.nlm.nih.gov/20532826/>
863. Wildenbeest JG, Oomen MW, Brüggemann RJ, de Boer M, Bijleveld Y, van den Berg JM, Kuijpers TW, Pajkrt D. Rhizopus oryzae skin infection treated with posaconazole in a boy with chronic granulomatous disease. Pediatr Infect Dis J. 2010 Jun;29(6):578. doi: 10.1097/INF.0b013e3181dc8352. PMID: 20508483. Link <https://pubmed.ncbi.nlm.nih.gov/20508483/>
864. Hyvernat H, Dunais B, Burel-Vandenbos F, Guidicelli S, Bernardin G, Gari-Toussaint M. Fatal peritonitis caused by Rhizopus microsporus. Med Mycol. 2010 Dec;48(8):1096-8. doi: 10.3109/13693786.2010.485281. Epub 2010 May 14. PMID: 20465520. Link <https://pubmed.ncbi.nlm.nih.gov/20465520/>
865. Wirths S, Schaub V, Adam P, Horger M, Faul C. Invasive Sinusitis nach allogener Stammzelltransplantation - Fall 4/2010 [Invasive sinusitis after allogenic stem cell transplantation - Case 4/2010]. Dtsch Med Wochenschr. 2010 May;135(20):1032. German. doi: 10.1055/s-0030-1247611. Epub 2010 May 11. PMID: 20461662. Link <https://pubmed.ncbi.nlm.nih.gov/20461662/>
866. Kusaba G, Ohsawa I, Ishii M, Inoshita H, Ohi H, Horikoshi S, Takase M, Yamaguchi Y, Tomino Y. Evidence of immunopathological traces in mucormycosis: an autopsy case. Clin Exp Nephrol. 2010 Aug;14(4):396-400. doi: 10.1007/s10157-010-0289-9. Epub 2010 May 12. PMID: 20461433. Link <https://pubmed.ncbi.nlm.nih.gov/20461433/>
867. Adalja A, Kaka AS. Necrotic arm lesions in an intravenous drug user. Intern Emerg Med. 2010 Oct;5(5):439-40. doi: 10.1007/s11739-010-0375-8. PMID: 20390464. Link <https://pubmed.ncbi.nlm.nih.gov/20390464/>
868. Kulendra K, Habibi M, Butler C, Clarke P, Howard D. Use of posaconazole in the treatment of infective rhinocerebral mucormycosis. J Laryngol Otol. 2010 Dec;124(12):1314-7. doi: 10.1017/S0022215110000678. Epub 2010 Apr 14. PMID: 20388246. Link <https://pubmed.ncbi.nlm.nih.gov/20388246/>
869. Giuliani A, Mettimano M, Viviani D, Scagliusi A, Bruno A, Russo A, Rotoli M, Savi L. An uncommon case of systemic Mucormycosis associated with spinal cord infarction in a recently diagnosed diabetic. Int J Immunopathol Pharmacol. 2010 Jan-Mar;23(1):355-8. doi: 10.1177/039463201002300135. PMID: 20378023. Link <https://pubmed.ncbi.nlm.nih.gov/20378023/>
870. Tsung LL, Zhu XL, Chu WC, Sun DT, Cheung KL, Leung TF. Intraventricular amphotericin for absidiomycosis in an immunocompetent child. Hong Kong Med J. 2010 Apr;16(2):137-40. PMID: 20354249. Link <https://pubmed.ncbi.nlm.nih.gov/20354249/>
871. Palacio-Bedoya F, Cadena JA, Thompson GR, Sutton DA, Owens AD, Patterson TF. A noninvasive renal fungus ball caused by Rhizopus--a previously unreported manifestation of zygomycosis. Med Mycol. 2010 Sep;48(6):866-9. doi: 10.3109/13693781003694796. PMID: 20353310. Link <https://pubmed.ncbi.nlm.nih.gov/20353310/>
872. Mayayo E, Landeyro J, Stchigel AM, Gazzoni A, Capilla J. Infiltración perineural por células fúngicas. Presentación de un caso y revisión de la literatura [Perineural spread by fungal cells. Case report and literature review]. Rev Iberoam Micol. 2010 Jun 30;27(2):94-7. Spanish. doi: 10.1016/j.riam.2009.12.002. Epub 2010 Mar 26. PMID: 20347372. Link <https://pubmed.ncbi.nlm.nih.gov/20347372/>
873. Koshy CG, Shah S, Mammen T. Subcutaneous emphysema of the chest: could it be pulmonary mucormycosis? Thorax. 2010 Mar;65(3):280. doi: 10.1136/thx.2009.115659. PMID: 20335307. Link <https://pubmed.ncbi.nlm.nih.gov/20335307/>
874. Mohanty D, Dhar M, Dwivedi S. Mucormycosis. Trop Doct. 2010 Apr;40(2):127-8. doi: 10.1258/td.2009.090209. PMID: 20305117. Link <https://pubmed.ncbi.nlm.nih.gov/20305117/>
875. Mitchell ME, McManus M, Dietz J, Camitta BM, Szabo S, Havens P. Absidia corymbifera endocarditis: survival after treatment of disseminated mucormycosis with radical resection of tricuspid valve and right ventricular free wall. J Thorac Cardiovasc Surg. 2010 Apr;139(4):e71-2. doi: 10.1016/j.jtcvs.2008.07.073. PMID: 20304127. Link <https://pubmed.ncbi.nlm.nih.gov/20304127/>
876. Mescam L, Lebeau B, Minet C, Pelloux H, Brambilla E, Sturm N. Thyroïdite chez une patiente immunodéprimée [Thyroiditis in an immunocompromised woman]. Ann Pathol. 2010 Feb;30(1):44-7. French. doi: 10.1016/j.annpat.2009.09.008. Epub 2010 Feb 4. PMID: 20223356. Link <https://pubmed.ncbi.nlm.nih.gov/20223356/>
877. McDermott NE, Barrett J, Hipp J, Merino MJ, Richard Lee CC, Waterman P, Domingo DL, Walsh TJ. Successful treatment of periodontal mucormycosis: report of a case and literature review. Oral Surg Oral Med Oral Pathol Oral Radiol Endod. 2010 Mar;109(3):e64-9. doi: 10.1016/j.tripleo.2009.11.012. PMID: 20219588. Link <https://pubmed.ncbi.nlm.nih.gov/20219588/>
878. Hofman V, Dhouibi A, Butori C, Padovani B, Gari-Toussaint M, Garcia-Hermoso D, Baumann M, Vénissac N, Cathomas G, Hofman P. Usefulness of molecular biology performed with formaldehyde-fixed paraffin embedded tissue for the diagnosis of combined pulmonary invasive mucormycosis and aspergillosis in an immunocompromised patient. Diagn Pathol. 2010 Jan 8;5:1. doi: 10.1186/1746-1596-5-1. PMID: 20205795; PMCID: PMC2823679. Link <https://pubmed.ncbi.nlm.nih.gov/20205795/>
879. Lo OS, Law WL. Ileocolonic mucormycosis in adult immunocompromised patients: a surgeon's perspective. World J Gastroenterol. 2010 Mar 7;16(9):1165-70. doi: 10.3748/wjg.v16.i9.1165. PMID: 20205292; PMCID: PMC2835798. Link <https://pubmed.ncbi.nlm.nih.gov/20205292/>
880. Jain S, Kapoor G. Severe life threatening neurotoxicity in a child with acute lymphoblastic leukemia receiving posaconazole and vincristine. Pediatr Blood Cancer. 2010 May;54(5):783. doi: 10.1002/pbc.22399. PMID: 20205256. Link <https://pubmed.ncbi.nlm.nih.gov/20205256/>
881. Aoki T, Kamezaki K, Miyamoto T, Nagafuji K, Mori Y, Yamauchi T, Takenaka K, Iwasaki H, Harada N, Shimono N, Teshima T, Akashi K. Cord blood stem cell transplantation in a patient with disseminated mucormycosis and acute myelogenous leukemia. Transpl Infect Dis. 2010 Jun;12(3):277-9. doi: 10.1111/j.1399-3062.2010.00496.x. Epub 2010 Feb 17. PMID: 20180927. Link https://pubmed.ncbi.nlm.nih.gov/20180927/
882. Li KW, Wen TF, Li GD. Hepatic mucormycosis mimicking hilar cholangiocarcinoma: a case report and literature review. World J Gastroenterol. 2010 Feb 28;16(8):1039-42. doi: 10.3748/wjg.v16.i8.1039. PMID: 20180248; PMCID: PMC2828593. Link <https://pubmed.ncbi.nlm.nih.gov/20180248/>
883. Lebeau O, Van Delden C, Garbino J, Robert J, Lamoth F, Passweg J, Chalandon Y. Disseminated Rhizopus microsporus infection cured by salvage allogeneic hematopoietic stem cell transplantation, antifungal combination therapy, and surgical resection. Transpl Infect Dis. 2010 Jun;12(3):269-72. doi: 10.1111/j.1399-3062.2009.00484.x. Epub 2010 Feb 16. PMID: 20163567. Link <https://pubmed.ncbi.nlm.nih.gov/20163567/>
884. Woo PC, Lau SK, Ngan AH, Tung ET, Leung SY, To KK, Cheng VC, Yuen KY. Lichtheimia hongkongensis sp. nov., a novel Lichtheimia spp. associated with rhinocerebral, gastrointestinal, and cutaneous mucormycosis. Diagn Microbiol Infect Dis. 2010 Mar;66(3):274-84. doi: 10.1016/j.diagmicrobio.2009.10.009. PMID: 20159375. Link <https://pubmed.ncbi.nlm.nih.gov/20159375/>
885. Hamadani M, Awan F, Villalona-Calero MA. Malignant thymoma with immunodeficiency (Good syndrome) associated with mucormycosis. Am J Clin Oncol. 2010 Feb;33(1):109. doi: 10.1097/COC.0b013e31802c5430. PMID: 20139744. Link <https://pubmed.ncbi.nlm.nih.gov/20139744/>
886. Garbino J, Myers C, Ambrosioni J, Gumy-Pause F. Report of a successful treatment of pulmonary Cunninghamella bertholletiae infection with liposomal amphotericin and posaconazole in a child with GvHD and review of the literature. J Pediatr Hematol Oncol. 2010 Mar;32(2):85-7. doi: 10.1097/MPH.0b013e3181c2bdce. PMID: 20118813. Link <https://pubmed.ncbi.nlm.nih.gov/20118813/>
887. Roux BG, Méchinaud F, Gay-Andrieu F, Lortholary O, Dannaoui E, Hoinard D, Corradini N. Successful triple combination therapy of disseminated absidia corymbifera infection in an adolescent with osteosarcoma. J Pediatr Hematol Oncol. 2010 Mar;32(2):131-3. doi: 10.1097/MPH.0b013e3181ca0dcf. PMID: 20098334. Link <https://pubmed.ncbi.nlm.nih.gov/20098334/>
888. Moriyama B, Torabi-Parizi P, Pratt AK, Henning SA, Pennick G, Shea YR, Roy Chowdhuri S, Rinaldi MG, Barrett AJ, Walsh TJ. Pharmacokinetics of liposomal amphotericin B in pleural fluid. Antimicrob Agents Chemother. 2010 Apr;54(4):1633-5. doi: 10.1128/AAC.01438-09. Epub 2010 Jan 19. PMID: 20086161; PMCID: PMC2849376. Link <https://pubmed.ncbi.nlm.nih.gov/20086161/>
889. Yi HS, Sym SJ, Park J, Cho EK, Shin DB, Lee JH. Typhlitis due to mucormycosis after chemotherapy in a patient with acute myeloid leukemia. Leuk Res. 2010 Jul;34(7):e173-5. doi: 10.1016/j.leukres.2009.12.022. Epub 2010 Jan 13. PMID: 20074799. Link <https://pubmed.ncbi.nlm.nih.gov/20074799/>
890. Sankar J, Arun S, Sankar MJ, Seth R, Thavraj V, Kabra SK, Vasantha M. 'Primary cutaneous mucormycosis during induction chemotherapy in a child with acute lymphoblastic leukemia'. Indian J Pediatr. 2009 Nov;76(11):1161-3. doi: 10.1007/s12098-009-0280-1. Epub 2010 Jan 14. PMID: 20072858. Link <https://pubmed.ncbi.nlm.nih.gov/20072858/>
891. Arce-Salinas CA, Pérez-Silva E. Mucormycosis complications in systemic lupus erythematosus. Lupus. 2010 Jul;19(8):985-8. doi: 10.1177/0961203309357574. Epub 2010 Jan 11. PMID: 20064915. Link <https://pubmed.ncbi.nlm.nih.gov/20064915/>
892. Leithauser M, Kahl C, Aepinus C, Prall F, Maruschke M, Riemer H, Wolff D, Jost K, Hilgendorf I, Freund M, Junghanss C. Invasive zygomycosis in patients with graft-versus-host disease after allogeneic stem cell transplantation. Transpl Infect Dis. 2010 Jun;12(3):251-7. doi: 10.1111/j.1399-3062.2009.00480.x. Epub 2009 Dec 7. PMID: 20002357. Link <https://pubmed.ncbi.nlm.nih.gov/20002357/>
893. Zaldivar RA, Leavitt JA, Griepentrog GJ, Woog JJ, Bradley EA. Rhino-orbital-cerebral mucormycosis: a lethal complication of body dysmorphic disorder. Ophthalmic Plast Reconstr Surg. 2009 Sep-Oct;25(5):398-9. doi: 10.1097/IOP.0b013e3181b54af3. PMID: 19966657. Link <https://pubmed.ncbi.nlm.nih.gov/19966657/>
894. Mousset S, Bug G, Heinz WJ, Tintelnot K, Rickerts V. Breakthrough zygomycosis on posaconazole prophylaxis after allogeneic stem cell transplantation. Transpl Infect Dis. 2010 Jun;12(3):261-4. doi: 10.1111/j.1399-3062.2009.00479.x. Epub 2009 Nov 30. PMID: 19954497. Link <https://pubmed.ncbi.nlm.nih.gov/19954497/>
895. Minet C, Bonadona A, Tabah A, Karkas A, Mescam L, Schwebel C, Hamidfar R, Pison C, Saint-Raymond C, Faure O, Salameire D, Timsit JF. Mucormycose disséminée d'évolution favorable chez une greffée pulmonaire [Non-fatal disseminated mucormycosis in a solid organ transplant]. Rev Mal Respir. 2009 Nov;26(9):998-1002. French. doi: 10.1016/s0761-8425(09)73337-6. PMID: 19953048. Link <https://pubmed.ncbi.nlm.nih.gov/19953048/>
896. Bansal S, Grover G, Grover M, Gupta AK. Isolated sphenoid mucormycosis presenting as visual impairment: changing trends? Am J Otolaryngol. 2010 Jan-Feb;31(1):64-6. doi: 10.1016/j.amjoto.2008.08.014. Epub 2009 Apr 23. PMID: 19944905. Link <https://pubmed.ncbi.nlm.nih.gov/19944905/>
897. Safder S, Carpenter JS, Roberts TD, Bailey N. The "Black Turbinate" sign: An early MR imaging finding of nasal mucormycosis. AJNR Am J Neuroradiol. 2010 Apr;31(4):771-4. doi: 10.3174/ajnr.A1808. Epub 2009 Nov 26. PMID: 19942703; PMCID: PMC7964235. Link <https://pubmed.ncbi.nlm.nih.gov/19942703/>
898. Shenoi S, Emery HM. Successful treatment of invasive gastric mucormycosis in a child with systemic lupus erythematosus. Lupus. 2010 Apr;19(5):646-9. doi: 10.1177/0961203309349117. Epub 2009 Nov 25. PMID: 19939907. Link <https://pubmed.ncbi.nlm.nih.gov/19939907/>
899. Kapur R, Aakalu VK, August CZ, Weiss RA. Mucormycosis infection of the lacrimal sac. Ophthalmic Plast Reconstr Surg. 2009 Nov-Dec;25(6):494-6. doi: 10.1097/IOP.0b013e3181b80e81. PMID: 19935261. Link <https://pubmed.ncbi.nlm.nih.gov/19935261/>
900. Kollmar N, Lakomek M, Kühnle I. Zygomycosis in a 13 year old girl with T-NHL. Klin Padiatr. 2009 Nov-Dec;221(6):382-3. doi: 10.1055/s-0029-1239555. Epub 2009 Nov 4. PMID: 19890792. Link <https://pubmed.ncbi.nlm.nih.gov/19890792/>
901. Scheckenbach K, Cornely O, Hoffmann TK, Engers R, Bier H, Chaker A, Greve J, Schipper J, Wagenmann M. Emerging therapeutic options in fulminant invasive rhinocerebral mucormycosis. Auris Nasus Larynx. 2010 Jun;37(3):322-8. doi: 10.1016/j.anl.2009.09.001. Epub 2009 Oct 25. PMID: 19857939. Link <https://pubmed.ncbi.nlm.nih.gov/19857939/>
902. Rassi SJ, Melkane AE, Rizk HG, Dahoui HA. Sinonasal mucormycosis in immunocompromised pediatric patients. J Pediatr Hematol Oncol. 2009 Dec;31(12):907-10. doi: 10.1097/MPH.0b013e3181bdbca0. PMID: 19855305. Link <https://pubmed.ncbi.nlm.nih.gov/19855305/>
903. Abuali MM, Posada R, Del Toro G, Roman E, Ramani R, Chaturvedi S, Chaturvedi V, LaBombardi VJ. Rhizomucor variabilis var. regularior and Hormographiella aspergillata infections in a leukemic bone marrow transplant recipient with refractory neutropenia. J Clin Microbiol. 2009 Dec;47(12):4176-9. doi: 10.1128/JCM.00305-09. Epub 2009 Oct 21. Erratum in: J Clin Microbiol. 2010 Mar;48(3):1018. PMID: 19846651; PMCID: PMC2786632. Link <https://pubmed.ncbi.nlm.nih.gov/19846651/>
904. Alvernia JE, Patel RN, Cai DZ, Dang N, Anderson DW, Melgar M. A successful combined endovascular and surgical treatment of a cranial base mucormycosis with an associated internal carotid artery pseudoaneurysm. Neurosurgery. 2009 Oct;65(4):733-40; discussion 740. doi: 10.1227/01.NEU.0000351773.74034.5E. PMID: 19834379. Link <https://pubmed.ncbi.nlm.nih.gov/19834379/>
905. Takashima R, Odaka M, Watanabe Y, Hirat K, Yoshida A. [Case of basilar artery occlusion caused by mucormycotic embolism in the course of myelodysplastic syndrome]. Brain Nerve. 2009 Sep;61(9):1079-82. Japanese. PMID: 19803408. Link <https://pubmed.ncbi.nlm.nih.gov/19803408/>
906. Chinen K, Matsumoto H, Fujioka Y. Cardiac mucormycosis presenting as a "fungus ball" in the left atrium. Intern Med. 2009;48(19):1781-2. doi: 10.2169/internalmedicine.48.2611. Epub 2009 Oct 1. PMID: 19797840. Link <https://pubmed.ncbi.nlm.nih.gov/19797840/>
907. Raymundo IT, Araújo BG, Costa Cde C, Tavares JP, Lima CG, Nascimento LA. Rhino-orbito-cerebral mucormycosis. Braz J Otorhinolaryngol. 2009 Jul-Aug;75(4):619. doi: 10.1016/s1808-8694(15)30505-x. PMID: 19784435. Link <https://pubmed.ncbi.nlm.nih.gov/19784435/>
908. Raymundo IT, Araújo BG, Costa Cde C, Tavares JP, Lima CG, Nascimento LA. Rhino-orbito-cerebral mucormycosis. Braz J Otorhinolaryngol. 2009 Jul-Aug;75(4):619. doi: 10.1016/s1808-8694(15)30505-x. PMID: 19784435. Link <https://pubmed.ncbi.nlm.nih.gov/19784435/>
909. Wall GC, Leman BI. Mucormycosis in a Crohn's disease patient treated with infliximab. Digestion. 2009;80(3):182-4. doi: 10.1159/000230676. Epub 2009 Sep 16. PMID: 19776582. Link <https://pubmed.ncbi.nlm.nih.gov/19776582/>
910. Burdick LM, Hamrock D, Mawhorter S, Tuthill R, Karai L. JAAD Grand Rounds quiz. Asymptomatic necrotic ulcer on leg. J Am Acad Dermatol. 2009 Jul;61(1):172-4. doi: 10.1016/j.jaad.2008.09.020. PMID: 19539869. Link <https://pubmed.ncbi.nlm.nih.gov/19539869/>
911. Chitsaz S, Bagheri J, Mandegar MH, Rayatzadeh H, Razavi J, Azadi L. Extensive sino-orbital zygomycosis after heart transplantation: a case report. Transplant Proc. 2009 Sep;41(7):2927-9. doi: 10.1016/j.transproceed.2009.07.001. PMID: 19765477. Link <https://pubmed.ncbi.nlm.nih.gov/19765477/>
912. Skiada A, Vrana L, Polychronopoulou H, Prodromou P, Chantzis A, Tofas P, Daikos GL. Disseminated zygomycosis with involvement of the central nervous system. Clin Microbiol Infect. 2009 Oct;15 Suppl 5:46-9. doi: 10.1111/j.1469-0691.2009.02980.x. PMID: 19754757. Link <https://pubmed.ncbi.nlm.nih.gov/19754757/>
913. Takahashi S, Horiguchi T, Mikami S, Kitamura Y, Kawase T. Subcortical intracerebral hemorrhage caused by mucormycosis in a patient with a history of bone-marrow transplantation. J Stroke Cerebrovasc Dis. 2009 Sep-Oct;18(5):405-6. doi: 10.1016/j.jstrokecerebrovasdis.2008.12.006. PMID: 19717028. Link <https://pubmed.ncbi.nlm.nih.gov/19717028/>
914. Tully CC, Romanelli AM, Sutton DA, Wickes BL, Hospenthal DR. Fatal Actinomucor elegans var. kuwaitiensis infection following combat trauma. J Clin Microbiol. 2009 Oct;47(10):3394-9. doi: 10.1128/JCM.00797-09. Epub 2009 Aug 12. PMID: 19675213; PMCID: PMC2756944. Link <https://pubmed.ncbi.nlm.nih.gov/19675213/>
915. Elinav H, Zimhony O, Cohen MJ, Marcovich AL, Benenson S. Rhinocerebral mucormycosis in patients without predisposing medical conditions: a review of the literature. Clin Microbiol Infect. 2009 Jul;15(7):693-7. doi: 10.1111/j.1469-0691.2009.02884.x. Epub 2009 Jul 16. PMID: 19624514. Link <https://pubmed.ncbi.nlm.nih.gov/19624514/>
916. Lekakis LJ, Lawson A, Prante J, Ribes J, Davis GJ, Monohan G, Baraboutis IG, Skoutelis AT, Howard DS. Fatal rhizopus pneumonia in allogeneic stem cell transplant patients despite posaconazole prophylaxis: two cases and review of the literature. Biol Blood Marrow Transplant. 2009 Aug;15(8):991-5. doi: 10.1016/j.bbmt.2009.04.007. Epub 2009 Jun 10. PMID: 19589489. Link <https://pubmed.ncbi.nlm.nih.gov/19589489/>
917. Sochaj M, Claridge M, Green NJ, Fox AD. Intravascular mucormycosis as a cause of arm ischemia in an immunocompromised patient. J Vasc Surg. 2009 Jul;50(1):193-4. doi: 10.1016/j.jvs.2009.01.057. PMID: 19563968. Link <https://pubmed.ncbi.nlm.nih.gov/19563968/>
918. Zhao Y, Zhang Q, Li L, Zhu J, Kang K, Chen L. Primary cutaneous mucormycosis caused by Rhizomucor variabilis in an immunocompetent patient. Mycopathologia. 2009 Nov;168(5):243-7. doi: 10.1007/s11046-009-9219-3. Epub 2009 Jun 27. PMID: 19562506. Link <https://pubmed.ncbi.nlm.nih.gov/19562506/>
919. Ramos A, Cuervas-Mons V, Noblejas A, Baños I, Duran P, Marcos R, Sánchez-Turrión V, Jiménez M, Arellano B, Corbacho C, Sánchez-Romero I. Breakthrough rhinocerebral mucormycosis in a liver transplant patient receiving caspofungin. Transplant Proc. 2009 Jun;41(5):1972-5. doi: 10.1016/j.transproceed.2009.01.077. PMID: 19545771. Link <https://pubmed.ncbi.nlm.nih.gov/19545771/>
920. Mezhir JJ, Mullane KM, Zarling J, Satoskar R, Pai RK, Roggin KK. Successful nonoperative management of gastrointestinal mucormycosis: novel therapy for invasive disease. Surg Infect (Larchmt). 2009 Oct;10(5):447-51. doi: 10.1089/sur.2008.049. PMID: 19485785. Link <https://pubmed.ncbi.nlm.nih.gov/19485785/>
921. Misaki H, Yamauchi T, Arai H, Yamamoto S, Sutoh H, Yoshida A, Tsutani H, Eguchi M, Nagoshi H, Naiki H, Baba H, Ueda T, Yamakawa M. Secondary malignant fibrous histiocytoma following refractory langerhans cell histiocytosis. J Clin Exp Hematop. 2009 May;49(1):33-7. doi: 10.3960/jslrt.49.33. PMID: 19474515. Link <https://pubmed.ncbi.nlm.nih.gov/19474515/>
922. Hatipoglu HG, Gurbuz MO, Yuksel E. Restricted diffusion in the optic nerve and retina demonstrated by MRI in rhino-orbital mucormycosis. J Neuroophthalmol. 2009 Mar;29(1):13-5. doi: 10.1097/WNO.0b013e318183bde4. PMID: 19458569. Link <https://pubmed.ncbi.nlm.nih.gov/19458569/>
923. Adegbola SO, Banerjee A, Mulcahy MP. Rhinocerebral mucormycosis. Br J Hosp Med (Lond). 2009 May;70(5):298-9. doi: 10.12968/hmed.2009.70.5.42238. PMID: 19451879. Link <https://pubmed.ncbi.nlm.nih.gov/19451879/>
924. Garg J, Sujatha S, Garg A, Parija SC. Nosocomial cutaneous zygomycosis in a patient with diabetic ketoacidosis. Int J Infect Dis. 2009 Nov;13(6):e508-10. doi: 10.1016/j.ijid.2009.02.018. Epub 2009 May 9. PMID: 19428282. Link <https://pubmed.ncbi.nlm.nih.gov/19428282/>
925. Jain D, Kohli K. Neonatal gastrointestinal mucormycosis clinically mimicking necrotizing enterocolitis. Eur J Pediatr Surg. 2009 Dec;19(6):405-7. doi: 10.1055/s-0029-1202251. PMID: 19408216. Link <https://pubmed.ncbi.nlm.nih.gov/19408216/>
926. Sales-Badía JG, Hervás VZ, Galbis-Caravajal JM. Mucormicosis traqueal [Tracheal mucormycosis]. Arch Bronconeumol. 2009 May;45(5):260-1. Spanish. doi: 10.1016/j.arbres.2009.01.010. Epub 2009 Apr 29. PMID: 19403226. Link <https://pubmed.ncbi.nlm.nih.gov/19403226/>
927. Saravia-Flores M, Guaran DM, Argueta V. Invasive cutaneous infection caused by Apophysomyces elegans associated with a spider bite. Mycoses. 2010 May;53(3):259-61. doi: 10.1111/j.1439-0507.2009.01698.x. Epub 2009 Mar 7. PMID: 19389070. Link <https://pubmed.ncbi.nlm.nih.gov/19389070/>
928. Hadzri MH, Azarisman SM, Fauzi AR, Kahairi A. Invasive rhinocerebral mucormycosis with orbital extension in poorly- controlled diabetes mellitus. Singapore Med J. 2009 Mar;50(3):e107-9. PMID: 19352553. Link <https://pubmed.ncbi.nlm.nih.gov/19352553/>
929. Piazza RC, Thomas WL, Stawski WS, Ford RD. Mucormycosis of the face. J Burn Care Res. 2009 May-Jun;30(3):520-3. doi: 10.1097/BCR.0b013e3181a28d2f. PMID: 19349886. Link Piazza RC, Thomas WL, Stawski WS, Ford RD. Mucormycosis of the face. J Burn Care Res. 2009 May-Jun;30(3):520-3. doi: 10.1097/BCR.0b013e3181a28d2f. PMID: 19349886. Link <https://pubmed.ncbi.nlm.nih.gov/19349886/>
930. Shah S, Suresh PV, Maheshwari S, Rao S. Cardiac mucormycosis with T-cell immunodeficiency. Indian Pediatr. 2009 Mar;46(3):257-9. PMID: 19346575. Link <https://pubmed.ncbi.nlm.nih.gov/19346575/>
931. Marom T, Watad W, Dan M, Roth Y. Posaconazole for bone-invading rhinomaxillary mucormycosis. Otolaryngol Head Neck Surg. 2009 Apr;140(4):610-1. doi: 10.1016/j.otohns.2008.11.022. PMID: 19328359. Link <https://pubmed.ncbi.nlm.nih.gov/19328359/>
932. El Fakih R, Saliba W, Mortada R. Quiz page. Disseminated zygomycosis with kidney microinfarction and pulmonary abscess. Am J Kidney Dis. 2009 Apr;53(4):xxxvii-xxxix. doi: 10.1053/j.ajkd.2008.08.018. PMID: 19324243. Link <https://pubmed.ncbi.nlm.nih.gov/19324243/>
933. Cavrini F, Stanzani M, Liguori G, Sambri V. Identification of an invasive infection of R. oryzae in a haematological patient using a molecular technique. Mycoses. 2010 May;53(3):269-71. doi: 10.1111/j.1439-0507.2009.01689.x. Epub 2009 Mar 17. PMID: 19302460. Link. <https://pubmed.ncbi.nlm.nih.gov/19302460/>
934. Koonce RC, Price CS, Sutton DA, Wickes BL, Montero PN, Morgan SJ. Lower-extremity zygomycosis in a patient with traumatic injuries. A case report. J Bone Joint Surg Am. 2009 Mar 1;91(3):686-92. doi: 10.2106/JBJS.H.00187. PMID: 19255231. Link <https://pubmed.ncbi.nlm.nih.gov/19255231/>
935. Ibrahim M, Chitnis S, Fallon K, Roberts T. Rhinocerebral mucormycosis in a 12-year-old girl. Arch Neurol. 2009 Feb;66(2):272-3. doi: 10.1001/archneurol.2008.546. PMID: 19204168. Link <https://pubmed.ncbi.nlm.nih.gov/19204168/>
936. Fouzi S, Ayadi H, Sellami A, Rekik WK, Guermazi N, Ayadi A, Ayoub A. Une opacité pulmonaire excavée chez un diabétique [Excavated pulmonary opacity in diabetes]. Ann Endocrinol (Paris). 2009 Apr;70(2):137-40. French. doi: 10.1016/j.ando.2008.11.001. Epub 2009 Feb 7. PMID: 19201393. Link <https://pubmed.ncbi.nlm.nih.gov/19201393/>
937. Ganesan P, Swaroop C, Ahuja A, Thulkar S, Bakhshi S. Early onset sinonasal mucormycosis during induction therapy of acute lymphoblastic leukemia: good outcome without surgical intervention. J Pediatr Hematol Oncol. 2009 Feb;31(2):152-3. doi: 10.1097/MPH.0b013e318191471c. PMID: 19194208. Link <https://pubmed.ncbi.nlm.nih.gov/19194208/>
938. De Pasqual A, Deprez M, Ghaye B, Frère P, Kaschten B, Hayette MP, Radermecker M, Martin D, Canivet JL. Mucormycose invasive du poumon et du rachis dorsal [Invasive pulmonary mucormycosis with invasion of the thoracic spine in a patient with myelodysplastic syndrome]. Rev Med Liege. 2008 Dec;63(12):702-6. French. PMID: 19180827. Link <https://pubmed.ncbi.nlm.nih.gov/19180827/>
939. de Mol P, Meis JF. Disseminated Rhizopus microsporus infection in a patient on oral corticosteroid treatment: a case report. Neth J Med. 2009 Jan;67(1):25-8. PMID: 19155544. Link <https://pubmed.ncbi.nlm.nih.gov/19155544/>
940. Lüer S, Berger S, Diepold M, Duppenthaler A, von Gunten M, Mühlethaler K, Wolf R, Aebi C. Treatment of intestinal and hepatic mucormycosis in an immunocompromized child. Pediatr Blood Cancer. 2009 Jul;52(7):872-4. doi: 10.1002/pbc.21918. PMID: 19127570. Link <https://pubmed.ncbi.nlm.nih.gov/19127570/>
941. Fahimzad A, Chavoshzadeh Z, Abdollahpour H, Klein C, Rezaei N. Necrosis of nasal cartilage due to mucormycosis in a patient with severe congenital neutropenia due to HAX1 deficiency. J Investig Allergol Clin Immunol. 2008;18(6):469-72. PMID: 19123440. Link <https://pubmed.ncbi.nlm.nih.gov/19123440/>
942. Martín-Moro JG, Calleja JM, García MB, Carretero JL, Rodríguez JG. Rhinoorbitocerebral mucormycosis: a case report and literature review. Med Oral Patol Oral Cir Bucal. 2008 Dec 1;13(12):E792-5. PMID: 19047969. Link <https://pubmed.ncbi.nlm.nih.gov/19047969/>
943. Motohashi K, Ito S, Hagihara M, Maruta A, Ishigatsubo Y, Kanamori H. Cutaneous zygomycosis caused by Cunninghamella bertholletiae in a patient with chronic myelogenous leukemia in blast crisis. Am J Hematol. 2009 Jul;84(7):447-8. doi: 10.1002/ajh.21289. PMID: 19021120. Link <https://pubmed.ncbi.nlm.nih.gov/19021120/>
944. Kasliwal MK, Reddy VS, Sinha S, Sharma BS, Das P, Suri V. Bilateral anterior cerebral artery aneurysm due to mucormycosis. J Clin Neurosci. 2009 Jan;16(1):156-9. doi: 10.1016/j.jocn.2008.04.019. Epub 2008 Nov 14. PMID: 19013802. Link <https://pubmed.ncbi.nlm.nih.gov/19013802/>
945. Trotter DJ, Gonis G, Cottrill E, Coombs C. Disseminated Saksenaea vasiformis in an immunocompetent host. Med J Aust. 2008 Nov 3;189(9):519-20. doi: 10.5694/j.1326-5377.2008.tb02149.x. PMID: 18976196. Link <https://pubmed.ncbi.nlm.nih.gov/18976196/>
946. Sharma A, Gupta V, Singh RS, Kakkar N, Singh S, Bambery P. Angioinvasive pulmonary mucormycosis presenting as multiple bilateral pulmonary nodules in a patient without obvious predisposing factors. Singapore Med J. 2008 Oct;49(10):e269-71. PMID: 18946595. Link <https://pubmed.ncbi.nlm.nih.gov/18946595/>
947. Corti G, Mondanelli N, Losco M, Bartolini L, Fontanelli A, Paradisi F. Post-traumatic infection of the lower limb caused by rare Enterobacteriaceae and Mucorales in a young healthy male. Int J Infect Dis. 2009 Mar;13(2):e57-60. doi: 10.1016/j.ijid.2008.06.029. Epub 2008 Oct 21. PMID: 18945631. Link <https://pubmed.ncbi.nlm.nih.gov/18945631/>
948. Madrigal B, Arenal JJ, Torres A, Peñarrubia MJ, Vara A, Ruiz M, Hernández A, Enríquez P. Mucormicosis yeyunal en paciente con linfoma de Hodgkin [Jejunal mucormycosis in a patient with Hodgkin's lymphoma]. Rev Esp Enferm Dig. 2008 Aug;100(8):507-10. Spanish. doi: 10.4321/s1130-01082008000800011. PMID: 18942905. Link <https://pubmed.ncbi.nlm.nih.gov/18942905/>
949. Fanci R, Pecile P, Di Lollo S, Dini C, Bosi A. Pulmonary mucormycosis with cervical lymph node involvement in a patient with acute myeloid leukaemia: a case report. Mycoses. 2008 Jul;51(4):354-6. doi: 10.1111/j.1439-0507.2008.01494.x. PMID: 18855847. Link <https://pubmed.ncbi.nlm.nih.gov/18855847/>
950. Tarani L, Costantino F, Notheis G, Wintergerst U, Venditti M, Di Biasi C, Friederici D, Pasquino AM. Long-term posaconazole treatment and follow-up of rhino-orbital-cerebral mucormycosis in a diabetic girl. Pediatr Diabetes. 2009 Jun;10(4):289-93. doi: 10.1111/j.1399-5448.2008.00465.x. Epub 2009 Sep 25. PMID: 18828793. Link <https://pubmed.ncbi.nlm.nih.gov/18828793/>
951. Itoh Y, Segawa H, Kito K, Hodohara K, Ishigaki H, Sugihara H, Fujiyama Y, Ogasawara K. Lipoid pneumonia with chronic myelomonocytic leukemia. Pathol Res Pract. 2009;205(2):143-7. doi: 10.1016/j.prp.2008.07.013. Epub 2008 Sep 20. PMID: 18805647. Link <https://pubmed.ncbi.nlm.nih.gov/18805647/>
952. Inagaki N, Sugimoto K, Hosone M, Isobe Y, Yamamoto Y, Sasaki M, Kato A, Mori T, Oshimi K. Disseminated Mucor infection and thrombotic microangiopathy in lymphoma-associated hemophagocytic syndrome. Int J Hematol. 2008 Oct;88(3):355-356. doi: 10.1007/s12185-008-0156-6. Epub 2008 Sep 6. PMID: 18773259. Link <https://pubmed.ncbi.nlm.nih.gov/18773259/>
953. Turunc T, Demiroglu YZ, Aliskan H, Colakoglu S, Arslan H. Eleven cases of mucormycosis with atypical clinical manifestations in diabetic patients. Diabetes Res Clin Pract. 2008 Nov;82(2):203-8. doi: 10.1016/j.diabres.2008.07.011. Epub 2008 Aug 28. PMID: 18760493. Link <https://pubmed.ncbi.nlm.nih.gov/18760493/>
954. Gattenlöhner S, Unzicker C, Wörner S, Stuhler G, Einsele H, Müller-Hermelink HK. Disseminated mucormycosis of an immunocompromised multiple myeloma patient: impact of biopsy of extramedullary tumours in refractory multiple myeloma. Ann Hematol. 2009 Apr;88(4):385-7. doi: 10.1007/s00277-008-0594-5. Epub 2008 Aug 29. PMID: 18758780. Link <https://pubmed.ncbi.nlm.nih.gov/18758780/>
955. Kishel JJ, Sivik J. Breakthrough invasive fungal infection in an immunocompromised host while on posaconazole prophylaxis: an omission in patient counseling and follow-up. J Oncol Pharm Pract. 2008 Dec;14(4):189-93. doi: 10.1177/1078155208094123. Link <https://pubmed.ncbi.nlm.nih.gov/18753183/>
956. Chew HH, Abuzeid A, Singh D, Tai CC. Surgical wound mucormycosis necessitating hand amputation: a case report. J Orthop Surg (Hong Kong). 2008 Aug;16(2):267-9. doi: 10.1177/230949900801600230. PMID: 18725688. Link <https://pubmed.ncbi.nlm.nih.gov/18725688/>
957. ägle C, Papageorgiou E, Preyer S, Löwenheim H, Schiefer U, Jägle H. Fulminanter Verlauf einer orbitalen Mucoracea-Infektion [Fulminant process of an orbital Mucoracea infection]. Ophthalmologe. 2009 Jun;106(6):547-50. German. doi: 10.1007/s00347-008-1830-z. PMID: 18709374. Link <https://pubmed.ncbi.nlm.nih.gov/18709374/>
958. Chopra H, Dua K, Bhatia S, Dua N, Mittal V. Invasive rhino-orbital fungal sinusitis following dental manipulation. Mycoses. 2009 Jul;52(4):368-71. doi: 10.1111/j.1439-0507.2008.01600.x. Epub 2008 Aug 13. PMID: 18705660. Link <https://pubmed.ncbi.nlm.nih.gov/18705660/>
959. Chen CK, Wan SH, Kou SK. A rare cutaneous fungal infection complicating bacterial necrotising fasciitis. Hong Kong Med J. 2008 Aug;14(4):314-6. PMID: 18685166. Link <https://pubmed.ncbi.nlm.nih.gov/18685166/>
960. Lechevalier P, Hermoso DG, Carol A, Bonacorsi S, Ferkdadji L, Fitoussi F, Lortholary O, Bourrillon A, Faye A, Dannaoui E, Angoulvant F. Molecular diagnosis of Saksenaea vasiformis cutaneous infection after scorpion sting in an immunocompetent adolescent. J Clin Microbiol. 2008 Sep;46(9):3169-72. doi: 10.1128/JCM.00052-08. Epub 2008 Jul 16. PMID: 18632909; PMCID: PMC2546755. Link <https://pubmed.ncbi.nlm.nih.gov/18632909/>
961. Page AV, Evans AJ, Snell L, Liles WC. Primary cutaneous mucormycosis in a lung transplant recipient: case report and concise review of the literature. Transpl Infect Dis. 2008 Dec;10(6):419-25. doi: 10.1111/j.1399-3062.2008.00324.x. Epub 2008 Jun 24. PMID: 18627579. Link <https://pubmed.ncbi.nlm.nih.gov/18627579/>
962. Shiva Prasad BN, Shenoy A, Nataraj KS. Primary gastrointestinal mucormycosis in an immunocompetent person. J Postgrad Med. 2008 Jul-Sep;54(3):211-3. doi: 10.4103/0022-3859.41805. PMID: 18626171. Link <https://pubmed.ncbi.nlm.nih.gov/18626171/>
963. Schlemmer F, Lagrange-Xélot M, Lacroix C, de La Tour R, Socié G, Molina JM. Breakthrough Rhizopus infection on posaconazole prophylaxis following allogeneic stem cell transplantation. Bone Marrow Transplant. 2008 Oct;42(8):551-2. doi: 10.1038/bmt.2008.199. Epub 2008 Jul 14. PMID: 18622416. Link <https://pubmed.ncbi.nlm.nih.gov/18622416/>
964. Garner D, Machin K. Investigation and management of an outbreak of mucormycosis in a paediatric oncology unit. J Hosp Infect. 2008 Sep;70(1):53-9. doi: 10.1016/j.jhin.2008.05.017. Epub 2008 Jul 14. PMID: 18621437. Link <https://pubmed.ncbi.nlm.nih.gov/18621437/>
965. Metallidis S, Chrysanthidis T, Kazakos E, Saraf A, Nikolaidis P. A fatal case of pacemaker lead endocarditis caused by Mucor spp. Int J Infect Dis. 2008 Nov;12(6):e151-2. doi: 10.1016/j.ijid.2008.03.034. Epub 2008 Jul 11. PMID: 18620884. Link <https://pubmed.ncbi.nlm.nih.gov/18620884/>
966. Mysorekar VV, Rao SG. Cytomegalovirus pneumonia with pulmonary mucormycosis. Indian J Pathol Microbiol. 2008 Apr-Jun;51(2):294-5. doi: 10.4103/0377-4929.41704. PMID: 18603715. Link <https://pubmed.ncbi.nlm.nih.gov/18603715/>
967. Zhan HX, Lv Y, Zhang Y, Liu C, Wang B, Jiang YY, Liu XM. Hepatic and renal artery rupture due to Aspergillus and Mucor mixed infection after combined liver and kidney transplantation: a case report. Transplant Proc. 2008 Jun;40(5):1771-3. doi: 10.1016/j.transproceed.2007.10.013. PMID: 18589192. Link <https://pubmed.ncbi.nlm.nih.gov/18589192/>
968. Mitchell GE, Reddy A, Shepherd ME. Acute oral and ocular changes in a patient with diabetic ketoacidosis. Am Fam Physician. 2008 Jun 1;77(11):1584, 1586-7. PMID: 18581840. Link <https://pubmed.ncbi.nlm.nih.gov/18581840/>
969. Ziakas PD, Adraktas P, Ntountas J, Skarpidi E, Filippi V, Kechagias D, Karianakis G. Isolated orbital mucormycosis: a rare presentation ending in a fatal outcome. Int J Infect Dis. 2009 Jan;13(1):112-4. doi: 10.1016/j.ijid.2008.01.015. Epub 2008 Jun 24. PMID: 18573673. Link <https://pubmed.ncbi.nlm.nih.gov/18573673/>
970. Chung CS, Wang WL, Liu KL, Lin JT, Wang HP. Green ulcer in the stomach: unusual mucormycosis infection. Gastrointest Endosc. 2008 Sep;68(3):566-7; discussion 567. doi: 10.1016/j.gie.2008.02.076. Epub 2008 Jun 17. PMID: 18561932. Link <https://pubmed.ncbi.nlm.nih.gov/18561932/>
971. Nomiya R, Nomiya S, Paparella MM. Mucormycosis of the temporal bone. Otol Neurotol. 2008 Oct;29(7):1041-2. doi: 10.1097/MAO.0b013e31817d0200. PMID: 18520621. Link <https://pubmed.ncbi.nlm.nih.gov/18520621/>
972. Baig WW, Ravindra Prabhu A, Natraj KS, Mathew M. Combined mucormycosis and candidiasis of the cecum presenting as a right iliac fossa mass in a patient with chronic kidney disease. Travel Med Infect Dis. 2008 May;6(3):145-7. doi: 10.1016/j.tmaid.2008.01.012. Epub 2008 Mar 18. PMID: 18486071. Link <https://pubmed.ncbi.nlm.nih.gov/18486071/>
973. Nalmas S, Bishburg E, Goldstein C. Mucormycosis in a transplanted kidney. Transpl Infect Dis. 2008 Jul;10(4):269-71. doi: 10.1111/j.1399-3062.2008.00314.x. Epub 2008 May 6. PMID: 18466194. Link <https://pubmed.ncbi.nlm.nih.gov/18466194/>
974. Ammari L, Kilani B, Tiouiri H, Kanoun F, Goubontini A, Mnif E, Zouiten F, Chaker E, Ben Chaabane T. Mucormycosis: four case reports. Tunis Med. 2008 Feb;86(2):165-8. PMID: 18444535. Link <https://pubmed.ncbi.nlm.nih.gov/18444535/>
975. Dhingra KK, Mandal S, Khurana N. Unsuspected intestinal mucormycosis in a neonate presenting as necrotizing enterocolitis (NEC). Eur J Pediatr Surg. 2008 Apr;18(2):119-20. doi: 10.1055/s-2007-965748. PMID: 18437658. Link <https://pubmed.ncbi.nlm.nih.gov/18437658/>
976. Johnson KE, Leahy K, Owens C, Blankson JN, Merz WG, Goldstein BJ. An atypical case of fatal zygomycosis: simultaneous cutaneous and laryngeal infection in a patient with a non-neutropenic solid prostatic tumor. Ear Nose Throat J. 2008 Mar;87(3):152-5. PMID: 18404912. Link <https://pubmed.ncbi.nlm.nih.gov/18404912/>
977. Pasticci MB, Terenzi A, Lapalorcia LM, Giovenale P, Pitzurra L, Costantini V, Lignani A, Gurdo G, Verzini F, Baldelli F. Absidia corymbifera necrotizing cellulitis in an immunocompromised patient while on voriconazole treatment. Ann Hematol. 2008 Aug;87(8):687-9. doi: 10.1007/s00277-008-0468-x. Epub 2008 Apr 10. PMID: 18401584. Link <https://pubmed.ncbi.nlm.nih.gov/18401584/>
978. Devi SC, Kanungo R, Barreto E, Thomas AG, Shashikala N, Srinivasan S, Anitha PK. Favorable outcome of amphotericin B treatment of zygomycotic necrotizing fascitis caused by Apophysomyces elegans. Int J Dermatol. 2008 Apr;47(4):407-9. doi: 10.1111/j.1365-4632.2008.03622.x. PMID: 18377611. Link <https://pubmed.ncbi.nlm.nih.gov/18377611/>
979. Jin H, Qiao J, Wang B, Wang H, Sun Q. Toxic epidermal necrolysis complicated by Mucor infection. Int J Dermatol. 2008 Apr;47(4):383-6. doi: 10.1111/j.1365-4632.2008.03421.x. PMID: 18377605. Link <https://pubmed.ncbi.nlm.nih.gov/18377605/>
980. Popa G, Blag C, Sasca F. Rhinocerebral mucormycosis in a child with acute lymphoblastic leukemia: a case report. J Pediatr Hematol Oncol. 2008 Feb;30(2):163-5. doi: 10.1097/MPH.0b013e31815c255f. PMID: 18376271. Link <https://pubmed.ncbi.nlm.nih.gov/18376271/>
981. Odessey E, Cohn A, Beaman K, Schechter L. Invasive mucormycosis of the maxillary sinus: extensive destruction with an indolent presentation. Surg Infect (Larchmt). 2008 Feb;9(1):91-8. doi: 10.1089/sur.2006.039. PMID: 18363473. Link <https://pubmed.ncbi.nlm.nih.gov/18363473/>
982. Shetty S, Kini U, Joy R. Isolated lingual mucormycosis in an infant with Down syndrome. Ear Nose Throat J. 2008 Jan;87(1):34-5, 43. PMID: 18357944. Link <https://pubmed.ncbi.nlm.nih.gov/18357944/>
983. Constantinides J, Misra A, Nassab R, Wilson Y. Absidia corymbifera fungal infection in burns: a case report and review of the literature. J Burn Care Res. 2008 Mar-Apr;29(2):416-9. doi: 10.1097/BCR.0b013e318166da78. PMID: 18354306. Link <https://pubmed.ncbi.nlm.nih.gov/18354306/>
984. Ledgard JP, van Hal S, Greenwood JE. Primary cutaneous zygomycosis in a burns patient: a review. J Burn Care Res. 2008 Mar-Apr;29(2):286-90. doi: 10.1097/BCR.0b013e31816673b1. PMID: 18354283. Link <https://pubmed.ncbi.nlm.nih.gov/18354283/>
985. Bakr A, Wafa E, Fouda A, Elagroudy A, Gheith O, Sobh M, Shokeir A, Ghoneim M. Successful treatment of mucormycosis in a renal allograft recipient. Clin Exp Nephrol. 2008 Jun;12(3):207-10. doi: 10.1007/s10157-008-0028-7. Epub 2008 Mar 20. PMID: 18351288. Link <https://pubmed.ncbi.nlm.nih.gov/18351288/>
986. Righi E, Giacomazzi CG, Lindstrom V, Albarello A, Soro O, Miglino M, Perotti M, Varnier OE, Gobbi M, Viscoli C, Bassetti M. A case of Cunninghamella bertholettiae rhino-cerebral infection in a leukaemic patient and review of recent published studies. Mycopathologia. 2008 Jun;165(6):407-10. doi: 10.1007/s11046-008-9098-z. Epub 2008 Mar 14. PMID: 18340546. Link <https://pubmed.ncbi.nlm.nih.gov/18340546/>
987. agi S, Miyashita N, Fukuda M, Obase Y, Yoshida K, Miyauchi A, Kawasaki K, Soda H, Oka M. Pulmonary mucormycosis (Cunninghamella bertholletiae) with cavitation diagnosed using ultra-thin fibre-optic bronchoscopy. Respirology. 2008 Mar;13(2):312-4. doi: 10.1111/j.1440-1843.2008.01240.x. PMID: 18339037. Link <https://pubmed.ncbi.nlm.nih.gov/18339037/>
988. Koyama N, Nagata M, Hagiwara K, Kanazawa M. Survival of a patient with pulmonary Cunninghamella bertholletiae infection without surgical intervention. Respirology. 2008 Mar;13(2):309-11. doi: 10.1111/j.1440-1843.2008.01234.x. PMID: 18339036. Link <https://pubmed.ncbi.nlm.nih.gov/18339036/>
989. Song YM, Shin SY. Bilateral ophthalmic artery occlusion in rhino-orbito-cerebral mucormycosis. Korean J Ophthalmol. 2008 Mar;22(1):66-9. doi: 10.3341/kjo.2008.22.1.66. PMID: 18323710; PMCID: PMC2629957. Link <https://pubmed.ncbi.nlm.nih.gov/18323710/>
990. Holley A, Mayes D, Browning R. A 40-year-old man with neutropenic fever and lobar consolidation. Chest. 2008 Mar;133(3):816-9. doi: 10.1378/chest.07-1627. PMID: 18321912. Link <https://pubmed.ncbi.nlm.nih.gov/18321912/>
991. Haliloglu NU, Yesilirmak Z, Erden A, Erden I. Rhino-orbito-cerebral mucormycosis: report of two cases and review of the literature. Dentomaxillofac Radiol. 2008 Mar;37(3):161-6. doi: 10.1259/dmfr/14698002. PMID: 18316508. Link <https://pubmed.ncbi.nlm.nih.gov/18316508/>
992. Dojcinovic I, Richter M. Mucormycoses: serious complication of high-dose corticosteroid therapy for traumatic optic neuropathy. Int J Oral Maxillofac Surg. 2008 Apr;37(4):391-4. doi: 10.1016/j.ijom.2007.09.177. Epub 2008 Mar 7. PMID: 18316177. Link <https://pubmed.ncbi.nlm.nih.gov/18316177/>
993. Dave SP, Vivero RJ, Roy S. Facial cutaneous mucormycosis in a full-term infant. Arch Otolaryngol Head Neck Surg. 2008 Feb;134(2):206-9. doi: 10.1001/archoto.2007.48. PMID: 18283166. Link <https://pubmed.ncbi.nlm.nih.gov/18283166/>
994. Koklu E, Akcakus M, Torun YA, Tulpar S, Tasdemir A. Primary gangrenous cutaneous mucormycosis of the scalp in a child: a case report. Pediatr Emerg Care. 2008 Feb;24(2):102-4. doi: 10.1097/PEC.0b013e318163dbf7. PMID: 18277847. Link <https://pubmed.ncbi.nlm.nih.gov/18277847/>
995. Berne JD, Villarreal DH, McGovern TM, Rowe SA, Moore FO, Norwood SH. A fatal case of posttraumatic gastric mucormycosis. J Trauma. 2009 Mar;66(3):933-5. doi: 10.1097/01.ta.0000233673.30138.0c. PMID: 18277268. Link <https://pubmed.ncbi.nlm.nih.gov/18277268/>
996. Eichna DM, Brown KS, Breen A, Dean RB. Mucormycosis: a rare but serious infection. Clin J Oncol Nurs. 2008 Feb;12(1):108-12. doi: 10.1188/08.CJON.108-112. PMID: 18258580. Link <https://pubmed.ncbi.nlm.nih.gov/18258580/>
997. Sedlacek M, Cotter JG, Suriawinata AA, Kaneko TM, Zuckerman RA, Parsonnet J, Block CA. Mucormycosis peritonitis: more than 2 years of disease-free follow-up after posaconazole salvage therapy after failure of liposomal amphotericin B. Am J Kidney Dis. 2008 Feb;51(2):302-6. doi: 10.1053/j.ajkd.2007.09.026. PMID: 18215708. Link <https://pubmed.ncbi.nlm.nih.gov/18215708/>
998. Quintano Ridero A, Rodríguez Borregán JC, Martín Fojaco J, Marco Moreno JM. Imagen de la semana. Mucormicosis rinoorbitaria [Image of the week. Rhino-orbital mucormycosis]. Med Clin (Barc). 2007 Nov 17;129(18):720. Spanish. doi: 10.1016/s0025-7753(07)72976-7. PMID: 18203338. Link <https://pubmed.ncbi.nlm.nih.gov/18203338/>
999. Clauss H, Samuel R. Simultaneous mold infections in an orthotopic heart transplant recipient. Transpl Infect Dis. 2008 Oct;10(5):343-5. doi: 10.1111/j.1399-3062.2007.00299.x. Epub 2008 Jan 9. PMID: 18194369. Link <https://pubmed.ncbi.nlm.nih.gov/18194369/>
1000. Gadadhar H, Hawkins S, Huffstutter JE, Panda M. Cutaneous mucormycosis complicating methotrexate, prednisone, and infliximab therapy. J Clin Rheumatol. 2007 Dec;13(6):361-2. doi: 10.1097/RHU.0b013e31815d3ddd. PMID: 18176152. Link <https://pubmed.ncbi.nlm.nih.gov/18176152/>
1001. Parra-Ruiz J, Peña-Monje A, Tomas-Jimenez C, Antelo-Lorenzo R, Escobar-Lara T, Hernández-Quero J. Septic arthritis due to Absidia corymbifera in a patient with HIV-1 infection. Infection. 2008 Jun;36(3):279-81. doi: 10.1007/s15010-007-6297-3. Epub 2007 Dec 14. PMID: 18084717. Link <https://pubmed.ncbi.nlm.nih.gov/18084717/>
1002. Samet JD, Horton KM, Fishman EK. Invasive gastric mucormycosis: CT findings. Emerg Radiol. 2008 Sep;15(5):349-51. doi: 10.1007/s10140-007-0689-7. Epub 2007 Dec 11. PMID: 18071767. Link <https://pubmed.ncbi.nlm.nih.gov/18071767/>
1003. Abinun M, Wright C, Gould K, Flood TJ, Cassidy J. Absidia corymbifera in a patient with chronic granulomatous disease. Pediatr Infect Dis J. 2007 Dec;26(12):1167-8. doi: 10.1097/INF.0b013e31815a0a2c. PMID: 18043464. Link <https://pubmed.ncbi.nlm.nih.gov/18043464/>
1004. Mimouni O, Curto CL, Gallucci A, Chossegros C, Deiss P. Une sinusite traînante [A long-lasting sinusitis]. Rev Stomatol Chir Maxillofac. 2008 Sep;109(4):249-50. French. doi: 10.1016/j.stomax.2007.06.028. Epub 2007 Nov 26. PMID: 18037147. Link <https://pubmed.ncbi.nlm.nih.gov/18037147/>
1005. Righi E, Giacomazzi CG, Bassetti M, Bisio F, Soro O, McDermott JL, Varnier OE, Ratto S, Viscoli C. Soft-tissue infection with Absidia corymbifera and kidney complications in an AIDS patient. Med Mycol. 2007 Nov;45(7):637-40. doi: 10.1080/13693780701435358. PMID: 18033616. Link <https://pubmed.ncbi.nlm.nih.gov/18033616/>
1006. Tayyebi N, Amouian S, Mohamadian N, Rahimi HR. Renal allograft mucormycosis: report of two cases. Surg Infect (Larchmt). 2007 Oct;8(5):535-8. doi: 10.1089/sur.2006.058. PMID: 17999588. Link <https://pubmed.ncbi.nlm.nih.gov/17999588/>
1007. Carpenter M, Polk C, Castellani R, Mochoruk K, Sanche S, Stern B, Donnenberg MS. Encephalitis of the basal ganglia in an injection drug user. Clin Infect Dis. 2007 Dec 1;45(11):1479, 1522-4. doi: 10.1086/522995. PMID: 17990237. Link <https://pubmed.ncbi.nlm.nih.gov/17990237/>
1008. Koc Z, Koc F, Yerdelen D, Ozdogu H. Rhino-orbital-cerebral mucormycosis with different cerebral involvements: infarct, hemorrhage, and ophthalmoplegia. Int J Neurosci. 2007 Dec;117(12):1677-90. doi: 10.1080/00207450601050238. PMID: 17987470. Link <https://pubmed.ncbi.nlm.nih.gov/17987470/>
1009. Jung JH, Choi HJ, Yoo J, Kang SJ, Lee KY. Emphysematous gastritis associated with invasive gastric mucormycosis: a case report. J Korean Med Sci. 2007 Oct;22(5):923-7. doi: 10.3346/jkms.2007.22.5.923. PMID: 17982248; PMCID: PMC2693866. Link <https://pubmed.ncbi.nlm.nih.gov/17982248/>
1010. Islam MN, Cohen DM, Celestina LJ, Ojha J, Claudio R, Bhattacharyya IB. Rhinocerebral zygomycosis: an increasingly frequent challenge: update and favorable outcomes in two cases. Oral Surg Oral Med Oral Pathol Oral Radiol Endod. 2007 Nov;104(5):e28-34. doi: 10.1016/j.tripleo.2007.06.014. PMID: 17964469. Link <https://pubmed.ncbi.nlm.nih.gov/17964469/>
1011. Ergene U, Ozbalci D, Baykal B, Metin Ciris I, Yariktas M. Aplastic anemia, mucormycosis and aspergillosis in infectious mononucleosis: success is possible. Transfus Apher Sci. 2007 Oct;37(2):125-9. doi: 10.1016/j.transci.2007.03.016. Epub 2007 Oct 23. PMID: 17959418. Link <https://pubmed.ncbi.nlm.nih.gov/17959418/>
1012. Carton S, Garson S, Benhaim T, Havet E, Massy S, Mertl P, Robbe M. Mucormycose cutanée primaire: à propos d'un cas [Primary cutaneous mucormycosis: a case report]. Ann Chir Plast Esthet. 2008 Aug;53(4):368-71. French. doi: 10.1016/j.anplas.2007.06.005. Epub 2007 Oct 23. PMID: 17959296. Link <https://pubmed.ncbi.nlm.nih.gov/17959296/>
1013. Lassalle S, Butori C, Hofman V, Gari-Toussaint M, Mouroux J, Hofman P. Pneumopathie à Cunninghamella bertholletiae compliquant une leucémie aigüe lymphoblastique [Pneumonia caused by Cunninghamella bertholletiae in a patient with acute lymphoblastic leukemia]. Ann Pathol. 2007 Apr;27(2):141-4. French. doi: 10.1016/s0242-6498(07)91302-4. PMID: 17909475. Link <https://pubmed.ncbi.nlm.nih.gov/17909475/>
1014. Auluck A. Maxillary necrosis by mucormycosis. a case report and literature review. Med Oral Patol Oral Cir Bucal. 2007 Sep 1;12(5):E360-4. PMID: 17767099. Link <https://pubmed.ncbi.nlm.nih.gov/17767099/>
1015. Bengel D, Susa M, Schreiber H, Ludolph AC, Tumani H. Early diagnosis of rhinocerebral mucormycosis by cerebrospinal fluid analysis and determination of 16s rRNA gene sequence. Eur J Neurol. 2007 Sep;14(9):1067-70. doi: 10.1111/j.1468-1331.2007.01878.x. PMID: 17718704. Link <https://pubmed.ncbi.nlm.nih.gov/17718704/>
1016. Metellus P, Laghmari M, Fuentes S, Eusebio A, Adetchessi T, Ranque S, Bouvier C, Dufour H, Grisoli F. Successful treatment of a giant isolated cerebral mucormycotic (zygomycotic) abscess using endoscopic debridement: case report and therapeutic considerations. Surg Neurol. 2008 May;69(5):510-5; discussion 515. doi: 10.1016/j.surneu.2007.02.035. Epub 2007 Aug 17. PMID: 17707491. Link <https://pubmed.ncbi.nlm.nih.gov/17707491/>
1017. McGuire FR, Grinnan DC, Robbins M. Mucormycosis of the bronchial anastomosis: a case of successful medical treatment and historic review. J Heart Lung Transplant. 2007 Aug;26(8):857-61. doi: 10.1016/j.healun.2007.05.010. PMID: 17692792. Link <https://pubmed.ncbi.nlm.nih.gov/17692792/>
1018. Tehmeena W, Hussain W, Zargar HR, Sheikh AR, Iqbal S. Primary cutaneous mucormycosis in an immunocompetent host. Mycopathologia. 2007 Oct;164(4):197-9. doi: 10.1007/s11046-007-9041-8. Epub 2007 Aug 4. PMID: 17676384. Link <https://pubmed.ncbi.nlm.nih.gov/17676384/>
1019. Margo CE, Linden C, Strickland-Marmol LB, Denietolis AL, McCaffrey JC, Kirk N. Rhinocerebral mucormycosis with perineural spread. Ophthalmic Plast Reconstr Surg. 2007 Jul-Aug;23(4):326-7. doi: 10.1097/IOP.0b013e318070855b. PMID: 17667114. Link <https://pubmed.ncbi.nlm.nih.gov/17667114/>
1020. Aslani J, Eizadi M, Kardavani B, Khoddami-Vishteh HR, Nemati E, Hoseini SM, Einollahi B. Mucormycosis after kidney transplantations: report of seven cases. Scand J Infect Dis. 2007;39(8):703-6. doi: 10.1080/00365540701299590. PMID: 17654347. Link <https://pubmed.ncbi.nlm.nih.gov/17654347/>
1021. Goyal A, Tyagi I, Syal R, Marak RS, Singh J. Apophysomyces elegans causing acute otogenic cervicofacial zygomycosis involving salivary glands. Med Mycol. 2007 Aug;45(5):457-61. doi: 10.1080/13693780701320600. PMID: 17654273. Link <https://pubmed.ncbi.nlm.nih.gov/17654273/>
1022. Uçkay I, Chalandon Y, Sartoretti P, Rohner P, Berney T, Hadaya K, van Delden C. Invasive zygomycosis in transplant recipients. Clin Transplant. 2007 Jul-Aug;21(4):577-82. doi: 10.1111/j.1399-0012.2007.00684.x. PMID: 17645724. Link <https://pubmed.ncbi.nlm.nih.gov/17645724/>
1023. Dogan MC, Leblebisatan G, Haytac MC, Antmen B, Surmegozler O. Oral mucormycosis in children with leukemia: report of 2 cases. Quintessence Int. 2007 Jun;38(6):515-20. PMID: 17625636. Link <https://pubmed.ncbi.nlm.nih.gov/17625636/>
1024. Kok J, Gilroy N, Halliday C, Lee OC, Novakovic D, Kevin P, Chen S. Early use of posaconazole in the successful treatment of rhino-orbital mucormycosis caused by Rhizopus oryzae. J Infect. 2007 Sep;55(3):e33-6. doi: 10.1016/j.jinf.2007.05.178. Epub 2007 Jul 10. PMID: 17624436. Link <https://pubmed.ncbi.nlm.nih.gov/17624436/>
1025. Chacko B, David VG, Tamilarasi V, Deepti AN, John GT. Pulmonary mucormycosis in a nondiabetic renal allograft recipient successfully managed by medical therapy alone. Transplantation. 2007 Jun 27;83(12):1656-7. doi: 10.1097/01.tp.0000266581.35950.15. PMID: 17589353. Link <https://pubmed.ncbi.nlm.nih.gov/17589353/>
1026. Pradhan A, Gadela S, Kumar RS, Kalghatghi A, Pradhan S. To bite the bullet of early graft nephrectomy: a case report. Transplant Proc. 2007 Jun;39(5):1664-5. doi: 10.1016/j.transproceed.2007.01.092. PMID: 17580213. Link <https://pubmed.ncbi.nlm.nih.gov/17580213/>
1027. Shindo M, Sato K, Jimbo J, Hosoki T, Ikuta K, Sano A, Nishimura K, Torimoto Y, Kohgo Y. [Breakthrough pulmonary mucormycosis during voriconazole treatment after reduced-intensity cord blood transplantation for a patient with acute myeloid leukemia]. Rinsho Ketsueki. 2007 May;48(5):412-7. Japanese. PMID: 17571588. Link <https://pubmed.ncbi.nlm.nih.gov/17571588/>
1028. Gelston CD, Durairaj VD, Simoes EA. Rhino-orbital mucormycosis causing cavernous sinus and internal carotid thrombosis treated with posaconazole. Arch Ophthalmol. 2007 Jun;125(6):848-9. doi: 10.1001/archopht.125.6.848. PMID: 17563004. Link <https://pubmed.ncbi.nlm.nih.gov/17563004/>
1029. Mohadjer Y, Smith ME, Akduman L. Mucormycosis endophthalmitis after cataract surgery. Ocul Immunol Inflamm. 2007 Mar-Apr;15(2):117-20. doi: 10.1080/09273940601186974. PMID: 17558837. Link <https://pubmed.ncbi.nlm.nih.gov/17558837/>
1030. Cantatore-Francis JL, Shin HT, Heilman E, Glick SA. Primary cutaneous zygomycosis in two immunocompromised children. Pediatr Dermatol. 2007 May-Jun;24(3):257-62. doi: 10.1111/j.1525-1470.2007.00398.x. PMID: 17542876. Link <https://pubmed.ncbi.nlm.nih.gov/17542876/>
1031. Sheibanifar M, Guerin AL, Clement L, Champigneulle J, Mainard L, Mandry D, Bordigoni P, Claudon M. Imagerie des formes viscérales graves de la mucormycose chez 5 patients immunodéprimés [Imaging of serious visceral forms of mucormycosis in five immunodepressed patients]. J Radiol. 2007 May;88(5 Pt 1):677-83. French. doi: 10.1016/s0221-0363(07)89875-1. PMID: 17541361. Link <https://pubmed.ncbi.nlm.nih.gov/17541361/>
1032. Belfiori R, Terenzi A, Marchesini L, Repetto A. Absidia Corymbifera in an immune competent accident victim with multiple abdominal injuries: case report. BMC Infect Dis. 2007 May 25;7:46. doi: 10.1186/1471-2334-7-46. PMID: 17531089; PMCID: PMC1891108. Link <https://pubmed.ncbi.nlm.nih.gov/17531089/>
1033. Kebapci N, Efe B, Alataş F, Açikalin M, Kiraz N. Pulmonary multinodular mucormycosis in type 1 diabetic patient with diabetic ketoacidosis. J Endocrinol Invest. 2007 Mar;30(3):247-52. doi: 10.1007/BF03347433. PMID: 17505160. Link <https://pubmed.ncbi.nlm.nih.gov/17505160/>
1034. Ozaras R, Yemisen M, Mete B, Mert A, Ozturk R, Tabak F. Acrocyanosis developed with amphotericin B deoxycholate but not with amphotericin B lipid complex. Mycoses. 2007 May;50(3):242. doi: 10.1111/j.1439-0507.2007.01360.x. PMID: 17472626. Link <https://pubmed.ncbi.nlm.nih.gov/17472626/>
1035. Salinas-Lara C, Rembao-Bojórquez D, de la Cruz E, Márquez C, Portocarrero L, Tena-Suck ML. Pituitary apoplexy due to mucormycosis infection in a patient with an ACTH producing pulmonary tumor. J Clin Neurosci. 2008 Jan;15(1):67-70. doi: 10.1016/j.jocn.2006.01.023. Epub 2007 Apr 25. PMID: 17462904. Link <https://pubmed.ncbi.nlm.nih.gov/17462904/>
1036. Ameen M, Arenas R, Martinez-Luna E, Reyes M, Zacarias R. The emergence of mucormycosis as an important opportunistic fungal infection: five cases presenting to a tertiary referral center for mycology. Int J Dermatol. 2007 Apr;46(4):380-4. doi: 10.1111/j.1365-4632.2007.03057.x. PMID: 17442077. Link <https://pubmed.ncbi.nlm.nih.gov/17442077/>
1037. Singh P, Taylor SF, Murali R, Gomes LJ, Kanthan GL, Maloof AJ. Disseminated mucormycosis and orbital ischaemia in combination immunosuppression with a tumour necrosis factor alpha inhibitor. Clin Exp Ophthalmol. 2007 Apr;35(3):275-80. doi: 10.1111/j.1442-9071.2007.01458.x. PMID: 17430516. Link <https://pubmed.ncbi.nlm.nih.gov/17430516/>
1038. Devlin SM, Hu B, Ippoliti A. Mucormycosis presenting as recurrent gastric perforation in a patient with Crohn's disease on glucocorticoid, 6-mercaptopurine, and infliximab therapy. Dig Dis Sci. 2007 Sep;52(9):2078-81. doi: 10.1007/s10620-006-9455-z. Epub 2007 Apr 4. PMID: 17406841. Link <https://pubmed.ncbi.nlm.nih.gov/17406841/>
1039. Mathur S, Karimi A, Mafee MF. Acute optic nerve infarction demonstrated by diffusion-weighted imaging in a case of rhinocerebral mucormycosis. AJNR Am J Neuroradiol. 2007 Mar;28(3):489-90. PMID: 17353318; PMCID: PMC7977816. Link <https://pubmed.ncbi.nlm.nih.gov/17353318/>
1040. Raizman NM, Parisien M, Grafe MW, Gordon RJ, Rosenwasser MP. Mucormycosis of the upper extremity in a patient with alcoholic encephalopathy. J Hand Surg Am. 2007 Mar;32(3):384-8. doi: 10.1016/j.jhsa.2007.01.009. PMID: 17336848. Link <https://pubmed.ncbi.nlm.nih.gov/17336848/>
1041. Barrak HA. Hard palate perforation due to mucormycosis: report of four cases. J Laryngol Otol. 2007 Nov;121(11):1099-102. doi: 10.1017/S0022215107006354. Epub 2007 Feb 26. PMID: 17319990. Link <https://pubmed.ncbi.nlm.nih.gov/17319990/>
1042. Virk RS, Arora P. Chronic sinonasal aspergillosis with associated mucormycosis. Ear Nose Throat J. 2007 Jan;86(1):22. PMID: 17315828. Link <https://pubmed.ncbi.nlm.nih.gov/17315828/>
1043. Romano C, Ghilardi A, Massai L, Capecchi PL, Miraccco C, Fimiani M. Primary subcutaneous zygomycosis due to Rhizopus oryzae in a 71-year-old man with normal immune status. Mycoses. 2007 Jan;50(1):82-4. doi: 10.1111/j.1439-0507.2006.01314.x. PMID: 17302755. Link <https://pubmed.ncbi.nlm.nih.gov/17302755/>
1044. Samant JS, Namgoong SH, Parveen T, Katner HP. Cytomegalovirus vasculitis and mucormycosis coinfection in late-stage HIV/AIDS. Am J Med Sci. 2007 Feb;333(2):122-4. doi: 10.1097/00000441-200702000-00011. PMID: 17301593. Link https://pubmed.ncbi.nlm.nih.gov/17301593/
1045. Abbas Z, Jafri W, Rasool S, Abid S, Hameed I. Mucormycosis in patients with complicated cirrhosis. Singapore Med J. 2007 Jan;48(1):69-73. PMID: 17245519. Link <https://pubmed.ncbi.nlm.nih.gov/17245519/>
1046. Safaya A, Batra K, Capoor M. A case of skull base mucormycosis with osteomyelitis secondary to temporal bone squamous cell carcinoma. Ear Nose Throat J. 2006 Dec;85(12):822-4. PMID: 17240707. Link <https://pubmed.ncbi.nlm.nih.gov/17240707/>
1047. Jayachandran S, Krithika C. Mucormycosis presenting as palatal perforation. Indian J Dent Res. 2006 Jul-Sep;17(3):139-42. doi: 10.4103/0970-9290.29873. PMID: 17176832. Link <https://pubmed.ncbi.nlm.nih.gov/17176832/>
1048. Munir N, Jones NS. Rhinocerebral mucormycosis with orbital and intracranial extension: a case report and review of optimum management. J Laryngol Otol. 2007 Feb;121(2):192-5. doi: 10.1017/S0022215106003409. Epub 2006 Nov 30. PMID: 17134533. Link <https://pubmed.ncbi.nlm.nih.gov/17134533/>
1049. Wu VC, Wang R, Lai TS, Wu KD. Deferoxamine-related fatal nasal-orbital-cerebral mucormycosis. Kidney Int. 2006 Dec;70(11):1888. doi: 10.1038/sj.ki.5001736. PMID: 17130818. Link <https://pubmed.ncbi.nlm.nih.gov/17130818/>
1050. Grant JM, St-Germain G, McDonald JC. Successful treatment of invasive Rhizopus infection in a child with thalassemia. Med Mycol. 2006 Dec;44(8):771-5. doi: 10.1080/13693780600930186. PMID: 17127635. Link <https://pubmed.ncbi.nlm.nih.gov/17127635/>
1051. Iwen PC, Sigler L, Noel RK, Freifeld AG. Mucor circinelloides was identified by molecular methods as a cause of primary cutaneous zygomycosis. J Clin Microbiol. 2007 Feb;45(2):636-40. doi: 10.1128/JCM.01907-06. Epub 2006 Nov 22. PMID: 17122018; PMCID: PMC1829032. Link <https://pubmed.ncbi.nlm.nih.gov/17122018/>
1052. Prasad N, Ram R, Satti Reddy V, Dakshinamurty KV. Non-fatal gastric mucormycosis in a renal transplant patient and review of the literature. Transpl Infect Dis. 2006 Dec;8(4):237-41. doi: 10.1111/j.1399-3062.2006.00142.x. PMID: 17116140. Link <https://pubmed.ncbi.nlm.nih.gov/17116140/>
1053. Tiong WH, Ismael T, McCann J. Post-traumatic and post-surgical Absidia corymbifera infection in a young, healthy man. J Plast Reconstr Aesthet Surg. 2006;59(12):1367-71. doi: 10.1016/j.bjps.2006.03.053. Epub 2006 Jun 5. PMID: 17113521. Link <https://pubmed.ncbi.nlm.nih.gov/17113521/>
1054. Schütz P, Behbehani JH, Khan ZU, Ahmad S, Kazem MA, Dhar R, Eskaf W, Hamed HH, Cunningham LL Jr. Fatal rhino-orbito-cerebral zygomycosis caused by Apophysomyces elegans in a healthy patient. J Oral Maxillofac Surg. 2006 Dec;64(12):1795-802. doi: 10.1016/j.joms.2006.05.010. PMID: 17113448. Link <https://pubmed.ncbi.nlm.nih.gov/17113448/>
1055. Chacko B, Ananthakrishna R, Vasuki Z, Thambu SD, Nair S. Mucormycosis isolated from perilymphatic tissue: an unusual presentation. J Postgrad Med. 2006 Oct-Dec;52(4):294-5. PMID: 17102550. Link <https://pubmed.ncbi.nlm.nih.gov/17102550/>
1056. Park SK, Jung H, Kang MS. Localized bilateral paranasal mucormycosis: a case in an immunocompetent patient. Acta Otolaryngol. 2006 Dec;126(12):1339-41. doi: 10.1080/00016480500316852. PMID: 17101598. Link <https://pubmed.ncbi.nlm.nih.gov/17101598/>
1057. Deja M, Wolf S, Weber-Carstens S, Lehmann TN, Adler A, Ruhnke M, Tintelnot K. Gastrointestinal zygomycosis caused by Mucor indicus in a patient with acute traumatic brain injury. Med Mycol. 2006 Nov;44(7):683-7. doi: 10.1080/13693780600803888. PMID: 17071566. Link <https://pubmed.ncbi.nlm.nih.gov/17071566/> Link <https://pubmed.ncbi.nlm.nih.gov/17071566/>
1058. Saito T, Ikezoe T, Daibata M, Takeuchi T, Ohtsuki Y, Taguchi H, Miyoshi I. Disseminated mucormycosis (zygomycosis) in acute myeloid leukemia. Intern Med. 2006;45(18):1073-4. doi: 10.2169/internalmedicine.45.1810. Epub 2006 Oct 16. PMID: 17043382. Link <https://pubmed.ncbi.nlm.nih.gov/17043382/>
1059. Vega W, Orellana M, Zaror L, Gené J, Guarro J. Saksenaea vasiformis infections: case report and literature review. Mycopathologia. 2006 Oct;162(4):289-94. doi: 10.1007/s11046-006-0061-6. PMID: 17039275. Link <https://pubmed.ncbi.nlm.nih.gov/17039275/>
1060. Zeddini A, Mekni A, Ferchichi L, Bel Haj Salah M, Kharrat S, Zainine R, Bellil K, Bellil S, Haouet S, Kchir N, Zitouna M. Mucormycose rhinocérébrale: à propos d'un cas [Rhinocerebral mucormycosis: A case report]. Med Mal Infect. 2006 Oct;36(10):517-9. French. doi: 10.1016/j.medmal.2006.05.017. Epub 2006 Oct 5. PMID: 17027215. Link <https://pubmed.ncbi.nlm.nih.gov/17027215/>
1061. Reed C, Ibrahim A, Edwards JE Jr, Walot I, Spellberg B. Deferasirox, an iron-chelating agent, as salvage therapy for rhinocerebral mucormycosis. Antimicrob Agents Chemother. 2006 Nov;50(11):3968-9. doi: 10.1128/AAC.01065-06. Epub 2006 Sep 25. PMID: 17000743; PMCID: PMC1635215. Link <https://pubmed.ncbi.nlm.nih.gov/17000743/>
1062. de Oliveira-Neto MP, Da Silva M, Fialho Monteiro PC, Lazera M, de Almeida Paes R, Novellino AB, Cuzzi T. Cutaneous mucormycosis in a young, immunocompetent girl. Med Mycol. 2006 Sep;44(6):567-70. doi: 10.1080/13693780600622411. Erratum in: Med Mycol. 2006 Dec;44(8):793. PMID: 16966176. Link <https://pubmed.ncbi.nlm.nih.gov/16966176/>
1063. Kantarcioğlu AS, Yücel A, Nagao K, Sato T, Inci E, Ogreden S, Kaytaz A, Alan S, Bozdağ Z, Edali N, Sar M, Kepil N, Oz B, Altas K. A Rhizopus oryzae strain isolated from resected bone and soft tissue specimens from a sinonasal and palatal mucormycosis case. Report of a case and in vitro experiments of yeastlike cell development. Med Mycol. 2006 Sep;44(6):515-21. doi: 10.1080/13693780600764973. PMID: 16966168. Link <https://pubmed.ncbi.nlm.nih.gov/16966168/>
1064. Rickerts V, Atta J, Herrmann S, Jacobi V, Lambrecht E, Bialek R, Just-Nübling G. Successful treatment of disseminated mucormycosis with a combination of liposomal amphotericin B and posaconazole in a patient with acute myeloid leukaemia. Mycoses. 2006;49 Suppl 1:27-30. doi: 10.1111/j.1439-0507.2006.01299.x. PMID: 16961579. Link <https://pubmed.ncbi.nlm.nih.gov/16961579/>
1065. Horger M, Hebart H, Schimmel H, Vogel M, Brodoefel H, Oechsle K, Hahn U, Mittelbronn M, Bethge W, Claussen CD. Disseminated mucormycosis in haematological patients: CT and MRI findings with pathological correlation. Br J Radiol. 2006 Sep;79(945):e88-95. doi: 10.1259/bjr/16038097. PMID: 16940368. Link <https://pubmed.ncbi.nlm.nih.gov/16940368/>
1066. Song KY, Kang WK, Park CW, Choi YJ, Rha SE, Park CH. Mucormycosis resulting in gastric perforation in a patient with acute myelogenous leukemia: report of a case. Surg Today. 2006;36(9):831-4. doi: 10.1007/s00595-006-3246-1. PMID: 16937290. Link <https://pubmed.ncbi.nlm.nih.gov/16937290/>
1067. Oh WS, Roumanas E. Dental implant-assisted prosthetic rehabilitation of a patient with a bilateral maxillectomy defect secondary to mucormycosis. J Prosthet Dent. 2006 Aug;96(2):88-95. doi: 10.1016/j.prosdent.2006.05.004. PMID: 16911884. Link <https://pubmed.ncbi.nlm.nih.gov/16911884/>
1068. Yu J, Li RY. Primary renal zygomycosis due to Rhizopus oryzae. Med Mycol. 2006 Aug;44(5):461-6. doi: 10.1080/13693780500338951. PMID: 16882613. Link <https://pubmed.ncbi.nlm.nih.gov/16882613/>
1069. De Decker K, Van Poucke S, Wojciechowski M, Ieven M, Colpaert C, Vogelaers D, Jorens PG. Successful use of posaconazole in a pediatric case of fungal necrotizing fasciitis. Pediatr Crit Care Med. 2006 Sep;7(5):482-5. doi: 10.1097/01.PCC.0000235255.68759.05. PMID: 16878050. Link <https://pubmed.ncbi.nlm.nih.gov/16878050/>
1070. Piccin A, Russell JD, Fleming P, Butler K, McDermott M, Smith OP, O'Marcaigh A. Invasive rhino-maxillary mucormycosis diagnosed before HSCT. Pediatr Blood Cancer. 2008 Feb;50(2):393-5. doi: 10.1002/pbc.20994. PMID: 16874768. Link
1071. Becker BC, Schuster FR, Ganster B, Seidl HP, Schmid I. Cutaneous mucormycosis in an immunocompromised patient. Lancet Infect Dis. 2006 Aug;6(8):536. doi: 10.1016/S1473-3099(06)70554-0. PMID: 16870532. Link <https://pubmed.ncbi.nlm.nih.gov/16870532/>
1072. Deboni MC, Pozzani VR, Lisboa T, Hiraki K, Viplich R, Naclério-Homem MG. Mucormycosis in an immunocompetent patient: follow-up of 1 year after treatment. Acta Otolaryngol. 2006 Sep;126(9):993-6. doi: 10.1080/00016480500534439. PMID: 16864500. Link <https://pubmed.ncbi.nlm.nih.gov/16864500/>
1073. Economopoulos N, Kelekis D, Papadopoulos A, Kontopoulou C, Brountzos EN. Subclavian artery occlusion and pseudoaneurysm caused by lung apex mucormycosis: successful treatment with transcatheter embolization. Cardiovasc Intervent Radiol. 2007 Jan-Feb;30(1):143-5. doi: 10.1007/s00270-005-0328-1. PMID: 16832593. Link <https://pubmed.ncbi.nlm.nih.gov/16832593/>
1074. Sharma R, Shivanand G, Kumar R, Prem S, Kandpal H, Das CJ, Sharma MC. Isolated renal mucormycosis: an unusual cause of acute renal infarction in a boy with aplastic anaemia. Br J Radiol. 2006 Jul;79(943):e19-21. doi: 10.1259/bjr/17821080. PMID: 16823048. Link <https://pubmed.ncbi.nlm.nih.gov/16823048/>
1075. Rutar T, Cockerham KP. Periorbital zygomycosis (mucormycosis) treated with posaconazole. Am J Ophthalmol. 2006 Jul;142(1):187-188. doi: 10.1016/j.ajo.2006.02.053. PMID: 16815283. Link <https://pubmed.ncbi.nlm.nih.gov/16815283/>
1076. Lai CC, Liaw SJ, Hsiao YC, Chiu YS, Laio WY, Lee LN, Hsueh PR. Empyema thoracis due to Rhizopus oryzae in an allogenic bone marrow transplant recipient. Med Mycol. 2006 Feb;44(1):75-8. doi: 10.1080/13693780500148392. PMID: 16805096. Link <https://pubmed.ncbi.nlm.nih.gov/16805096/>
1077. Vetrone G, Grazi GL, Ercolani G, Ravaioli M, Faenza S, Enrico B, Tumietto F, Pinna AD. Successful treatment of rhinomaxillary form of mucormycosis infection after liver transplantation: a case report. Transplant Proc. 2006 Jun;38(5):1445-7. doi: 10.1016/j.transproceed.2006.02.102. PMID: 16797328. Link <https://pubmed.ncbi.nlm.nih.gov/16797328/>
1078. Yang G, Lin J, Dong X, Wang H, Liu X, An L. A case of rhinocerebral zygomycosis caused by Rhizopus arrhizus. Int J Dermatol. 2006 Jun;45(6):780-3. doi: 10.1111/j.1365-4632.2006.02710.x. PMID: 16796654. Link <https://pubmed.ncbi.nlm.nih.gov/16796654/>
1079. Pellacchia V, Terenzi V, Moricca LM, Buonaccorsi S, Indrizzi E, Fini G. Brain abscess by mycotic and bacterial infection in a diabetic patient: clinical report and review of literature. J Craniofac Surg. 2006 May;17(3):578-84. doi: 10.1097/00001665-200605000-00034. PMID: 16770203. Link <https://pubmed.ncbi.nlm.nih.gov/16770203/>
1080. Sakorafas GH, Tsolakides G, Grigoriades K, Bakoyiannis CN, Peros G. Colonic mucormycosis: an exceptionally rare cause of massive lower gastrointestinal bleeding. Dig Liver Dis. 2006 Aug;38(8):616-7. doi: 10.1016/j.dld.2006.03.018. Epub 2006 Jun 5. PMID: 16750662. Link <https://pubmed.ncbi.nlm.nih.gov/16750662/>
1081. Ferchichi L, Chadli-Debbiche A, Koubâa W, Khayat O, Labbène N, Ben Gamra O, Khedim A, Ben Ayed M. la mucormycose rhinocérébrale chez le diabétique. A propos de quatre observations [Rhinocerebral mucormycosis in four diabetics]. J Mal Vasc. 2006 May;31(2):85-7. French. doi: 10.1016/s0398-0499(06)76524-6. PMID: 16733440. Link <https://pubmed.ncbi.nlm.nih.gov/16733440/>
1082. Saşmaz I, Leblebisatan G, Antmen B, Binokay F, Tunali N, Kilinç Y. Cardiac mucormycosis in a child with severe aplastic anemia: a case report. Pediatr Hematol Oncol. 2006 Jul-Aug;23(5):433-7. doi: 10.1080/08880010600692138. PMID: 16728364. Link <https://pubmed.ncbi.nlm.nih.gov/16728364/>
1083. Hu WT, Leavitt JA, Moore EJ, Noseworthy JH. MRI findings of rapidly progressive ophthalmoplegia and blindness in mucormycosis. Neurology. 2006 May 23;66(10):E40. doi: 10.1212/01.wnl.0000204231.85308.7e. PMID: 16717200. Link <https://pubmed.ncbi.nlm.nih.gov/16717200/>
1084. Jeevanan J, Gendeh BS, Faridah HA, Vikneswaran T. Rhino-orbito-cerebral mucormycosis: a treatment dilemma. Med J Malaysia. 2006 Mar;61(1):106-8. PMID: 16708746. Link <https://pubmed.ncbi.nlm.nih.gov/16708746/>
1085. Thapar VK, Deshpande A, Jain VK, Bhowate P, Madiwale C. Isolated breast mucormycosis. J Postgrad Med. 2006 Apr-Jun;52(2):134-5. PMID: 16679679. Link <https://pubmed.ncbi.nlm.nih.gov/16679679/>
1086. Alfano C, Chiummariello S, Dessy LA, Bistoni G, Scuderi N. Combined mucormycosis and Aspergillosis of the rhinocerebral region. In Vivo. 2006 Mar-Apr;20(2):311-5. PMID: 16634536. Link <https://pubmed.ncbi.nlm.nih.gov/16634536/>
1087. Hayes D Jr. Nosocomial pulmonary Rhizopus diagnosed by bronchoalveolar lavage with cytology in a child with acute lymphoblastic leukemia. Pediatr Hematol Oncol. 2006 Jun;23(4):323-7. doi: 10.1080/08880010600629742. PMID: 16621774. Link <https://pubmed.ncbi.nlm.nih.gov/16621774/>
1088. Ayadi-Kaddour A, Braham E, Ismail O, Saïji E, Bourguiba M, Zaïmi M, Zoghlami F, El Mezni F. Mucormycose pulmonaire. A propos de deux cas [Pulmonary mucormycosis. Two cases]. Rev Pneumol Clin. 2006 Feb;62(1):37-42. French. doi: 10.1016/s0761-8417(06)75412-8. PMID: 16604040. Link <https://pubmed.ncbi.nlm.nih.gov/16604040/>
1089. Liapis CD, Petrikkos GL, Paraskevas KI, Skiada A, Nikolaou AC, Tzortzis G, Kostakis AG. External Iliac artery stent mucormycosis in a renal transplant patient. Ann Vasc Surg. 2006 Mar;20(2):253-7. doi: 10.1007/s10016-006-9007-1. Epub 2006 Mar 21. PMID: 16550480. Link <https://pubmed.ncbi.nlm.nih.gov/16550480/>
1090. Fogarty C, Regennitter F, Viozzi CF. Invasive fungal infection of the maxilla following dental extractions in a patient with chronic obstructive pulmonary disease. J Can Dent Assoc. 2006 Mar;72(2):149-52. PMID: 16545177. Link <https://pubmed.ncbi.nlm.nih.gov/16545177/>
1091. Grandin W, Dessieux T, Hounfodji P, Viquesnel G, Ouchikhe A, Gérard JL. Mucormycose pulmonaire chez un patient polytraumatisé [Pulmonary mucormycosis in a multiple-trauma patient]. Ann Fr Anesth Reanim. 2006 May;25(5):521-4. French. doi: 10.1016/j.annfar.2005.12.007. Epub 2006 Mar 10. PMID: 16531002. Link <https://pubmed.ncbi.nlm.nih.gov/16531002/>
1092. Shah A, Lagvankar S, Shah A. Cutaneous mucormycosis in children. Indian Pediatr. 2006 Feb;43(2):167-70. PMID: 16528115. Link <https://pubmed.ncbi.nlm.nih.gov/16528115/>
1093. Liang KP, Tleyjeh IM, Wilson WR, Roberts GD, Temesgen Z. Rhino-orbitocerebral mucormycosis caused by Apophysomyces elegans. J Clin Microbiol. 2006 Mar;44(3):892-8. doi: 10.1128/JCM.44.3.892-898.2006. PMID: 16517873; PMCID: PMC1393113. Link <https://pubmed.ncbi.nlm.nih.gov/16517873/>
1094. Al-Ajam MR, Bizri AR, Mokhbat J, Weedon J, Lutwick L. Mucormycosis in the Eastern Mediterranean: a seasonal disease. Epidemiol Infect. 2006 Apr;134(2):341-6. doi: 10.1017/S0950268805004930. PMID: 16490139; PMCID: PMC2870385. Link <https://pubmed.ncbi.nlm.nih.gov/16490139/>
1095. Monecke S, Hochauf K, Gottschlich B, Ehricht R. A case of peritonitis caused by Rhizopus microsporus. Mycoses. 2006 Mar;49(2):139-42. doi: 10.1111/j.1439-0507.2006.01190.x. PMID: 16466449. Link <https://pubmed.ncbi.nlm.nih.gov/16466449/>
1096. Tricot S, Gastine T, Sendid B, Wurtz A, de Botton S, Alfandari S. Zygomycose pulmonaire chez un patient traité pour une aspergillose invasive possible [Pulmonary zygomycosis in a patient treated for invasive aspergillosis]. Med Mal Infect. 2006 Feb;36(2):118-21. French. doi: 10.1016/j.medmal.2005.12.003. Epub 2006 Feb 3. PMID: 16458464. Link <https://pubmed.ncbi.nlm.nih.gov/16458464/>
1097. Kotoulas C, Psathakis K, Tsintiris K, Sampaziotis D, Karnesis L, Laoutidis G. Pulmonary mucormycosis presenting as Horner's syndrome. Asian Cardiovasc Thorac Ann. 2006 Feb;14(1):86-7. doi: 10.1177/021849230601400123. PMID: 16432130. Link <https://pubmed.ncbi.nlm.nih.gov/16432130/>
1098. O'Neill BM, Alessi AS, George EB, Piro J. Disseminated rhinocerebral mucormycosis: a case report and review of the literature. J Oral Maxillofac Surg. 2006 Feb;64(2):326-33. doi: 10.1016/j.joms.2005.10.017. PMID: 16413907. Link <https://pubmed.ncbi.nlm.nih.gov/16413907/>
1099. Jayasuriya NS, Tilakaratne WM, Amaratunga EA, Ekanayake MK. An unusual presentation of rhinofacial zygomycosis due to Cunninghamella sp. in an immunocompetent patient: a case report and literature review. Oral Dis. 2006 Jan;12(1):67-9. doi: 10.1111/j.1601-0825.2005.01154.x. PMID: 16390472. Link <https://pubmed.ncbi.nlm.nih.gov/16390472/>
1100. Simmons JH, Zeitler PS, Fenton LZ, Abzug MJ, Fiallo-Scharer RV, Klingensmith GJ. Rhinocerebral mucormycosis complicated by internal carotid artery thrombosis in a pediatric patient with type 1 diabetes mellitus: a case report and review of the literature. Pediatr Diabetes. 2005 Dec;6(4):234-8. doi: 10.1111/j.1399-543X.2005.00118.x. PMID: 16390393. Link <https://pubmed.ncbi.nlm.nih.gov/16390393/>
1101. Passos XS, Sales WS, Maciel PJ, Costa CR, Ferreira DM, do Silva MR. Nosocomial invasive infection caused by Cunninghamella bertholletiae: case report. Mycopathologia. 2006 Jan;161(1):33-5. doi: 10.1007/s11046-005-0068-4. PMID: 16389482. Link <https://pubmed.ncbi.nlm.nih.gov/16389482/>
1102. Pimentel JD, Dreyer G, Lum GD. Peritonitis due to Cunninghamella bertholletiae in a patient undergoing continuous ambulatory peritoneal dialysis. J Med Microbiol. 2006 Jan;55(Pt 1):115-118. doi: 10.1099/jmm.0.46202-0. PMID: 16388039. Link <https://pubmed.ncbi.nlm.nih.gov/16388039/>
1103. Brugière O, Dauriat G, Mal H, Marrash-Chalha R, Fournier M, Groussard O, Besnard M, Lesèche G, Dupont B. Pulmonary mucormycosis (zygomycosis) in a lung transplant recipient: recovery after posaconazole therapy. Transplantation. 2005 Nov 15;80(9):1361-2. PMID: 16382553. Link <https://pubmed.ncbi.nlm.nih.gov/16382553/>
1104. Chopra H, Dua K, Malhotra V, Gupta RP, Puri H. Invasive fungal sinusitis of isolated sphenoid sinus in immunocompetent subjects. Mycoses. 2006 Jan;49(1):30-6. doi: 10.1111/j.1439-0507.2005.01170.x. PMID: 16367816. Link <https://pubmed.ncbi.nlm.nih.gov/16367816/>
1105. Chen F, Lü G, Kang Y, Ma Z, Lu C, Wang B, Li J, Liu J, Li H. Mucormycosis spondylodiscitis after lumbar disc puncture. Eur Spine J. 2006 Mar;15(3):370-6. doi: 10.1007/s00586-005-1025-0. Epub 2005 Nov 18. PMID: 16328227; PMCID: PMC3489305. Link <https://pubmed.ncbi.nlm.nih.gov/16328227/>
1106. Aboltins CA, Pratt WA, Solano TR. Fungemia secondary to gastrointestinal Mucor indicus infection. Clin Infect Dis. 2006 Jan 1;42(1):154-5. doi: 10.1086/498751. PMID: 16323109. Link <https://pubmed.ncbi.nlm.nih.gov/16323109/>
1107. Chan-Tack KM, Nemoy LL, Perencevich EN. Central venous catheter-associated fungemia secondary to mucormycosis. Scand J Infect Dis. 2005;37(11-12):925-7. doi: 10.1080/00365540500262542. PMID: 16308234. Link <https://pubmed.ncbi.nlm.nih.gov/16308234/>
1108. Schlebusch S, Looke DF. Intraabdominal zygomycosis caused by Syncephalastrum racemosum infection successfully treated with partial surgical debridement and high-dose amphotericin B lipid complex. J Clin Microbiol. 2005 Nov;43(11):5825-7. doi: 10.1128/JCM.43.11.5825-5827.2005. PMID: 16272533; PMCID: PMC1287817. Link <https://pubmed.ncbi.nlm.nih.gov/16272533/>
1109. Iwen PC, Freifeld AG, Sigler L, Tarantolo SR. Molecular identification of Rhizomucor pusillus as a cause of sinus-orbital zygomycosis in a patient with acute myelogenous leukemia. J Clin Microbiol. 2005 Nov;43(11):5819-21. doi: 10.1128/JCM.43.11.5819-5821.2005. PMID: 16272531; PMCID: PMC1287840. Link <https://pubmed.ncbi.nlm.nih.gov/16272531/>
1110. Verma A, Brozman B, Petito CK. Isolated cerebral mucormycosis: report of a case and review of the literature. J Neurol Sci. 2006 Jan 15;240(1-2):65-9. doi: 10.1016/j.jns.2005.09.010. Epub 2005 Nov 2. PMID: 16269155. Link <https://pubmed.ncbi.nlm.nih.gov/16269155/>
1111. Chandu A, MacIsaac RJ, MacGregor DP, Campbell MC, Wilson MJ, Bach LA. A case of mucormycosis limited to the parotid gland. Head Neck. 2005 Dec;27(12):1108-11. doi: 10.1002/hed.20287. PMID: 16240333. Link <https://pubmed.ncbi.nlm.nih.gov/16240333/>
1112. Garbino J, Uçkay I, Amini K, Puppo M, Richter M, Lew D. Absidia posttraumatic infection: successful treatment with posaconazole. J Infect. 2005 Oct;51(3):e135-8. doi: 10.1016/j.jinf.2004.11.002. Epub 2004 Dec 2. PMID: 16230192. Link <https://pubmed.ncbi.nlm.nih.gov/16230192/>
1113. Vichova Z, Beuret P, Boyer M, Chanoz J. Mucormycose pulmonaire fatale chez une patiente diabétique [Fatal pulmonary mucormycosis in a diabetic patient]. Ann Fr Anesth Reanim. 2006 Jan;25(1):40-2. French. doi: 10.1016/j.annfar.2005.07.077. Epub 2005 Oct 12. PMID: 16226426. Link <https://pubmed.ncbi.nlm.nih.gov/16226426/>
1114. Alkhunaizi AM, Amir AA, Al-Tawfiq JA. Invasive fungal infections in living unrelated renal transplantation. Transplant Proc. 2005 Sep;37(7):3034-7. doi: 10.1016/j.transproceed.2005.07.029. PMID: 16213296. Link <https://pubmed.ncbi.nlm.nih.gov/16213296/>
1115. El Deeb Y, Al Soub H, Almaslamani M, Al Khuwaiter J, Taj-Aldeen SJ. Post-traumatic cutaneous mucormycosis in an immunocompetent patient. Ann Saudi Med. 2005 Jul-Aug;25(4):343-5. doi: 10.5144/0256-4947.2005.343. PMID: 16212131; PMCID: PMC6148005. Link <https://pubmed.ncbi.nlm.nih.gov/16212131/>
1116. Cheema SA, Amin F. Five cases of rhinocerebral mucormycosis. Br J Oral Maxillofac Surg. 2007 Mar;45(2):161-2. doi: 10.1016/j.bjoms.2005.06.021. Epub 2005 Sep 12. PMID: 16162369. Link <https://pubmed.ncbi.nlm.nih.gov/16162369/>
1117. Jouhet C, Mohty AM, Taseï AM, Charbonnier A, Ferrando M, Grob JJ, Richard MA. Hypodermite granulomateuse révélatrice d'une mucormycose chez une malade immunodéprimée [Granulomatous hypodermitis revealing a cutaneous mucormycosis in an immunodepressed patient]. Ann Dermatol Venereol. 2005 Jun-Jul;132(6-7 Pt 1):536-9. French. doi: 10.1016/s0151-9638(05)79334-0. PMID: 16142101. Link <https://pubmed.ncbi.nlm.nih.gov/16142101/>
1118. Tidwell J, Higuera S, Hollier LH Jr. Facial reconstruction after mucormycosis in an immunocompetent host. Am J Otolaryngol. 2005 Sep-Oct;26(5):333-6. doi: 10.1016/j.amjoto.2005.01.017. PMID: 16137533. Link <https://pubmed.ncbi.nlm.nih.gov/16137533/>
1119. Brugière O, Dauriat G, Mal H, Marrash-Chalha R, Fournier M, Groussard O, Besnard M, Lesèche G, Dupont B. Pulmonary mucormycosis (zygomycosis) in a lung transplant recipient: recovery after posaconazole therapy. Transplantation. 2005 Aug 27;80(4):544-5. doi: 10.1097/01.tp.0000168343.47569.1c. Erratum in: Transplantation. 2005 Oct 15;80(7):999. Brugière, Olivier [added]; Dauriat, Gaële [added]; Mal, Hervé [added]; Marrash-Chalha, Rolana [added]; Fournier, Michel [added]; Groussard, Odile [added]; Besnard, Mathieu [added]; Lesèche, Guy [added]. Corrected and republished in: Transplantation. 2005 Nov 15;80(9):1361-2. PMID: 16123738. Link <https://pubmed.ncbi.nlm.nih.gov/16123738/>
1120. Sivakumar S, Mathews MS, George B. Cunninghamella pneumonia in postbone marrow transplant patient: first case report from India. Mycoses. 2005 Sep;48(5):360-2. doi: 10.1111/j.1439-0507.2005.01139.x. PMID: 16115110. Link <https://pubmed.ncbi.nlm.nih.gov/16115110/>
1121. Pérez-Uribe A, Molina de Soschin D, Arenas R, Reyes M. Mucormicosis cutánea primaria en un paciente con virus de la inmunodeficiencia humana [Primary cutaneous mucormycosis. Report of a case in a HIV patient]. Rev Iberoam Micol. 2005 Jun;22(2):118-21. Spanish. doi: 10.1016/s1130-1406(05)70021-8. PMID: 16107172. Link <https://pubmed.ncbi.nlm.nih.gov/16107172/>
1122. Ghafoor T, Majeed A, Muhammad S, Mahmud S, Mahmood A. Rhinocerebral mucormycosis. J Pak Med Assoc. 2004 Dec;54(12):632-4. PMID: 16104493. Link <https://pubmed.ncbi.nlm.nih.gov/16104493/>
1123. Huang SF, Chiou SC. Radiology quiz case 1: rhinocerebral mucormycosis with cavernous sinus thrombosis. Arch Otolaryngol Head Neck Surg. 2005 Aug;131(8):738, 742. doi: 10.1001/archotol.131.8.738. PMID: 16103313. Link <https://pubmed.ncbi.nlm.nih.gov/16103313/>
1124. Fujimoto A, Nagao K, Tanaka K, Yamagami J, Udagawa SI, Sugiura M. The first case of cutaneous mucormycosis caused by Rhizopus azygosporus. Br J Dermatol. 2005 Aug;153(2):428-30. doi: 10.1111/j.1365-2133.2005.06593.x. PMID: 16086761. Link <https://pubmed.ncbi.nlm.nih.gov/16086761/>
1125. Karanth M, Taniere P, Barraclough J, Murray JA. A rare presentation of zygomycosis (mucormycosis) and review of the literature. J Clin Pathol. 2005 Aug;58(8):879-81. doi: 10.1136/jcp.2004.021816. PMID: 16049294; PMCID: PMC1770885. Link <https://pubmed.ncbi.nlm.nih.gov/16049294/>
1126. Stamm B. Mucormycosis of the stomach in a patient with multiple trauma. Histopathology. 2005 Aug;47(2):222-3. doi: 10.1111/j.1365-2559.2005.02080.x. PMID: 16045789. Link <https://pubmed.ncbi.nlm.nih.gov/16045789/>
1127. Trabelsi A, Soua A, Sriha B, Mili AF, Mokni M, Laarif M, Korbi S. Mucormycose et diabète: a propos de trois cas [Mucormycosis and diabetes: three cases reported]. Rev Med Liege. 2005 May-Jun;60(5-6):545-8. French. PMID: 16035325. Link <https://pubmed.ncbi.nlm.nih.gov/16035325/>
1128. Huang JS, Kok SH, Lee JJ, Hsu WY, Chiang CP, Kuo YS. Extensive maxillary sequestration resulting from mucormycosis. Br J Oral Maxillofac Surg. 2005 Dec;43(6):532-4. doi: 10.1016/j.bjoms.2005.05.012. Epub 2005 Jul 15. PMID: 16024140. Link <https://pubmed.ncbi.nlm.nih.gov/16024140/>
1129. Lokeshwar N, Kumar L, Kumari M. Severe bone marrow aplasia following imatinib mesylate in a patient with chronic myelogenous leukemia. Leuk Lymphoma. 2005 May;46(5):781-4. doi: 10.1080/10428190500046778. PMID: 16019519. Link <https://pubmed.ncbi.nlm.nih.gov/16019519/>
1130. Krauze A, Krenke K, Matysiak M, Kulus M. Fatal course of pulmonary Absidia sp. infection in a 4-year-old girl undergoing treatment for acute lymphoblastic leukemia. J Pediatr Hematol Oncol. 2005 Jul;27(7):386-8. doi: 10.1097/01.mph.0000171285.89950.00. PMID: 16012329. Link <https://pubmed.ncbi.nlm.nih.gov/16012329/>
1131. Mekeel KL, Hemming AW, Reed AI, Matsumoto T, Fujita S, Schain DC, Nelson DR, Dixon LR, Fujikawa T. Hepatic mucormycosis in a renal transplant recipient. Transplantation. 2005 Jun 15;79(11):1636. doi: 10.1097/01.tp.0000158715.12772.85. PMID: 15940058. Link <https://pubmed.ncbi.nlm.nih.gov/15940058/>
1132. Kumar V, Aggarwal A, Taneja R, Saha SS, Khazanchi RK, Kler N, Saluja S. Primary cutaneous mucormycosis in a premature neonate and its management by tumescent skin grafting. Br J Plast Surg. 2005 Sep;58(6):852-4. doi: 10.1016/j.bjps.2005.01.005. PMID: 15936002. Link <https://pubmed.ncbi.nlm.nih.gov/15936002/>
1133. Orguc S, Yücetürk AV, Demir MA, Goktan C. Rhinocerebral mucormycosis: perineural spread via the trigeminal nerve. J Clin Neurosci. 2005 May;12(4):484-6. doi: 10.1016/j.jocn.2004.07.015. PMID: 15925791. Link <https://pubmed.ncbi.nlm.nih.gov/15925791/>
1134. Hampson FG, Ridgway EJ, Feeley K, Reilly JT. A fatal case of disseminated zygomycosis associated with the use of blood glucose self-monitoring equipment. J Infect. 2005 Dec;51(5):e269-72. doi: 10.1016/j.jinf.2005.03.010. PMID: 15904967. Link <https://pubmed.ncbi.nlm.nih.gov/15904967/>
1135. Samanta TK, Biswas J, Gopal L, Kumarasamy N, Solomon S. Panophthalmitis due to rhizopus in an AIDS patient: a clinicopathological study. Indian J Ophthalmol. 2001 Mar;49(1):49-51. PMID: 15887716. Link <https://pubmed.ncbi.nlm.nih.gov/15887716/>
1136. Miyamoto H, Hayashi H, Nakajima H. Cutaneous mucormycosis in a patient with acute lymphocytic leukemia. J Dermatol. 2005 Apr;32(4):273-7. doi: 10.1111/j.1346-8138.2005.tb00761.x. PMID: 15863849. Link <https://pubmed.ncbi.nlm.nih.gov/15863849/>
1137. Davari HR, Malekhossini SA, Salahi HA, Bahador A, Saberifirozi M, Geramizadeh B, Lahsaee SM, Khosravi MB, Imanieh MH, Bagheri MH. Outcome of mucormycosis in liver transplantation: four cases and a review of literature. Exp Clin Transplant. 2003 Dec;1(2):147-52. PMID: 15859921. Link <https://pubmed.ncbi.nlm.nih.gov/15859921/>
1138. Lewejohann J, Muhl E, Birth M, Kujath P, Bruch HP. Pulmonale Zygomykose--eine seltene angioinvasive Pilzinfektion [Pulmonary zygomycosis--a rare angioinvasive fungal infection]. Mycoses. 2005;48 Suppl 1:99-107. German. doi: 10.1111/j.1439-0507.2005.01118.x. PMID: 15826297. Link <https://pubmed.ncbi.nlm.nih.gov/15826297/>
1139. Barron MA, Lay M, Madinger NE. Surgery and treatment with high-dose liposomal amphotericin B for eradication of craniofacial zygomycosis in a patient with Hodgkin's disease who had undergone allogeneic hematopoietic stem cell transplantation. J Clin Microbiol. 2005 Apr;43(4):2012-4. doi: 10.1128/JCM.43.4.2012-2014.2005. PMID: 15815047; PMCID: PMC1081393. Link <https://pubmed.ncbi.nlm.nih.gov/15815047/>
1140. Chadli-Chaieb M, Bchir A, Fathallah-Mili A, Ach K, Maaroufi A, Garrouche A, Chaieb L. La mucormycose chez le diabétique [Mucormycosis in the diabetic patient]. Presse Med. 2005 Feb 12;34(3):218-22. French. doi: 10.1016/s0755-4982(05)88251-x. PMID: 15798533. Link <https://pubmed.ncbi.nlm.nih.gov/15798533/>
1141. Peter L, Krolak-Salmon P, Pignat JC, Dardel P, Vighetto A. Une mucormycose rhinocérébrale [Rhinocerebral mucormycosis]. Rev Neurol (Paris). 2005 Feb;161(2):214-7. French. doi: 10.1016/s0035-3787(05)85025-8. PMID: 15798521. Link <https://pubmed.ncbi.nlm.nih.gov/15798521/>
1142. McLintock LA, Gibson BE, Jones BL. Mixed pulmonary fungal infection with Aspergillus fumigatus and Absidia corymbifera in a patient with relapsed acute myeloid leukaemia. Br J Haematol. 2005 Mar;128(6):737. doi: 10.1111/j.1365-2141.2005.05379.x. PMID: 15755275. Link <https://pubmed.ncbi.nlm.nih.gov/15755275/>
1143. Andresen D, Donaldson A, Choo L, Knox A, Klaassen M, Ursic C, Vonthethoff L, Krilis S, Konecny P. Multifocal cutaneous mucormycosis complicating polymicrobial wound infections in a tsunami survivor from Sri Lanka. Lancet. 2005 Mar 5-11;365(9462):876-8. doi: 10.1016/S0140-6736(05)71046-1. Erratum in: Lancet. 2005 Jul 2-8;366(9479):28. PMID: 15752532. Link <https://pubmed.ncbi.nlm.nih.gov/15752532/>
1144. Chen L, Xiao Y, Wang X. Successful treatment of mucormycosis in the pulmonary artery after cardiac surgery. J Card Surg. 2005 Mar-Apr;20(2):186-8. doi: 10.1111/j.0886-0440.2005.200382q.x. PMID: 15725148. Link <https://pubmed.ncbi.nlm.nih.gov/15725148/>
1145. Oren I. Breakthrough zygomycosis during empirical voriconazole therapy in febrile patients with neutropenia. Clin Infect Dis. 2005 Mar 1;40(5):770-1. doi: 10.1086/427759. PMID: 15714432. Link <https://pubmed.ncbi.nlm.nih.gov/15714432/>
1146. Vigouroux S, Morin O, Moreau P, Méchinaud F, Morineau N, Mahé B, Chevallier P, Guillaume T, Dubruille V, Harousseau JL, Milpied N. Zygomycosis after prolonged use of voriconazole in immunocompromised patients with hematologic disease: attention required. Clin Infect Dis. 2005 Feb 15;40(4):e35-7. doi: 10.1086/427752. Epub 2005 Jan 25. PMID: 15712069. Link <https://pubmed.ncbi.nlm.nih.gov/15712069/>
1147. Ketenci I, Unlü Y, Sentürk M, Tuncer E. Indolent mucormycosis of the sphenoid sinus. Otolaryngol Head Neck Surg. 2005 Feb;132(2):341-2. doi: 10.1016/j.otohns.2004.09.041. PMID: 15692554. Link <https://pubmed.ncbi.nlm.nih.gov/15692554/>
1148. Mater A, Al-Sulaiti G, Johnston DL, Slinger R. A 4-year-old child with leukemia and an enlarging arm lesion. CMAJ. 2005 Feb 1;172(3):332. doi: 10.1503/cmaj.1041231. PMID: 15684115; PMCID: PMC545756. Link <https://pubmed.ncbi.nlm.nih.gov/15684115/>
1149. Kerbaul F, Guidon C, Collart F, Lépidi H, Cayatte B, Bonnet M, Bellezza M, Métras D, Gouin F. Abdominal wall mucormycosis after heart transplantation. J Cardiothorac Vasc Anesth. 2004 Dec;18(6):822-3. doi: 10.1053/j.jvca.2004.08.033. PMID: 15651004 Link <https://pubmed.ncbi.nlm.nih.gov/15651004/>
1150. Bashir G, Shakeel S, Wani T, Kakru DK. Pulmonary pseudallescheriasis in a patient with healed tuberculosis. Mycopathologia. 2004 Oct;158(3):289-91. doi: 10.1007/s11046-004-2224-7. PMID: 15645170. Link <https://pubmed.ncbi.nlm.nih.gov/15645170/>
1151. Dobrilovic N, Wait MA. Pulmonary mucormycosis. Ann Thorac Surg. 2005 Jan;79(1):354. doi: 10.1016/S0003-4975(03)01287-6. PMID: 15620984. Link <https://pubmed.ncbi.nlm.nih.gov/15620984/>
1152. Ayabe T, Matsuzaki Y, Edagawa M, Shimizu T, Hara M, Ninomiya H, Yamashita A, Marutsuka K, Onitsuka T. [Bilateral lower lobectomies for pulmonary mucormycosis]. Kyobu Geka. 2004 Dec;57(13):1185-90. Japanese. PMID: 15609654. Link <https://pubmed.ncbi.nlm.nih.gov/15609654/>
1153. van Dam AP, Pruijm MT, Harinck BI, Gelinck LB, Kuijper EJ. Pneumonia involving Aspergillus and Rhizopus spp. after a near-drowning incident with subsequent Nocardia cyriacigeorgici and N. farcinica coinfection as a late complication. Eur J Clin Microbiol Infect Dis. 2005 Jan;24(1):61-4. doi: 10.1007/s10096-004-1263-9. PMID: 15599783. Link <https://pubmed.ncbi.nlm.nih.gov/15599783/>
1154. Rubin AI, Grossman ME. Bull's-eye cutaneous infarct of zygomycosis: a bedside diagnosis confirmed by touch preparation. J Am Acad Dermatol. 2004 Dec;51(6):996-1001. doi: 10.1016/j.jaad.2004.07.027. PMID: 15583599. Link <https://pubmed.ncbi.nlm.nih.gov/15583599/>
1155. Eicken Jv, Preyer S, Wilhelm H. Potenziell letaler Orbitaprozess [Potentially fatal orbital disorder]. Klin Monbl Augenheilkd. 2004 Nov;221(11):948-52. German. doi: 10.1055/s-2004-813597. PMID: 15562359. Link <https://pubmed.ncbi.nlm.nih.gov/15562359/>
1156. Diven SC, Angel CA, Hawkins HK, Rowen JL, Shattuck KE. Intestinal zygomycosis due to Absidia corymbifera mimicking necrotizing enterocolitis in a preterm neonate. J Perinatol. 2004 Dec;24(12):794-6. doi: 10.1038/sj.jp.7211186. PMID: 15558003. Link <https://pubmed.ncbi.nlm.nih.gov/15558003/>
1157. Mohammed S, Sahoo TP, Jayshree RS, Bapsy PP, Hema S. Sino-oral zygomycosis due to Absidia corymbifera in a patient with acute leukemia. Med Mycol. 2004 Oct;42(5):475-8. doi: 10.1080/13693780412331298848. PMID: 15552649. Link <https://pubmed.ncbi.nlm.nih.gov/15552649/>
1158. Gibbs SD, Herbert KE, McCormack C, Seymour JF, Prince HM. Alemtuzumab: effective monotherapy for simultaneous B-cell chronic lymphocytic leukaemia and Sézary syndrome. Eur J Haematol. 2004 Dec;73(6):447-9. doi: 10.1111/j.1600-0609.2004.00332.x. PMID: 15522069. Link <https://pubmed.ncbi.nlm.nih.gov/15522069/>
1159. Hilal AA, Taj-Aldeen SJ, Mirghani AH. Rhinoorbital mucormycosis secondary to Rhizopus oryzae: a case report and literature review. Ear Nose Throat J. 2004 Aug;83(8):556, 558-60, 562. PMID: 15487636. Link <https://pubmed.ncbi.nlm.nih.gov/15487636/>
1160. Maiorano E, Favia G, Capodiferro S, Montagna MT, Lo Muzio L. Combined mucormycosis and aspergillosis of the oro-sinonasal region in a patient affected by Castleman disease. Virchows Arch. 2005 Jan;446(1):28-33. doi: 10.1007/s00428-004-1126-x. Epub 2004 Oct 5. PMID: 15480762. Link <https://pubmed.ncbi.nlm.nih.gov/15480762/>
1161. Scharf JL, Soliman AM. Chronic rhizopus invasive fungal rhinosinusitis in an immunocompetent host. Laryngoscope. 2004 Sep;114(9):1533-5. doi: 10.1097/00005537-200409000-00005. PMID: 15475776. Link <https://pubmed.ncbi.nlm.nih.gov/15475776/>
1162. Horré R, Jovanić B, Herff S, Marklein G, Zhou H, Heinze I, De Hoog GS, Rüchel R, Schaal KP. Wound infection due to Absidia corymbifera and Candida albicans with fatal outcome. Med Mycol. 2004 Aug;42(4):373-8. doi: 10.1080/1369378032000141426. PMID: 15473364. Link <https://pubmed.ncbi.nlm.nih.gov/15473364/>
1163. Reid VJ, Solnik DL, Daskalakis T, Sheka KP. Management of bronchovascular mucormycosis in a diabetic: a surgical success. Ann Thorac Surg. 2004 Oct;78(4):1449-51. doi: 10.1016/S0003-4975(03)01406-1. PMID: 15464515. Link <https://pubmed.ncbi.nlm.nih.gov/15464515/>
1164. Sehgal A, Raghavendran M, Kumar D, Srivastava A, Dubey D, Kumar A. Rhinocerebral mucormycosis causing basilar artery aneurysm with concomitant fungal colonic perforation in renal allograft recipient: a case report. Transplantation. 2004 Sep 27;78(6):949-50. doi: 10.1097/01.tp.0000129798.22312.1e. PMID: 15385821. Link <https://pubmed.ncbi.nlm.nih.gov/15385821/>
1165. Blin N, Morineau N, Gaillard F, Morin O, Milpied N, Harousseau JL, Moreau P. Disseminated mucormycosis associated with invasive pulmonary aspergillosis in a patient treated for post-transplant high-grade non-Hodgkin's lymphoma. Leuk Lymphoma. 2004 Oct;45(10):2161-3. doi: 10.1080/10428190410001700803. PMID: 15370266. Link <https://pubmed.ncbi.nlm.nih.gov/15370266/>
1166. Basti A, Taylor S, Tschopp M, Sztajzel J. Fatal fulminant myocarditis caused by disseminated mucormycosis. Heart. 2004 Oct;90(10):e60. doi: 10.1136/hrt.2004.038273. PMID: 15367533; PMCID: PMC1768475. Link <https://pubmed.ncbi.nlm.nih.gov/15367533/>
1167. Kobayashi M, Togitani K, Machida H, Uemura Y, Ohtsuki Y, Taguchi H. Molecular polymerase chain reaction diagnosis of pulmonary mucormycosis caused by Cunninghamella bertholletiae. Respirology. 2004 Aug;9(3):397-401. doi: 10.1111/j.1440-1843.2004.00582.x. PMID: 15363015. Link <https://pubmed.ncbi.nlm.nih.gov/15363015/>
1168. Mehta P, Augustson B, Krishnamurthy S, Jacob A, Roy D, Olliff J, Cook M, Craddock C, Mahendra P. Successful allogeneic haematopoietic stem cell transplantation in patients with poor-risk leukaemia and prior invasive fungal infection. Bone Marrow Transplant. 2004 Nov;34(9):825-6. doi: 10.1038/sj.bmt.1704685. PMID: 15361904. Link <https://pubmed.ncbi.nlm.nih.gov/15361904/>
1169. Kordy FN, Al-Mohsen IZ, Hashem F, Almodovar E, Al Hajjar S, Walsh TJ. Successful treatment of a child with posttraumatic necrotizing fasciitis caused by Apophysomyces elegans: case report and review of literature. Pediatr Infect Dis J. 2004 Sep;23(9):877-9. doi: 10.1097/01.inf.0000136870.17071.fd. PMID: 15361732. Link <https://pubmed.ncbi.nlm.nih.gov/15361732/>
1170. Welk B, House AA, Ralph E, Tweedy E, Luke PP. Successful treatment of primary bilateral renal mucormycosis with bilateral nephrectomy. Urology. 2004 Sep;64(3):590. doi: 10.1016/j.urology.2004.04.071. PMID: 15351613. Link <https://pubmed.ncbi.nlm.nih.gov/15351613/>
1171. Chaney S, Gopalan R, Berggren RE. Pulmonary Pseudallescheria boydii infection with cutaneous zygomycosis after near drowning. South Med J. 2004 Jul;97(7):683-7. doi: 10.1097/00007611-200407000-00014. PMID: 15301126. Link <https://pubmed.ncbi.nlm.nih.gov/15301126/>
1172. Droll A, Kunz F, Passweg JR, Michot M. Cerebral mucormycosis in a patient with myelodysplastic syndrome taking corticosteroids. Br J Haematol. 2004 Aug;126(4):441. doi: 10.1111/j.1365-2141.2004.05080.x. PMID: 15287935. Link <https://pubmed.ncbi.nlm.nih.gov/15287935/>
1173. Cady FM, Madory JA, Hoda RS. Polypoid sinonasal lesion in a diabetic patient. Diagn Cytopathol. 2004 Jul;31(1):31-2. doi: 10.1002/dc.10426. PMID: 15236261. Link <https://pubmed.ncbi.nlm.nih.gov/15236261/>
1174. Kerr OA, Bong C, Wallis C, Tidman MJ. Primary cutaneous mucormycosis masquerading as pyoderma gangrenosum. Br J Dermatol. 2004 Jun;150(6):1212-3. doi: 10.1111/j.1365-2133.2004.05826.x. PMID: 15214915. Link <https://pubmed.ncbi.nlm.nih.gov/15214915/>
1175. Nichol PF, Corliss RF, Rajpal S, Helin M, Lund DP. Perforation of the appendix from intestinal mucormycosis in a neonate. J Pediatr Surg. 2004 Jul;39(7):1133-5. doi: 10.1016/j.jpedsurg.2004.03.068. PMID: 15213917. Link <https://pubmed.ncbi.nlm.nih.gov/15213917/>
1176. Mattner F, Weissbrodt H, Strueber M. Two case reports: fatal Absidia corymbifera pulmonary tract infection in the first postoperative phase of a lung transplant patient receiving voriconazole prophylaxis, and transient bronchial Absidia corymbifera colonization in a lung transplant patient. Scand J Infect Dis. 2004;36(4):312-4. doi: 10.1080/00365540410019408. PMID: 15198193. Link <https://pubmed.ncbi.nlm.nih.gov/15198193/>
1177. Wolf O, Gil Z, Leider-Trejo L, Khafif A, Biderman P, Fliss DM. Tracheal mucormycosis presented as an intraluminal soft tissue mass. Head Neck. 2004 Jun;26(6):541-3. doi: 10.1002/hed.20055. PMID: 15162356. Link <https://pubmed.ncbi.nlm.nih.gov/15162356/>
1178. Ortín X, Escoda L, Llorente A, Rodriguez R, Martínez S, Boixadera J, Cabezudo E, Ugarriza A. Cunninghamella bertholletiae infection (mucormycosis) in a patient with acute T-cell lymphoblastic leukemia. Leuk Lymphoma. 2004 Mar;45(3):617-20. doi: 10.1080/1042819031000139693. PMID: 15160928. Link <https://pubmed.ncbi.nlm.nih.gov/15160928/>
1179. Numa WA Jr, Foster PK, Wachholz J, Civantos F, Gomez-Fernandez C, Weed DT. Cutaneous mucormycosis of the head and neck with parotid gland involvement: first report of a case. Ear Nose Throat J. 2004 Apr;83(4):282, 284, 286 passim. PMID: 15147102. Link <https://pubmed.ncbi.nlm.nih.gov/15147102/>
1180. Singh SK, Wadhwa P, Sakhuja V. Isolated bilateral renal mucormycosis. Urology. 2004 May;63(5):979-80. doi: 10.1016/j.urology.2003.11.020. PMID: 15134995. Link <https://pubmed.ncbi.nlm.nih.gov/15134995/>
1181. Quinio D, Karam A, Leroy JP, Moal MC, Bourbigot B, Masure O, Sassolas B, Le Flohic AM. Zygomycosis caused by Cunninghamella bertholletiae in a kidney transplant recipient. Med Mycol. 2004 Apr;42(2):177-80. doi: 10.1080/13693780310001644644. PMID: 15124871. Link <https://pubmed.ncbi.nlm.nih.gov/15124871/>
1182. Siu KL, Lee WH. A rare cause of intestinal perforation in an extreme low birth weight infant--gastrointestinal mucormycosis: a case report. J Perinatol. 2004 May;24(5):319-21. doi: 10.1038/sj.jp.7211090. PMID: 15116129 Link <https://pubmed.ncbi.nlm.nih.gov/15116129/>
1183. Mutsukura K, Tsuboi Y, Imamura A, Fujiki F, Yamada T. [Garcin syndrome in a patient with rhinocerebral mucormycosis]. No To Shinkei. 2004 Mar;56(3):231-5. Japanese. PMID: 15112447. Link <https://pubmed.ncbi.nlm.nih.gov/15112447/>
1184. Takabayashi M, Sakai R, Sakamoto H, Kakinuma M, Iemoto Y, Kanamori H, Ishigatsubo Y. Cutaneous mucormycosis during induction chemotherapy for acute lymphocytic leukemia. Leuk Lymphoma. 2004 Jan;45(1):199-200. doi: 10.1080/1042819031000139675. PMID: 15061222. Link <https://pubmed.ncbi.nlm.nih.gov/15061222/>
1185. Brun AL, Guichard JP, Assouline E, Genauzeau I, Reizine D, Merland JJ. Mucormycose rhinocérébrale [Rhinocerebral mucormycosis]. J Neuroradiol. 2004 Jan;31(1):74-6. French. doi: 10.1016/s0150-9861(04)96884-8. PMID: 15026737. Link <https://pubmed.ncbi.nlm.nih.gov/15026737/>
1186. Azadeh B, McCarthy DO, Dalton A, Campbell F. Gastrointestinal zygomycosis: two case reports. Histopathology. 2004 Mar;44(3):298-300. doi: 10.1111/j.1365-2559.2004.01761.x. PMID: 14987236. Link <https://pubmed.ncbi.nlm.nih.gov/14987236/>
1187. Chassaing N, Valton L, Kany M, Bonnet E, Uro-Coste E, Delisle MB, Bousquet P, Géraud G. Traitement d'un abcès rhino-cérébral mycotique par adjonction d'oxygénothérapie hyperbare [Rhino-cerebral fungal infection successfully treated with supplementary hyperbaric oxygen therapy]. Rev Neurol (Paris). 2003 Dec;159(12):1178-80. French. PMID: 14978421. Link <https://pubmed.ncbi.nlm.nih.gov/14978421/>
1188. Shand JM, Albrecht RM, Burnett HF 3rd, Miyake A. Invasive fungal infection of the midfacial and orbital complex due to Scedosporium apiospermum and mucormycosis. J Oral Maxillofac Surg. 2004 Feb;62(2):231-4. doi: 10.1016/j.joms.2003.04.013. PMID: 14762757. Link <https://pubmed.ncbi.nlm.nih.gov/14762757/>
1189. Benekli M, Crane JK, Conti RR, Kremzier JE, Bidani R. Pulmonary mucormycosis in a patient with chronic obstructive pulmonary disease: diagnosis by fine needle aspiration cytology. J Thorac Cardiovasc Surg. 2004 Feb;127(2):588-9. doi: 10.1016/j.jtcvs.2003.09.042. PMID: 14762378. Link <https://pubmed.ncbi.nlm.nih.gov/14762378/>
1190. Sammassimo S, Mazzotta S, Tozzi M, Gentili S, Lenoci M, Santopietro R, Bucalossi A, Bocchia M, Lauria F. Disseminated mucormycosis in a patient with acute myeloblastic leukemia misdiagnosed as infection by Enterococcus faecium. J Clin Microbiol. 2004 Jan;42(1):487-9. doi: 10.1128/JCM.42.1.487-489.2004. PMID: 14715813; PMCID: PMC321679. Link <https://pubmed.ncbi.nlm.nih.gov/14715813/>
1191. Yasui H, Adachi Y, Ishii Y, Kato Y, Imai K. Mucormycosis as an etiology of cerebral hemorrhage in patients with chronic neutrophilic leukemia. Am J Med. 2003 Dec 1;115(8):674-6. doi: 10.1016/s0002-9343(03)00426-1. PMID: 14656625. Link <https://pubmed.ncbi.nlm.nih.gov/14656625/>
1192. Mok CC, Que TL, Tsui EY, Lam WY. Mucormycosis in systemic lupus erythematosus. Semin Arthritis Rheum. 2003 Oct;33(2):115-24. doi: 10.1016/s0049-0172(03)00081-7. PMID: 14625819. Link <https://pubmed.ncbi.nlm.nih.gov/14625819/>
1193. Leitner C, Hoffmann J, Zerfowski M, Reinert S. Mucormycosis: necrotizing soft tissue lesion of the face. J Oral Maxillofac Surg. 2003 Nov;61(11):1354-8. doi: 10.1016/s0278-2391(03)00740-7. PMID: 14613095. Link <https://pubmed.ncbi.nlm.nih.gov/14613095/>
1194. Boyd AS, Wiser B, Sams HH, King LE. Gangrenous cutaneous mucormycosis in a child with a solid organ transplant: a case report and review of the literature. Pediatr Dermatol. 2003 Sep-Oct;20(5):411-5. doi: 10.1046/j.1525-1470.2003.20508.x. PMID: 14521558. Link <https://pubmed.ncbi.nlm.nih.gov/14521558/>
1195. Serna JH, Wanger A, Dosekun AK. Successful treatment of mucormycosis peritonitis with liposomal amphotericin B in a patient on long-term peritoneal dialysis. Am J Kidney Dis. 2003 Sep;42(3):E14-7. doi: 10.1016/s0272-6386(03)00797-2. PMID: 12955706. Link <https://pubmed.ncbi.nlm.nih.gov/12955706/>
1196. Ladurner R, Brandacher G, Steurer W, Schneeberger S, Bösmüller C, Freund MC, Kreczy A, Königsrainer A, Margreiter R. Lessons to be learned from a complicated case of rhino-cerebral mucormycosis in a renal allograft recipient. Transpl Int. 2003 Dec;16(12):885-9. doi: 10.1007/s00147-003-0648-5. Epub 2003 Aug 27. PMID: 12942168. Link <https://pubmed.ncbi.nlm.nih.gov/12942168/>
1197. Kofteridis DP, Karabekios S, Panagiotides JG, Bizakis J, Kyrmizakis D, Saridaki Z, Gikas A. Successful treatment of rhinocerebral mucormycosis with liposomal amphotericin B and surgery in two diabetic patients with renal dysfunction. J Chemother. 2003 Jun;15(3):282-6. doi: 10.1179/joc.2003.15.3.282. PMID: 12868556. Link <https://pubmed.ncbi.nlm.nih.gov/12868556/>
1198. Clark FL, Batra RS, Gladstone HB. Mohs micrographic surgery as an alternative treatment method for cutaneous mucormycosis. Dermatol Surg. 2003 Aug;29(8):882-5. doi: 10.1046/j.1524-4725.2003.29240.x. PMID: 12859396. Link <https://pubmed.ncbi.nlm.nih.gov/12859396/>
1199. Lucas JB, Salyer RD, Watson DW. Gangrenous primary cutaneous mucormycosis of the scalp in an iatrogenically immunosuppressed trauma patient. Otolaryngol Head Neck Surg. 2003 Jun;128(6):912-4. doi: 10.1016/s0194-5998(03)00463-7. PMID: 12825050. Link <https://pubmed.ncbi.nlm.nih.gov/12825050/>
1200. Thami GP, Kaur S, Bawa AS, Chander J, Mohan H, Bedi MS. Post-surgical zygomycotic necrotizing subcutaneous infection caused by Absidia corymbifera. Clin Exp Dermatol. 2003 May;28(3):251-3. doi: 10.1046/j.1365-2230.2003.01244.x. PMID: 12780704. Link <https://pubmed.ncbi.nlm.nih.gov/12780704/>
1201. Aboutanos MB, Joshi M, Scalea TM. Isolated pulmonary mucormycosis in a patient with multiple injuries: a case presentation and review of the literature. J Trauma. 2003 May;54(5):1016-9. doi: 10.1097/01.TA.0000023169.90650.6B. PMID: 12777921. Link <https://pubmed.ncbi.nlm.nih.gov/12777921/>
1202. Tobón AM, Arango M, Fernández D, Restrepo A. Mucormycosis (zygomycosis) in a heart-kidney transplant recipient: recovery after posaconazole therapy. Clin Infect Dis. 2003 Jun 1;36(11):1488-91. doi: 10.1086/375075. Epub 2003 May 16. PMID: 12766845. Link <https://pubmed.ncbi.nlm.nih.gov/12766845/>
1203. Hanse MC, Nijssen PC. Unilateral palsy of all cranial nerves (Garcin syndrome) in a patient with rhinocerebral mucormycosis. J Neurol. 2003 Apr;250(4):506-7. doi: 10.1007/s00415-003-1019-y. PMID: 12760392. Link <https://pubmed.ncbi.nlm.nih.gov/12760392/>
1204. Paydas S, Yavuz S, Disel U, Yildirim M, Besen A, Sahin B, Gumurdulu D, Yuce E. Mucormycosis of the tongue in a patient with acute lymphoblastic leukemia: a possible relation with use of a tongue depressor. Am J Med. 2003 May;114(7):618-20. doi: 10.1016/s0002-9343(03)00092-5. PMID: 12753891. Link <https://pubmed.ncbi.nlm.nih.gov/12753891/>
1205. Pandit SR, Raheem MA. Mucormycosis in acute myeloid leukaemia. Br J Haematol. 2003 May;121(3):382. doi: 10.1046/j.1365-2141.2003.04218.x. PMID: 12716358. Link <https://pubmed.ncbi.nlm.nih.gov/12716358/>
1206. Kumar A, Khilnani GC, Aggarwal S, Kumar S, Banerjee U, Xess I. Primary cutaneous mucormycosis in an immunocompetent host: report of a case. Surg Today. 2003;33(4):319-22. doi: 10.1007/s005950300073. PMID: 12707834. Link <https://pubmed.ncbi.nlm.nih.gov/12707834/>
1207. Keogh CF, Brown JA, Phillips P, Cooperberg PL. Renal mucormycosis in an AIDS patient: imaging features and pathologic correlation. AJR Am J Roentgenol. 2003 May;180(5):1278-80. doi: 10.2214/ajr.180.5.1801278. PMID: 12704037. Link <https://pubmed.ncbi.nlm.nih.gov/12704037/>
1208. Minz M, Sharma A, Kashyap R, Udgiri N, Heer M, Kumar V, Vaiphei K. Isolated renal allograft arterial mucormycosis: an extremely rare complication. Nephrol Dial Transplant. 2003 May;18(5):1034-5. doi: 10.1093/ndt/gfg086. PMID: 12686694. Link <https://pubmed.ncbi.nlm.nih.gov/12686694/>
1209. Merlino J, Temes RT, Joste NE, Gill IS. Invasive pulmonary mucormycosis with ruptured pseudoaneurysm. Ann Thorac Surg. 2003 Apr;75(4):1332. doi: 10.1016/s0003-4975(02)04066-3. PMID: 12683595. Link <https://pubmed.ncbi.nlm.nih.gov/12683595/>
1210. Lenane P, Keane CO, Loughlin SO. Mucor mycosis infection presenting as a non-healing ulcer in an immunocompromised patient. Clin Exp Dermatol. 2003 Mar;28(2):157-9. doi: 10.1046/j.1365-2230.2003.01187.x. PMID: 12653703. Link <https://pubmed.ncbi.nlm.nih.gov/12653703/>
1211. Hamilton JF, Bartkowski HB, Rock JP. Management of CNS mucormycosis in the pediatric patient. Pediatr Neurosurg. 2003 Apr;38(4):212-5. doi: 10.1159/000069101. PMID: 12646741. Link <https://pubmed.ncbi.nlm.nih.gov/12646741/>
1212. Larsen K, von Buchwald C, Ellefsen B, Francis D. Unexpected expansive paranasal sinus mucormycosis. ORL J Otorhinolaryngol Relat Spec. 2003 Jan-Feb;65(1):57-60. doi: 10.1159/000068657. PMID: 12624509. Link <https://pubmed.ncbi.nlm.nih.gov/12624509/>
1213. Wang J, Harvey CM, Calhoun JH, Yin LY, Mader JT. Systemic Apophysomyces elegans after trauma: case report and literature review. Surg Infect (Larchmt). 2002 Fall;3(3):283-9. doi: 10.1089/109629602761624243. PMID: 12542929. Link <https://pubmed.ncbi.nlm.nih.gov/12542929/>
1214. Spira A, Brecher S, Karlinsky J. Pulmonary mucormycosis in the setting of chronic obstructive pulmonary disease. A case report and review of the literature. Respiration. 2002;69(6):560-3. doi: 10.1159/000066467. PMID: 12457013. Link <https://pubmed.ncbi.nlm.nih.gov/12457013/>
1215. Park YS, Lee JD, Kim TH, Joo YH, Lee JH, Lee TS, Kim EK. Gastric mucormycosis. Gastrointest Endosc. 2002 Dec;56(6):904-5. doi: 10.1067/mge.2002.128699. PMID: 12447307. Link <https://pubmed.ncbi.nlm.nih.gov/12447307/>
1216. Oh D, Notrica D. Primary cutaneous mucormycosis in infants and neonates: case report and review of the literature. J Pediatr Surg. 2002 Nov;37(11):1607-11. doi: 10.1053/jpsu.2002.36193. PMID: 12407548. Link <https://pubmed.ncbi.nlm.nih.gov/12407548/>
1217. Virally ML, Riveline JP, Virally J, Chevojon P, Regnard JF, Belmekki A, Devidas A. Pulmonary mucormycosis in a diabetic patient with HIV. Diabetes Care. 2002 Nov;25(11):2105. doi: 10.2337/diacare.25.11.2105. PMID: 12401766. Link <https://pubmed.ncbi.nlm.nih.gov/12401766/>
1218. Alsuwaida K. Primary cutaneous mucormycosis complicating the use of adhesive tape to secure the endotracheal tube. Can J Anaesth. 2002 Oct;49(8):880-2. doi: 10.1007/BF03017426. PMID: 12374722. Link <https://pubmed.ncbi.nlm.nih.gov/12374722/>
1219. Zhang R, Zhang JW, Szerlip HM. Endocarditis and hemorrhagic stroke caused by Cunninghamella bertholletiae infection after kidney transplantation. Am J Kidney Dis. 2002 Oct;40(4):842-6. doi: 10.1053/ajkd.2002.35698. PMID: 12324922. Link <https://pubmed.ncbi.nlm.nih.gov/12324922/>
1220. Zaizen Y, Ohtsu T. Successful treatment of pulmonary mucormycosis, a rare pulmonary fungal infection, in a patient with diabetes mellitus. J Thorac Cardiovasc Surg. 2002 Oct;124(4):838-40. doi: 10.1067/mtc.2002.125283. PMID: 12324746. Link <https://pubmed.ncbi.nlm.nih.gov/12324746/>
1221. Voitl P, Scheibenpflug C, Weber T, Janata O, Rokitansky AM. Combined antifungal treatment of visceral mucormycosis with caspofungin and liposomal amphotericin B. Eur J Clin Microbiol Infect Dis. 2002 Aug;21(8):632-4. doi: 10.1007/s10096-002-0781-6. Epub 2002 Aug 15. PMID: 12226700. Link <https://pubmed.ncbi.nlm.nih.gov/12226700/>
1222. Wehl G, Hoegler W, Kropshofer G, Meister B, Fink FM, Heitger A. Rhinocerebral mucormycosis in a boy with recurrent acute lymphoblastic leukemia: long-term survival with systemic antifungal treatment. J Pediatr Hematol Oncol. 2002 Aug-Sep;24(6):492-4. doi: 10.1097/00043426-200208000-00017. PMID: 12218600. Link <https://pubmed.ncbi.nlm.nih.gov/12218600/>
1223. Lesueur BW, Warschaw K, Fredrikson L. Necrotizing cellulitis caused by Apophysomyces elegans at a patch test site. Am J Contact Dermat. 2002 Sep;13(3):140-2. PMID: 12165933. Link <https://pubmed.ncbi.nlm.nih.gov/12165933/>
1224. Blair JE, Fredrikson LJ, Pockaj BA, Lucaire CS. Locally invasive cutaneous Apophysomyces elegans infection acquired from snapdragon patch test. Mayo Clin Proc. 2002 Jul;77(7):717-20. doi: 10.4065/77.7.717. PMID: 12108611. Link <https://pubmed.ncbi.nlm.nih.gov/12108611/>
1225. Hunter AJ, Bryant RE. Abdominal wall mucormycosis successfully treated with amphotericin and itraconazole. J Infect. 2002 Apr;44(3):203-4. doi: 10.1053/jinf.2002.0972. PMID: 12099754. Link <https://pubmed.ncbi.nlm.nih.gov/12099754/>
1226. Lee DG, Choi JH, Choi SM, Yoo JH, Kim YJ, Min CK, Lee S, Kim DW, Shin WS, Kim CC. Two cases of disseminated mucormycosis in patients following allogeneic bone marrow transplantation. J Korean Med Sci. 2002 Jun;17(3):403-6. doi: 10.3346/jkms.2002.17.3.403. PMID: 12068148; PMCID: PMC3054875. Link <https://pubmed.ncbi.nlm.nih.gov/12068148/>
1227. Rumboldt Z, Castillo M. Indolent intracranial mucormycosis: case report. AJNR Am J Neuroradiol. 2002 Jun-Jul;23(6):932-4. PMID: 12063219; PMCID: PMC7976923. Link <https://pubmed.ncbi.nlm.nih.gov/12063219/>
1228. Dökmetaş HS, Canbay E, Yilmaz S, Elaldi N, Topalkara A, Oztoprak I, Yildiz E. Diabetic ketoacidosis and rhino-orbital mucormycosis. Diabetes Res Clin Pract. 2002 Aug;57(2):139-42. doi: 10.1016/s0168-8227(02)00021-9. PMID: 12062859. Link <https://pubmed.ncbi.nlm.nih.gov/12062859/>
1229. Donado-Uña JR, Díaz-Hellín V, López-Encuentra A, Echave-Sustaeta JM. Persistent cavitations in pulmonary mucormycosis after apparently successful amphotericin B. Eur J Cardiothorac Surg. 2002 May;21(5):940-2. doi: 10.1016/s1010-7940(02)00081-7. PMID: 12062298. Link <https://pubmed.ncbi.nlm.nih.gov/12062298/>
1230. Vaideeswar P. Fatal haemoptysis due to mucormycotic intrapulmonary arterial aneurysm. Int J Cardiol. 2002 Jun;83(3):273-4. doi: 10.1016/s0167-5273(02)00050-5. PMID: 12036534. Link <https://pubmed.ncbi.nlm.nih.gov/12036534/>
1231. Tedeschi A, Montillo M, Cairoli R, Marenco P, Cafro A, Oreste P, Nosari A, Morra E. Prior invasive pulmonary and cerebellar mucormycosis is not a primary contraindication to perform an autologous stem cell transplatation in leukemia. Leuk Lymphoma. 2002 Mar;43(3):657-9. doi: 10.1080/10428190290012236. PMID: 12002776. Link <https://pubmed.ncbi.nlm.nih.gov/12002776/>
1232. Yamauchi T, Misaki H, Arai H, Iwasaki H, Naiki H, Ueda T. An autopsy case of disseminated mucormycosis in a neutropenic patient receiving chemotherapy for the underlying solid malignancy. J Infect Chemother. 2002 Mar;8(1):103-5. doi: 10.1007/s101560200015. PMID: 11957129. Link <https://pubmed.ncbi.nlm.nih.gov/11957129/>
1233. Paulo De Oliveira JE, Milech A. A fatal case of gastric mucormycosis and diabetic ketoacidosis. Endocr Pract. 2002 Jan-Feb;8(1):44-6. doi: 10.4158/EP.8.1.44. PMID: 11939759. Link <https://pubmed.ncbi.nlm.nih.gov/11939759/>
1234. Mondy KE, Haughey B, Custer PL, Wippold FJ 2nd, Ritchie DJ, Mundy LM. Rhinocerebral mucormycosis in the era of lipid-based amphotericin B: case report and literature review. Pharmacotherapy. 2002 Apr;22(4):519-26. doi: 10.1592/phco.22.7.519.33679. PMID: 11939688. Link <https://pubmed.ncbi.nlm.nih.gov/11939688/>
1235. Ma B, Seymour JF, Januszewicz H, Slavin MA. Cure of pulmonary Rhizomucor pusillus infection in a patient with hairy-cell leukemia: role of liposomal amphotericin B and GM-CSF. Leuk Lymphoma. 2001 Nov-Dec;42(6):1393-9. doi: 10.3109/10428190109097768. PMID: 11911424. Link <https://pubmed.ncbi.nlm.nih.gov/11911424/>
1236. Bienfang DC, Karluk D. Case records of the Massachusetts General Hospital. Weekly clinicopathological exercises. Case 9-2002. An 80-year-old woman with sudden unilateral blindness. N Engl J Med. 2002 Mar 21;346(12):924-9. doi: 10.1056/NEJMcpc020009. PMID: 11907293. Link <https://pubmed.ncbi.nlm.nih.gov/11907293/>
1237. Tryfon S, Stanopoulos I, Kakavelas E, Nikolaidou A, Kioumis I. Rhinocerebral mucormycosis in a patient with latent diabetes mellitus: a case report. J Oral Maxillofac Surg. 2002 Mar;60(3):328-30. doi: 10.1053/joms.2002.30600. PMID: 11887153. Link <https://pubmed.ncbi.nlm.nih.gov/11887153/>
1238. Jiménez C, Lumbreras C, Aguado JM, Loinaz C, Paseiro G, Andrés A, Morales JM, Sánchez G, García I, del Palacio A, Moreno E. Successful treatment of mucor infection after liver or pancreas-kidney transplantation. Transplantation. 2002 Feb 15;73(3):476-80. doi: 10.1097/00007890-200202150-00026. PMID: 11884949. Link <https://pubmed.ncbi.nlm.nih.gov/11884949/>
1239. Romano C, Miracco C, Massai L, Piane R, Alessandrini C, Petrini C, Luzi P. Case report. Fatal rhinocerebral zygomycosis due to Rhizopus oryzae. Mycoses. 2002 Feb;45(1-2):45-9. PMID: 11856437. Link <https://pubmed.ncbi.nlm.nih.gov/11856437/>
1240. Sato M, Gemma H, Sano T, Ono T, Atsumi E, Ito I, Chida K, Nakamura H. [Pulmonary mucormycosis caused by Cunninghamella bertholletiae in a non-immunocompromised woman]. Nihon Kokyuki Gakkai Zasshi. 2001 Oct;39(10):758-62. Japanese. PMID: 11828731. Link <https://pubmed.ncbi.nlm.nih.gov/11828731/>
1241. Cloughley R, Kelehan J, Corbett-Feeney G, Murray M, Callaghan J, Regan P, Cormican M. Soft tissue infection with Absidia corymbifera in a patient with idiopathic aplastic anemia. J Clin Microbiol. 2002 Feb;40(2):725-7. doi: 10.1128/JCM.40.2.725-727.2002. PMID: 11826008; PMCID: PMC153381. Link <https://pubmed.ncbi.nlm.nih.gov/11826008/>
1242. Chkhotua A, Yussim A, Tovar A, Weinberger M, Sobolev V, Bar-Nathan N, Shaharabani E, Shapira Z, Mor E. Mucormycosis of the renal allograft: case report and review of the literature. Transpl Int. 2001 Dec;14(6):438-41. doi: 10.1007/s001470100010. PMID: 1179304 Link <https://pubmed.ncbi.nlm.nih.gov/11793042/>
1243. Vera A, Hubscher SG, McMaster P, Buckels JA. Invasive gastrointestinal zygomycosis in a liver transplant recipient: case report. Transplantation. 2002 Jan 15;73(1):145-7. doi: 10.1097/00007890-200201150-00027. PMID: 11792995. Link <https://pubmed.ncbi.nlm.nih.gov/11792995/>
1244. Oliveira V, Costa A. Hematoma cerebral causado por mucormicosis [Cerebral hematoma caused by mucormycosis]. Rev Neurol. 2001 Nov 16-30;33(10):951-3. Spanish. PMID: 11785007. Link <https://pubmed.ncbi.nlm.nih.gov/11785007/>
1245. Lari AR, Kanjoor JR, Vulvoda M, Katchy KC, Khan ZU. Orbital reconstruction following sino-nasal mucormycosis. Br J Plast Surg. 2002 Jan;55(1):72-5. doi: 10.1054/bjps.2001.3725. PMID: 11783974. Link <https://pubmed.ncbi.nlm.nih.gov/11783974/>
1246. Sykes LM, Sukha A. Potential risk of serious oral infections in the diabetic patient: a clinical report. J Prosthet Dent. 2001 Dec;86(6):569-73. doi: 10.1067/mpr.2001.120200. PMID: 11753304. Link <https://pubmed.ncbi.nlm.nih.gov/11753304/>
1247. Cagatay AA, Oncü SS, Calangu SS, Yildirmak TT, Ozsüt HH, Eraksoy HH. Rhinocerebral mucormycosis treated with 32 gram liposomal amphotericin B and incomplete surgery: a case report. BMC Infect Dis. 2001;1:22. doi: 10.1186/1471-2334-1-22. Epub 2001 Nov 23. PMID: 11737868; PMCID: PMC60655. Link <https://pubmed.ncbi.nlm.nih.gov/11737868/>
1248. Lee E, Vershvovsky Y, Miller F, Waltzer W, Suh H, Nord EP. Combined medical surgical therapy for pulmonary mucormycosis in a diabetic renal allograft recipient. Am J Kidney Dis. 2001 Dec;38(6):E37. doi: 10.1053/ajkd.2001.29293. PMID: 11728997. Link <https://pubmed.ncbi.nlm.nih.gov/11728997/>
1249. Kobayashi M, Hiruma M, Matsushita A, Kawai M, Ogawa H, Udagawa S. Cutaneous zygomycosis: a case report and review of Japanese reports. Mycoses. 2001;44(7-8):311-5. PMID: 11714067. Link <https://pubmed.ncbi.nlm.nih.gov/11714067/>
1250. Mileshkin L, Slavin M, Seymour JF, McKenzie A. Successful treatment of rhinocerebral zygomycosis using liposomal nystatin. Leuk Lymphoma. 2001 Sep-Oct;42(5):1119-23. doi: 10.3109/10428190109097734. PMID: 11697631. Link <https://pubmed.ncbi.nlm.nih.gov/11697631/>
1251. Sharma RR, Pawar SJ, Delmendo A, Lad SD, Athale SD. Fatal rhino-orbito-cerebral mucormycosis in an apparently normal host: case report and literature review. J Clin Neurosci. 2001 Nov;8(6):583-6. doi: 10.1054/jocn.2000.0818. PMID: 11683615. Link <https://pubmed.ncbi.nlm.nih.gov/11683615/>
1252. Maddox L, Long GD, Vredenburgh JJ, Folz RJ. Rhizopus presenting as an endobronchial obstruction following bone marrow transplant. Bone Marrow Transplant. 2001 Sep;28(6):634-6. doi: 10.1038/sj.bmt.1703191. PMID: 11607783. Link <https://pubmed.ncbi.nlm.nih.gov/11607783/>
1253. Mata-Essayag S, Magaldi S, de Capriles CH, Henao L, Garrido L, Pacillo V. Mucor indicus necrotizing fasciitis. Int J Dermatol. 2001 Jun;40(6):406-8. doi: 10.1046/j.1365-4362.2001.01246-3.x. PMID: 11589747. Link <https://pubmed.ncbi.nlm.nih.gov/11589747/>
1254. Mandava P, Chaljub G, Patterson K, Hollingsworth JW. MR imaging of cavernous sinus invasion by mucormycosis: a case study. Clin Neurol Neurosurg. 2001 Jul;103(2):101-4. doi: 10.1016/s0303-8467(01)00122-6. PMID: 11516553. Link <https://pubmed.ncbi.nlm.nih.gov/11516553/>
1255. Sobel JD. Vaginal mucormycosis: a case report. Infect Dis Obstet Gynecol. 2001;9(2):117-8. doi: 10.1155/S1064744901000205. PMID: 11495552; PMCID: PMC1784639. Link <https://pubmed.ncbi.nlm.nih.gov/11495552/>
1256. Mani NB, Sood BP, Suri S, Vasishta RK. Mucormycosis of rectum - computed tomography findings. Clin Radiol. 2001 Aug;56(8):680-2. doi: 10.1053/crad.1999.0456. PMID: 11467873. Link <https://pubmed.ncbi.nlm.nih.gov/11467873/>
1257. Narain S, Mitra M, Barton RC, Evans EG, Hutchinson C. Post-traumatic fungal keratitis caused by Absidia corymbifera, with successful medical treatment. Eye (Lond). 2001 Jun;15(Pt 3):352-3. doi: 10.1038/eye.2001.119. PMID: 11450745. Link <https://pubmed.ncbi.nlm.nih.gov/11450745/>
1258. del Río Pérez O, Santín Cerezales M, Mañós M, Rufí Rigau G, Gudiol Munté F. Mucormicosis: una infección clásica con una alta mortalidad. Presentación de 5 casos [Mucormycosis: a classical infection with a high mortality rate. Report of 5 cases]. Rev Clin Esp. 2001 Apr;201(4):184-7. Spanish. PMID: 11447902. Link <https://pubmed.ncbi.nlm.nih.gov/11447902/>
1259. Garey KW, Pendland SL, Huynh VT, Bunch TH, Jensen GM, Pursell KJ. Cunninghamella bertholletiae infection in a bone marrow transplant patient: amphotericin lung penetration, MIC determinations, and review of the literature. Pharmacotherapy. 2001 Jul;21(7):855-60. doi: 10.1592/phco.21.9.855.34560. PMID: 11444582. Link <https://pubmed.ncbi.nlm.nih.gov/11444582/>
1260. Hejny C, Kerrison JB, Newman NJ, Stone CM. Rhino-orbital mucormycosis in a patient with acquired immunodeficiency syndrome (AIDS) and neutropenia. Am J Ophthalmol. 2001 Jul;132(1):111-2. doi: 10.1016/s0002-9394(00)00933-8. PMID: 11438066. Link <https://pubmed.ncbi.nlm.nih.gov/11438066/>
1261. Marcó del Pont J, De Cicco L, Gallo G, Llera J, De Santibanez E, D'agostino D. Hepatic arterial thrombosis due to Mucor species in a child following orthotopic liver transplantation. Transpl Infect Dis. 2000 Mar;2(1):33-5. doi: 10.1034/j.1399-3062.2000.020107.x. PMID: 11429008. Link <https://pubmed.ncbi.nlm.nih.gov/11429008/>
1262. Pérez Fernández CA, Armengot Carceller M, Alba García JR, Montero Balaguer B, Ballester E, Basterra Alegría J. Colonización benigna sinusal por mucor asociada a desviación septal [Benign sinusal mucor colonization in association with septal deviation]. Acta Otorrinolaringol Esp. 2001 Mar;52(2):157-61. Spanish. doi: 10.1016/s0001-6519(01)78192-7. PMID: 11428273. Link <https://pubmed.ncbi.nlm.nih.gov/11428273/>
1263. Ju JH, Park HS, Shin MJ, Yang CW, Kim YS, Choi YJ, Song HJ, Kim SW, Chung IS, Bang BK. Successful treatment of massive lower gastrointestinal bleeding caused by mixed infection of cytomegalovirus and mucormycosis in a renal transplant recipient. Am J Nephrol. 2001 May-Jun;21(3):232-6. doi: 10.1159/000046253. PMID: 11423694. Link <https://pubmed.ncbi.nlm.nih.gov/11423694/>
1264. Ribeiro NF, Cousin GC, Wilson GE, Butterworth DM, Woodwards RT. Lethal invasive mucormycosis: case report and recommendations for treatment. Int J Oral Maxillofac Surg. 2001 Apr;30(2):156-9. doi: 10.1054/ijom.2000.0010. PMID: 11405452. Link <https://pubmed.ncbi.nlm.nih.gov/11405452/>
1265. Kim J, Fortson JK, Cook HE. A fatal outcome from rhinocerebral mucormycosis after dental extractions: a case report. J Oral Maxillofac Surg. 2001 Jun;59(6):693-7. doi: 10.1053/joms.2001.23407. PMID: 11381399. Link <https://pubmed.ncbi.nlm.nih.gov/11381399/>
1266. Bakshi NA, Volk EE. Pulmonary mucormycosis diagnosed by fine needle aspiration cytology. A case report. Acta Cytol. 2001 May-Jun;45(3):411-4. doi: 10.1159/000327640. PMID: 11393076. Link <https://pubmed.ncbi.nlm.nih.gov/11393076/>
1267. Garcia-Diaz JB, Palau L, Pankey GA. Resolution of rhinocerebral zygomycosis associated with adjuvant administration of granulocyte-macrophage colony-stimulating factor. Clin Infect Dis. 2001 Jun 15;32(12):e145-50. doi: 10.1086/320767. Epub 2001 May 7. PMID: 11360225. Link <https://pubmed.ncbi.nlm.nih.gov/11360225/>
1268. Björkholm M, Runarsson G, Celsing F, Kalin M, Petrini B, Engervall P. Liposomal amphotericin B and surgery in the successful treatment of invasive pulmonary mucormycosis in a patient with acute T-lymphoblastic leukemia. Scand J Infect Dis. 2001;33(4):316-9. doi: 10.1080/003655401300077469. PMID: 11345227. Link <https://pubmed.ncbi.nlm.nih.gov/11345227/>
1269. Goh AS, Francis IC, Kappagoda MB, Filipic M. Orbital inflammation in a patient with extrascleral spread of choroidal malignant melanoma. Clin Exp Ophthalmol. 2001 Apr;29(2):97-9. doi: 10.1046/j.1442-9071.2001.d01-14.x. PMID: 11341456. Link <https://pubmed.ncbi.nlm.nih.gov/11341456/>
1270. Garau M, Sánchez-Alor G, Santos-Briz A, Tena D, González-Escalada A, del Palacio A. Infección de herida quirúrgica en paciente trasplantado [Infection of the surgical wound in a transplanted patient]. Enferm Infecc Microbiol Clin. 2001 Mar;19(3):127-9. Spanish. doi: 10.1016/s0213-005x(01)72582-7. PMID: 11333590. Link <https://pubmed.ncbi.nlm.nih.gov/11333590/>
1271. Lerchenmüller C, Göner M, Büchner T, Berdel WE. Rhinocerebral zygomycosis in a patient with acute lymphoblastic leukemia. Ann Oncol. 2001 Mar;12(3):415-9. doi: 10.1023/a:1011119018112. PMID: 11332157. Link <https://pubmed.ncbi.nlm.nih.gov/11332157/>
1272. Ryan M, Yeo S, Maguire A, Webb D, O'Marcaigh A, McDermott M, Butler K, O'Meara A. Rhinocerebral zygomycosis in childhood acute lymphoblastic leukaemia. Eur J Pediatr. 2001 Apr;160(4):235-8. doi: 10.1007/s004310000703. PMID: 11317646. Link <https://pubmed.ncbi.nlm.nih.gov/11317646/>
1273. Castillo L, Hofman V, Bétis F, Piche M, Roger PM, Santini J, Hofman P. Longterm survival in acute rhinocerebral mucormycosis with giant cell arteritis and foreign body granulomas. Pathol Res Pract. 2001;197(3):199-203. doi: 10.1078/0344-0338-00034. PMID: 11314785. Link <https://pubmed.ncbi.nlm.nih.gov/11314785/>
1274. Page R, Gardam DJ, Heath CH. Severe cutaneous mucormycosis (Zygomycosis) due to Apophysomyces elegans. ANZ J Surg. 2001 Mar;71(3):184-6. doi: 10.1046/j.1440-1622.2001.02054.x. PMID: 11277150. Link <https://pubmed.ncbi.nlm.nih.gov/11277150/>
1275. Garcia-Covarrubias L, Bartlett R, Barratt DM, Wassermann RJ. Rhino-orbitocerebral mucormycosis attributable to Apophysomyces elegans in an immunocompetent individual: case report and review of the literature. J Trauma. 2001 Feb;50(2):353-7. doi: 10.1097/00005373-200102000-00027. PMID: 11242306. Link <https://pubmed.ncbi.nlm.nih.gov/11242306/>
1276. Nevitt PC, Das Narla L, Hingsbergen EA. Mucormycosis resulting in a pseudoaneurysm in the spleen. Pediatr Radiol. 2001 Feb;31(2):115-6. doi: 10.1007/s002470000383. PMID: 11214679. Link <https://pubmed.ncbi.nlm.nih.gov/11214679/>
1277. Pelton RW, Peterson EA, Patel BC, Davis K. Successful treatment of rhino-orbital mucormycosis without exenteration: the use of multiple treatment modalities. Ophthalmic Plast Reconstr Surg. 2001 Jan;17(1):62-6. doi: 10.1097/00002341-200101000-00012. PMID: 11206749. Link <https://pubmed.ncbi.nlm.nih.gov/11206749/>
1278. Bhansali A, Sharma A, Kashyap A, Gupta A, Dash RJ. Mucor endophthalmitis. Acta Ophthalmol Scand. 2001 Feb;79(1):88-90. doi: 10.1034/j.1600-0420.2001.079001088.x. PMID: 11167298. Link <https://pubmed.ncbi.nlm.nih.gov/11167298/>
1279. Davel G, Featherston P, Fernández A, Abrantes R, Canteros C, Rodero L, Sztern C, Perrotta D. Maxillary sinusitis caused by Actinomucor elegans. J Clin Microbiol. 2001 Feb;39(2):740-2. doi: 10.1128/JCM.39.2.740-742.2001. PMID: 11158140; PMCID: PMC87809. Link <https://pubmed.ncbi.nlm.nih.gov/11158140/>
1280. Holtom PD, Obuch AB, Ahlmann ER, Shepherd LE, Patzakis MJ. Mucormycosis of the tibia: a case report and review of the literature. Clin Orthop Relat Res. 2000 Dec;(381):222-8. PMID: 11127659. Link <https://pubmed.ncbi.nlm.nih.gov/11127659/>
[truncated: 7,587 more chars]
